# Identifying the tumor immune microenvironment-associated prognostic genes for prostate cancer

**DOI:** 10.1007/s12672-023-00856-3

**Published:** 2024-02-20

**Authors:** Shi Zong, Ji Gao

**Affiliations:** Department of Urology, Union Hospital of Jilin University, No.126, Xian Tai Road, Chang Chun, 130021 China

**Keywords:** Tumor microenvironment, Prostate cancer, Immune infiltration

## Abstract

**Purpose:**

This study aimed to explore novel tumor immune microenvironment (TIME)-associated biomarkers in prostate adenocarcinoma (PRAD).

**Methods:**

PRAD RNA-sequencing data were obtained from UCSC Xena database as the training dataset. The ESTIMATE package was used to evaluate stromal, immune, and tumor purity scores. Differentially expressed genes (DEGs) related to TIME were screened using the immune and stromal scores. Gene functions were analyzed using DAVID. The LASSO method was performed to screen prognostic TIME-related genes. Kaplan–Meier curves were used to evaluate the prognosis of samples. The correlation between the screened genes and immune cell infiltration was explored using Tumor IMmune Estimation Resource. The GSE70768 dataset from the Gene Expression Omnibus was used to validate the expression of the screened genes.

**Results:**

The ESTIMATE results revealed that high immune, stromal, and ESTIMATE scores and low tumor purity had better prognoses. Function analysis indicated that DEGs are involved in the cytokine–cytokine receptor interaction signaling pathway. In TIME-related DEGs, *METTL7B*, *HOXB8*, and *TREM1* were closely related to the prognosis. Samples with low expression levels of *METTL7B*, *HOXB8*, and *TREM1* had better survival times. Similarly, both the validation dataset and qRT-PCR suggested that *METTL7B*, *HOXB8*, and *TREM1* were significantly decreased. The three genes showed a positive correlation with immune infiltration.

**Conclusions:**

This study identified three TIME-related genes, namely, *METTL7B*, *HOXB8*, and *TREM1*, which correlated with the prognosis of patients with PRAD. Targeting the TIME-related genes might have important clinical implications when making decisions for immunotherapy in PRAD.

**Supplementary Information:**

The online version contains supplementary material available at 10.1007/s12672-023-00856-3.

## Introduction

Prostate adenocarcinoma (PRAD) remains the leading cause of cancer related mortality among men in the United States [1]. Despite advances in clinical care, mortality rates remain high, indicating a need for better understanding of the factors influencing PRAD prognosis and treatment response [2, 3].

Recent evidence suggests that PRAD prognosis is heavily dependent on the tumor microenvironment [4]. The tumor immune microenvironment (TIME), comprised of extracellular matrix, stromal cells, and other tumor associated cells, can modulate the tumor’s response to therapy and can influence the progression of the tumor [5]. TIME has been reported to profoundly influence the growth and metastasis of cancer. It affects prognosis, tumor growth, and treatment response through a variety of mechanisms [6]. The composition of the tumor microenvironment can affect prognosis by influencing the proliferation, metastasis, and drug resistance of cancer cells [7]. Factors like the abundance of immune cells, angiogenesis (new blood vessel formation), and cytokines (types of proteins) released by the environment can either inhibit or promote the growth of cancer cells [8]. These signals can also have a large impact on how a patient responds to treatment; for example, certain treatments may be targeted more directly at immunoprivileged environments, and certain drugs tested in preclinical trials may not reach the tumor due to an immunosuppressive microenvironment [9]. In terms of tumor growth, TIME affect the rate of metastasis, and provide signals for cell growth, movement, and survival [10]. TIME could also be a source of genetic mutations, which can lead to the selection of drug resistant tumor cells [11]. Additionally, the presence of certain immune cells can influence the growth and progression of tumors [12]. Finally, the microenvironment can also influence response to treatments. Factors like hypoxia (low oxygen) or the immune cell composition can affect the effectiveness of certain therapies [13]. Additionally, certain signaling pathways in the microenvironment can be targeted by drugs to suppress tumor growth and improve outcomes [14]. Overall, the tumor microenvironment is an essential component of tumor biology and can affect the prognosis, growth, and response to treatments of cancer.

In this study, we downloaded the PRAD RNA-sequencing (RNA-seq) data from the UCSC Xena database. Then, the ESTIMATE method was employed to analyze the immune and stromal scores and tumor purity of PRAD samples. In addition, we analyzed the correlation between TIME and clinical information, from which we obtained the novel TIME-related prognostic genes for PRAD.

## Materials and methods

### Data processing

RNA-seq data of PRAD were obtained from UCSC Xena (https://xenabrowser.net/datapages/) according to Illumina HiSeq 2000 RNA-seq platform. The data included 551 samples of which 546 samples (494 tumor samples and 52 controls) with clinical information were used as the training dataset. Meanwhile, the GSE70768 dataset was obtained from NCBI Gene Expression Omnibus (https://www.ncbi.nlm.nih.gov/) database, including 199 samples (25 tumor samples and 74 controls), which was regarded as the validation dataset [15].

### Evaluation of immune and stromal scores of samples using ESTIMATE

To calculate the immune, stromal, tumor purity, and ESTIMATE scores of samples in the UCSC dataset, the ESTIMATE package in R3.6.1 (http://127.0.0.1:29606/library/estimate/html/estimateScore.html) [16] was used. The samples were divided into high or low- scores groups according to the median value of immune, stromal, tumor purity, and ESTIMATE scores, respectively. Then, by combining with clinical information of the samples (including overall survival [OS], recurrence-free survival (RFS, and disease-free survival [DFS]), the Kaplan–Meier (KM) of survival package (version 2.41-1, http://bioconductor.org/packages/survivalr/) [17] in R3.6.1 was employed to assess the prognostic difference in different levels of TIME scores.

### Screening of TIME-related genes

According to the immune and stromal scores, samples were separated into two groups including high and low stromal scores and high and low immune scores. Then, the limma package in R3.6.1 (version 3.34.7, https://bioconductor.org/packages/release/bioc/html/limma.html) [18] was employed to screen differentially expressed genes (DEGs) in the two groups with the threshold of the false discovery rate (FDR) of < 0.05 and |log2fold change (FC)|>0.5. The screened DEGs were visualized using heatmap constructed by pheatmap (https://cran.r-project.org/web/packages/pheatmap/index.html) in R3.6.1 [19, 20]. Then, the overlapped DEGs between the two groups were retained for the subsequent analysis. Gene Ontology (GO) and Kyoto Encyclopedia of Genes and Genomes analyses were performed on the overlapped DEGs via DAVIA (version 6.8, https://david.ncifcrf.gov/) [21, 22] with the threshold of FDR < 0.05.

### Screening of TIME-related prognostic genes

The univariate Cox regression analysis of the survival package (version 2.41-1) [17] was conducted to obtain the TIME-related prognostic genes on the basis of the abovementioned TIME-related DEGs. Then, LASSO in LARS package (version 1.2, https://cran.r-project.org/web/packages/lars/index.html.) [23] was used to further obtain prognosis-related DEGs. Furthermore, the multivariate Cox regression analysis in R3.6.1 (version 2.41-1) [17] was used to screen independent TIME-related prognostic genes, and the results were visualized using forestplot (version 1.10, https://cran.r-project.org/web/packages/forestplot/index.html.) [24]. Then, the TIME-related genes were differentially expressed in the PRAD tumor and control samples from the UCSC dataset. Based on the DEG expression, the samples were separated into high and low expression groups. The Fisher precision test in R3.6.1 was used to analyze the difference between the two groups. Moreover, the significance of OS, RFS, and DFS in different groups was assessed using the KM curves [17]. The immunohistochemistry staining of TIME-related prognostic DEGs was validated using the Human Protein Atlas (HPA) (https://www.proteinatlas.org/) [25].

### Correlation analysis between novel TIME-related DEGs and TIME

The TIME-related DEG expression level was extracted from the UCSC dataset, and cor function (https://www.rdocumentation.org/packages/stats/versions/3.6.1/topics/cor) was then performed to calculate the association between TIME-related DEGs and TIME scores. The results were represented using a correlation scatter plot.

Tumor IMmune Estimation Resource (https://cistrome.shinyapps.io/timer/) [26] was applied to analyze the association between TIME-related DEGs and six tumor-infiltrating immune cells (i.e., B cells, CD4 + T cells, CD8 + T cells, neutrophils, macrophages, and dendritic cells). Then, the correlation between TIME-related genes and infiltration of various immune cell subtypes was analyzed.

### Validation of TIME-related DEGs

Initially, the expression levels of TIME-related DEGs were obtained from GSE70768 (validation dataset), and the difference in TIME-related DEGs between PRAD tumor samples and controls was then analyzed. Meanwhile, we collected 10 pairs PRAD tissues and adjacent tissues to validate the TIME-related DEGs. The expression of the TIME-related DEGs using quantitative real-time PCR (qRT-PCR) through the instruction of previous study. The results were calculated using 2^−△△Ct^ method. Then, the samples were divided into high and low expression groups according to the clinical prognostic information of PRAD samples. KM curves were made to assess the significance of sample prognosis in the high and low expression groups.

## Results

### Evaluation of immune and stromal scores of samples using ESTIMATE

**Fig. 1:**
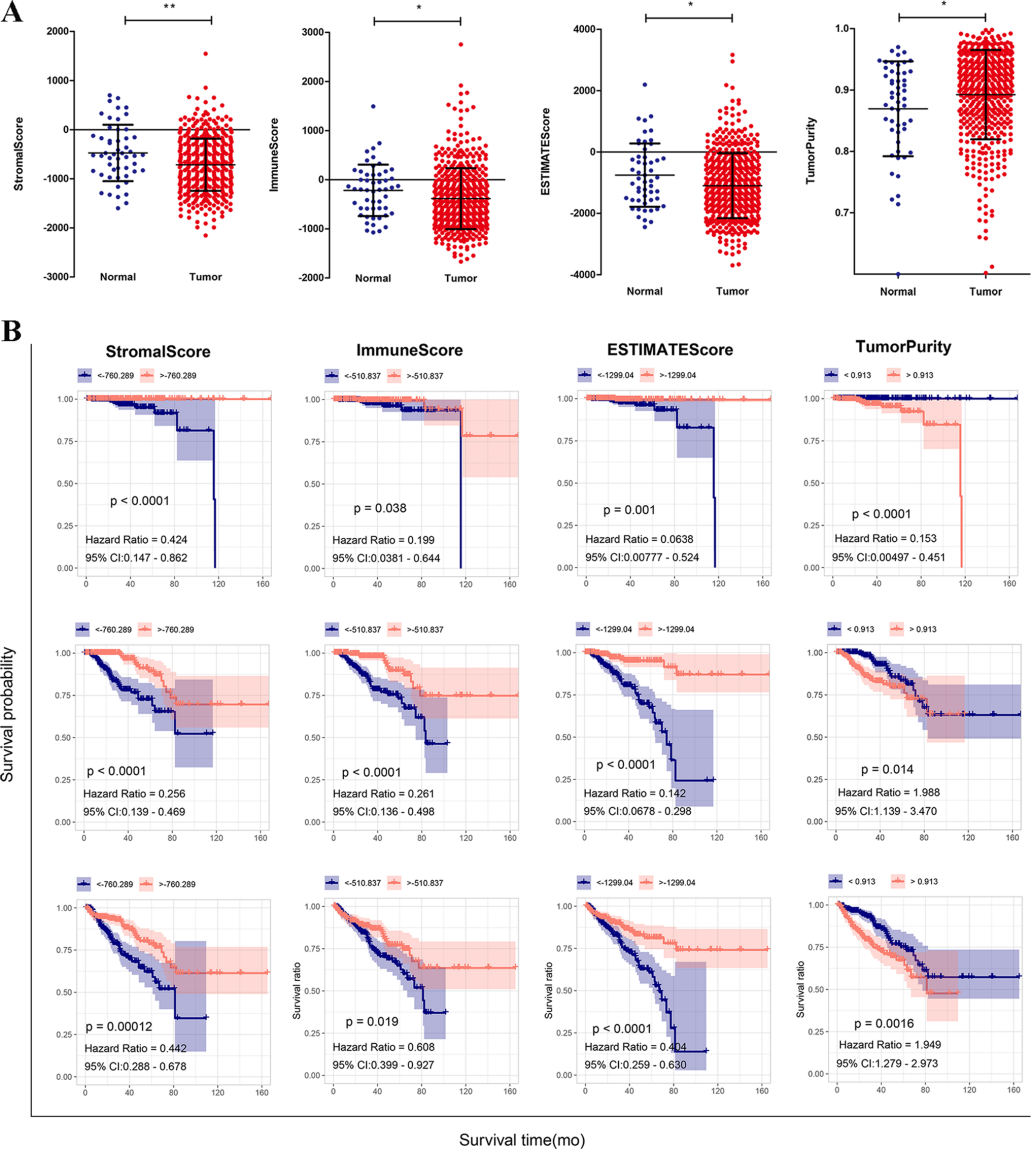
Evaluation of immune and stromal scores of samples using ESTIMATE. **A** Stromal, immune, and ESTIMATE scores and purity in controls and PRAD tumor tissues. **B** KM curves for evaluating the OS, RFS, and DFS in different levels of stromal, immune, and ESTIMATE scores and purity. *DFS* disease-free survival, *OS* overall survival, *PRAD* prostate adenocarcinoma, *RFS* recurrence-free survival

ESTIMATE results indicated that the ESTIMATE, immune, and stromal scores in PRAD tumors were significantly lower than those in controls; contrary results were obtained in tumor purity (Fig. 1A, Supplementary Table 1). Then, the samples were divided into high or low scores of ESTIMATE, immune, and stromal. As shown in Fig. 1B, high ESTIMATE, immune, and stromal scores correlated with good prognosis including OS, RFS, and DFS. By contrast, the higher tumor the purity score, the worse the patient’s prognosis.

### Screening of TIME-associated genes

**Fig. 2:**
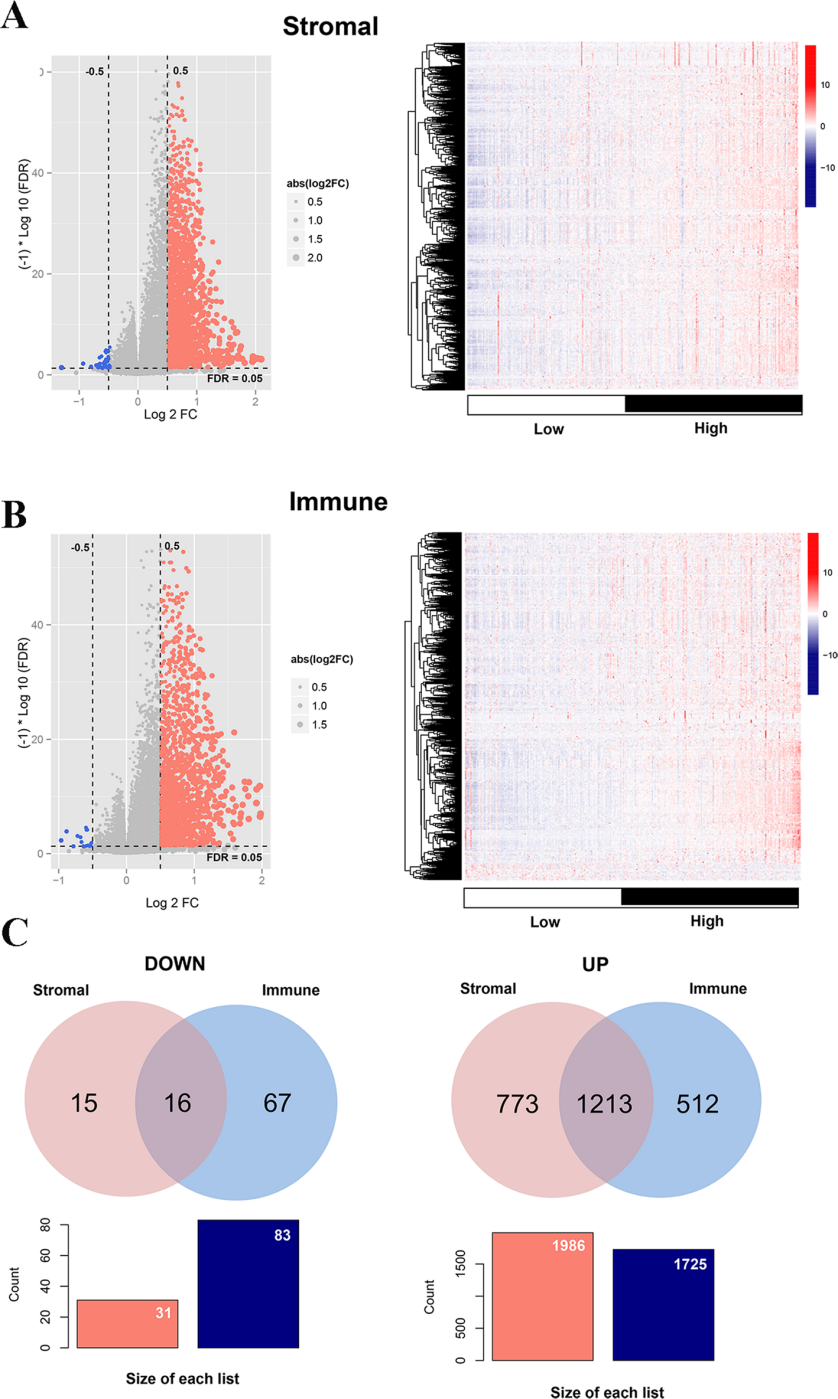
Screening of TIME-related genes. **A** DEGs in the high and low stromal score groups. **B** DEGs in the high and low immune score groups. **C** Overlapped DEGs in the grouping of stromal and immune scores. *DEGs* differentially expressed genes, *TIME* tumor immune microenvironment

The samples were divided into high and low stromal scores and high and low immune scores. A total of 2017 and 1808 DEGs were screened from these two groups, which were visualized using heatmap and volcano map (Fig. 2A, B). Then, the overlapped DEGs between the stromal and immune groups were obtained; as a result, 16 downregulated and 1213 upregulated DEGs were screened (Fig. 2C).

**Fig. 3:**
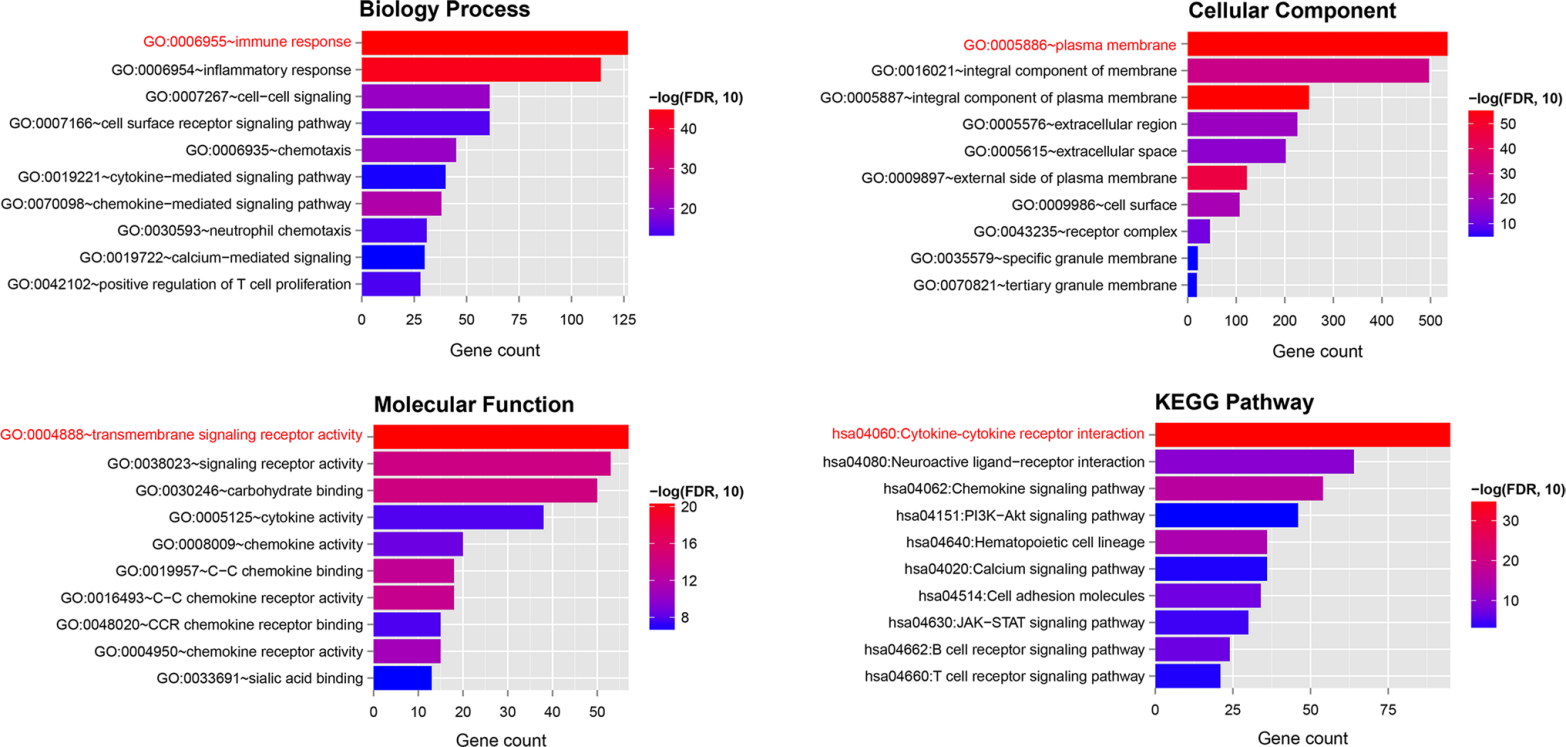
GO function and KEGG pathway analysis for TIME-related DEGs. *DEGs* differentially expressed genes, *GO* Gene Ontology, *KEGG* Kyoto Encyclopedia of Genes and Genomes, *TIME* tumor immune microenvironment

The GO and KEGG analysis results indicated that the overlapped DEGs were involved in 136 biology processes (BPs), 17 cellular components, 31 molecular functions, and 19 KEGG pathways (Supplementary Table 2). The top 10 GO and KEGG are presented in Fig. 3, indicating that the overlapped DEGs significantly participated in immune response, plasma membrane, transmembrane signaling receptor activity, and cytokine–cytokine receptor interaction. Among the GO function and KEGG pathways, cytokine–cytokine receptor interaction was the most significant pathway that is separately displayed in Supplementary Fig. 1. Furthermore, the overlapped DEGs in this pathway were labeled, such as *CCR8*, *CCL21*, and *IL2RA*.

### Screening of TIME-related prognostic genes

**Fig. 4:**
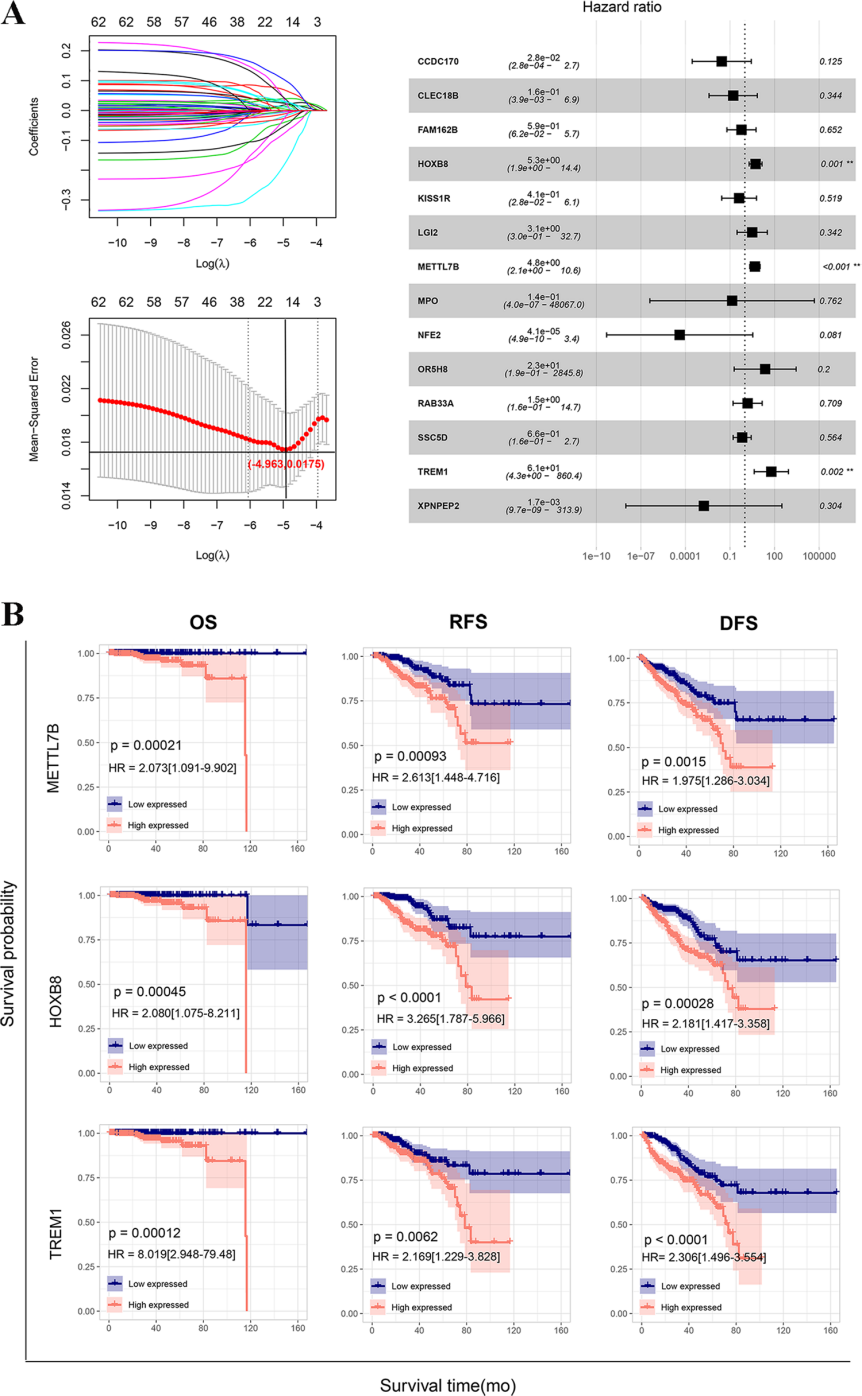
Screening of TIME-related prognostic genes. **A** LASSO parameter and multivariate Cox regression analysis forest display map. **B** KM curves for evaluating the importance of *METTL7B, HOXB8*, and *TREM1* through OS, RFS, and DFS. *DFS* disease-free survival, *KM* Kaplan–Meier, *OS* overall survival, *RFS* recurrence-free survival

In total, 62 TIME-associated prognostic genes were screened from the overlapped TIME-related DEGs. Then, 14 optimized DEGs were obtained from the TIME-related prognostic genes using the LASSO method. Finally, three DEGs (*METTL7B*, *HOXB8*, and *TREM1*) were screened, which were significantly related to independent prognosis (Fig. 4A).

According to the expression levels of *METTL7B*, *HOXB8*, and *TREM1*, the samples in the UCSC dataset were divided into the high and low expression groups. The KM curves indicated that *METTL7B*, *HOXB8*, and *TREM1* correlated with the OS, RFS, and DFS of the samples. Samples with low expression levels of *METTL7B*, *HOXB8*, and *TREM1* had better prognoses (Fig. 4B). Moreover, the correlation between the gene level and clinicopathological information of PRAD is analyzed in Table 1. Notably, low expression levels of *HOXB8* and *TREM1* significantly correlated with a low tumor recurrence rate.

**Table 1:** Statistical comparison of clinical information of samples from groups with different expression levels of mettl7b, Hoxb8 and TREM1

Characteristics total cases	METTL7B expression		P value	HOXB8 expression		P value	TREM1 expression		P value
	Low	High		Low	High		Low	High	
Age (years)									
≤ 60	121	100	0.07022	115	106	0.4692	124	97	0.01855
> 60	126	147		132	141		123	150	
Pathologic M									
M0	226	226	0.2484	225	227	0.2484	223	229	0.9998
M1	0	3		0	3		1	2	
Pathologic N									
N0	175	169	0.6172	178	166	0.103	171	173	0.132
N1	37	41		32	46		31	47	
Pathologic T									
T1-T2	94	92	0.9258	98	88	0.4016	108	78	0.008936
T3-T4	150	151		146	155		137	164	
Gleason score									
6–7	152	138	0.2348	161	129	0.00456	154	136	0.1202
8–10	95	109		86	118		93	111	
psa value									
0–1	202	189	0.04302	197	194	0.7578	203	188	0.002813
Over 1	17	29		22	24		13	33	
Radiation therapy									
Yes	24	35	0.2082	22	37	0.0495	23	36	0.0688
No	194	192		199	187		202	184	
Tumor recurrence									
Yes	25	33	0.2608	22	36	0.04664	17	41	0.001021
No	189	179		189	179		196	172	

### *Correlation analysis of METTL7B*, *HOXB8, and TREM1 and TIME*

**Fig. 5:**
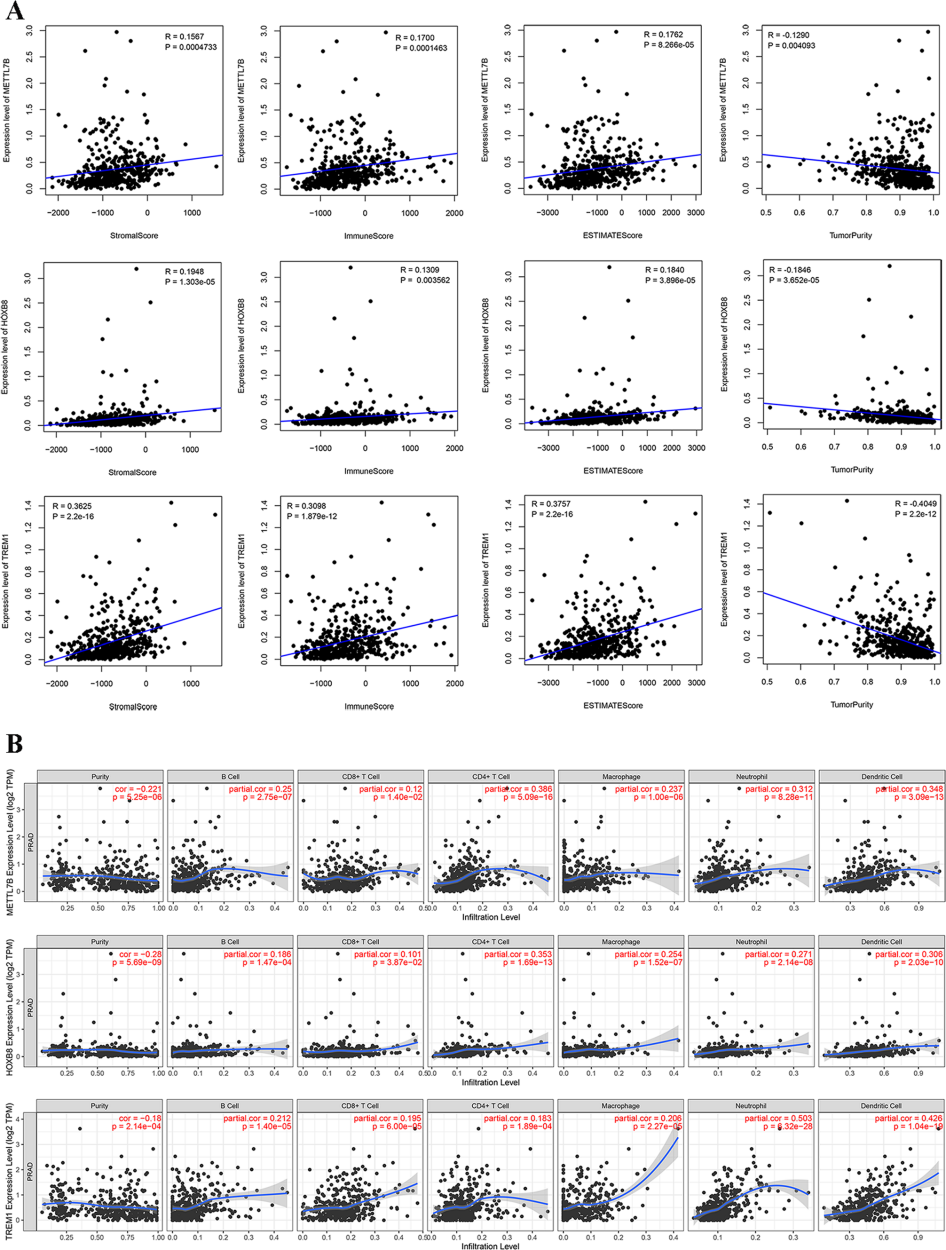
Correlation analyses of *METTL7B, HOXB8*, and *TREM1* and TIME. **A** Scatter plot of the correlation between *METTL7B, HOXB8*, and *TREM1* and stromal, immune, ESTIMATE, and purity. **B** Scatter plot of the correlation between *METTL7B, HOXB8*, and *TREM1* and different proportions of immune cells

To evaluate the association between *METTL7B*, *HOXB8*, and *TREM1* and TIME, the cor function was performed to obtain the relationship between TIME scores and gene expression. A shown in Fig. 5A, the findings revealed that the expression levels of *METTL7B*, *HOXB8*, and *TREM1* showed a significant positive correlation with the ESTIMATE, stromal, and immune scores and a negative correlation with tumor purity. Moreover, we analyzed the association between the expression of the three genes and immune infiltration level in PRAD. As shown in Fig. 5B, the expression of *METTL7B* significantly correlated with CD4 + T cells, neutrophils, and dendritic cells (R > 0.3). *HOXB8* showed a significant correlation with CD4 + T cells and dendritic cells (R > 0.3). *TREM1* was positively related to the infiltration levels of neutrophils and dendritic cells (R = 0.503; R = 0.426). *METTL7B*, *HOXB8*, and *TREM1* were positively correlated with macrophages (R > 0.2).

### Validation of METTL7B, HOXB8, and TREM1 in PRAD

**Fig. 6:**
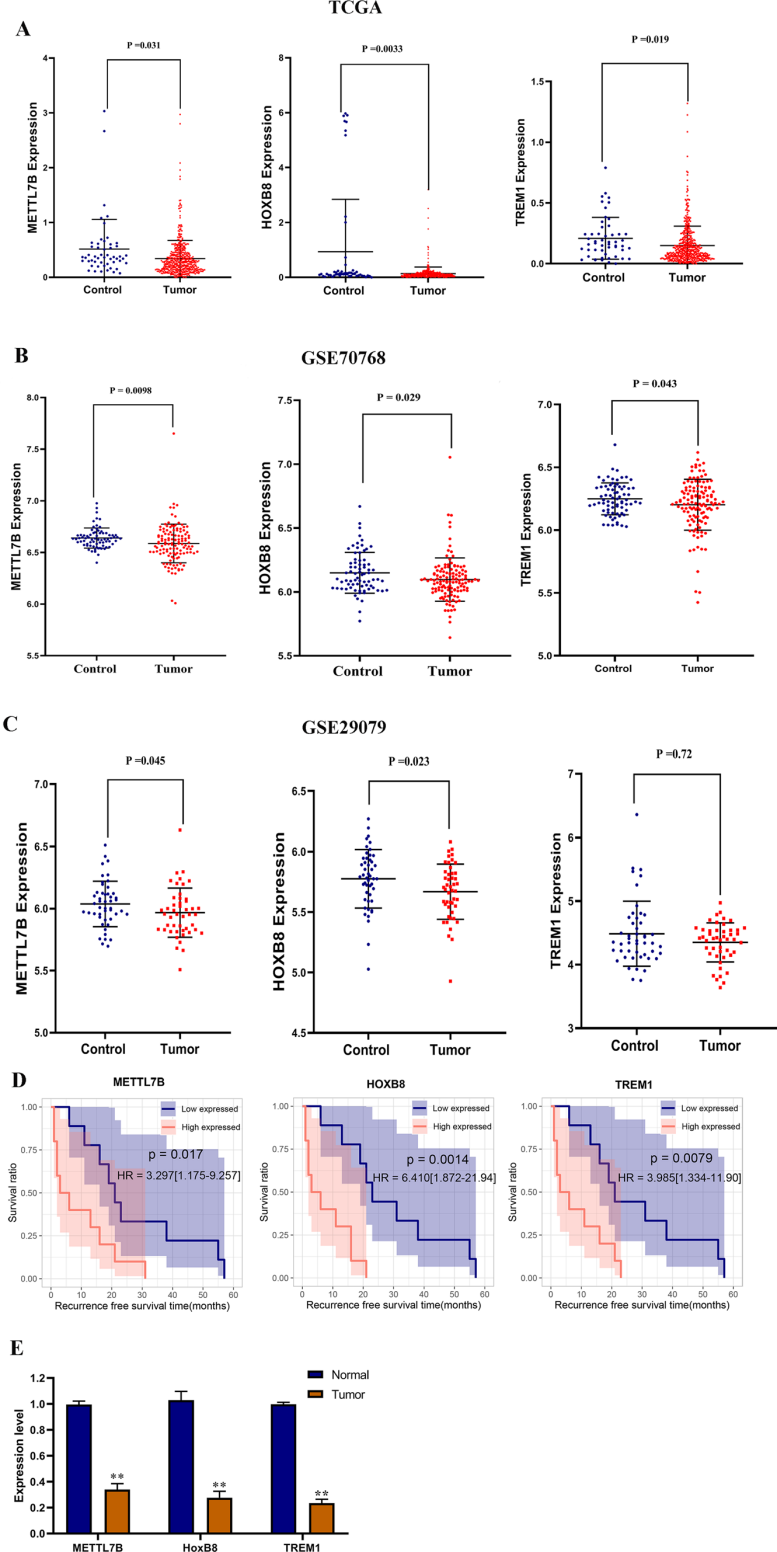
Validation of *METTL7B*, *HOXB8*, and *TREM1* in PRAD. Expression levels of *METTL7B*, *HOXB8*, and *TREM1* in PRAD tumors and normal control samples from TCGA (**A**), GSE70768 (**B**) and GSE29079 (**C**). **D**. KM curves for evaluating the importance of *METTL7B, HOXB8, and TREM1* through OS, RFS, and DFS. **E**. Expression levels of *METTL7B*, *HOXB8*, and *TREM1* in PRAD tumors and normal control samples using qRT-PCR. *DFS* disease-free survival, *KM* Kaplan–Meier, *OS* overall survival, *RFS* recurrence-free survival, *qRT-PCR* quantitative real-time PCR

To validate the importance of *METTL7B*, *HOXB8*, and *TREM1* in PRAD, we obtained their expression levels from the GSE70768 dataset. Figure 6A–C shows that the expression levels of the three genes were consistent with the UCSC dataset. Moreover, KM curves revealed that low expression levels of *METTL7B*, *HOXB8*, and *TREM1* correlated with a good prognosis, which was also consistent with the training dataset (Fig. 6D). Furthermore, we examined these genes between PRAD tissues and adjacent tissues, similar results that the expression of *METTL7B*, *HOXB8*, and *TREM1* in PRAD was significantly decreased (P < 0.01, Fig. 6E).

## Discussion

Multiple factors, stages, and genes are involved in the occurrence and development of PRAD, where TIME is an important factor. Recently, immunotherapy was the novel treatment for PRAD tumors, whereas the clinical outcome was related to the characteristics of malignant tumors, such as hormone dependence, low tumor mutation load, and immunosuppressive microenvironment. Besides, previous studies have found that TIME correlated with the prognosis of patients with PRAD [27]. Thus, further exploration of TIME in PRAD was significant to help doctors make decisions about the treatment method and predict the prognosis for PRAD.

The results of this study revealed that the PRAD samples with high immune and stromal scores had a better prognosis, and those with high tumor purity had a worse prognosis. In addition, we collected the OS, RFS, and DFS of samples to examine the correlation in immune and stromal scores and survival time using KM curves. The results obtained were similar to the ESTIMATE results. These observations were consistent with the results of previous studies. For example, Chen et al. [28] suggested that stromal, immune, and ESTIMATE scores closely correlated with the OS of patients with PRAD. In addition, similar results were obtained in multiple cancer types, such as breast cancer, bladder cancer, and lung adenocarcinoma, indicating that the stromal, immune, and ESTIMATE scores and tumor purity in TIME played a significant role in immunotherapy [29–31]. Xiang et al. [32] indicated that the stromal, immune, and ESTIMATE scores and tumor purity in the microenvironment were associated with TIME. Thus, our study screened TIME-related DEGs by comparing stromal scores and immune scores. Subsequently, 1229 TIME-related DEGs were screened using the limma package. GO BP results indicated the involvement of DEGs in the immune response. Immune response regulated the development of PRAD tumors, which played an important role when making decisions for immunotherapy [33, 34]. The DEGs might be novel biomarkers for treating PRAD. In addition, KEGG results indicated that the DEGs were related to the cytokine–cytokine receptor interaction pathway. Previous studies have found that this pathway always involved some immune-related genes that are involved in different cancers, such as renal cell carcinoma and hepatocellular carcinoma [35, 36]. This pathway might be an important factor in the immunotherapy for PRAD. Further experiments must be performed to understand the mechanism of immunotherapy in PRAD.

Previous studies have indicated that the TIME was related to the prognosis of patients with PRAD. In this study, three prognostic DEGs, namely, *METTL7B*, *HOXB8*, and *TREM1*, were identified. The KM curves were used to evaluate the correlation between the three DEGs and patient prognosis, suggesting that patients with low expression levels of *METTL7B*, *HOXB8*, and *TREM1* had good OS, RFS, and DFS. The results were validated using the external cohort, which obtained the same results as TCGA dataset. Our results were similar with the previous studies that have used external cohorts to validated the prognostic value of the biomarkers in PRAD [37–39]. The method further confirmed the results in this study. This novel TIME hub genes-related risk score model provides a new theoretical basis for the prognosis assessment of PRAD patients, which is expected to be further applied in the future clinical management. A prospective study of clinical cohorts recruiting PRAD patients in different stage will help validating this risk score model. The expression of *METTL7B*, *HOXB8*, and *TREM1* was examined each month. Then, the follow-up was performed to observe the prognosis of PRAD patients. The KM curves and survival analysis will be carried out to the correlation between risk score model and prognosis. This study is expected to be conducted for 5 years or even longer to obtain good persuasiveness.

Meanwhile, significant differences in *METTL7B*, *HOXB8*, and *TREM1* were found between the controls and tumor samples in both mRNA levels. The three genes might be novel TIME-related biomarkers for PRAD. *METTL7B*, an alkyl thiol methyltransferase, could metabolize hydrogen sulfide (H_2_S) [40]. H_2_S was found to participate in the epithelial–mesenchymal transition and tumor migration and invasion [41]. A recent study found that the expression of *METTL7B* positively correlated with immunosuppressive cells suggesting that it might play a significant role in modulating TIME [42]. Meanwhile, *METTL7B* expression was positively associated with CD4 + T cells and dendritic cells. All the results indicated that *METTL7B* could be used to predict the TIME in PRAD. Moreover, Redecke et al. [43] reported that *HOXB8* transfected in mouse bone marrow cells with unlimited proliferative capacity that could enable investigations of immune cell differentiation and function. This study found that *HOXB8* is closely correlated with CD4 + T cells. Besides, Zhao et al. [44] pointed out that high expression levels of *TREM1* had improved the infiltration of regulatory T cells and reduced the infiltration of CD8 + T cells. Similarly, this study found that the expression of *TREM1* could regulate the TIME, including neutrophils and dendritic cells. Previous studies have suggested that CD4 + T cells, CD8 + T cells, neutrophils, and dendritic cells are associated with the impairment of proliferation, cytokine production, and migratory capacities of effector T cells [45]. Besides, Meng et al. [46] firstly pointed out the infiltration of immunocytes among PRAD via the CIBERSORT algorithm. This study indicated that M2 macrophages was related to gene markers, whick could predict the prognosis of PRAD patients. These results were consistent with our study that we found that *METTL7B*, *HOXB8*, and *TREM1* were positively correlated with M2 macrophages. Regulating the expression levels of *METTL7B*, *HOXB8*, and *TREM1* may have remarkable clinical applications in enhancing immunotherapy. Immunotherapy has shown good prospects in treating cancer. We will continue to focus on the genes related to tumor microenvironment of PRAD in the future. Further exploration on genes related to tumor microenvironment will help treating patients with PRAD using immunotherapy as soon. Thus, more experiments such as mice experiments, molecular biology research and clinical test need be performed to validate these results in this study.

## Conclusions

This study explored the expression levels of three TIME-related genes including *METTL7B*, *HOXB8*, and *TREM1*, which correlated with the prognosis of patients with PRAD. Moreover, targeting the TIME-related genes might have important clinical implications when making decisions for immunotherapy in PRAD.

### Supplementary Information

Supplementary material 1

Supplementary material 2

Supplementary material 3

## Data Availability

The datasets generated and/or analysed during the current study are available in the UCSC Xena repository, (https://xenabrowser.net/datapages/?dataset=TCGA-PRAD.htseq_fpkm.tsv&host=https%3A%2F%2Fgdc.xenahubs.net&removeHub=https%3A%2F%2Fxena.treehouse.gi.ucsc.edu%3A443). GSE70768: https://www.ncbi.nlm.nih.gov/geo/query/acc.cgi?acc=GSE70768.
