# Supplementary figures and images for: Identifying the tumor immune microenvironment-associated prognostic genes for prostate cancer
Source: Discov Oncol. 2024 Feb 20;15:42. doi: 10.1007/s12672-023-00856-3 (PMC10879074; doi:10.1007/s12672-023-00856-3)

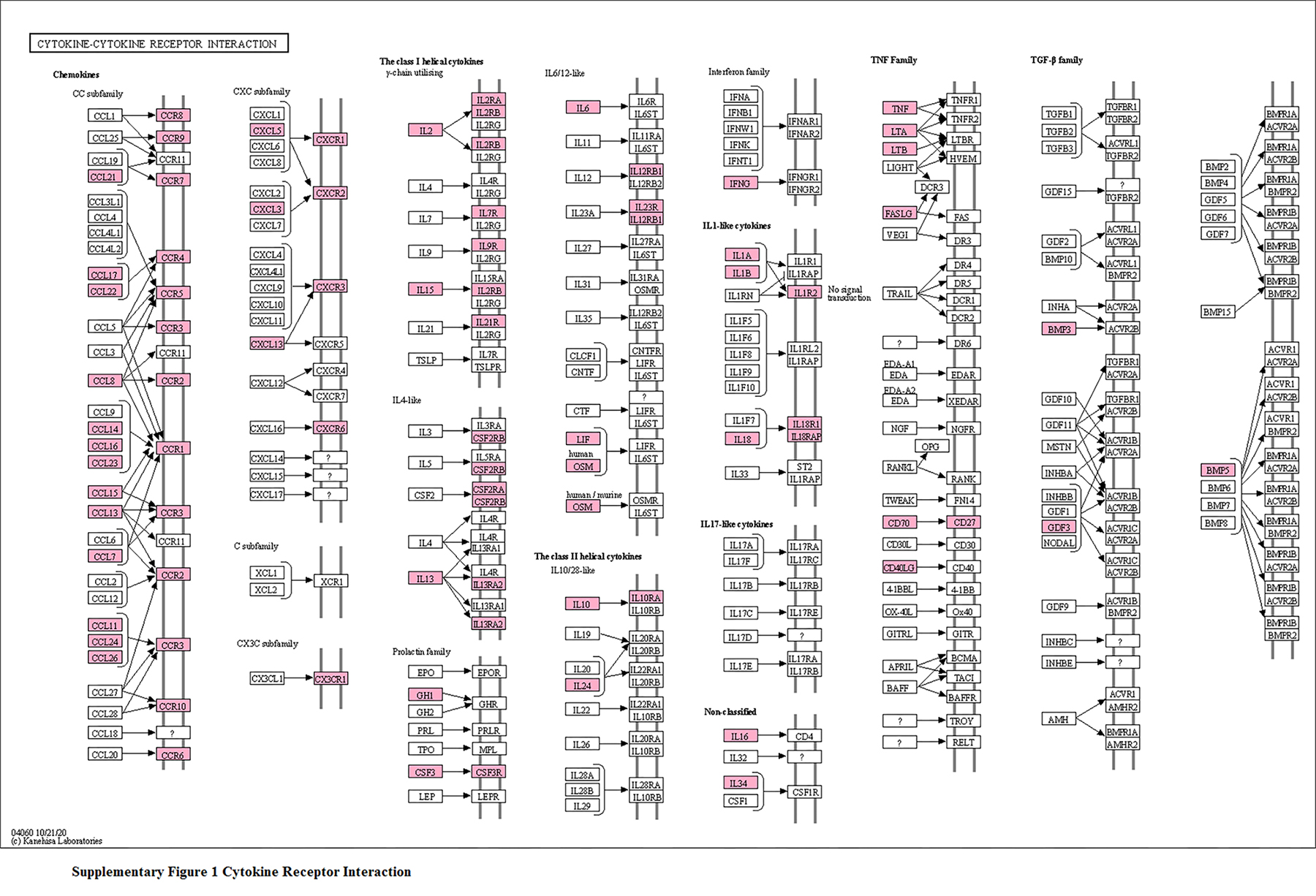

Supplement: Supplementary file 1 — Supplementary material 1 [file 12672_2023_856_MOESM1_ESM.jpg]
