# Supplementary material for: Identifying the tumor immune microenvironment-associated prognostic genes for prostate cancer
Source: Discov Oncol. 2024 Feb 20;15:42. doi: 10.1007/s12672-023-00856-3 (PMC10879074; doi:10.1007/s12672-023-00856-3)
Supplement: Supplementary file 2 — Supplementary material 2 [file 12672_2023_856_MOESM2_ESM.docx]

Supplementary Table 2 The GO and KEGG results indicated that the overlapped DEGs were involved in 136 biology processes (BPs), 17 cellular component, 31 molecular function, and 19 KEGG pathways

| Category | Term | Count | PValue | FDR | Genes |
| --- | --- | --- | --- | --- | --- |
| GOTERM_BP_DIRECT | GO:0006955~immune response | 127 | 7.12E-50 | 2.67E-46 | CD86, CSF3, ADAMDEC1, GPR65, CD80, LST1, LY75, CXCL13, TNF, IFI44L, VPREB3, CYSLTR2, IL18RAP, CCRL2, TNFSF11, HLA-DOB, TNFRSF4, CMKLR1, GBP6, CD96, IL1R2, LAX1, CHIT1, CLEC4C, CLEC4D, CLEC4E, SEMA7A, IGSF6, AIRE, CD1E, CD1D, CD1C, CD1B, CD1A, CD79B, HRH2, ICOS, CCR10, CCR1, XCR1, TNFSF14, LIF, PRG2, IL2, RAET1E, GPR183, TNFSF8, IL7R, HAMP, CD22, CIITA, FASLG, FCAR, CXCL5, TNFSF13B, SPN, FCGR3B, CCR9, CCR8, CTLA4, CCR7, CTSG, CCR6, CCR5, CCR4, CTSC, CCR3, CCR2, IL10, CR2, IL15, IL13, IL18, IL16, SERPINB9, PDCD1LG2, VAV1, TLR1, IL1A, NCR3, TMIGD2, AIM2, ITGAD, IL1B, LTA, IRF8, TLR8, TLR7, LCP2, TLR10, TLR6, PKHD1L1, LTB, MS4A2, TLR2, CCL14, CX3CR1, CCL13, CCL11, PTAFR, CXCR6, CST7, IL1RL1, CCL8, CNR2, CXCR1, CXCR3, CXCR2, FCGR1A, S1PR4, CCL16, MICB, CCL15, CCL24, CCL23, CCL22, CCL21, CD70, GZMA, OSM, LILRB2, CD40LG, IL2RA, ACKR4, FCGR2B, FCGR2C, IL18R1 |
| GOTERM_BP_DIRECT | GO:0006954~inflammatory response | 114 | 9.65E-48 | 1.81E-44 | GPR68, IL23R, LY75, CXCL13, TNF, CRHBP, IL18RAP, ADORA3, CCRL2, ADORA1, C3AR1, PROK2, TNFRSF4, PTGDR, CMKLR1, GBP5, CD96, CD180, CYBB, PRKCQ, S100A8, SEMA7A, C5AR2, FPR1, LY96, PLD4, FPR3, FPR2, MEFV, HRH1, SCUBE1, FUT7, CLEC7A, NLRP3, S100A12, NLRP1, CCR1, XCR1, LY86, PPBP, SELE, IL6, VNN1, NOX4, CIITA, TNFAIP6, PIK3CD, ITGAL, CXCL3, PIK3CG, CXCL5, PSTPIP1, BDKRB2, OLR1, CCR7, CCR5, CCR4, PF4V1, CCR3, CCR2, HAVCR2, PTGIR, IL15, IL13, IL18, TACR1, TLR1, IL1A, HCK, NCR3, AIM2, IL1B, KIT, XCL2, TLR8, TLR7, TLR10, TLR6, MS4A2, TLR2, CCL14, CX3CR1, CCL13, CCL11, PTAFR, CXCR6, PTGS1, CCL8, CCL7, CNR2, CXCR3, CXCR2, CCL17, CCL16, CCL15, KLRG1, CCL24, PLA2G2D, CCL23, CCL22, CCL21, IL34, P2RX7, CD40LG, THEMIS2, CAMK4, TNIP3, IL2RA, SIGLEC1, FOLR2, FCGR2B, IL18R1, PF4, CCL26 |
| GOTERM_BP_DIRECT | GO:0070098~chemokine-mediated signaling pathway | 38 | 9.70E-28 | 1.21E-24 | CCL14, CX3CR1, CCL13, CCL11, CXCR6, CXCL13, CXCL3, CXCL5, CCL8, CCL7, CXCR1, CXCR3, CCRL2, CXCR2, CCR9, CCR8, CCR6, CCR5, CCR4, CCL17, CCR10, PF4V1, CCR3, CCL16, CCR2, CCL15, CMKLR1, CCR1, CCL24, CCL23, XCR1, CCL22, CCL21, PPBP, ACKR4, XCL2, PF4, CCL26 |
| GOTERM_BP_DIRECT | GO:0007267~cell-cell signaling | 61 | 4.79E-24 | 4.36E-21 | TNFAIP6, SIRPG, FASLG, TRH, CXCL13, AREG, CXCL5, FGF9, GRAP2, ADORA1, INSL3, CCR5, CD33, GAL3ST4, TSHB, FCRL2, PTGIR, FGFBP2, IL15, IL18, WNT9B, SSTR3, PGF, EREG, ADCYAP1, IL1B, LTA, PGR, LTB, CX3CR1, CCL13, ENPEP, TRHDE, GATA1, TSPAN32, PTHLH, GJC2, CCL8, CCL7, CCL17, CCL16, CCL15, TNFSF18, CCR1, CCL24, CCL23, CCL22, CCL21, CD70, LILRB2, TBX5, ESR2, IL2, POMC, BMP3, FGF14, FGF18, TNFSF8, GRAP, SIGLEC6, CCL26 |
| GOTERM_BP_DIRECT | GO:0006935~chemotaxis | 45 | 5.81E-24 | 4.36E-21 | CX3CR1, CCL13, CCL11, C5AR2, PTAFR, FPR1, PIK3C2G, FPR3, CXCR6, FPR2, CXCL5, SPN, CCL8, CCL7, CXCR3, CCRL2, CXCR2, C3AR1, CCR9, PROK2, CCR8, CCR7, NCKAP1L, CCR6, CCR5, CCR4, CCL17, CCR10, CCR3, CCL16, CCR2, CCL15, CMKLR1, CCR1, CCL24, CCL23, XCR1, CCL22, CMTM5, IL16, RNASE2, FOSL1, ACKR4, DOCK2, CCL26 |
| GOTERM_BP_DIRECT | GO:0007166~cell surface receptor signaling pathway | 61 | 9.75E-18 | 6.09E-15 | CD86, SIGLEC9, TNFRSF13B, CD80, MS4A7, CD3G, PTH1R, CD3E, CXCL13, SIRPB1, ASGR2, SPN, FCRLA, MILR1, FCGR3B, BDKRB2, FCER1A, MS4A14, CCR5, FCRL1, FCRL2, FCRL5, FCRL6, FCRL3, GFRA1, PDCD1LG2, MS4A6A, DOK2, IFNG, MS4A2, MET, MS4A1, ADCYAP1R1, IGSF6, KLRB1, LY96, LILRA1, FPR2, CALCR, CXCR1, CXCR3, CXCR2, FCGR1A, GPHA2, KLRG1, CCR1, LILRB2, LILRB5, P2RX7, MARCO, PTPRC, FCGR2A, IL2RA, GPR182, CD28, CD27, KLRD1, CD247, FCGR2B, IL7R, FCGR2C |
| GOTERM_BP_DIRECT | GO:0042102~positive regulation of T cell proliferation | 28 | 2.20E-17 | 1.18E-14 | SASH3, CD86, CD80, IL23R, CLECL1, TNFRSF13C, CD1D, VTCN1, CD3E, TNFSF13B, IL12B, NCKAP1L, JAK3, HAVCR2, VCAM1, CD70, IL15, LILRB2, PDCD1LG2, IL6, PTPRC, CD40LG, CD6, CD209, IL1B, CD28, PRKCQ, CARD11 |
| GOTERM_BP_DIRECT | GO:0030593~neutrophil chemotaxis | 31 | 2.88E-17 | 1.35E-14 | CCL14, CCL13, CCL11, CSF3R, PIK3CD, CXCL13, CXCL3, TREM1, CXCL5, PIK3CG, CCL8, CCL7, CXCR1, CXCR2, S100A12, NCKAP1L, CCL17, PF4V1, CCL16, CCL15, CCL24, CCL23, CCL22, CCL21, PPBP, VAV1, IL1B, XCL2, S100A8, PF4, CCL26 |
| GOTERM_BP_DIRECT | GO:0019221~cytokine-mediated signaling pathway | 40 | 1.11E-15 | 4.16E-13 | LILRA6, CSF3, CSF3R, FLT3, IL23R, EBI3, CSF2RB, LILRA1, LILRA2, CSF2RA, LILRA4, LILRA5, STAT4, IL21R, IL12B, TNFSF11, CTSG, IL12RB1, IL13RA2, JAK3, CCR2, CCR1, IL10RA, IL1R2, IRAK3, IL16, LILRB1, LILRB2, LILRB5, EREG, IL1A, HCK, IL6, IL1B, KIT, IL2RB, IL7R, IL9R, PF4, CRLF2 |
| GOTERM_BP_DIRECT | GO:0019722~calcium-mediated signaling | 30 | 1.97E-15 | 6.70E-13 | CX3CR1, LRRK2, HTR2B, CXCR6, FPR2, TNF, SAMD14, CXCR1, CXCR3, CCRL2, CXCR2, PLCG2, CCR9, CCR8, TNFSF11, CCR7, CCR6, CCR5, CCR4, CCR10, CCR3, CCR2, CCR1, P2RY12, XCR1, SELE, POMC, TREML1, ACKR4, BTK |
| GOTERM_BP_DIRECT | GO:0007165~signal transduction | 138 | 6.46E-15 | 2.02E-12 | CSF3R, HHIP, GLDN, CRHBP, FGF7, FGF9, SIT1, ADORA3, CLEC5A, ADORA1, TNFRSF8, VSTM1, PRKCB, FLT3LG, ANK2, PGF, MOB3B, DOK2, PGR, CD226, GRAPL, CARD11, CHRNA1, GRIA2, ENPEP, PDE1C, PDE1B, RRAD, SH2D2A, CHRNA6, FPR1, DAPP1, FPR3, CSF2RB, ARHGAP15, RASGRP2, TRHDE, RASGRP1, CD79B, ARHGAP20, SCUBE1, INPP5D, TNFRSF17, NLRP3, FAM83B, TNFSF18, TNFSF14, TSLP, ESR1, ARHGAP25, ESR2, BST1, NR4A3, FGF14, FGF18, PNOC, TNFSF8, CHRFAM7A, IL7R, ARHGAP9, ITK, TNFAIP6, LRRK2, IL24, RASGRF1, PIK3CD, FASLG, TRH, ITGAL, RND2, LRRC2, SIRPB1, CXCL5, TNFSF13B, SPN, PSTPIP1, TAGAP, CASP1, CCR6, CLIC2, CCR5, CD33, IL10, RIPK3, KSR1, IL15, IL13, IL16, CEACAM3, TLR1, NCR1, APBB1IP, CD200R1, IL1B, CEACAM4, KIT, XCL2, LTA, TLR6, ITGB1BP2, LTB, MET, IL9R, TLR2, GUCY2C, DTHD1, CCL13, CCL11, RETN, LILRA2, REM1, IL1RL1, CCL8, CCL7, CXCR2, IL12RB1, FCGR1A, CCL15, CCL24, RCVRN, CCL23, CCL22, TRAT1, CD70, MACC1, CMTM5, LILRB1, LILRB2, POMC, GPR141, P2RX6, CAMK4, IL2RB, SIGLEC8, FCGR2B, CD244, IL18R1, CCL26 |
| GOTERM_BP_DIRECT | GO:0060326~cell chemotaxis | 26 | 5.44E-14 | 1.57E-11 | CX3CR1, CXCR6, CXCR1, CXCR3, CCRL2, CXCR2, CCR9, CCR8, CCR7, CCR6, CCR5, CCR4, EPHB1, CCR10, CCR3, CCR2, CCR1, PDGFRA, XCR1, VCAM1, CCL21, HGF, BIN2, KIT, ACKR4, PRKCQ |
| GOTERM_BP_DIRECT | GO:0006968~cellular defense response | 22 | 3.81E-13 | 1.02E-10 | CX3CR1, ITK, NCF1, TRAT1, NCF2, PRF1, LY96, LILRB2, SPN, NCR1, FOSL1, GNLY, CLEC5A, CXCR2, CCR9, MNDA, CCR6, CD300C, CCR5, CCR3, CCR2, KLRG1 |
| GOTERM_BP_DIRECT | GO:0071222~cellular response to lipopolysaccharide | 41 | 4.53E-13 | 1.13E-10 | CD86, CX3CR1, CSF3, CEBPE, CD80, IL24, LY96, CXCL13, CXCL3, TNF, GATA1, NUGGC, FCAR, CXCL5, MRC1, CASP1, ANKRD1, IL12B, NLRP3, CTSG, CCR5, PF4V1, HAVCR2, IL10, CD180, LILRB1, PPBP, LILRB2, PDCD1LG2, KMO, IL1A, IL6, IL1B, TNIP3, CARD17, IRF8, BPI, CARD16, CD68, HAMP, PF4 |
| GOTERM_BP_DIRECT | GO:0070374~positive regulation of ERK1 and ERK2 cascade | 45 | 6.71E-13 | 1.57E-10 | CCL14, CCL13, SEMA7A, CCL11, C5AR2, HTR2B, PTPN22, HTR2A, FPR2, RASGRP1, TNF, P2RY6, CCL8, CALCR, CCL7, CCR7, TNFAIP8L3, CCL17, CCL16, HAVCR2, CCL15, SLAMF1, CCR1, NTRK1, CCL24, PDGFRA, CASR, CCL23, CCL22, NPY5R, CCL21, GPR55, GPBAR1, SCIMP, HCRTR1, IL1A, MARCO, ADCYAP1, PTPRC, GPR183, FGF18, XCL2, NOX4, FGF10, CCL26 |
| GOTERM_BP_DIRECT | GO:0032729~positive regulation of interferon-gamma production | 26 | 8.77E-13 | 1.94E-10 | SASH3, IL23R, EBI3, PTPN22, CD3E, RASGRP1, TNF, CLEC7A, IL12B, SLAMF6, IL12RB1, CCR2, HAVCR2, SLAMF1, CRTAM, IL18, LILRB1, IL2, IL1B, LTA, IRF8, TLR8, TLR7, CD226, CD244, IL18R1 |
| GOTERM_BP_DIRECT | GO:0007155~cell adhesion | 76 | 2.57E-12 | 5.36E-10 | ROBO2, CSF3R, SIGLEC9, ITGAM, TNFAIP6, SIRPG, ICAM3, HAPLN3, ITGAL, HAPLN1, HAPLN2, PSTPIP1, CDH2, CCR8, ITGB7, SVEP1, PLXNC1, CD33, CCR3, CD96, EGFL6, MYBPC2, ITGA4, CD300A, EPHA8, IZUMO1R, CLDN11, HCK, MMRN1, ITGAD, NINJ2, ADAM12, CLDN18, CD226, DSCAML1, CDH17, CX3CR1, SELPLG, CCL11, FPR2, PCDH19, LY9, COL19A1, CXCR3, HAS1, ADAM23, CLCA2, SLAMF7, NCAM1, SLAMF1, CCR1, SIGLEC14, CD72, VCAM1, CNTN5, MUC16, PCDH9, SIGLEC11, SIGLEC10, SELE, LYVE1, GP5, IL2, THEMIS2, SELL, FAP, PARVG, PRPH2, CNTN2, FOLR3, SIGLEC8, FOLR2, SIGLEC7, CD22, SIGLEC6, SIGLEC5 |
| GOTERM_BP_DIRECT | GO:0045087~innate immune response | 77 | 9.44E-12 | 1.86E-09 | PGLYRP4, CD84, ITGAM, NCF1, NCF2, CLEC10A, PIK3CD, SLA, C4BPA, PRDM1, TREM1, PIK3CG, OASL, PSTPIP1, CLEC5A, TNFAIP8L2, JAK3, HAVCR2, ZBP1, TRIM61, CD180, CYBB, MPEG1, TLR1, FGR, HCK, CLEC4C, AIM2, BTK, TLR8, TLR10, BPI, TLR7, PADI4, WFDC10B, TLR6, CLEC4E, S100A8, TMEM106A, TLR2, BLK, CX3CR1, CFI, DEFB124, LY96, PLD4, CD1D, LILRA2, MEFV, LY9, LILRA4, LILRA5, NLRP6, S100A12, NLRP3, SLAMF6, FCGR1A, SLC15A3, SLAMF1, KLRG1, APOBEC3H, IL34, MX2, SIGLEC10, SH2D1B, LY86, SSC5D, TREML1, IGLL5, MARCO, GZMM, VNN1, CD6, CD209, PYDC1, CD244, APOBEC3A |
| GOTERM_BP_DIRECT | GO:0002548~monocyte chemotaxis | 19 | 1.52E-11 | 2.86E-09 | CCL14, CCL24, CCL13, CCL23, CCL11, CCL22, CCL21, IL6, CCL8, CCL7, XCL2, S100A12, TNFSF11, CTSG, CCL17, CCL16, CCR2, CCL26, CCL15 |
| GOTERM_BP_DIRECT | GO:0071346~cellular response to interferon-gamma | 27 | 3.55E-11 | 6.35E-09 | CCL14, CCL13, CIITA, CCL11, FASLG, TNF, FCAR, CCL8, CCL7, MRC1, CASP1, IL12B, IL12RB1, CCL17, CCL16, CCL15, GBP6, CCL24, GBP5, CCL23, CCL22, CCL21, XCL2, IRF8, TDGF1, CCL26, TLR2 |
| GOTERM_BP_DIRECT | GO:0032743~positive regulation of interleukin-2 production | 16 | 3.84E-10 | 6.47E-08 | SASH3, CD86, CD80, DEFB124, VTCN1, CD3E, IL1A, PTPRC, CLEC7A, IRF4, IL1B, CD28, PLCG2, PRKCQ, CARD11, CCR2 |
| GOTERM_BP_DIRECT | GO:0002250~adaptive immune response | 60 | 3.97E-10 | 6.47E-08 | CD86, ITK, CD84, TNFRSF13B, CLEC10A, PIK3CD, CD3G, TNFRSF13C, PRDM1, CD3E, TREM1, PIK3CG, SIT1, CTLA4, JAK3, HLA-DOB, HAVCR2, PRKCB, THEMIS, PDCD1LG2, LAX1, CLEC4C, CLEC4D, IFNG, BTK, SKAP1, LILRA6, CX3CR1, CD1E, LILRA1, VTCN1, CD1C, CD1B, CD1A, CD79B, CD79A, BTLA, SLAMF7, TNFRSF17, LAIR1, MICB, SLAMF1, TNFSF18, TRAT1, CRTAM, SIGLEC10, SH2D1B, LILRB1, LILRB2, LILRB4, IL2, LILRB5, CD6, CD209, CAMK4, GPR183, KLRD1, PDCD1, CD247, CD244 |
| GOTERM_BP_DIRECT | GO:0007186~G-protein coupled receptor signaling pathway | 101 | 1.47E-09 | 2.30E-07 | FCN1, GPR68, GPR65, OR5K2, MRGPRE, HTR4, OR52N4, ADORA3, CCRL2, GPR171, ADORA1, C3AR1, PROK2, RGS9, PTGDR, CMKLR1, CASR, GPR78, TAAR1, ADCYAP1, FPR1, FPR2, GPR82, PIK3R6, PIK3R5, HRH1, NLRP6, OR5H8, CCR10, XCR1, GPR15, HTR1E, NMBR, LPAR5, GPR183, GPR182, GPR18, GPR25, HTR2B, PTH1R, CD3E, AREG, PIK3CG, GPR132, GNGT2, CCR9, BDKRB2, CCR8, CCR7, CCR6, CCR5, CCR4, TSHB, CCR2, PTGIR, NPY5R, GPBAR1, GPR31, VAV1, XCL2, RGS18, CCL14, CX3CR1, CCL13, CCL11, RGS13, RGS16, PTAFR, CXCR6, P2RY6, GNG2, CCL8, CCL7, CXCR1, CXCR3, P2RY2, KISS1R, GNG8, CCL17, S1PR4, CCL16, CCL15, P2RY12, MAS1L, P2RY13, CCL24, CCL23, CCL22, OR10G2, CCL21, P2RY14, GPR55, AKR1C2, HCRTR1, GPR141, MAS1, GPR142, ACKR4, LGR6, KCNK2, CCL26 |
| GOTERM_BP_DIRECT | GO:0032755~positive regulation of interleukin-6 production | 25 | 1.84E-09 | 2.77E-07 | PTAFR, DEFB124, LILRA2, TNF, LILRA5, CLEC7A, PLCG2, TSLP, IL16, LILRB2, SCIMP, EREG, P2RX7, TLR1, IL1A, ADCYAP1, IL6, IFNG, POU2AF1, IL1B, TLR8, TLR7, TLR6, TMEM106A, TLR2 |
| GOTERM_BP_DIRECT | GO:0043406~positive regulation of MAP kinase activity | 22 | 2.74E-09 | 3.95E-07 | FLT3, IL34, NTRK3, LRRK2, HTR2B, HTR2A, RASGRP1, TNF, PIK3R6, PIK3CG, PIK3R5, P2RX7, GH1, PTPRC, IL1B, KIT, FGF18, S100A12, NOX4, TNFSF11, TDGF1, ELANE |
| GOTERM_BP_DIRECT | GO:0042110~T cell activation | 17 | 9.43E-09 | 1.31E-06 | ITK, CD84, TNFSF14, PIK3CD, CD3G, CD3E, RASGRP1, LY9, PIK3CG, PTPRC, CLEC7A, IRF4, TREML2, CD28, SLAMF7, CD48, SLAMF6 |
| GOTERM_BP_DIRECT | GO:0032731~positive regulation of interleukin-1 beta production | 19 | 1.13E-08 | 1.46E-06 | GBP5, PYHIN1, MEFV, LILRA2, TNF, LILRA5, P2RX7, IL6, AIM2, IFNG, CLEC7A, CASP1, TLR8, PYDC1, NLRP3, MNDA, NLRP1, TLR6, TMEM106A |
| GOTERM_BP_DIRECT | GO:0007200~phospholipase C-activating G-protein coupled receptor signaling pathway | 19 | 1.13E-08 | 1.46E-06 | P2RY12, CX3CR1, CASR, C5AR2, FPR1, HTR2B, FPR3, PTH1R, TACR1, FPR2, HTR2A, NMBR, ESR1, HRH1, P2RY6, P2RY2, CXCR2, C3AR1, CMKLR1 |
| GOTERM_BP_DIRECT | GO:0050729~positive regulation of inflammatory response | 24 | 1.43E-08 | 1.79E-06 | TNFSF18, ZBP1, CCL24, TSLP, IL15, OSM, IL18, IL16, TNF, IL2, LILRA5, IL1RL1, FABP4, IFNG, IL1B, CASP1, IL12B, S100A12, TLR10, TLR7, NLRP1, S100A8, CCR2, TLR2 |
| GOTERM_BP_DIRECT | GO:0048247~lymphocyte chemotaxis | 14 | 2.01E-08 | 2.44E-06 | CCL14, CCL24, CCL13, CCL23, CCL11, CCL22, CCL21, CCL8, CCL7, XCL2, CCL17, CCL16, CCL26, CCL15 |
| GOTERM_BP_DIRECT | GO:0032695~negative regulation of interleukin-12 production | 11 | 2.11E-08 | 2.47E-06 | IL10, TLR8, CCR7, LILRB1, IRAK3, MEFV, TIGIT, JAK3, SLAMF1, CMKLR1, LILRA5 |
| GOTERM_BP_DIRECT | GO:0032760~positive regulation of tumor necrosis factor production | 24 | 2.55E-08 | 2.90E-06 | SASH3, CD84, LRRK2, PTAFR, CYBB, LY96, LILRA2, RASGRP1, LILRA5, SPN, TLR1, IL1A, IL6, PTPRC, IFNG, CLEC7A, PLCG2, IL12B, TNFRSF8, TMEM106A, CCR2, HAVCR2, PF4, TLR2 |
| GOTERM_BP_DIRECT | GO:0031295~T cell costimulation | 15 | 3.12E-08 | 3.44E-06 | CD86, TNFSF14, CCL21, CD80, TNFRSF13C, PDCD1LG2, CD3E, VAV1, TNFSF13B, TMIGD2, CD40LG, CD5, CD28, ICOS, CARD11 |
| GOTERM_BP_DIRECT | GO:0007187~G-protein coupled receptor signaling pathway, coupled to cyclic nucleotide second messenger | 18 | 9.38E-08 | 1.01E-05 | CCR1, PTGIR, XCR1, HTR1E, HTR1F, HTR2B, PTH1R, HTR2A, SSTR2, HTR4, SSTR3, HRH1, HTR7, CNR2, ADRB3, HRH2, CNR1, DRD1 |
| GOTERM_BP_DIRECT | GO:0002407~dendritic cell chemotaxis | 10 | 1.03E-07 | 1.08E-05 | CCR1, CXCR1, CCL21, GPR183, CXCR2, CCR7, CCR6, CCR5, PIK3CG, CCR2 |
| GOTERM_BP_DIRECT | GO:0001819~positive regulation of cytokine production | 18 | 1.20E-07 | 1.21E-05 | IL10, ITK, IL15, CRTAM, HTR2B, PIK3CD, IRAK3, CLEC9A, TNF, PIK3CG, EREG, FGR, IL1A, TMIGD2, IFNG, CLEC5A, CD28, CLEC4E |
| GOTERM_BP_DIRECT | GO:0042130~negative regulation of T cell proliferation | 15 | 1.62E-07 | 1.60E-05 | IL10, CD86, PLA2G2D, CR1, CD80, LILRB1, LILRB2, PDCD1LG2, VTCN1, LILRB4, SPN, IL2RA, CTLA4, CLEC4G, HAVCR2 |
| GOTERM_BP_DIRECT | GO:0035589~G-protein coupled purinergic nucleotide receptor signaling pathway | 9 | 2.50E-07 | 2.41E-05 | P2RY12, P2RY8, P2RY13, P2RY10, P2RY6, P2RY2, P2RY14, GPR171, PTAFR |
| GOTERM_BP_DIRECT | GO:0032733~positive regulation of interleukin-10 production | 14 | 2.63E-07 | 2.46E-05 | SASH3, TSLP, HGF, IL13, LILRA5, IL6, CD40LG, CLEC7A, IRF4, CD28, PLCG2, IL12B, TIGIT, TLR2 |
| GOTERM_BP_DIRECT | GO:0006952~defense response | 19 | 3.82E-07 | 3.49E-05 | CD84, CSF3R, CEBPE, MX2, SP140, HP, CYBB, SSC5D, LILRA1, CXCL13, LILRA2, MPO, CXCL5, LILRB5, STAT4, NOX4, NLRP3, CD48, FCGR2B |
| GOTERM_BP_DIRECT | GO:0030101~natural killer cell activation | 11 | 8.39E-07 | 7.49E-05 | NCR1, NCR3, IL2RB, IL18, IL21R, IL12B, PIK3CD, SLAMF7, RASGRP1, IL2, IL18R1 |
| GOTERM_BP_DIRECT | GO:0044419~interspecies interaction between organisms | 9 | 9.67E-07 | 8.43E-05 | FCER2, CLEC4C, CLEC4D, CD209, CLEC10A, CLEC17A, CLEC4E, CLEC4G, ASGR2 |
| GOTERM_BP_DIRECT | GO:0008284~positive regulation of cell proliferation | 60 | 1.03E-06 | 8.76E-05 | CD86, CSF3, FLT3, IL24, SIRPG, HTR2B, FASLG, HTR2A, PTH1R, GLI1, HAPLN3, AREG, CXCL5, TCL1A, FGF7, FGF9, PROK2, INSL3, SERPINB3, PDGFRA, CASR, CR1, IL15, FLT3LG, PGF, EREG, HCK, ADCYAP1, IFNG, IL1B, KIT, PRTN3, CCL14, PTHLH, PLAC8, CLEC7A, CXCR3, CXCR2, HAS2, SLAMF1, NTRK2, TSLP, IL34, NTRK3, LIF, OSM, AKR1C2, S100B, IL2, BMP5, FOSL1, BST1, MAS1, IL6, FGF18, FOLR2, IL7R, TDGF1, FGF10, CRLF2 |
| GOTERM_BP_DIRECT | GO:0006959~humoral immune response | 16 | 1.26E-06 | 1.05E-04 | AIRE, EBI3, PAX5, TREM1, TNF, BST1, IL6, IFNG, POU2AF1, GPR183, CD28, LTA, PDCD1, CCR6, MS4A1, CCR2 |
| GOTERM_BP_DIRECT | GO:0042531~positive regulation of tyrosine phosphorylation of STAT protein | 17 | 1.31E-06 | 1.07E-04 | TNFSF18, TSLP, IL15, FLT3, IL23R, IL24, IL13, LIF, OSM, IL18, TNF, IL2, GH1, IL6, IFNG, KIT, IL12B |
| GOTERM_BP_DIRECT | GO:0071356~cellular response to tumor necrosis factor | 25 | 1.50E-06 | 1.20E-04 | CCL14, CCL13, CCL11, ADAMTS12, FCAR, CRHBP, TCL1A, CCL8, CCL7, ANKRD1, HAS2, CCL17, CCL16, CCL15, CCL24, CCL23, CCL22, VCAM1, CCL21, DCSTAMP, FABP4, XCL2, TDGF1, HAMP, CCL26 |
| GOTERM_BP_DIRECT | GO:0007169~transmembrane receptor protein tyrosine kinase signaling pathway | 24 | 1.63E-06 | 1.27E-04 | BLK, NTRK1, PDGFRA, NTRK2, MUSK, TRAT1, FLT3, BDNF, EPHA8, NTRK3, SLA, NGF, CD3E, GFRA2, RASGRP4, FGR, HCK, DOK2, DOK5, KIT, BDKRB2, LCP2, MET, EPHB1 |
| GOTERM_BP_DIRECT | GO:0006874~cellular calcium ion homeostasis | 21 | 2.50E-06 | 1.89E-04 | CCR1, CCL14, CCL13, CCL23, CASR, CCL11, PRKCB, HTR2B, ANK2, PTH1R, GRIK2, HTR2A, SLC8A1, CCL8, CCL7, SV2A, RGN, ELANE, CCR2, CCL15, DRD5 |
| GOTERM_BP_DIRECT | GO:0002430~complement receptor mediated signaling pathway | 8 | 2.52E-06 | 1.89E-04 | CR2, CR1, C5AR2, FPR1, C3AR1, FPR3, FPR2, CMKLR1 |
| GOTERM_BP_DIRECT | GO:0032735~positive regulation of interleukin-12 production | 13 | 3.46E-06 | 2.55E-04 | IL23R, DEFB124, IL16, SCIMP, CD40LG, IFNG, CLEC7A, PLCG2, IRF8, IL12B, CCR7, LTB, TLR2 |
| GOTERM_BP_DIRECT | GO:0007202~activation of phospholipase C activity | 11 | 3.85E-06 | 2.73E-04 | P2RY12, CD86, ITK, ADCYAP1R1, P2RY6, GPR55, HTR2B, HTR2A, SELE, S1PR4, RASGRP4 |
| GOTERM_BP_DIRECT | GO:0032740~positive regulation of interleukin-17 production | 10 | 3.86E-06 | 2.73E-04 | IL6, IL15, IL23R, OSM, IL18, IL12B, PRKCQ, SLAMF6, LY9, IL2 |
| GOTERM_BP_DIRECT | GO:0043410~positive regulation of MAPK cascade | 26 | 4.06E-06 | 2.82E-04 | FLT3, TNF, LILRA5, FGF9, CDH2, ADORA1, PLCG2, NTRK1, NTRK2, KSR1, PDE6G, EPHA8, HGF, FCRL3, NTRK3, LIF, OSM, GPR37L1, IL6, PTPRC, ADRB3, DOK5, KIT, TDGF1, TMEM106A, FGF10 |
| GOTERM_BP_DIRECT | GO:0051092~positive regulation of NF-kappaB transcription factor activity | 26 | 4.56E-06 | 3.11E-04 | CX3CR1, TNF, IL18RAP, CLEC7A, PLCG2, S100A12, TNFSF11, NLRP3, TNFSF18, NTRK1, RIPK3, PRKCB, IL18, IRAK3, AIM2, CD40LG, SLCO3A1, IL1B, BTK, PRKCQ, CARD16, TLR6, S100A8, CARD11, IL18R1, TLR2 |
| GOTERM_BP_DIRECT | GO:0050731~positive regulation of peptidyl-tyrosine phosphorylation | 19 | 4.71E-06 | 3.14E-04 | CSF3, IL15, HGF, CD80, LIF, OSM, GFRA1, HTR2A, CD3E, EFNA5, GATA1, GH1, FGF7, IL6, PTPRC, CLEC7A, PLCG2, TDGF1, FGF10 |
| GOTERM_BP_DIRECT | GO:0016064~immunoglobulin mediated immune response | 9 | 4.86E-06 | 3.14E-04 | CD19, INPP5D, IL21R, CD27, TLR8, CSF2RB, FCGR2B, IL13RA2, IL9R |
| GOTERM_BP_DIRECT | GO:0030889~negative regulation of B cell proliferation | 9 | 4.86E-06 | 3.14E-04 | BLK, IL10, TNFRSF13B, CD300A, INPP5D, BTK, CTLA4, MNDA, FCGR2B |
| GOTERM_BP_DIRECT | GO:0032753~positive regulation of interleukin-4 production | 10 | 5.72E-06 | 3.64E-04 | SASH3, CD86, CD40LG, IRF4, CD28, CLECL1, NLRP3, PRKCQ, CD3E, HAVCR2 |
| GOTERM_BP_DIRECT | GO:0030183~B cell differentiation | 17 | 7.22E-06 | 4.51E-04 | IL10, NTRK1, CR2, VCAM1, ITGA4, FLT3, TNFSF13B, CD79B, LYL1, CD79A, PTPRC, CD40LG, KIT, PLCG2, MS4A1, JAK3, CARD11 |
| GOTERM_BP_DIRECT | GO:0050901~leukocyte tethering or rolling | 9 | 7.70E-06 | 4.73E-04 | CX3CR1, SPN, SELPLG, VCAM1, SELL, ITGA4, ITGB7, SELE, TNF |
| GOTERM_BP_DIRECT | GO:0043547~positive regulation of GTPase activity | 28 | 7.95E-06 | 4.81E-04 | CCL14, CCL13, CCL11, RGS16, RASGRF1, RASGRP2, RASGRP1, RASGRP4, DOCK10, DOCK11, CCL8, CCL7, CCL17, CCL16, CCL15, NTRK1, CCL24, CCL23, CCL22, CCL21, ELMOD1, VAV1, RGMA, ADCYAP1, RGN, XCL2, PRTN3, CCL26 |
| GOTERM_BP_DIRECT | GO:0001916~positive regulation of T cell mediated cytotoxicity | 10 | 8.31E-06 | 4.87E-04 | P2RX7, PTPRC, IL23R, IL12B, CD1E, CD1D, IL12RB1, CD1C, CD1B, CD1A |
| GOTERM_BP_DIRECT | GO:0045954~positive regulation of natural killer cell mediated cytotoxicity | 10 | 8.31E-06 | 4.87E-04 | NCR3, RAET1E, IL18RAP, KLRC4, CRTAM, KLRD1, SLAMF6, CD226, RASGRP1, VAV1 |
| GOTERM_BP_DIRECT | GO:0032496~response to lipopolysaccharide | 25 | 9.22E-06 | 5.32E-04 | IL23R, LY96, PTPN22, CSF2RB, FASLG, MPO, CNR2, CNR1, CCR7, ELANE, CD96, PTGIR, VCAM1, IL10RA, IL13, FMO1, IRAK3, SELE, P2RX7, CD6, IL1B, LTA, S100A8, FGF10, TLR2 |
| GOTERM_BP_DIRECT | GO:0071347~cellular response to interleukin-1 | 18 | 1.03E-05 | 5.88E-04 | CCL14, CCL24, CCL13, CCL23, CCL11, CCL22, CCL21, ADAMTS12, KMO, CCL8, CCL7, ANKRD1, XCL2, HAS2, CCL17, CCL16, CCL26, CCL15 |
| GOTERM_BP_DIRECT | GO:0014068~positive regulation of phosphatidylinositol 3-kinase signaling | 17 | 1.41E-05 | 7.91E-04 | NTRK1, PDGFRA, NTRK2, CSF3, NCF1, FLT3, HGF, NTRK3, OSM, IL18, AGAP2, PRR5L, TNF, FGR, GH1, KIT, CD28 |
| GOTERM_BP_DIRECT | GO:0042113~B cell activation | 11 | 1.81E-05 | 9.96E-04 | FCRL1, CD86, CD79A, PRKCB, BTK, PIK3CD, MS4A1, LAX1, IKZF3, RASGRP1, CD22 |
| GOTERM_BP_DIRECT | GO:0048245~eosinophil chemotaxis | 8 | 2.33E-05 | 1.27E-03 | CCL24, CCL13, HRH1, CCL8, CCL11, CCL7, CCL21, CCL26 |
| GOTERM_BP_DIRECT | GO:0030890~positive regulation of B cell proliferation | 12 | 2.78E-05 | 1.49E-03 | SASH3, BST1, PTPRC, GPR183, FCRL3, IL13, NCKAP1L, TNFRSF13C, TNFRSF4, CARD11, IL2, TNFSF13B |
| GOTERM_BP_DIRECT | GO:0031663~lipopolysaccharide-mediated signaling pathway | 11 | 3.09E-05 | 1.63E-03 | HCK, CD6, IL1B, PTAFR, IL18, PLCG2, LY96, BPI, PTPN22, TNF, TLR2 |
| GOTERM_BP_DIRECT | GO:0031640~killing of cells of other organism | 15 | 3.60E-05 | 1.87E-03 | CCL13, PGLYRP4, CCL11, CCL22, CCL21, PPBP, CXCL13, CXCL3, POMC, CCL8, GNLY, S100A12, CCL17, HAMP, PF4 |
| GOTERM_BP_DIRECT | GO:0061844~antimicrobial humoral immune response mediated by antimicrobial peptide | 19 | 4.53E-05 | 2.33E-03 | CCL13, PGLYRP4, CCL11, CCL22, TSLP, CCL21, PPBP, CXCL13, CXCL3, CXCL5, POMC, CCL8, GNLY, S100A12, BPI, CCL17, HAMP, PF4V1, PF4 |
| GOTERM_BP_DIRECT | GO:0048006~antigen processing and presentation, endogenous lipid antigen via MHC class Ib | 5 | 5.12E-05 | 2.56E-03 | CD1E, CD1D, CD1C, CD1B, CD1A |
| GOTERM_BP_DIRECT | GO:0033004~negative regulation of mast cell activation | 5 | 5.12E-05 | 2.56E-03 | MILR1, CD84, CNR2, CNR1, CD300LF |
| GOTERM_BP_DIRECT | GO:0002376~immune system process | 13 | 5.24E-05 | 2.59E-03 | CD300A, HP, FCGR2A, SLFN11, IRF4, CD300LB, IRF8, CD300E, OLR1, VSTM1, PRKCQ, CD300C, CD300LF |
| GOTERM_BP_DIRECT | GO:0043552~positive regulation of phosphatidylinositol 3-kinase activity | 10 | 5.46E-05 | 2.66E-03 | P2RY12, FGR, PDGFRA, CCL21, FLT3, EPHA8, CD19, KIT, CCR7, TNFAIP8L3 |
| GOTERM_BP_DIRECT | GO:0006508~proteolysis | 47 | 6.16E-05 | 2.96E-03 | PCSK2, FCN1, ADAMDEC1, ENPEP, CFH, PAPLN, CFI, FGL2, KLK5, CPZ, TRHDE, ADAMTS4, CAPN8, ADAMTS14, CASP5, ADAM23, CASP1, CLCA2, OLR1, CTSG, ELANE, CPA5, ADAMTS6, CTSC, MMP3, PRSS57, NAALADL1, PM20D1, MMP12, GZMK, CAPN11, GZMM, FAP, MMP16, XPNPEP2, PAPPA, MMP23B, MMP17, ADAM12, DPEP2, PYDC1, MMP19, PRTN3, CPVL, PGPEP1L, TRABD2B, DPEP3 |
| GOTERM_BP_DIRECT | GO:0001501~skeletal system development | 21 | 6.96E-05 | 3.30E-03 | COL13A1, WFIKKN2, HOXD13, GDF3, HOXD12, PTH1R, HAPLN3, PTHLH, HAPLN1, COL19A1, HAPLN2, BMP5, GLI2, PAX1, DLL3, ADAMTS4, BMP3, CLEC3A, EXTL1, MMP16, CMKLR1 |
| GOTERM_BP_DIRECT | GO:0033198~response to ATP | 8 | 7.98E-05 | 3.74E-03 | P2RX7, P2RX6, TRPC3, P2RX2, IL1B, CASP1, ABCC9, SLC8A1 |
| GOTERM_BP_DIRECT | GO:0051482~positive regulation of cytosolic calcium ion concentration involved in phospholipase C-activating G-protein coupled signaling pathway | 10 | 9.21E-05 | 4.26E-03 | P2RY8, GPR174, P2RY10, GPR65, GPR55, C3AR1, DRD1, LPAR4, F2RL2, GPR18 |
| GOTERM_BP_DIRECT | GO:0006909~phagocytosis | 14 | 9.42E-05 | 4.31E-03 | NCF2, CEBPE, NCF4, ICAM3, PLD4, ITGAL, P2RY6, CEACAM4, ADORA1, IRF8, PRTN3, MET, ELANE, SLAMF1 |
| GOTERM_BP_DIRECT | GO:0002523~leukocyte migration involved in inflammatory response | 7 | 1.12E-04 | 4.99E-03 | FUT7, NLRP3, CCR6, SELE, TNF, S100A8, ELANE |
| GOTERM_BP_DIRECT | GO:0032725~positive regulation of granulocyte macrophage colony-stimulating factor production | 7 | 1.12E-04 | 4.99E-03 | IL1B, IL23R, CD80, IL18, IL12B, LILRA2, RASGRP1 |
| GOTERM_BP_DIRECT | GO:0042100~B cell proliferation | 10 | 1.18E-04 | 5.19E-03 | IL10, CD79A, CR2, PTPRC, CD40LG, CD70, IL7R, MS4A1, RASGRP1, CARD11 |
| GOTERM_BP_DIRECT | GO:0007275~multicellular organism development | 28 | 1.25E-04 | 5.43E-03 | CSF3, FLT3, GPR65, HOXD13, MEOX1, TCL1A, TBX21, TNFRSF17, LEFTY2, CCL17, EPHB1, NTRK1, PDGFRA, NFE2, NTRK2, MUSK, EPHA8, NTRK3, LIF, OSM, EN2, WNT9B, PAX5, FOXL1, TBX4, KIT, PDCD1, MET |
| GOTERM_BP_DIRECT | GO:0042104~positive regulation of activated T cell proliferation | 9 | 1.26E-04 | 5.43E-03 | TMIGD2, IL23R, IL2RA, IL18, IL12B, IL12RB1, JAK3, IL2, SLAMF1 |
| GOTERM_BP_DIRECT | GO:0032715~negative regulation of interleukin-6 production | 14 | 1.29E-04 | 5.50E-03 | IL10, CD84, C5AR2, HGF, IRAK3, PTPN22, TNF, LILRB4, CD200R1, INPP5D, BPI, NCKAP1L, HAVCR2, SLAMF1 |
| GOTERM_BP_DIRECT | GO:0046718~viral entry into host cell | 17 | 1.53E-04 | 6.45E-03 | SERPINB3, CD86, CR2, CR1, SELPLG, GPR15, CD80, HTR2A, CD209, MRC1, CLEC5A, NCAM1, ITGB7, CCR5, TNFRSF4, CLEC4G, SLAMF1 |
| GOTERM_BP_DIRECT | GO:0043029~T cell homeostasis | 9 | 1.65E-04 | 6.81E-03 | P2RX7, GPR174, LGALS2, SLC46A2, TNFSF14, RIPK3, SIT1, NCKAP1L, JAK3 |
| GOTERM_BP_DIRECT | GO:0034113~heterotypic cell-cell adhesion | 9 | 1.65E-04 | 6.81E-03 | CD200R1, VCAM1, PTPRC, ITGAD, ITGA4, ITGB7, LILRB2, CD1D, GLDN |
| GOTERM_BP_DIRECT | GO:0050852~T cell receptor signaling pathway | 17 | 1.73E-04 | 7.04E-03 | ITK, BTN1A1, THEMIS, PIK3CD, PTPN22, VTCN1, CD3E, PTPRC, THEMIS2, FCHO1, INPP5D, CD28, PLCG2, CTLA4, LCP2, CD247, SKAP1 |
| GOTERM_BP_DIRECT | GO:0032722~positive regulation of chemokine production | 11 | 1.86E-04 | 7.51E-03 | IL1RL1, IL6, IFNG, TSLP, AIRE, IL18, DEFB124, TLR7, TNF, HAVCR2, TLR2 |
| GOTERM_BP_DIRECT | GO:0045060~negative thymic T cell selection | 6 | 2.12E-04 | 8.36E-03 | PTPRC, AIRE, CD28, CCR7, DOCK2, CD3E |
| GOTERM_BP_DIRECT | GO:1903238~positive regulation of leukocyte tethering or rolling | 6 | 2.12E-04 | 8.36E-03 | FUT7, ITGA4, PTAFR, SELE, ELANE, CCR2 |
| GOTERM_BP_DIRECT | GO:0043303~mast cell degranulation | 7 | 2.50E-04 | 9.78E-03 | MILR1, NR4A3, KIT, PIK3CD, RASGRP1, PIK3CG, PTGDR |
| GOTERM_BP_DIRECT | GO:0001934~positive regulation of protein phosphorylation | 26 | 2.77E-04 | 1.07E-02 | SEMA7A, LRRK2, PRR5L, FPR2, EFNA5, RASGRP1, TNF, FGF7, FGF9, RSPO1, NTRK1, NTRK2, MUSK, IL34, HGF, NTRK3, MOB3B, PGF, MARCO, IFNG, SLCO3A1, CD6, IL1B, FGF18, FAM20A, FGF10 |
| GOTERM_BP_DIRECT | GO:0002224~toll-like receptor signaling pathway | 8 | 2.91E-04 | 1.11E-02 | TLR1, PLCG2, LY96, TLR8, TLR10, TLR7, TLR6, TLR2 |
| GOTERM_BP_DIRECT | GO:0050766~positive regulation of phagocytosis | 11 | 3.26E-04 | 1.23E-02 | IFNG, IL15, CLEC7A, IL1B, IL2RB, SIRPG, FPR2, DOCK2, FCGR2B, TNF, SIRPB1 |
| GOTERM_BP_DIRECT | GO:0048007~antigen processing and presentation, exogenous lipid antigen via MHC class Ib | 5 | 3.27E-04 | 1.23E-02 | CD1E, CD1D, CD1C, CD1B, CD1A |
| GOTERM_BP_DIRECT | GO:0050868~negative regulation of T cell activation | 7 | 3.57E-04 | 1.31E-02 | CTSG, PTPN22, TNFAIP8L2, VTCN1, TIGIT, LAX1, JAK3 |
| GOTERM_BP_DIRECT | GO:0002526~acute inflammatory response | 7 | 3.57E-04 | 1.31E-02 | VCAM1, VNN1, HP, TACR1, TREM1, TNF, S100A8 |
| GOTERM_BP_DIRECT | GO:0051897~positive regulation of protein kinase B signaling | 19 | 3.69E-04 | 1.34E-02 | P2RY12, CX3CR1, CSF3, CCL21, OSM, IL18, PIK3CD, AKR1C2, TNF, PIK3CG, PIK3R5, CALCR, CD28, NOX4, TNFSF11, CCR7, TNFAIP8L3, HPSE, MET |
| GOTERM_BP_DIRECT | GO:0002639~positive regulation of immunoglobulin production | 8 | 3.85E-04 | 1.39E-02 | IL10, SASH3, CD86, IL6, PTPRC, IL13, TNFRSF4, IL2 |
| GOTERM_BP_DIRECT | GO:0001775~cell activation | 6 | 5.36E-04 | 1.91E-02 | TLR1, PDGFRA, CLEC7A, PLCG2, TLR6, TLR2 |
| GOTERM_BP_DIRECT | GO:0098609~cell-cell adhesion | 24 | 5.69E-04 | 2.01E-02 | ROBO2, KIRREL3, ITGAM, VCAM1, NEGR1, CNTN5, COL13A1, ITGA4, DCC, ICAM3, ITGAL, COL19A1, LGALS2, VNN1, SRPX2, ITGAD, CDH2, IGDCC4, CLEC7A, FAT3, FAT4, SIGLEC1, ICOS, CD33 |
| GOTERM_BP_DIRECT | GO:0032689~negative regulation of interferon-gamma production | 10 | 6.22E-04 | 2.13E-02 | IL10, CD96, IL1RL1, CR1, NLRP6, LILRB1, PDCD1LG2, LILRB4, HAVCR2, SLAMF1 |
| GOTERM_BP_DIRECT | GO:0002638~negative regulation of immunoglobulin production | 5 | 6.24E-04 | 2.13E-02 | CR1, FCRL3, FCGR2B, IL13RA2, CD22 |
| GOTERM_BP_DIRECT | GO:2000556~positive regulation of T-helper 1 cell cytokine production | 5 | 6.24E-04 | 2.13E-02 | IL1B, TBX21, IL18, IL18R1, SLAMF1 |
| GOTERM_BP_DIRECT | GO:0050859~negative regulation of B cell receptor signaling pathway | 5 | 6.24E-04 | 2.13E-02 | CD300A, FCRL3, PLCL2, FCGR2B, CD22 |
| GOTERM_BP_DIRECT | GO:0042832~defense response to protozoan | 8 | 6.43E-04 | 2.16E-02 | IL10, GBP6, CLEC7A, IRF4, IRF8, IL12B, TSPAN32, PF4 |
| GOTERM_BP_DIRECT | GO:0034765~regulation of ion transmembrane transport | 19 | 6.46E-04 | 2.16E-02 | CLIC6, CATSPER1, KCNF1, KCNG2, HVCN1, KCNA2, CACNA2D3, KCNA3, KCNJ15, CYBB, CACNA1F, NALCN, CACNG6, TMEM37, CALHM1, SCN5A, SCN3A, KCNH1, KCNJ3 |
| GOTERM_BP_DIRECT | GO:0055085~transmembrane transport | 27 | 7.90E-04 | 2.57E-02 | SLC46A2, SLC47A1, ABCB1, SLC43A3, ABCB4, SLC35F4, PRF1, AQP5, GJC2, SV2A, SLCO2B1, SLC16A4, MFSD2A, SLC22A11, SLC10A4, SLC14A2, ABCA6, SPNS3, ABCC9, ABCA8, SLC51B, ANO5, SLC4A8, SLCO3A1, FXYD2, SLCO4C1, CFTR |
| GOTERM_BP_DIRECT | GO:0030595~leukocyte chemotaxis | 6 | 7.94E-04 | 2.57E-02 | IL10, CX3CR1, CNR2, GPR183, IL16, PF4 |
| GOTERM_BP_DIRECT | GO:0097028~dendritic cell differentiation | 6 | 7.94E-04 | 2.57E-02 | BLK, BATF3, FLT3, IRF8, LILRB1, GATA1 |
| GOTERM_BP_DIRECT | GO:0045580~regulation of T cell differentiation | 6 | 7.94E-04 | 2.57E-02 | SLC46A2, IL15, TBX21, CRTAM, CARD11, CCR2 |
| GOTERM_BP_DIRECT | GO:0007189~adenylate cyclase-activating G-protein coupled receptor signaling pathway | 18 | 8.58E-04 | 2.75E-02 | PTGIR, GPR78, GPR65, GPR3, PTH1R, ADCY7, PTHLH, ADCYAP1, CALCR, CNR2, ADRB3, CNR1, CXCR3, DRD1, S1PR4, GPHA2, PF4, DRD5 |
| GOTERM_BP_DIRECT | GO:0019233~sensory perception of pain | 11 | 8.74E-04 | 2.78E-02 | P2RX7, ADCYAP1, CNR2, CNR1, HOXD1, KCNA2, PROK2, IL12B, TACR1, HOXB8, CCR2 |
| GOTERM_BP_DIRECT | GO:0032757~positive regulation of interleukin-8 production | 12 | 9.43E-04 | 2.97E-02 | TLR1, FCN1, IL6, CLEC7A, IL1B, TLR8, TLR7, LILRA2, TNF, CD244, ELANE, TLR2 |
| GOTERM_BP_DIRECT | GO:0001774~microglial cell activation | 8 | 1.02E-03 | 3.19E-02 | TLR1, ITGAM, IFNG, IL13, FPR2, TLR6, TNF, TLR2 |
| GOTERM_BP_DIRECT | GO:0008037~cell recognition | 5 | 1.07E-03 | 3.27E-02 | NCR3, CLEC7A, CD5, CRTAM, CD226 |
| GOTERM_BP_DIRECT | GO:0060732~positive regulation of inositol phosphate biosynthetic process | 5 | 1.07E-03 | 3.27E-02 | MAS1, ADCYAP1R1, PTAFR, PTH1R, CD244 |
| GOTERM_BP_DIRECT | GO:0046641~positive regulation of alpha-beta T cell proliferation | 5 | 1.07E-03 | 3.27E-02 | PTPRC, EBI3, CD28, CD3E, CCR2 |
| GOTERM_BP_DIRECT | GO:0002755~MyD88-dependent toll-like receptor signaling pathway | 7 | 1.18E-03 | 3.57E-02 | TLR1, BTK, TLR8, TLR10, IRAK3, TLR6, TLR2 |
| GOTERM_BP_DIRECT | GO:0030574~collagen catabolic process | 9 | 1.19E-03 | 3.57E-02 | MMP12, ADAMTS14, MMP16, MMP23B, MMP17, MMP3, MMP19, PRTN3, KLK6 |
| GOTERM_BP_DIRECT | GO:0007159~leukocyte cell-cell adhesion | 8 | 1.27E-03 | 3.77E-02 | VCAM1, PTPRC, CD40LG, ITGA4, CD209, OLR1, ITGAL, SELE |
| GOTERM_BP_DIRECT | GO:0032720~negative regulation of tumor necrosis factor production | 13 | 1.38E-03 | 4.06E-02 | IL10, C5AR2, LILRB1, IRAK3, PTPN22, LILRB4, LILRA4, POMC, BPI, CD33, GPR18, HAVCR2, SLAMF1 |
| GOTERM_BP_DIRECT | GO:0051781~positive regulation of cell division | 10 | 1.43E-03 | 4.19E-02 | IL1A, FGF7, FGF9, MACC1, IL1B, OSM, HTR2B, PPBP, PGF, EREG |
| GOTERM_BP_DIRECT | GO:0007218~neuropeptide signaling pathway | 16 | 1.52E-03 | 4.35E-02 | NPY5R, PMCH, SORCS1, GPR84, NMBR, SSTR2, SORCS2, SSTR3, HCRTR1, POMC, ADCYAP1, CYSLTR2, PNOC, SCG5, KISS1R, PROK2 |
| GOTERM_BP_DIRECT | GO:0032693~negative regulation of interleukin-10 production | 7 | 1.52E-03 | 4.35E-02 | IL23R, IL12B, LILRB1, PDCD1LG2, FCGR2B, JAK3, LILRB4 |
| GOTERM_BP_DIRECT | GO:0070269~pyroptosis | 7 | 1.52E-03 | 4.35E-02 | ZBP1, AIM2, NLRP6, GZMA, CASP1, NLRP1, ELANE |
| GOTERM_BP_DIRECT | GO:0061760~antifungal innate immune response | 6 | 1.57E-03 | 4.47E-02 | CX3CR1, CLEC4C, CLEC4D, CLEC7A, PLCG2, CLEC4E |
| GOTERM_BP_DIRECT | GO:0050727~regulation of inflammatory response | 14 | 1.63E-03 | 4.59E-02 | ZBP1, SEMA7A, CMA1, ADAMTS12, SELE, ESR1, BST1, MAS1, HCK, CASP5, NLRP6, CASP1, NLRP1, CCR2 |
| GOTERM_BP_DIRECT | GO:0050776~regulation of immune response | 9 | 1.69E-03 | 4.70E-02 | SPN, FCGR2A, FCGR3B, FCER1A, PDCD1, FCGR1A, FCGR2B, FCGR2C, CD22 |
| GOTERM_BP_DIRECT | GO:0002544~chronic inflammatory response | 5 | 1.71E-03 | 4.70E-02 | CCL11, VCAM1, VNN1, CXCL13, S100A8 |
| GOTERM_BP_DIRECT | GO:0033089~positive regulation of T cell differentiation in thymus | 5 | 1.71E-03 | 4.70E-02 | VNN1, TESPA1, IL7R, RASGRP1, GLI2 |
| GOTERM_BP_DIRECT | GO:0002732~positive regulation of dendritic cell cytokine production | 4 | 1.72E-03 | 4.71E-02 | CLEC7A, KIT, PLCG2, SCIMP |
| GOTERM_CC_DIRECT | GO:0005887~integral component of plasma membrane | 250 | 9.86E-59 | 2.96E-56 | HHIP, AQP9, ICAM3, AQP5, HTR4, GPR174, MILR1, HTR7, C3AR1, TNFSF11, EPHB1, CMKLR1, MFSD2A, SLC34A2, MUSK, EPHA8, EREG, NINJ2, CD226, CFTR, ADCYAP1R1, ENPEP, IGSF6, ABCB4, GYPE, SLC22A2, CD1E, LPAR4, CD1D, PCDH19, CD1C, SLC6A20, CD1A, CD79B, FLRT2, CD19, SYNDIG1, NCKAP1L, PROM1, CCR10, CCR1, MSR1, XCR1, GPR15, NMBR, GP5, MARCO, GPR183, FXYD1, CD28, CD27, CHRFAM7A, TNFSF8, GPR18, CD22, GPR25, SIGLEC9, TNFRSF13B, CD3G, PTH1R, GRIK2, PTPRH, CD3E, FCRLA, MRC1, CCR9, FCER1A, CCR8, OLR1, CTLA4, CD37, CCR6, CCR5, CCR4, CCR3, CD33, SLC22A11, CCR2, FCRL1, FCRL2, TMEM150B, FCRL5, FCRL6, FCRL3, TMC8, GPBAR1, GPR31, TACR1, SORCS2, NCR1, TLR1, FCER2, NCR3, SLC7A7, ADRB3, MMP16, CEACAM4, KIT, B3GNT3, TLR10, TLR6, TLR2, SELPLG, PTAFR, KCNA2, CXCR6, LILRA2, ADCY7, LILRA4, GPA33, CXCR3, HAS1, ADAM23, CXCR2, KISS1R, HAS2, SLC17A3, P2RY10, CD72, CD70, IL10RA, GPR55, LILRB2, GPR3, LYVE1, XG, MAS1, CD40LG, SLCO3A1, SLCO4C1, CD69, LGR6, SIGLEC7, F2RL2, SIGLEC6, CD84, CSF3R, GPR68, GPR65, LY75, TNF, SLC8A1, CYSLTR2, CDH2, SIT1, SLCO2B1, ADORA3, CLEC5A, CCRL2, ADORA1, SLC16A7, PLXNC1, TNFRSF4, SLC16A4, KCNH1, KIRREL3, PDGFRA, CD96, CASR, SCARA5, BTN1A1, CYBB, CD300C, TRABD2B, CLEC4G, CHRNA1, GRIA2, SEMA7A, HVCN1, CHRNA6, CSF2RB, GPR84, TRHDE, CSF2RA, CALHM1, HRH1, CALCR, HRH2, BTLA, CLCA2, DRD1, SLC38A3, SLC38A5, STRA6, DRD5, CD163, VCAM1, HTR1E, HTR1F, TNFRSF9, KLRC4, TRPV2, TRPV3, SELE, PTPRC, SELL, PRPH2, KLRD1, FLT3, HTR2B, FASLG, HTR2A, TSPAN11, SIRPB1, FCAR, SPN, FCGR3B, BDKRB2, PTGIR, TRPC6, CR1, TRPC3, NPY5R, TRPC4, SSTR2, SSTR3, MS4A2, MET, MS4A1, IL9R, CX3CR1, SEMA3A, TSPAN32, P2RY8, P2RY6, CNR2, CNR1, P2RY2, FCGR1A, S1PR4, NTRK1, NTRK2, PCDH9, TRAT1, NTRK3, KCNJ15, HCRTR1, P2RX7, P2RX6, FCGR2A, CLEC2B, CD6, CD5, P2RX2, IL2RB, ACKR4, EVI2B, FCGR2B, KCNK2, FCGR2C |
| GOTERM_CC_DIRECT | GO:0005886~plasma membrane | 535 | 1.21E-58 | 2.96E-56 | CLEC10A, C4BPA, MRGPRE, HTR4, GLDN, HTR7, EVA1A, GPR171, C3AR1, MS4A14, CMKLR1, PRKCB, MCEMP1, DKK1, TAAR1, SLC5A9, BIN2, PRKCQ, ENTHD1, SKAP1, ENPEP, ABCB1, COL13A1, ABCB4, FPR1, FPR3, FPR2, LY9, SLC5A5, FLRT2, PLCG2, S100A12, NCKAP1L, ICOS, PROM1, CADM3, GPR15, ABCA6, APCDD1L, ABCA8, CP, GP5, TREML1, DCHS2, FXYD2, GPR183, GPR182, FXYD1, PNOC, TREML2, NOX4, FXYD7, FAT3, TNFSF8, FAT4, XKR4, GPR18, GAPT, ITK, GPR25, SIGLEC9, MS4A4E, PTH1R, MS4A4A, ASGR2, GPR132, MRC1, CTLA4, OLR1, DLGAP2, JAK3, FCRL6, GPR31, TACR1, PRSS12, APBB1IP, FCER2, MS4A6A, CD200R1, SLC7A7, MMP16, MMP17, TLR10, DSCAML1, TMEM106A, GAS7, ERVFRD-1, PTAFR, KCNA2, CLECL1, KCNA3, ADCY7, GPA33, HAS1, ADAM23, CSMD3, IL12RB1, SLC17A3, P2RY12, P2RY13, P2RY10, OR10G2, IL10RA, P2RY14, GPR3, SNX20, GPR141, GPR142, SLCO3A1, SLCO4C1, SIGLEC1, SIGLEC8, LGR6, SIGLEC7, SIGLEC6, SIGLEC5, CRLF2, RAB3C, KCNG2, CSF3R, NCF1, GPR68, GPR65, TNF, SLC8A1, RAB44, BEST4, CYSLTR2, CDH2, SLCO2B1, ADORA3, GRAP2, CLEC5A, ADORA1, TNFRSF8, SLC16A7, PLXNC1, TNFRSF4, KCNH1, PPP1R16B, GPR78, BTN1A1, CD180, DIO3, CLEC4C, CLEC4D, FRMD7, RAB37, RAB38, S100A3, CLEC4E, S100A8, CLEC4G, GRIA2, SEMA7A, KCNE1, KCNE5, HVCN1, LY96, GPR84, GPR82, CLEC7A, BTLA, CLCA2, CD300LB, TNFRSF17, DRD1, CD300LF, LRRC4C, SLC38A3, SLC38A5, GRIA3, STRA6, DRD5, KCNF1, CD163, SLC14A2, HTR1E, HTR1F, TNFRSF9, PTPRE, BFSP2, PTPRC, SNAP25, ITGAM, FLT3, LRRK2, SCHIP1, RASGRF1, HTR2B, PIK3CD, PIK3C2G, FASLG, TRH, HTR2A, ITGAL, TREM1, FCAR, PIK3CG, TNFSF13B, SPN, PSTPIP1, FCGR3B, ITGB7, SGCG, CR2, TRPC6, CR1, TRPC3, ITGA4, TRPC4, SGIP1, ITGAD, MET, CATSPER1, CX3CR1, PLEKHH2, SLC47A1, CACNG6, P2RY6, FCHO1, P2RY2, IL21R, SLITRK5, FCGR1A, TIGIT, KCNJ3, NTRK1, NTRK2, KCNIP1, PCDH9, NTRK3, KCNJ15, HCRTR1, P2RX7, P2RX6, FCGR2A, CLEC2B, P2RX2, SYT10, IL2RA, IL2RB, PDCD1, CD247, FCGR2B, FOLR2, KCNK2, CD244, TRAF3IP3, SLC46A2, CPNE5, IL23R, AQP9, PRF1, ICAM3, MLC1, AQP5, OR52N4, VSIG1, INSC, PIEZO2, TNFSF11, EPHB1, PTGDR, MFSD2A, ARL11, SLC34A2, ARL14, MUSK, EPHA8, IL1R2, KRT1, FLT3LG, ANK2, GPR37L1, SLC51B, BTK, CD226, CFTR, GPM6A, ADCYAP1R1, RRAD, SLC22A2, GYPE, EBI3, LPAR4, CD1D, PCDH19, SLC6A20, CD1C, CD1B, PIK3R6, CD1A, PIK3R5, CD79B, CD79A, OTOA, NKAIN3, INPP5D, CD19, SLAMF7, SLAMF6, SCN3A, CCR10, SLC10A4, CCR1, TNFSF18, MSR1, XCR1, SIGLEC14, TNFSF14, SIGLEC11, SIGLEC10, NMBR, MARCO, BST1, RAET1E, VNN1, PARVG, LPAR5, CD209, CD28, CD27, TDGF1, CD22, SCN2B, CLIC6, ROBO2, TNFRSF13B, CD3G, GRIK2, TNFRSF13C, CD3E, GLIPR1, KCNT2, SLC22A16, NMRK2, CCR9, FCER1A, CCR8, TNFAIP8L3, CCR7, CTSG, CCR6, CLIC2, CCR5, CCR4, CCR3, CD33, CCR2, SLC22A11, GPBAR1, IZUMO1R, IL16, GFRA1, PDCD1LG2, ANO5, GFRA2, RAB33A, VAV1, RGMA, NAALADL1, CEACAM3, NCR1, CLDN11, TLR1, IL1A, DCSTAMP, HCK, NCR3, ADRB3, CEACAM4, KIT, CLDN18, TLR8, TLR7, CD48, TLR6, TLR2, LILRA6, GUCY2C, SELPLG, ADH1B, CXCR6, LILRA1, LILRA2, STX11, LILRA4, LILRA5, NKD2, DRP2, REM1, PTCHD1, CXCR1, CXCR3, CXCR2, KISS1R, SUSD3, LAIR2, LY6G6D, LAIR1, MICB, CD72, CD70, LILRB1, LILRB2, LYVE1, LILRB4, LILRB5, MAS1, GFI1B, CD40LG, FAP, KCNS2, CD68, F2RL2, IL18R1, CD86, CD84, OR5K2, CD80, SIRPG, IL18RAP, SIT1, CCRL2, ENPP6, PRND, RGS9, KIRREL3, PDGFRA, CD96, CASR, CD300A, CYBB, LAX1, ADAM19, GPRIN3, ADAM12, CD300E, AMPH, PRTN3, CD300C, CARD11, DPEP3, CHRNA1, KLRB1, C5AR2, CHRNA6, DAPP1, CSF2RB, CABP1, EFNA5, RASGRP2, NALCN, TRHDE, SLC1A7, CSF2RA, RASGRP1, RASGRP4, HRH1, SCUBE1, CALCR, NLRP6, HRH2, CDH23, OR5H8, VCAM1, CNTN5, MUC16, CRTAM, TRPV2, TRPV3, ESR1, SELE, LRP1B, SELL, CNTN2, KLRD1, IL7R, FGF10, RTP1, MS4A7, PLEK, SIRPB2, RND2, SIRPB1, BDKRB2, CASP1, SCN5A, PTGIR, RASA4B, NPY5R, DCC, ABCC9, IRAK3, SSTR2, SSTR3, FGR, GAP43, TMIGD2, IGDCC4, LTA, LTB, MS4A2, DOCK2, MS4A1, CDH17, IL9R, CAMK1G, RGS18, CLEC12A, RGS13, RGS16, IL1RL1, GNG2, CYTH4, CLMP, CNR2, CNR1, SV2A, GNG8, NCAM1, EPS8L3, S1PR4, KLRG1, MAS1L, NEGR1, PDE6G, TRAT1, MX2, ZNF804A, SLC4A8, XPNPEP2, CD5, ACKR4 |
| GOTERM_CC_DIRECT | GO:0009897~external side of plasma membrane | 122 | 8.40E-49 | 1.37E-46 | CD86, CD84, CSF3R, CD80, CLEC10A, IL23R, LY75, TNF, CCRL2, IL12B, IL13RA2, TNFRSF4, PDGFRA, SCARA5, BTN1A1, CLEC4C, CLEC4D, CD226, CLEC4E, CLEC4G, GRIA2, SEMA7A, ENPEP, EBI3, CD1E, CSF2RB, VTCN1, CD1D, CD1C, CD1B, CSF2RA, LY9, CD1A, CD79B, CD79A, SCUBE1, CLEC7A, CD19, SLAMF7, SLAMF6, CCR10, SLAMF1, CCR1, MSR1, CD163, XCR1, VCAM1, TNFRSF9, KLRC4, CLEC17A, SELE, RAET1E, PTPRC, CD209, CD28, CD27, KLRD1, IL7R, CD22, ITGAM, FASLG, CD3G, TNFRSF13C, ITGAL, CD3E, ASGR2, SERPINA5, SPN, CCR9, CCR8, CTLA4, CCR7, CCR6, CCR5, CCR4, CD33, CCR3, SLC22A11, CCR2, ITGA4, FCRL6, IL13, GFRA1, PDCD1LG2, GFRA2, FCER2, CD200R1, ITGAD, KIT, TLR8, CD48, MS4A2, MS4A1, IL9R, CX3CR1, CXCR6, IL1RL1, CXCR1, CXCR3, CXCR2, IL21R, NCAM1, IL12RB1, LY6G6D, KCNJ3, MICB, P2RY12, LILRB1, P2RX7, IGLL5, CD40LG, CLEC2B, CD6, CD5, IL2RA, IL2RB, ACKR4, PDCD1, CD69, FCGR2B, CD244, CRLF2 |
| GOTERM_CC_DIRECT | GO:0016021~integral component of membrane | 497 | 2.99E-33 | 3.67E-31 | TMEM200A, CLEC10A, TMEM200C, MRGPRE, GLDN, RNF112, HTR7, EVA1A, GPR171, C3AR1, SMCO2, MS4A14, VSTM1, CMKLR1, CACNA2D3, MCEMP1, TAAR1, CH25H, SLC5A9, ENPEP, ABCB1, HS3ST3A1, ABCB4, FPR1, FPR3, FPR2, LY9, SLC5A5, HSD11B1, GJC2, CHST11, CHST13, ICOS, PROM1, CADM3, GPR15, ABCA6, APCDD1L, ABCA8, TREML1, DCHS2, GPR183, GPR182, FXYD1, TREML2, NOX4, FXYD7, FAT3, CHRFAM7A, TNFSF8, FAT4, XKR4, GPR18, GAPT, SIGLEC9, MS4A4E, PTH1R, AREG, MS4A4A, ASGR2, M1AP, GPR132, CTLA4, OLR1, HAVCR2, FCRL2, CHST6, FCRL3, TACR1, FCER2, TMEM37, MS4A6A, CD200R1, SLC7A7, TLR10, DSCAML1, TMEM106A, ERVFRD-1, PTAFR, KCNA2, CLECL1, KCNA3, ADCY7, TMEM71, EXTL1, SLC17A9, HAS1, ADAM23, GCNT4, CSMD3, SLC17A3, RPRM, P2RY12, LRRC25, EVC2, P2RY13, OR10G2, IL10RA, P2RY14, GPR55, FMO1, FMO2, FMO3, GPR3, GPR141, TMEM156, GPR142, SLCO3A1, A4GNT, SIGLEC1, SIGLEC8, SIGLEC6, SIGLEC5, CRLF2, KCNG2, CSF3R, GPR68, GPR65, LY75, CXCL13, TNF, SLC8A1, ATCAY, CYSLTR2, CDH2, SLCO2B1, ADORA3, CLEC5A, ADORA1, TNFRSF8, SLC16A7, IL13RA2, HLA-DOB, SLC16A4, GPR78, BTN1A1, CD180, DIO3, CLEC4C, CLEC4D, MFNG, MAL, CLEC4E, CLEC4G, CHODL, GRIA2, KCNE1, KCNE5, HVCN1, VTCN1, CYP19A1, GPR82, CLEC7A, CD300LB, TNFRSF17, SLC15A3, CD300LF, LRRC4C, SLC38A3, RNF175, SLC38A5, GRIA3, STRA6, TMEM132C, KCNF1, CD163, SLC14A2, BDNF, TNFRSF9, TVP23A, PTPRE, TMIE, PTPRC, RNF186, ITGAM, FLT3, HTR2B, FASLG, ITGAL, TSPAN11, SYNE3, TREM1, FCAR, TNFSF13B, ADAMTS4, SPN, FCGR3B, GAL3ST3, SGCG, SLFN12L, GAL3ST4, CR2, TRPC6, CR1, TRPC3, ITGA4, TRPC4, TMEM255A, SEZ6L, CYP2A7, CYP2A6, ACTBL2, PKHD1L1, MET, CX3CR1, SLC47A1, TSPAN32, P2RY8, CACNG6, P2RY6, P2RY2, IL21R, CEACAM21, SLITRK5, FCGR1A, TIGIT, KCNJ3, LINGO2, NTRK2, PCDH9, NTRK3, KCNJ15, TM6SF1, TM6SF2, HCRTR1, P2RX7, P2RX6, EVI2A, FCGR2A, SYT10, IL2RA, IL2RB, PDCD1, CD247, EVI2B, FCGR2B, KCNK2, FCGR2C, CD244, TRAF3IP3, SLC46A2, IL23R, AQP9, LST1, PRF1, ICAM3, MLC1, SOGA3, AQP5, OR52N4, NIPAL4, AADACL4, VSIG1, PIEZO2, TNFSF11, EPHB1, PTGDR, MFSD2A, SLC34A2, TMEM52B, MUSK, IL1R2, FLT3LG, GPR37L1, CPT1C, SLC51B, SOAT2, NINJ2, PAPPA, CD226, CFTR, GPM6A, ADCYAP1R1, IGSF6, SLC22A2, GYPE, CD1E, CD1D, PCDH19, CD1B, CD1A, CD79B, CD79A, NKAIN3, CD19, ARSH, SLAMF8, ST8SIA4, OTOF, SLAMF7, SLAMF6, SCN3A, CCR10, SLAMF1, SLC10A4, CCR1, TNFSF18, MSR1, XCR1, SIGLEC14, TNFSF14, SIGLEC11, SIGLEC10, NMBR, MARCO, RAET1E, VNN1, LPAR5, CD209, CD28, TMCC2, TDGF1, CD22, ROBO2, SLC35F4, CD3G, CLEC9A, GRIK2, TNFRSF13C, CD3E, GLIPR1, KCNT2, SLC22A16, CCR9, FCER1A, CCR8, CCR7, CD37, CCR5, CCR4, CCR3, CD33, CCR2, SLC22A11, TMEM150B, GPBAR1, SORCS1, GAB3, PDCD1LG2, ANO5, SORCS2, NAALADL1, CEACAM3, NCR1, CLDN11, TLR1, DCSTAMP, NCR3, ADRB3, MMP23B, CEACAM4, KIT, B3GNT3, CLDN18, TLR8, TLR7, CD48, TLR6, TLR2, LILRA6, GUCY2C, SELPLG, SLC43A3, CXCR6, LILRA1, STX11, LILRA5, PTCHD1, CXCR1, CXCR3, HSD17B2, CXCR2, MGAT3, KISS1R, SUSD3, LY6G6D, LAIR1, MICB, CD70, TMEM176A, LILRB1, LILRB2, LYVE1, LILRB4, LILRB5, XG, MAS1, CD40LG, FAP, KCNS2, LRRN4CL, CD69, CD68, F2RL2, IL18R1, CD86, NRK, OR5K2, CD80, SIRPG, GIMAP1, FAIM2, GIMAP5, GXYLT2, FAM162B, SIT1, CCRL2, MALRD1, PRND, KIRREL3, CD96, CASR, CD300A, SPNS3, CYBB, KMO, LAX1, ERMN, MPEG1, ADAM19, ERN2, ADAM12, CD300E, CHRNA1, FBN2, KLRB1, C5AR2, CHRNA6, PLD4, CSF2RB, SEL1L2, CACNA1F, NALCN, SLC1A7, FUT7, CALCR, HRH2, CDH23, OR5H8, VCAM1, MUC16, WSCD2, KLRC4, RARRES1, CRTAM, TRPV2, CLEC17A, TRPV3, EPHX3, ESR1, SELE, LRP1B, SYNDIG1L, SELL, FAM151A, PRPH2, FAM163A, KLRD1, IL7R, RTP5, ABCD2, RTP1, MS4A7, SIRPB2, SIRPB1, BDKRB2, SCN5A, GALNT9, GALNT8, PTGIR, DCC, ABCC9, SYNGR3, TMIGD2, IGDCC4, LTB, CDH17, IL9R, TMEM45A, CLEC12A, DLL3, AGPAT4, IL1RL1, CLMP, CNR2, CNR1, SV2A, NCAM1, KLRG1, MAS1L, CMTM5, ZNF804A, HS3ST5, SCIMP, SLC4A8, CD6, CD5 |
| GOTERM_CC_DIRECT | GO:0009986~cell surface | 107 | 1.42E-23 | 1.40E-21 | CD86, SLC46A2, HHIP, CD80, TNF, GLDN, CDH2, TNN, CLEC5A, TNFRSF4, KCNH1, CASR, FLT3LG, CD226, CFTR, CHRNA1, ADCYAP1R1, KCNE1, ABCB1, CD1D, CD1B, LY9, OTOA, SCUBE1, CLEC7A, SLAMF8, PROM1, CCR10, ELANE, SLAMF1, TNFSF18, VCAM1, TRPV2, CLEC17A, TREML1, LRFN5, PTPRC, CD209, TREML2, CD28, CNTN2, TDGF1, CD22, FGF10, ROBO2, CIITA, ITGAM, RTP1, CLEC9A, ITGAL, AREG, SIRPB1, SPN, MRC1, CCR9, FCER1A, ITGB7, CCR7, CTSG, SCN5A, CCR6, CCR5, CD33, HAVCR2, FCRL1, FCRL2, CR1, ITGA4, FCRL5, IL15, TRPC4, DCC, FCRL3, TACR1, RGMA, CEACAM3, DCSTAMP, IL1A, CD200R1, ITGAD, MMP16, CEACAM4, DSCAML1, MET, MS4A1, CDH17, TLR2, CX3CR1, TSPAN32, LILRA5, SRPX2, CLMP, CXCR2, KISS1R, NCAM1, TIGIT, MICB, P2RY12, NTRK1, LILRB2, LILRB4, MAS1, CD40LG, FAP, IL2RB, FOLR2, KCNK2 |
| GOTERM_CC_DIRECT | GO:0005576~extracellular region | 226 | 1.33E-20 | 1.08E-18 | PGLYRP4, ADAMDEC1, HHIP, PRF1, C4BPA, ISM1, ISM2, VPREB3, CRHBP, LIPC, IL12B, PROK2, TNFSF11, EPHB1, IL1R2, KRT1, FLT3LG, RNASE2, DKK1, DKK2, EREG, CHIT1, BIN2, OPTC, PAPPA, PLBD1, PADI2, CFH, COL13A1, CFI, EBI3, KLK5, ADAMTS12, KLK6, PLAC8, OTOA, ADAMTS14, ARSI, S100A12, FAM180A, ICOS, ELANE, TSLP, SIGLEC10, LIF, PRG2, PPBP, CP, PM20D1, BST1, BPIFB1, VNN1, CD209, PNOC, CD27, TDGF1, HAMP, SFTA2, ARHGAP9, IL24, WFIKKN2, AREG, SERPINA5, C1QTNF2, C1QTNF7, ADAMTSL4, GNLY, OLR1, CTSG, RSPO1, CTSC, IL10, SERPINB3, IL15, IL13, IL18, WNT9B, IL16, IZUMO1R, HBA1, PDCD1LG2, PRSS12, MMP12, IL1A, CD200R1, MMP16, IL1B, MMP17, COL21A1, MMP19, DNASE1L3, CCL13, CCL11, SAA4, PAPLN, DEFB124, AOAH, LILRA2, COL19A1, LILRA5, ABI3BP, ADAM23, LAIR2, LY6G6D, GPHA2, CCL17, LEFTY2, CCL16, CCL23, CCL22, CCL21, IL34, OSM, LILRB1, PRSS35, SIGLEC1, F2RL2, SIGLEC6, PF4, CRLF2, FCN1, CSF3, CSF3R, LRCOL1, PMCH, HP, F13A1, CXCL13, MPO, TNF, FGF7, FGF9, SVEP1, ENPP6, IL13RA2, PRND, KIRREL3, SPINK2, HGF, KERA, PGF, MPEG1, ADCYAP1, COL4A4, ADAM12, SCG5, PRTN3, S100A8, FBN2, LY96, CSF2RA, CLCA2, NLRP3, CPA5, CD163, CNTN5, BDNF, IL2, BMP5, GH1, BMP3, IL6, FGF14, FGF18, SPINK13, HPSE, IL7R, FGF10, TNFAIP6, PLEK, FASLG, TRH, CXCL3, HAPLN3, TREM1, FCAR, CXCL5, HAPLN1, HAPLN2, TNFSF13B, ADAMTS4, HK3, FCGR3B, CASP1, FDCSP, INSL3, TSHB, ADAMTS6, MMP3, NGF, FGR, CFAP221, METTL24, IFNG, MMRN1, CHI3L2, XCL2, BPI, DOCK2, MET, ITIH3, SEMA3A, PRCD, FGL2, TULP2, RETN, PTHLH, IL1RL1, CCL7, NCAM1, PLA2G2D, NEGR1, CMA1, PLA2G2C, GZMA, LGI2, S100B, POMC, GZMK, NELL1, GZMM, CD6, XPNPEP2, MNDA, FOLR3, FOLR2 |
| GOTERM_CC_DIRECT | GO:0005615~extracellular space | 202 | 5.64E-18 | 3.95E-16 | C4BPA, SOGA3, GLDN, VPREB3, CRHBP, LIPC, IL12B, VSTM1, TNFSF11, KRT1, FLT3LG, RNASE2, DKK1, DKK2, EREG, KRT9, CHIT1, PAPPA, PLBD1, WFDC10B, CFH, COL13A1, CFI, EBI3, KLK5, CD1E, CD1D, CD1C, CD1B, CD1A, KLK6, FLRT2, S100A12, PROM1, ELANE, TNFSF18, TNFSF14, TSLP, LIF, PPBP, GDF3, GP5, CP, BPIFB1, RAET1E, TNFSF8, TDGF1, HAMP, PCSK2, IL24, WFIKKN2, CHRDL2, AREG, SERPINA5, GLIPR1, GNLY, SOSTDC1, CTSG, CTSC, PF4V1, IL10, SERPINB3, FGFBP2, IL15, IL13, IL18, WNT9B, TMC8, IL16, SERPINB9, HBA1, SERPINB7, MMP12, IL1A, MMP23B, IL1B, KIT, COL21A1, MMP19, ANGPTL5, FAM20A, DSCAML1, CCL14, CCL13, CCL11, CST7, COL19A1, LILRA5, SRPX2, ABI3BP, GPHA2, CCL17, LEFTY2, CCL16, MICB, CCL15, CCL24, CCL23, CCL22, CCL21, CD70, IL34, OSM, LILRB2, CD40LG, FAP, CR1L, CFHR3, SCRG1, PF4, CCL26, FCN1, CSF3, HP, CLEC18B, CXCL13, MPO, TNF, FGF7, PODNL1, FGF9, TNN, SVEP1, IL13RA2, HGF, BTN1A1, CD180, PRSS57, KERA, KMO, PGF, ADCYAP1, FRMD7, MFNG, COL4A4, PRTN3, S100A8, SEMA7A, LY96, CABP1, SCUBE1, LRRC4C, CPA5, VCAM1, BDNF, RARRES1, LY86, SSC5D, SELE, IL2, BMP5, GH1, BMP3, IL6, FAM151A, FGF18, HPSE, FGF10, ITGAM, TNFAIP6, LRRK2, FASLG, CPZ, CXCL3, HAPLN3, CXCL5, TNFSF13B, SPN, ADAMTS4, INSL3, TSHB, CR2, CR1, EGFL6, MMP3, NGF, TMIGD2, ACTBL2, IFNG, CHI3L2, XCL2, LTA, BPI, PKHD1L1, LTB, MS4A1, IL9R, KRT81, SEMA3A, FGL2, RETN, KRT86, PTHLH, CLEC3A, CCL8, CCL7, LINGO2, CMA1, CMTM5, S100B, POMC, PI16 |
| GOTERM_CC_DIRECT | GO:0043235~receptor complex | 46 | 4.42E-14 | 2.71E-12 | ADCYAP1R1, CSF3R, FLT3, IL23R, MTTP, EBI3, LY96, GPR84, PTH1R, CSF2RA, SLITRK5, IL12B, OLR1, ITGB7, IL12RB1, IL13RA2, EPHB1, NTRK1, PDGFRA, NTRK2, CR2, FCRL5, MUSK, EPHA8, NTRK3, GPBAR1, TRPV3, GFRA1, GPR37L1, SCIMP, GFRA2, LRP1B, TLR1, P2RX6, CD200R1, ADRB3, CR1L, P2RX2, KIT, TLR10, TLR7, KLRD1, TLR6, MET, CRLF2, TLR2 |
| GOTERM_CC_DIRECT | GO:0070821~tertiary granule membrane | 19 | 1.26E-07 | 6.86E-06 | SNAP25, ITGAM, SIGLEC14, CLEC12A, CD300A, PTAFR, CYBB, MCEMP1, GPR84, LILRB2, FPR2, FCAR, CLEC4C, CLEC4D, CLEC5A, OLR1, CD33, LAIR1, SIGLEC5 |
| GOTERM_CC_DIRECT | GO:0035579~specific granule membrane | 21 | 1.98E-07 | 9.72E-06 | SNAP25, ITGAM, CLEC12A, HVCN1, CYBB, MCEMP1, GPR84, FPR2, ITGAL, FCAR, CEACAM3, BST1, RAB44, CLEC4D, RAB37, SLCO4C1, CLEC5A, C3AR1, OLR1, CD33, LAIR1 |
| GOTERM_CC_DIRECT | GO:0031225~anchored component of membrane | 21 | 5.38E-06 | 2.40E-04 | SEMA7A, NEGR1, TREH, GFRA1, EFNA5, GFRA2, RGMA, BST1, OTOA, FCGR3B, VNN1, XPNPEP2, MMP17, DPEP2, CD48, NCAM1, LY6G6D, ENPP6, PRND, TDGF1, DPEP3 |
| GOTERM_CC_DIRECT | GO:0044853~plasma membrane raft | 11 | 1.51E-05 | 6.15E-04 | CR1, SELPLG, ITGAM, CDH2, MAL, HAS2, LCP2, PRTN3, MS4A1, SKAP1, MS4A4A |
| GOTERM_CC_DIRECT | GO:0001772~immunological synapse | 11 | 1.68E-04 | 6.32E-03 | CD6, GZMA, CRTAM, CD28, PRKCQ, CD37, CD3E, SCIMP, CARD11, SKAP1, HAVCR2 |
| GOTERM_CC_DIRECT | GO:0043005~neuron projection | 39 | 3.35E-04 | 1.17E-02 | PCSK2, CHRNA1, CX3CR1, GPM6A, SNAP25, ADCYAP1R1, KLHL14, CPNE5, LRRK2, CHRNA6, KCNA2, RASGRF1, STMN2, RASGRP2, KIF17, PTGS1, CALB2, RNF112, ATCAY, FLRT2, CDH2, SV2A, BAALC, NCAM1, EPHB1, BCL11B, NPY5R, EPHA8, ANK2, SSTR2, SSTR3, RGMA, FOSL1, SLC4A8, ADCYAP1, CPEB1, FRMD7, CHRFAM7A, CAMK1G |
| GOTERM_CC_DIRECT | GO:0030425~dendrite | 46 | 3.72E-04 | 1.22E-02 | PCSK2, GRIA2, NCF1, SEMA3A, LRRK2, KCNA2, ELAVL4, HTR2B, HTR2A, HTR4, SLC8A1, CALB2, DRP2, TRIM9, CRHBP, HRH1, SAMD14, HTR7, ATCAY, CNR2, HRH2, SV2A, ADORA1, KIF21B, EPHB1, CCR2, KCNH1, KIRREL3, NTRK1, NTRK2, RCVRN, HTR1E, KCNIP1, NEGR1, HTR1F, BDNF, CYBB, TACR1, NGF, CPT1C, PRSS12, SLC4A8, GAP43, CPEB1, PNOC, FAT3 |
| GOTERM_CC_DIRECT | GO:0030667~secretory granule membrane | 16 | 6.98E-04 | 2.14E-02 | CR1, SIGLEC9, HVCN1, PTAFR, FPR1, SIRPB1, CLEC4C, FCGR2A, FCGR3B, PTPRC, SELL, CXCR1, CXCR2, NCKAP1L, SIGLEC5, TLR2 |
| GOTERM_CC_DIRECT | GO:0045121~membrane raft | 28 | 1.49E-03 | 4.29E-02 | KCNE1, ABCB4, LRRK2, KCNA3, MLC1, FAIM2, TNF, CD1A, CD79A, CNR1, CD19, INPP5D, OLR1, BAALC, EPHB1, ANK2, SELE, TLR1, PTPRC, BTK, MAL, CD48, CD226, TLR6, HPSE, TDGF1, CARD11, TLR2 |
| GOTERM_MF_DIRECT | GO:0004888~transmembrane signaling receptor activity | 57 | 1.48E-24 | 1.58E-21 | CD3G, CD3E, SPN, FCRLA, MILR1, FCGR3B, MRC1, FCER1A, TNFRSF8, FCRL1, FCRL2, CR2, FCRL5, CD300A, DCC, FCRL6, FCRL3, TLR1, CD300E, TLR8, TLR10, TLR7, TLR6, CD300C, TLR2, CHRNA1, IGSF6, KLRB1, CHRNA6, LILRA1, CD79B, CD79A, CD300LB, IL21R, CD300LF, FCGR1A, SLAMF1, CD72, KLRC4, SELE, LYVE1, LILRB5, TREML1, P2RX6, MARCO, FCGR2A, EVI2A, GPR182, CD27, CHRFAM7A, KLRD1, CD247, CD69, FCGR2B, SIGLEC8, LGR6, FCGR2C |
| GOTERM_MF_DIRECT | GO:0030246~carbohydrate binding | 50 | 9.73E-18 | 5.18E-15 | FCN1, SIGLEC9, CLEC10A, LY75, CLEC18B, CLEC9A, ASGR2, LGALS2, CLEC5A, OLR1, SVEP1, GAL3ST3, CD33, GALNT9, GALNT8, KRT1, FCER2, CLEC4C, CLEC4D, LGALS12, CHI3L2, CLEC4E, CLEC4G, CHODL, KLRB1, CLEC12A, CLECL1, CLEC3A, CLEC7A, KLRG1, SIGLEC14, CD72, SIGLEC11, SIGLEC10, PRG2, CLEC17A, SELE, IL2, CLEC2B, SELL, CD209, CNTN2, KLRD1, CD69, SIGLEC1, SIGLEC8, SIGLEC7, CD22, SIGLEC6, SIGLEC5 |
| GOTERM_MF_DIRECT | GO:0038023~signaling receptor activity | 53 | 2.15E-17 | 7.63E-15 | CD86, CSF3R, TNFRSF13B, LY75, TNFRSF13C, GRIK2, TREM1, MRC1, ITGB7, CCR6, CD33, CMKLR1, CD300A, BTN1A1, KRT1, IZUMO1R, GFRA1, GFRA2, TLR1, CD200R1, TLR8, TLR10, CD48, TLR6, PKHD1L1, TLR2, GRIA2, ADCYAP1R1, CSF2RB, FPR2, LILRA2, CSF2RA, P2RY6, GPA33, CXCR3, P2RY2, SLAMF8, BTLA, TNFRSF17, GRIA3, STRA6, SLAMF1, KLRG1, IL10RA, TNFRSF9, LYVE1, CD5, TREML2, FOLR3, FOLR2, SIGLEC7, CD244, IL18R1 |
| GOTERM_MF_DIRECT | GO:0016493~C-C chemokine receptor activity | 18 | 1.07E-16 | 2.86E-14 | CCR1, CX3CR1, XCR1, CXCR6, CXCR1, CXCR3, CCRL2, CXCR2, ACKR4, CCR9, CCR8, CCR7, CCR6, CCR5, CCR4, CCR10, CCR3, CCR2 |
| GOTERM_MF_DIRECT | GO:0019957~C-C chemokine binding | 18 | 3.47E-16 | 7.38E-14 | CCR1, CX3CR1, XCR1, CXCR6, CXCR1, CXCR3, CCRL2, CXCR2, ACKR4, CCR9, CCR8, CCR7, CCR6, CCR5, CCR4, CCR10, CCR3, CCR2 |
| GOTERM_MF_DIRECT | GO:0004950~chemokine receptor activity | 15 | 6.44E-14 | 1.14E-11 | CCR1, CX3CR1, XCR1, CXCR1, CXCR3, CCRL2, ACKR4, CCR9, CCR8, CCR6, CCR5, CCR4, CCR3, CCR2, CMKLR1 |
| GOTERM_MF_DIRECT | GO:0008009~chemokine activity | 20 | 2.73E-11 | 4.16E-09 | CCL14, CCL24, CCL13, CCL23, CCL11, CCL22, CCL21, PPBP, CXCL13, CXCL3, CXCL5, CCL8, CCL7, XCL2, CCL17, PF4V1, CCL16, CCL26, PF4, CCL15 |
| GOTERM_MF_DIRECT | GO:0005125~cytokine activity | 38 | 1.70E-10 | 2.26E-08 | CSF3, IL24, EBI3, FASLG, TNF, AREG, TNFSF13B, IL12B, TNFSF11, VSTM1, LEFTY2, IL10, TNFSF18, TNFSF14, TSLP, CD70, IL15, IL34, CMTM5, IL13, LIF, OSM, IL18, WNT9B, FLT3LG, IL16, GDF3, IL2, BMP5, BMP3, IL1A, IL6, CD40LG, IFNG, IL1B, LTA, TNFSF8, LTB |
| GOTERM_MF_DIRECT | GO:0048020~CCR chemokine receptor binding | 15 | 2.43E-10 | 2.88E-08 | CCL14, CCL24, CCL13, CCL23, CCL11, CCL22, CCL21, CCL8, CCL7, CCRL2, XCL2, CCL17, CCL16, CCL26, CCL15 |
| GOTERM_MF_DIRECT | GO:0033691~sialic acid binding | 13 | 1.52E-09 | 1.61E-07 | FCN1, SIGLEC14, SIGLEC9, SIGLEC11, SIGLEC10, SELE, ST8SIA4, SIGLEC8, SIGLEC7, CD22, CD33, SIGLEC6, SIGLEC5 |
| GOTERM_MF_DIRECT | GO:0045028~G-protein coupled purinergic nucleotide receptor activity | 9 | 1.62E-07 | 1.57E-05 | P2RY12, P2RY8, P2RY13, P2RY10, P2RY6, P2RY2, P2RY14, GPR171, PTAFR |
| GOTERM_MF_DIRECT | GO:0071723~lipopeptide binding | 8 | 2.83E-07 | 2.32E-05 | TLR1, CD1E, CD1D, TLR6, CD1C, CD1B, CD1A, TLR2 |
| GOTERM_MF_DIRECT | GO:0032396~inhibitory MHC class I receptor activity | 8 | 2.83E-07 | 2.32E-05 | LILRA6, LILRB1, LILRB2, LILRA1, LILRA2, LILRA4, LILRB5, LILRA5 |
| GOTERM_MF_DIRECT | GO:0004896~cytokine receptor activity | 16 | 6.87E-07 | 5.23E-05 | CSF3R, FLT3, IL10RA, IL23R, EBI3, CSF2RB, CSF2RA, IL1RL1, IL2RB, IL21R, IL12B, IL12RB1, IL13RA2, IL7R, IL9R, CRLF2 |
| GOTERM_MF_DIRECT | GO:0008083~growth factor activity | 28 | 2.09E-06 | 1.49E-04 | CSF3, AREG, FGF7, FGF9, IL12B, LEFTY2, IL10, BDNF, IL34, HGF, MACC1, LIF, OSM, PPBP, GDF3, NGF, DKK1, IL2, PGF, BMP5, EREG, GH1, BMP3, IL6, FGF14, FGF18, TDGF1, FGF10 |
| GOTERM_MF_DIRECT | GO:0015026~coreceptor activity | 13 | 3.23E-06 | 2.04E-04 | CD86, FCRL1, GPR15, ITGA4, CD80, LY96, CXCR6, LILRA4, RGMA, TMIGD2, CD28, CCR8, CCR5 |
| GOTERM_MF_DIRECT | GO:0005102~receptor binding | 49 | 3.25E-06 | 2.04E-04 | FCN1, ICAM3, SLA, FASLG, TNFSF13B, PLXNC1, RSPO1, INSL3, HGF, BTN1A1, WNT9B, FLT3LG, IZUMO1R, GFRA1, NGF, SSTR3, FGR, HCK, ADCYAP1, LTA, PRTN3, PGR, LTB, TLR6, BLK, CCL13, SELPLG, FGL2, VTCN1, LILRA4, SRPX2, TESPA1, TIGIT, CCL17, CCL15, TNFSF18, CD72, TNFSF14, CD70, CRTAM, LIF, P2RX7, POMC, BMP3, PTPRC, TNFSF8, TDGF1, HAMP, CD22 |
| GOTERM_MF_DIRECT | GO:0008201~heparin binding | 28 | 4.19E-06 | 2.48E-04 | CFH, COL13A1, CXCL13, MPO, SERPINA5, FGF7, CCL8, LIPC, FGF9, CCL7, ABI3BP, CTSG, RSPO1, ELANE, PF4V1, CCL15, CCL23, PLA2G2D, PRG2, PRSS57, PGF, NELL1, FGF14, PTPRC, SELL, LGR6, PF4, FGF10 |
| GOTERM_MF_DIRECT | GO:0001618~virus receptor activity | 17 | 9.14E-06 | 5.12E-04 | SERPINB3, CD86, CR2, CR1, SELPLG, GPR15, CD80, HTR2A, CD209, MRC1, CLEC5A, NCAM1, ITGB7, CCR5, TNFRSF4, CLEC4G, SLAMF1 |
| GOTERM_MF_DIRECT | GO:0004875~complement receptor activity | 7 | 1.65E-05 | 8.77E-04 | CR2, C5AR2, FPR1, C3AR1, FPR3, FPR2, CMKLR1 |
| GOTERM_MF_DIRECT | GO:0050135~NAD(P)+ nucleosidase activity | 8 | 1.96E-05 | 9.48E-04 | TLR1, IL1RL1, BST1, IL18RAP, TLR10, TLR6, IL18R1, TLR2 |
| GOTERM_MF_DIRECT | GO:0061809~NAD+ nucleotidase, cyclic ADP-ribose generating | 8 | 1.96E-05 | 9.48E-04 | TLR1, IL1RL1, BST1, IL18RAP, TLR10, TLR6, IL18R1, TLR2 |
| GOTERM_MF_DIRECT | GO:0038187~pattern recognition receptor activity | 8 | 3.16E-05 | 1.46E-03 | FCN1, MARCO, CLEC4D, CLEC7A, TLR8, TLR7, CLEC4E, TLR2 |
| GOTERM_MF_DIRECT | GO:0030884~exogenous lipid antigen binding | 5 | 6.16E-05 | 2.63E-03 | CD1E, CD1D, CD1C, CD1B, CD1A |
| GOTERM_MF_DIRECT | GO:0030883~endogenous lipid antigen binding | 5 | 6.16E-05 | 2.63E-03 | CD1E, CD1D, CD1C, CD1B, CD1A |
| GOTERM_MF_DIRECT | GO:0005164~tumor necrosis factor receptor binding | 10 | 7.85E-05 | 3.21E-03 | CD40LG, TNFSF14, CD70, LTA, TNFSF11, TNFSF8, FASLG, LTB, TNF, TNFSF13B |
| GOTERM_MF_DIRECT | GO:0019955~cytokine binding | 11 | 1.82E-04 | 7.19E-03 | CSF3R, IL23R, TNFRSF9, EBI3, KIT, IL12B, IL12RB1, IL13RA2, CSF2RA, ELANE, CRLF2 |
| GOTERM_MF_DIRECT | GO:0004222~metalloendopeptidase activity | 19 | 2.33E-04 | 8.86E-03 | ADAMDEC1, PAPLN, MMP3, ADAMTS12, MMP12, ADAMTS4, ADAM19, ADAMTS14, MMP16, ADAMTSL4, PAPPA, MMP23B, MMP17, ADAM12, ADAM23, CLCA2, MMP19, ADAMTS6, TRABD2B |
| GOTERM_MF_DIRECT | GO:0005244~voltage-gated ion channel activity | 12 | 5.01E-04 | 1.84E-02 | CACNG6, CLIC6, TMEM37, CALHM1, KCNIP1, KCNA3, CYBB, SCN5A, CLIC2, NALCN, SCN3A, SCN2B |
| GOTERM_MF_DIRECT | GO:0005030~neurotrophin receptor activity | 4 | 8.26E-04 | 2.93E-02 | NTRK1, NTRK2, NTRK3, GFRA1 |
| GOTERM_MF_DIRECT | GO:0045236~CXCR chemokine receptor binding | 6 | 1.40E-03 | 4.81E-02 | PPBP, CXCL13, CXCL3, CXCL5, PF4V1, PF4 |
| KEGG_PATHWAY | hsa04060:Cytokine-cytokine receptor interaction | 95 | 9.25E-39 | 2.30E-36 | CSF3, CSF3R, TNFRSF13B, IL23R, IL24, FASLG, TNFRSF13C, CXCL13, CXCL3, TNF, CXCL5, TNFSF13B, IL18RAP, CCR9, IL12B, TNFSF11, TNFRSF8, CCR8, CCR7, CCR6, IL13RA2, CCR5, CCR4, TNFRSF4, PF4V1, CCR3, CCR2, IL10, IL15, IL1R2, IL13, IL18, IL16, NGF, IL1A, IFNG, IL1B, XCL2, LTA, LTB, IL9R, CCL14, CX3CR1, CCL13, CCL11, EBI3, CSF2RB, CXCR6, CSF2RA, IL1RL1, CCL8, CCL7, CXCR1, CXCR3, CXCR2, IL21R, TNFRSF17, IL12RB1, CCL17, CCR10, CCL16, CCL15, TNFSF18, CCR1, CCL24, CCL23, XCR1, CCL22, TNFSF14, CCL21, TSLP, CD70, IL34, IL10RA, TNFRSF9, LIF, OSM, PPBP, GDF3, IL2, BMP5, GH1, BMP3, IL6, CD40LG, IL2RA, IL2RB, ACKR4, CD27, TNFSF8, IL7R, IL18R1, PF4, CCL26, CRLF2 |
| KEGG_PATHWAY | hsa04062:Chemokine signaling pathway | 54 | 7.36E-19 | 6.11E-17 | ITK, NCF1, PIK3CD, CXCL13, CXCL3, PIK3CG, CXCL5, GNGT2, CCR9, CCR8, CCR7, CCR6, CCR5, JAK3, CCR4, PF4V1, CCR3, CCR2, PRKCB, VAV1, FGR, HCK, XCL2, DOCK2, CCL14, CX3CR1, CCL13, CCL11, CXCR6, RASGRP2, ADCY7, PIK3R6, PIK3R5, GNG2, CCL8, CCL7, CXCR1, CXCR3, CXCR2, PLCG2, GNG8, CCL17, CCR10, CCL16, CCL15, CCR1, CCL24, CCL23, XCR1, CCL22, CCL21, PPBP, PF4, CCL26 |
| KEGG_PATHWAY | hsa04640:Hematopoietic cell lineage | 36 | 1.64E-16 | 1.02E-14 | CSF3, CSF3R, ITGAM, FLT3, CD1E, CD3G, CD1D, CD1C, CD3E, CD1B, TNF, CSF2RA, CD1A, CD19, CD37, FCGR1A, HLA-DOB, CD33, CR2, CR1, ITGA4, IL1R2, FLT3LG, GP5, FCER2, IL1A, IL6, CD5, CR1L, IL1B, IL2RA, KIT, IL7R, MS4A1, CD22, IL9R |
| KEGG_PATHWAY | hsa04080:Neuroactive ligand-receptor interaction | 64 | 9.30E-12 | 4.63E-10 | PMCH, HTR2B, HTR2A, TRH, GRIK2, PTH1R, HTR4, CYSLTR2, HTR7, ADORA3, ADORA1, C3AR1, BDKRB2, CTSG, INSL3, TSHB, PTGDR, PTGIR, NPY5R, TACR1, SSTR2, TAAR1, SSTR3, ADCYAP1, ADRB3, CHRNA1, GRIA2, ADCYAP1R1, CHRNA6, PTAFR, FPR1, FPR3, LPAR4, FPR2, P2RY8, P2RY6, HRH1, CALCR, CNR2, HRH2, CNR1, P2RY2, KISS1R, DRD1, GPHA2, S1PR4, GRIA3, DRD5, P2RY13, P2RY10, HTR1E, HTR1F, GZMA, P2RY14, NMBR, HCRTR1, P2RX7, POMC, GH1, P2RX6, MAS1, P2RX2, PNOC, F2RL2 |
| KEGG_PATHWAY | hsa04514:Cell adhesion molecules | 34 | 2.29E-09 | 9.49E-08 | CD86, SELPLG, ITGAM, CD80, ICAM3, VTCN1, ITGAL, SPN, CDH2, CTLA4, NCAM1, ITGB7, LRRC4C, ICOS, TIGIT, HLA-DOB, CADM3, VCAM1, ITGA4, NEGR1, PDCD1LG2, SELE, CLDN11, PTPRC, CD40LG, SELL, CD6, CD28, CLDN18, CNTN2, CD226, PDCD1, SIGLEC1, CD22 |
| KEGG_PATHWAY | hsa04662:B cell receptor signaling pathway | 24 | 5.53E-09 | 1.72E-07 | LILRA6, CR2, CD72, PRKCB, PIK3CD, DAPP1, LILRB1, LILRB2, LILRA1, LILRA2, LILRB4, LILRB5, VAV1, LILRA4, LILRA5, CD79B, CD79A, CD19, INPP5D, BTK, PLCG2, FCGR2B, CD22, CARD11 |
| KEGG_PATHWAY | hsa04630:JAK-STAT signaling pathway | 30 | 2.56E-06 | 4.91E-05 | CSF3, CSF3R, IL23R, IL24, PIK3CD, CSF2RB, CSF2RA, STAT4, IL21R, IL12B, IL12RB1, IL13RA2, JAK3, IL10, PDGFRA, TSLP, IL15, IL10RA, IL13, LIF, OSM, IL2, GH1, IL6, IFNG, IL2RA, IL2RB, IL7R, IL9R, CRLF2 |
| KEGG_PATHWAY | hsa04020:Calcium signaling pathway | 36 | 2.86E-05 | 3.73E-04 | PDE1C, PDE1B, PTAFR, HTR2B, HTR2A, CACNA1F, HTR4, ADCY7, SLC8A1, HRH1, FGF7, CYSLTR2, HTR7, FGF9, HRH2, PLCG2, BDKRB2, DRD1, DRD5, NTRK1, PDGFRA, NTRK2, PRKCB, HGF, NTRK3, TACR1, NGF, P2RX7, P2RX6, ADRB3, P2RX2, CAMK4, FGF18, MET, CAMK1G, FGF10 |
| KEGG_PATHWAY | hsa04660:T cell receptor signaling pathway | 21 | 3.18E-05 | 3.73E-04 | IL10, ITK, PIK3CD, CD3G, CD3E, RASGRP1, TNF, IL2, VAV1, PTPRC, CD40LG, IFNG, GRAP2, CD28, CTLA4, LCP2, PRKCQ, PDCD1, CD247, ICOS, CARD11 |
| KEGG_PATHWAY | hsa04151:PI3K-Akt signaling pathway | 46 | 7.25E-05 | 7.52E-04 | CSF3, CSF3R, FLT3, PIK3CD, FASLG, LPAR4, EFNA5, AREG, PIK3R6, PIK3CG, PIK3R5, FGF7, TCL1A, GNGT2, GNG2, FGF9, TNN, CD19, GNG8, ITGB7, JAK3, NTRK1, PDGFRA, NTRK2, ITGA4, BDNF, HGF, OSM, FLT3LG, NGF, IL2, PGF, EREG, GH1, IL6, LPAR5, PPP2R2B, COL4A4, IL2RA, KIT, IL2RB, FGF18, IL7R, MET, FGF10, TLR2 |
| KEGG_PATHWAY | hsa04064:NF-kappa B signaling pathway | 20 | 1.03E-04 | 1.03E-03 | CCL13, VCAM1, TNFSF14, BCL2A1, CCL21, PRKCB, LY96, TNFRSF13C, CXCL3, TNF, TNFSF13B, CD40LG, IL1B, LTA, BTK, PLCG2, TNFSF11, PRKCQ, LTB, CARD11 |
| KEGG_PATHWAY | hsa04014:Ras signaling pathway | 32 | 4.07E-04 | 3.62E-03 | FLT3, RASGRF1, PIK3CD, FASLG, EFNA5, RASGRP2, RASGRP1, RASGRP4, FGF7, HTR7, GNGT2, GNG2, FGF9, PLCG2, GNG8, NTRK1, PDGFRA, NTRK2, PLA2G2D, RASA4B, KSR1, PRKCB, PLA2G2C, BDNF, HGF, FLT3LG, NGF, PGF, KIT, FGF18, MET, FGF10 |
| KEGG_PATHWAY | hsa04658:Th1 and Th2 cell differentiation | 17 | 6.35E-04 | 5.45E-03 | IL13, CD3G, CD3E, RUNX3, IL2, DLL3, IFNG, IL2RA, TBX21, IL2RB, STAT4, IL12B, PRKCQ, CD247, IL12RB1, JAK3, HLA-DOB |
| KEGG_PATHWAY | hsa04650:Natural killer cell mediated cytotoxicity | 20 | 1.27E-03 | 1.02E-02 | PRKCB, SH2D1B, PRF1, PIK3CD, FASLG, ITGAL, TNF, VAV1, NCR1, NCR3, FCGR3B, RAET1E, IFNG, PLCG2, LCP2, CD48, KLRD1, CD247, CD244, MICB |
| KEGG_PATHWAY | hsa04659:Th17 cell differentiation | 18 | 1.39E-03 | 1.08E-02 | IL23R, EBI3, CD3G, CD3E, IL2, IL6, IFNG, IRF4, IL1B, IL2RA, TBX21, IL2RB, IL21R, PRKCQ, CD247, IL12RB1, JAK3, HLA-DOB |
| KEGG_PATHWAY | hsa04145:Phagosome | 22 | 2.23E-03 | 1.68E-02 | MSR1, ITGAM, TUBAL3, NCF1, NCF2, NCF4, CYBB, MPO, FCAR, MARCO, FCGR2A, FCGR3B, CLEC7A, CD209, MRC1, OLR1, TLR6, FCGR1A, FCGR2B, HLA-DOB, FCGR2C, TLR2 |
| KEGG_PATHWAY | hsa04625:C-type lectin receptor signaling pathway | 17 | 2.43E-03 | 1.78E-02 | IL10, CCL22, KSR1, PIK3CD, TNF, IL2, IL6, CLEC4D, CLEC7A, CD209, IL1B, CASP1, PLCG2, IL12B, NLRP3, CLEC4E, CCL17 |
| KEGG_PATHWAY | hsa05200:Pathways in cancer | 54 | 5.89E-03 | 3.93E-02 | CSF3R, FLT3, HHIP, IL23R, PIK3CD, FASLG, GLI1, GLI2, FGF7, GNGT2, FGF9, BDKRB2, IL12B, JAK3, PDGFRA, IL15, PRKCB, DCC, HGF, IL13, WNT9B, FLT3LG, PGF, CCNA1, IFNG, COL4A4, KIT, MET, CSF2RB, LPAR4, RASGRP2, RASGRP1, CSF2RA, ADCY7, RASGRP4, DLL3, GNG2, PLCG2, STAT4, GNG8, IL12RB1, RUNX1T1, NTRK1, ESR1, ESR2, IL2, IL6, LPAR5, IL2RA, IL2RB, FGF18, IL7R, GSTM5, FGF10 |
| KEGG_PATHWAY | hsa05134:Legionellosis | 11 | 6.00E-03 | 3.93E-02 | IL6, CR1, ITGAM, CR1L, IL1B, CASP1, IL18, IL12B, CXCL3, TNF, TLR2 |
