# Supplementary material for: Identifying the tumor immune microenvironment-associated prognostic genes for prostate cancer
Source: Discov Oncol. 2024 Feb 20;15:42. doi: 10.1007/s12672-023-00856-3 (PMC10879074; doi:10.1007/s12672-023-00856-3)
Supplement: Supplementary file 3 — Supplementary material 3 [file 12672_2023_856_MOESM3_ESM.docx]

Supplementary Table 1 The estimate, immune, tromal scores in PRAD tumors

Ensembl_ID Symbol TCGA-CH-5761-11 TCGA-CH-5767-11 TCGA-CH-5768-11 TCGA-CH-5769-11 TCGA-EJ-7115-11 TCGA-EJ-7123-11 TCGA-EJ-7125-11 TCGA-EJ-7314-11 TCGA-EJ-7315-11 TCGA-EJ-7317-11 TCGA-EJ-7321-11 TCGA-EJ-7327-11 TCGA-EJ-7328-11 TCGA-EJ-7330-11 TCGA-EJ-7331-11 TCGA-EJ-7781-11 TCGA-EJ-7782-11 TCGA-EJ-7783-11 TCGA-EJ-7784-11 TCGA-EJ-7785-11 TCGA-EJ-7786-11 TCGA-EJ-7789-11 TCGA-EJ-7792-11 TCGA-EJ-7793-11 TCGA-EJ-7794-11 TCGA-EJ-7797-11 TCGA-EJ-A8FO-11 TCGA-G9-6333-11 TCGA-G9-6342-11 TCGA-G9-6348-11 TCGA-G9-6351-11 TCGA-G9-6356-11 TCGA-G9-6362-11 TCGA-G9-6363-11 TCGA-G9-6365-11 TCGA-G9-6384-11 TCGA-G9-6496-11 TCGA-G9-6499-11 TCGA-HC-7211-11 TCGA-HC-7737-11 TCGA-HC-7738-11 TCGA-HC-7740-11 TCGA-HC-7742-11 TCGA-HC-7745-11 TCGA-HC-7747-11 TCGA-HC-7752-11 TCGA-HC-7819-11 TCGA-HC-8258-11 TCGA-HC-8259-11 TCGA-HC-8260-11 TCGA-HC-8262-11 TCGA-J4-A83J-11 TCGA-2A-A8VL-01 TCGA-2A-A8VO-01 TCGA-2A-A8VT-01 TCGA-2A-A8VV-01 TCGA-2A-A8VX-01 TCGA-2A-A8W1-01 TCGA-2A-A8W3-01 TCGA-2A-AAYF-01 TCGA-2A-AAYO-01 TCGA-2A-AAYU-01 TCGA-4L-AA1F-01 TCGA-CH-5737-01 TCGA-CH-5738-01 TCGA-CH-5739-01 TCGA-CH-5740-01 TCGA-CH-5741-01 TCGA-CH-5743-01 TCGA-CH-5744-01 TCGA-CH-5745-01 TCGA-CH-5746-01 TCGA-CH-5748-01 TCGA-CH-5750-01 TCGA-CH-5751-01 TCGA-CH-5752-01 TCGA-CH-5753-01 TCGA-CH-5754-01 TCGA-CH-5761-01 TCGA-CH-5762-01 TCGA-CH-5763-01 TCGA-CH-5764-01 TCGA-CH-5765-01 TCGA-CH-5766-01 TCGA-CH-5767-01 TCGA-CH-5768-01 TCGA-CH-5769-01 TCGA-CH-5771-01 TCGA-CH-5772-01 TCGA-CH-5788-01 TCGA-CH-5789-01 TCGA-CH-5790-01 TCGA-CH-5791-01 TCGA-CH-5792-01 TCGA-CH-5794-01 TCGA-EJ-5494-01 TCGA-EJ-5495-01 TCGA-EJ-5496-01 TCGA-EJ-5497-01 TCGA-EJ-5498-01 TCGA-EJ-5499-01 TCGA-EJ-5501-01 TCGA-EJ-5502-01 TCGA-EJ-5503-01 TCGA-EJ-5504-01 TCGA-EJ-5505-01 TCGA-EJ-5506-01 TCGA-EJ-5507-01 TCGA-EJ-5508-01 TCGA-EJ-5509-01 TCGA-EJ-5510-01 TCGA-EJ-5511-01 TCGA-EJ-5512-01 TCGA-EJ-5514-01 TCGA-EJ-5515-01 TCGA-EJ-5516-01 TCGA-EJ-5517-01 TCGA-EJ-5518-01 TCGA-EJ-5519-01 TCGA-EJ-5521-01 TCGA-EJ-5522-01 TCGA-EJ-5524-01 TCGA-EJ-5525-01 TCGA-EJ-5526-01 TCGA-EJ-5527-01 TCGA-EJ-5530-01 TCGA-EJ-5531-01 TCGA-EJ-5532-01 TCGA-EJ-5542-01 TCGA-EJ-7115-01 TCGA-EJ-7123-01 TCGA-EJ-7125-01 TCGA-EJ-7218-01 TCGA-EJ-7312-01 TCGA-EJ-7314-01 TCGA-EJ-7315-01 TCGA-EJ-7317-01 TCGA-EJ-7318-01 TCGA-EJ-7321-01 TCGA-EJ-7325-01 TCGA-EJ-7327-01 TCGA-EJ-7328-01 TCGA-EJ-7330-01 TCGA-EJ-7331-01 TCGA-EJ-7781-01 TCGA-EJ-7782-01 TCGA-EJ-7783-01 TCGA-EJ-7784-01 TCGA-EJ-7785-01 TCGA-EJ-7786-01 TCGA-EJ-7788-01 TCGA-EJ-7789-01 TCGA-EJ-7791-01 TCGA-EJ-7792-01 TCGA-EJ-7793-01 TCGA-EJ-7794-01 TCGA-EJ-7797-01 TCGA-EJ-8468-01 TCGA-EJ-8469-01 TCGA-EJ-8470-01 TCGA-EJ-8472-01 TCGA-EJ-8474-01 TCGA-EJ-A46B-01 TCGA-EJ-A46D-01 TCGA-EJ-A46E-01 TCGA-EJ-A46F-01 TCGA-EJ-A46G-01 TCGA-EJ-A46H-01 TCGA-EJ-A46I-01 TCGA-EJ-A65B-01 TCGA-EJ-A65D-01 TCGA-EJ-A65E-01 TCGA-EJ-A65F-01 TCGA-EJ-A65G-01 TCGA-EJ-A65J-01 TCGA-EJ-A65M-01 TCGA-EJ-A6RA-01 TCGA-EJ-A6RC-01 TCGA-EJ-A7NF-01 TCGA-EJ-A7NG-01 TCGA-EJ-A7NH-01 TCGA-EJ-A7NJ-01 TCGA-EJ-A7NK-01 TCGA-EJ-A7NM-01 TCGA-EJ-A7NN-01 TCGA-EJ-A8FN-01 TCGA-EJ-A8FO-01 TCGA-EJ-A8FP-01 TCGA-EJ-A8FS-01 TCGA-EJ-A8FU-01 TCGA-EJ-AB20-01 TCGA-EJ-AB27-01 TCGA-FC-7708-01 TCGA-FC-7961-01 TCGA-FC-A4JI-01 TCGA-FC-A5OB-01 TCGA-FC-A66V-01 TCGA-FC-A6HD-01 TCGA-FC-A8O0-01 TCGA-G9-6329-01 TCGA-G9-6332-01 TCGA-G9-6333-01 TCGA-G9-6336-01 TCGA-G9-6338-01 TCGA-G9-6339-01 TCGA-G9-6342-01 TCGA-G9-6343-01 TCGA-G9-6347-01 TCGA-G9-6348-01 TCGA-G9-6351-01 TCGA-G9-6353-01 TCGA-G9-6354-01 TCGA-G9-6356-01 TCGA-G9-6361-01 TCGA-G9-6362-01 TCGA-G9-6363-01 TCGA-G9-6364-01 TCGA-G9-6365-01 TCGA-G9-6366-01 TCGA-G9-6367-01 TCGA-G9-6369-01 TCGA-G9-6370-01 TCGA-G9-6371-01 TCGA-G9-6373-01 TCGA-G9-6377-01 TCGA-G9-6378-01 TCGA-G9-6379-01 TCGA-G9-6384-01 TCGA-G9-6385-01 TCGA-G9-6494-01 TCGA-G9-6496-01 TCGA-G9-6498-01 TCGA-G9-6499-01 TCGA-G9-7509-01 TCGA-G9-7510-01 TCGA-G9-7519-01 TCGA-G9-7521-01 TCGA-G9-7522-01 TCGA-G9-7523-01 TCGA-G9-7525-01 TCGA-G9-A9S0-01 TCGA-G9-A9S4-01 TCGA-G9-A9S7-01 TCGA-H9-7775-01 TCGA-H9-A6BX-01 TCGA-H9-A6BY-01 TCGA-HC-7075-01 TCGA-HC-7077-01 TCGA-HC-7078-01 TCGA-HC-7079-01 TCGA-HC-7080-01 TCGA-HC-7081-01 TCGA-HC-7209-01 TCGA-HC-7210-01 TCGA-HC-7211-01 TCGA-HC-7212-01 TCGA-HC-7213-01 TCGA-HC-7230-01 TCGA-HC-7231-01 TCGA-HC-7232-01 TCGA-HC-7233-01 TCGA-HC-7736-01 TCGA-HC-7737-01 TCGA-HC-7738-01 TCGA-HC-7740-01 TCGA-HC-7742-01 TCGA-HC-7744-01 TCGA-HC-7745-01 TCGA-HC-7747-01 TCGA-HC-7748-01 TCGA-HC-7749-01 TCGA-HC-7750-01 TCGA-HC-7752-01 TCGA-HC-7817-01 TCGA-HC-7818-01 TCGA-HC-7819-01 TCGA-HC-7820-01 TCGA-HC-7821-01 TCGA-HC-8213-01 TCGA-HC-8216-01 TCGA-HC-8256-01 TCGA-HC-8257-01 TCGA-HC-8258-01 TCGA-HC-8259-01 TCGA-HC-8260-01 TCGA-HC-8261-01 TCGA-HC-8262-01 TCGA-HC-8264-01 TCGA-HC-8265-01 TCGA-HC-8266-01 TCGA-HC-A48F-01 TCGA-HC-A4ZV-01 TCGA-HC-A631-01 TCGA-HC-A632-01 TCGA-HC-A6AL-01 TCGA-HC-A6AN-01 TCGA-HC-A6AO-01 TCGA-HC-A6AP-01 TCGA-HC-A6AQ-01 TCGA-HC-A6AS-01 TCGA-HC-A6HX-01 TCGA-HC-A6HY-01 TCGA-HC-A76W-01 TCGA-HC-A76X-01 TCGA-HC-A8CY-01 TCGA-HC-A8D0-01 TCGA-HC-A8D1-01 TCGA-HC-A9TE-01 TCGA-HC-A9TH-01 TCGA-HI-7168-01 TCGA-HI-7169-01 TCGA-HI-7170-01 TCGA-HI-7171-01 TCGA-J4-8198-01 TCGA-J4-8200-01 TCGA-J4-A67K-01 TCGA-J4-A67L-01 TCGA-J4-A67M-01 TCGA-J4-A67N-01 TCGA-J4-A67O-01 TCGA-J4-A67Q-01 TCGA-J4-A67R-01 TCGA-J4-A67S-01 TCGA-J4-A67T-01 TCGA-J4-A6G1-01 TCGA-J4-A6G3-01 TCGA-J4-A6M7-01 TCGA-J4-A83I-01 TCGA-J4-A83J-01 TCGA-J4-A83K-01 TCGA-J4-A83L-01 TCGA-J4-A83M-01 TCGA-J4-A83N-01 TCGA-J4-AATV-01 TCGA-J4-AATZ-01 TCGA-J4-AAU2-01 TCGA-J9-A52B-01 TCGA-J9-A52C-01 TCGA-J9-A52D-01 TCGA-J9-A52E-01 TCGA-J9-A8CK-01 TCGA-J9-A8CL-01 TCGA-J9-A8CM-01 TCGA-J9-A8CN-01 TCGA-J9-A8CP-01 TCGA-KC-A4BL-01 TCGA-KC-A4BN-01 TCGA-KC-A4BR-01 TCGA-KC-A4BV-01 TCGA-KC-A7F3-01 TCGA-KC-A7F5-01 TCGA-KC-A7F6-01 TCGA-KC-A7FA-01 TCGA-KC-A7FD-01 TCGA-KC-A7FE-01 TCGA-KK-A59V-01 TCGA-KK-A59X-01 TCGA-KK-A59Y-01 TCGA-KK-A59Z-01 TCGA-KK-A5A1-01 TCGA-KK-A6DY-01 TCGA-KK-A6E0-01 TCGA-KK-A6E1-01 TCGA-KK-A6E2-01 TCGA-KK-A6E3-01 TCGA-KK-A6E4-01 TCGA-KK-A6E5-01 TCGA-KK-A6E6-01 TCGA-KK-A6E7-01 TCGA-KK-A6E8-01 TCGA-KK-A7AP-01 TCGA-KK-A7AQ-01 TCGA-KK-A7AU-01 TCGA-KK-A7AV-01 TCGA-KK-A7AW-01 TCGA-KK-A7AY-01 TCGA-KK-A7AZ-01 TCGA-KK-A7B0-01 TCGA-KK-A7B1-01 TCGA-KK-A7B2-01 TCGA-KK-A7B3-01 TCGA-KK-A7B4-01 TCGA-KK-A8I4-01 TCGA-KK-A8I5-01 TCGA-KK-A8I6-01 TCGA-KK-A8I7-01 TCGA-KK-A8I8-01 TCGA-KK-A8I9-01 TCGA-KK-A8IA-01 TCGA-KK-A8IB-01 TCGA-KK-A8IC-01 TCGA-KK-A8ID-01 TCGA-KK-A8IF-01 TCGA-KK-A8IG-01 TCGA-KK-A8IH-01 TCGA-KK-A8II-01 TCGA-KK-A8IJ-01 TCGA-KK-A8IK-01 TCGA-KK-A8IL-01 TCGA-KK-A8IM-01 TCGA-M7-A71Y-01 TCGA-M7-A71Z-01 TCGA-M7-A720-01 TCGA-M7-A721-01 TCGA-M7-A722-01 TCGA-M7-A723-01 TCGA-M7-A724-01 TCGA-M7-A725-01 TCGA-MG-AAMC-01 TCGA-QU-A6IL-01 TCGA-QU-A6IM-01 TCGA-QU-A6IN-01 TCGA-QU-A6IO-01 TCGA-QU-A6IP-01 TCGA-SU-A7E7-01 TCGA-TK-A8OK-01 TCGA-TP-A8TT-01 TCGA-TP-A8TV-01 TCGA-V1-A8MF-01 TCGA-V1-A8MG-01 TCGA-V1-A8MK-01 TCGA-V1-A8ML-01 TCGA-V1-A8MM-01 TCGA-V1-A8MU-01 TCGA-V1-A8WL-01 TCGA-V1-A8WN-01 TCGA-V1-A8WS-01 TCGA-V1-A8WV-01 TCGA-V1-A8WW-01 TCGA-V1-A8X3-01 TCGA-V1-A9O5-01 TCGA-V1-A9O7-01 TCGA-V1-A9O9-01 TCGA-V1-A9OA-01 TCGA-V1-A9OF-01 TCGA-V1-A9OH-01 TCGA-V1-A9OL-01 TCGA-V1-A9OQ-01 TCGA-V1-A9OT-01 TCGA-V1-A9OX-01 TCGA-V1-A9OY-01 TCGA-V1-A9Z7-01 TCGA-V1-A9Z8-01 TCGA-V1-A9Z9-01 TCGA-V1-A9ZG-01 TCGA-V1-A9ZI-01 TCGA-V1-A9ZK-01 TCGA-V1-A9ZR-01 TCGA-VN-A88I-01 TCGA-VN-A88K-01 TCGA-VN-A88L-01 TCGA-VN-A88M-01 TCGA-VN-A88N-01 TCGA-VN-A88O-01 TCGA-VN-A88P-01 TCGA-VN-A88Q-01 TCGA-VN-A88R-01 TCGA-VN-A943-01 TCGA-VP-A872-01 TCGA-VP-A875-01 TCGA-VP-A876-01 TCGA-VP-A878-01 TCGA-VP-A879-01 TCGA-VP-A87B-01 TCGA-VP-A87C-01 TCGA-VP-A87D-01 TCGA-VP-A87E-01 TCGA-VP-A87H-01 TCGA-VP-A87J-01 TCGA-VP-A87K-01 TCGA-VP-AA1N-01 TCGA-WW-A8ZI-01 TCGA-X4-A8KQ-01 TCGA-X4-A8KS-01 TCGA-XA-A8JR-01 TCGA-XJ-A83F-01 TCGA-XJ-A83G-01 TCGA-XJ-A83H-01 TCGA-XJ-A9DI-01 TCGA-XJ-A9DK-01 TCGA-XJ-A9DQ-01 TCGA-XJ-A9DX-01 TCGA-XK-AAIR-01 TCGA-XK-AAIV-01 TCGA-XK-AAIW-01 TCGA-XK-AAJ3-01 TCGA-XK-AAJA-01 TCGA-XK-AAJP-01 TCGA-XK-AAJR-01 TCGA-XK-AAJT-01 TCGA-XK-AAJU-01 TCGA-XK-AAK1-01 TCGA-XQ-A8TA-01 TCGA-XQ-A8TB-01 TCGA-Y6-A8TL-01 TCGA-Y6-A9XI-01 TCGA-YJ-A8SW-01 TCGA-YL-A8HJ-01 TCGA-YL-A8HK-01 TCGA-YL-A8HL-01 TCGA-YL-A8HM-01 TCGA-YL-A8HO-01 TCGA-YL-A8S8-01 TCGA-YL-A8S9-01 TCGA-YL-A8SA-01 TCGA-YL-A8SB-01 TCGA-YL-A8SC-01 TCGA-YL-A8SH-01 TCGA-YL-A8SI-01 TCGA-YL-A8SJ-01 TCGA-YL-A8SK-01 TCGA-YL-A8SL-01 TCGA-YL-A8SO-01 TCGA-YL-A8SP-01 TCGA-YL-A8SQ-01 TCGA-YL-A8SR-01 TCGA-YL-A9WH-01 TCGA-YL-A9WI-01 TCGA-YL-A9WJ-01 TCGA-YL-A9WK-01 TCGA-YL-A9WL-01 TCGA-YL-A9WX-01 TCGA-YL-A9WY-01 TCGA-ZG-A8QW-01 TCGA-ZG-A8QX-01 TCGA-ZG-A8QY-01 TCGA-ZG-A8QZ-01 TCGA-ZG-A9KY-01 TCGA-ZG-A9L0-01 TCGA-ZG-A9L1-01 TCGA-ZG-A9L2-01 TCGA-ZG-A9L4-01 TCGA-ZG-A9L5-01 TCGA-ZG-A9L6-01 TCGA-ZG-A9L9-01 TCGA-ZG-A9LB-01 TCGA-ZG-A9LM-01 TCGA-ZG-A9LN-01 TCGA-ZG-A9LS-01 TCGA-ZG-A9LU-01 TCGA-ZG-A9LY-01 TCGA-ZG-A9LZ-01 TCGA-ZG-A9M4-01 TCGA-ZG-A9MC-01 TCGA-ZG-A9N3-01 TCGA-ZG-A9ND-01 TCGA-ZG-A9NI-01 TCGA-V1-A9O5-06

Solid Tissue Normal Solid Tissue Normal Solid Tissue Normal Solid Tissue Normal Solid Tissue Normal Solid Tissue Normal Solid Tissue Normal Solid Tissue Normal Solid Tissue Normal Solid Tissue Normal Solid Tissue Normal Solid Tissue Normal Solid Tissue Normal Solid Tissue Normal Solid Tissue Normal Solid Tissue Normal Solid Tissue Normal Solid Tissue Normal Solid Tissue Normal Solid Tissue Normal Solid Tissue Normal Solid Tissue Normal Solid Tissue Normal Solid Tissue Normal Solid Tissue Normal Solid Tissue Normal Solid Tissue Normal Solid Tissue Normal Solid Tissue Normal Solid Tissue Normal Solid Tissue Normal Solid Tissue Normal Solid Tissue Normal Solid Tissue Normal Solid Tissue Normal Solid Tissue Normal Solid Tissue Normal Solid Tissue Normal Solid Tissue Normal Solid Tissue Normal Solid Tissue Normal Solid Tissue Normal Solid Tissue Normal Solid Tissue Normal Solid Tissue Normal Solid Tissue Normal Solid Tissue Normal Solid Tissue Normal Solid Tissue Normal Solid Tissue Normal Solid Tissue Normal Solid Tissue Normal Primary Tumor Primary Tumor Primary Tumor Primary Tumor Primary Tumor Primary Tumor Primary Tumor Primary Tumor Primary Tumor Primary Tumor Primary Tumor Primary Tumor Primary Tumor Primary Tumor Primary Tumor Primary Tumor Primary Tumor Primary Tumor Primary Tumor Primary Tumor Primary Tumor Primary Tumor Primary Tumor Primary Tumor Primary Tumor Primary Tumor Primary Tumor Primary Tumor Primary Tumor Primary Tumor Primary Tumor Primary Tumor Primary Tumor Primary Tumor Primary Tumor Primary Tumor Primary Tumor Primary Tumor Primary Tumor Primary Tumor Primary Tumor Primary Tumor Primary Tumor Primary Tumor Primary Tumor Primary Tumor Primary Tumor Primary Tumor Primary Tumor Primary Tumor Primary Tumor Primary Tumor Primary Tumor Primary Tumor Primary Tumor Primary Tumor Primary Tumor Primary Tumor Primary Tumor Primary Tumor Primary Tumor Primary Tumor Primary Tumor Primary Tumor Primary Tumor Primary Tumor Primary Tumor Primary Tumor Primary Tumor Primary Tumor Primary Tumor Primary Tumor Primary Tumor Primary Tumor Primary Tumor Primary Tumor Primary Tumor Primary Tumor Primary Tumor Primary Tumor Primary Tumor Primary Tumor Primary Tumor Primary Tumor Primary Tumor Primary Tumor Primary Tumor Primary Tumor Primary Tumor Primary Tumor Primary Tumor Primary Tumor Primary Tumor Primary Tumor Primary Tumor Primary Tumor Primary Tumor Primary Tumor Primary Tumor Primary Tumor Primary Tumor Primary Tumor Primary Tumor Primary Tumor Primary Tumor Primary Tumor Primary Tumor Primary Tumor Primary Tumor Primary Tumor Primary Tumor Primary Tumor Primary Tumor Primary Tumor Primary Tumor Primary Tumor Primary Tumor Primary Tumor Primary Tumor Primary Tumor Primary Tumor Primary Tumor Primary Tumor Primary Tumor Primary Tumor Primary Tumor Primary Tumor Primary Tumor Primary Tumor Primary Tumor Primary Tumor Primary Tumor Primary Tumor Primary Tumor Primary Tumor Primary Tumor Primary Tumor Primary Tumor Primary Tumor Primary Tumor Primary Tumor Primary Tumor Primary Tumor Primary Tumor Primary Tumor Primary Tumor Primary Tumor Primary Tumor Primary Tumor Primary Tumor Primary Tumor Primary Tumor Primary Tumor Primary Tumor Primary Tumor Primary Tumor Primary Tumor Primary Tumor Primary Tumor Primary Tumor Primary Tumor Primary Tumor Primary Tumor Primary Tumor Primary Tumor Primary Tumor Primary Tumor Primary Tumor Primary Tumor Primary Tumor Primary Tumor Primary Tumor Primary Tumor Primary Tumor Primary Tumor Primary Tumor Primary Tumor Primary Tumor Primary Tumor Primary Tumor Primary Tumor Primary Tumor Primary Tumor Primary Tumor Primary Tumor Primary Tumor Primary Tumor Primary Tumor Primary Tumor Primary Tumor Primary Tumor Primary Tumor Primary Tumor Primary Tumor Primary Tumor Primary Tumor Primary Tumor Primary Tumor Primary Tumor Primary Tumor Primary Tumor Primary Tumor Primary Tumor Primary Tumor Primary Tumor Primary Tumor Primary Tumor Primary Tumor Primary Tumor Primary Tumor Primary Tumor Primary Tumor Primary Tumor Primary Tumor Primary Tumor Primary Tumor Primary Tumor Primary Tumor Primary Tumor Primary Tumor Primary Tumor Primary Tumor Primary Tumor Primary Tumor Primary Tumor Primary Tumor Primary Tumor Primary Tumor Primary Tumor Primary Tumor Primary Tumor Primary Tumor Primary Tumor Primary Tumor Primary Tumor Primary Tumor Primary Tumor Primary Tumor Primary Tumor Primary Tumor Primary Tumor Primary Tumor Primary Tumor Primary Tumor Primary Tumor Primary Tumor Primary Tumor Primary Tumor Primary Tumor Primary Tumor Primary Tumor Primary Tumor Primary Tumor Primary Tumor Primary Tumor Primary Tumor Primary Tumor Primary Tumor Primary Tumor Primary Tumor Primary Tumor Primary Tumor Primary Tumor Primary Tumor Primary Tumor Primary Tumor Primary Tumor Primary Tumor Primary Tumor Primary Tumor Primary Tumor Primary Tumor Primary Tumor Primary Tumor Primary Tumor Primary Tumor Primary Tumor Primary Tumor Primary Tumor Primary Tumor Primary Tumor Primary Tumor Primary Tumor Primary Tumor Primary Tumor Primary Tumor Primary Tumor Primary Tumor Primary Tumor Primary Tumor Primary Tumor Primary Tumor Primary Tumor Primary Tumor Primary Tumor Primary Tumor Primary Tumor Primary Tumor Primary Tumor Primary Tumor Primary Tumor Primary Tumor Primary Tumor Primary Tumor Primary Tumor Primary Tumor Primary Tumor Primary Tumor Primary Tumor Primary Tumor Primary Tumor Primary Tumor Primary Tumor Primary Tumor Primary Tumor Primary Tumor Primary Tumor Primary Tumor Primary Tumor Primary Tumor Primary Tumor Primary Tumor Primary Tumor Primary Tumor Primary Tumor Primary Tumor Primary Tumor Primary Tumor Primary Tumor Primary Tumor Primary Tumor Primary Tumor Primary Tumor Primary Tumor Primary Tumor Primary Tumor Primary Tumor Primary Tumor Primary Tumor Primary Tumor Primary Tumor Primary Tumor Primary Tumor Primary Tumor Primary Tumor Primary Tumor Primary Tumor Primary Tumor Primary Tumor Primary Tumor Primary Tumor Primary Tumor Primary Tumor Primary Tumor Primary Tumor Primary Tumor Primary Tumor Primary Tumor Primary Tumor Primary Tumor Primary Tumor Primary Tumor Primary Tumor Primary Tumor Primary Tumor Primary Tumor Primary Tumor Primary Tumor Primary Tumor Primary Tumor Primary Tumor Primary Tumor Primary Tumor Primary Tumor Primary Tumor Primary Tumor Primary Tumor Primary Tumor Primary Tumor Primary Tumor Primary Tumor Primary Tumor Primary Tumor Primary Tumor Primary Tumor Primary Tumor Primary Tumor Primary Tumor Primary Tumor Primary Tumor Primary Tumor Primary Tumor Primary Tumor Primary Tumor Primary Tumor Primary Tumor Primary Tumor Primary Tumor Primary Tumor Primary Tumor Primary Tumor Primary Tumor Primary Tumor Primary Tumor Primary Tumor Primary Tumor Primary Tumor Primary Tumor Primary Tumor Primary Tumor Primary Tumor Primary Tumor Primary Tumor Primary Tumor Primary Tumor Primary Tumor Primary Tumor Primary Tumor Primary Tumor Primary Tumor Primary Tumor Primary Tumor Primary Tumor Primary Tumor Primary Tumor Primary Tumor Primary Tumor Primary Tumor Primary Tumor Primary Tumor Primary Tumor Primary Tumor Primary Tumor Primary Tumor Primary Tumor Primary Tumor Primary Tumor Primary Tumor Primary Tumor Primary Tumor Primary Tumor Primary Tumor Primary Tumor Primary Tumor Primary Tumor Primary Tumor Primary Tumor Primary Tumor Primary Tumor Primary Tumor Primary Tumor Primary Tumor Primary Tumor Primary Tumor Primary Tumor Primary Tumor Primary Tumor Primary Tumor Primary Tumor Primary Tumor Primary Tumor Primary Tumor Primary Tumor Primary Tumor Primary Tumor Primary Tumor Primary Tumor Primary Tumor Primary Tumor Primary Tumor Primary Tumor Primary Tumor Primary Tumor Primary Tumor Primary Tumor Primary Tumor Primary Tumor Primary Tumor Primary Tumor Primary Tumor Primary Tumor Primary Tumor Primary Tumor Primary Tumor Primary Tumor Primary Tumor Primary Tumor Primary Tumor Primary Tumor Primary Tumor Primary Tumor Primary Tumor Primary Tumor Primary Tumor Primary Tumor Metastatic

ENSG00000121410.10 A1BG 0.091467462 0.061097195 0.063237382 0.006095682 0.044114789 0.036750946 0.047080491 0.125232226 0.085788714 0.047899827 0.125821941 0.112563629 0.114319402 0.178228036 0.069761984 0.129405748 0.101610931 0.045095729 0.047117503 0.053662993 0.195819937 0.080126086 0.069769715 0.072772222 0.063393537 0.096929531 0.076218958 0.036526986 0.012198746 0.047258126 0.035054266 0.063747365 0.060701699 0.020875738 0.091207208 0.089366393 0.03388764 0.035625638 0.310119314 0.179654867 0.158741851 0.155939008 0.054620773 0.11768283 0.204764246 0.128957123 0.11596434 0.156912666 0.132876839 0.145244579 0.069197728 0.111869549 0.061450912 0.030281293 0.04102008 0.043500679 0.058153333 0.007681803 0.046601851 0.033747827 0.023911929 0.043215339 0.126136901 0.021324357 0.027952669 0.006665701 0.020256708 0.015690908 0.028631613 0.017499794 0.04483895 0.038025517 0.019382915 0.028815498 0.043984673 0.135820234 0.034398702 0.256997263 0.022844254 0 0.068141013 0.053659365 0.033398709 0.110999086 0.031929816 0.005537848 0.021719861 0.036213433 0.012903057 0 0.059918069 0.192419341 0.09550871 0.042049068 0.004956778 0.103661979 0.02194195 0.015456954 0.034175386 0.044959855 0.040034647 0.046232107 0.058282137 0.075097504 0.028339454 0.04643731 0.043480038 0.023092523 0.051255533 0.051019166 0.026367781 0.016140082 0.07294591 0.115785817 0.111417703 0.055886455 0.00490255 0.026399455 0.017968844 0.02609898 0.018215663 0.025212414 0 0.033437168 0.037339973 0.096909469 0.020891129 0.039600207 0.032660522 0.047947706 0.06519541 0.026389482 0.014852769 0.027236383 0.028089569 0.024060481 0.068763776 0.048515787 0.048670199 0.042952398 0.050481083 0.089524418 0.097853386 0.085120306 0.038145322 0.031647257 0.063946142 0.048900305 0.028796771 0.069662802 0.032249048 0.126480739 0.082019671 0.048797624 0.019045536 0.022199783 0.040607516 0.072255202 0.044048333 0.0480834 0.050581606 0.034682127 0.063674789 0.076818468 0.109746014 0.024758895 0.086154979 0.088645209 0.14958803 0.158456109 0.01782865 0.029337801 0.01468488 0.117132569 0.030325891 0.021496183 0.03154047 0.037003922 0.007238214 0.066458049 0.215882613 0.222963203 0.054466137 0.287391791 0.063766928 0.016319105 0.138359292 0.136371349 0.067225933 0.154928571 0.098408886 0.045443934 0.080649929 0.089256535 0.01938599 0.029428682 0.143571783 0.065409771 0.142450135 0.041712654 0.020793391 0.186713378 0.067640239 0.019749096 0.02482192 0.041446306 0.099537273 0.017451774 0.019943009 0.016716084 0.032491935 0.039367958 0.029480022 0.041031059 0.024096219 0.015977384 0.056144622 0.079024385 0.004927397 0.026650411 0.005911221 0.017605473 0.070827835 0.036054882 0.017194875 0.060416032 0.05846066 0.033433599 0.069463409 0.063049789 0.053155105 0.046583286 0.016562311 0.095453342 0.085624368 0.010691735 0.076207765 0.026788963 0.05200279 0 0.02282925 0.14597518 0.026669482 0.0165731 0.341642821 0.025404716 0.029066287 0.016218569 0.031334102 0.195387417 0.048908266 0.04989864 0.019252999 0.035694495 0.129867131 0.021557609 0.023933043 0.1176619 0.017821464 0.011225179 0.035355332 0.156771311 0.072424776 0.029080899 0.239005326 0.024195977 0.011948219 0.096464483 0.053543803 0.051090821 0.032599733 0.045008934 0.087070193 0.156105037 0.027089333 0.01840954 0.023748846 0.040522981 0 0.052910049 0.045203292 0.050853838 0.018345195 0.032971231 0.133666691 0.01063527 0.021091603 0.057986565 0.067692351 0.048286148 0.035852147 0.062044816 0.022134949 0.015895203 0.055348145 0.007099426 0.044886128 0.061529559 0.056842602 0.068455792 0.110239892 0.043968585 0.096607348 0.018859952 0.045735524 0.062104146 0.079782711 0.204573478 0.076604217 0.045715239 0.04407147 0.047231926 0.04198878 0.025589629 0.055653753 0.032742449 0.0370439 0.051073322 0.030806382 0.216169766 0.063251227 0.044451601 0.027330886 0.067865841 0.131177988 0.066447346 0.078249035 0.06518464 0.033290581 0.107048688 0.08984287 0.059198326 0.038580296 0.033578857 0.03588775 0.083665799 0.043867109 0.120787263 0.007479828 0.078607127 0.06273813 0.086233809 0.017483882 0.02247212 0.15500309 0.109780015 0.017958066 0.12431494 0.063701205 0.051925143 0.033548819 0.062476806 0.028646071 0.036491271 0.109273623 0.075541364 0.035797956 0.027380979 0.008064843 0.167268479 0.091096769 0.007291929 0.007876515 0.033861269 0.083913938 0.006490715 0.079534216 0.045156084 0.040527768 0.006934231 0.043499291 0.051464132 0.105614922 0.127571437 0.04181689 0.019863325 0.030736732 0.021939585 0.02216467 0.065815873 0.322477551 0.034514251 0.125991063 0.008961489 0.032601529 0.069532089 0.036553095 0.058056142 0.105786786 0.046369316 0.064319224 0.282203707 0.040917548 0.069042532 0.055733335 0.244505007 0.013836569 0.008233678 0.151548416 0.057430065 0.054779992 0.03582716 0.066092887 0.061085372 0.025638078 0.064376739 0.011546006 0.112054171 0.009138768 0.018283318 0.017600761 0.035651757 0.016543458 0.008675354 0.02913556 0.074361197 0.057890174 0.057291374 0.071887155 0.117035136 0.116294702 0.21729198 0.086786784 0.100125837 0.048018123 0.117096666 0.094333141 0.124395999 0.050135419 0.02641432 0.14422198 0.035354949 0.096192763 0.052443202 0.025861984 0.028777094 0.2010712 0.042850011 0.031669271 0.084340433 0.201851886 0.101454717 0.07015175 0.018401553 0.041730631 0.039081161 0.041635004 0.075043697 0.117855888 0.048928578 0.052642343 0.055414495 0.021649332 0.021416624 0.034844704 0.030081182 0.348335974 0.03578058 0.046916636 0.018011643 0.009131638 0.156820884 0.015365499 0.0162379 0.145257684 0.071378699 0.078247037 0.086955379 0.012466637 0.030888951 0.021759544 0.028326888 0.088475113 0.088425367 0.05659022 0.018088257 0.090983501 0.029561097 0.0845993 0.10726502 0.126621473 0.166572793 0.028298965 0.012554799 0.023530333 0 0.166229196 0.022509506 0.060677302 0.09644422 0.05024438 0 0.129618589 0.050799296 0.066434964 0.055788022 0.335034196 0.059663707 0.329353287 0 0.04048347 0.107083885 0.020810113 0.040467586 0.017141339 0.069572834 0.163107459 0.049366044 0.047891062 0.070156903 0.100226633 0.015799112 0.063915832 0.226522792 0.09551676 0.07311186 0.006812373 0.024091628 0.104708776 0.056347258 0.059210507 0.06890369 0.125601743 0.139267731 0.012812014 0.025835683 0.033689678 0.104599663 0.087215759 0.046284029 0.045374266 0.09352935 0.163466923 0.061926839 0.042559261 0.010994845 0.149456757 0.051825385 0.06621018 0.100499459 0.034563664 0.012352817 0.014254889 0.093769568 0 0.045496712 0.022665904 0.066956308

ENSG00000175899.13 A2M 5.514677113 6.042150169 4.748808251 6.388853934 6.060058424 7.755135245 6.100671598 7.878020197 9.620651215 6.345989711 6.698722971 6.303253782 8.211404354 8.169438653 7.740402457 7.712647902 5.521849806 7.10563356 6.542387135 6.305997977 9.288119605 6.20306329 6.232162222 8.372118295 7.351939246 7.529504533 6.250921541 5.79402002 5.341818966 7.383086063 5.751846485 4.819353036 5.181933307 7.052870974 4.686982283 5.956656068 6.430596758 6.753443798 4.467129461 5.667716244 5.30641464 4.910772739 5.734016113 6.089413287 5.161060475 7.521884258 5.568313193 3.871591409 7.16663763 5.554382738 6.863502898 7.402353265 5.149918504 7.156932292 5.4517188 5.937892409 5.85266953 3.639717374 6.33008451 5.840168227 6.794927459 6.343535554 7.872366898 5.465201012 7.913531445 7.227461492 5.784159974 5.088033128 6.359962268 5.661955963 6.799557189 5.03053753 5.712838209 5.706065286 6.731157345 6.485861343 5.392649304 6.989535247 6.537766614 7.158022426 6.886603023 6.53500801 6.183468035 5.908348052 5.447614271 5.724374529 6.422128064 7.263172781 7.126508754 5.323476336 6.339804804 5.446175297 6.401085502 7.415440309 5.38246935 7.648076151 7.719172002 5.119309709 6.512420309 7.116865914 6.568457844 6.06392613 7.340407129 6.228792391 8.208533641 5.567925545 5.025431707 6.475716619 6.742214849 7.076395182 6.043400248 7.325997411 5.318838202 8.462701543 5.800834882 7.470568532 7.527531234 7.795564068 6.654908537 6.154058825 6.225889087 6.909124946 7.744960769 7.082628149 7.305679537 7.212467529 7.578118393 7.778202986 7.176969519 6.676904216 6.081848989 7.833145153 6.78473584 5.540779113 5.827995771 8.000140653 6.799208646 5.961374052 5.61689841 5.584402266 7.378512327 7.149221622 7.119157174 7.668731978 6.593983159 7.109769629 6.572021037 5.842005457 7.245038178 7.110557054 8.39524903 6.509386729 8.308015918 7.627732852 5.387801484 7.568241219 6.26690383 7.332516363 5.966731085 5.940925752 5.692383321 6.732616515 5.357347188 5.654270786 5.658384964 4.889966588 6.762479184 7.991171383 5.240722009 6.580643384 4.24839583 5.524271185 6.203062265 5.472233959 5.978570973 5.736123036 5.648500476 7.497447088 4.287624918 6.933610391 6.173030041 4.606483712 5.465322053 6.41444812 6.533215871 6.342446939 5.736195587 6.171821115 4.80760231 6.432916643 9.574082936 6.053350077 7.608088454 7.223906812 3.900447042 4.584890425 5.396533507 4.367784498 4.655614786 6.38389826 4.358245862 5.675626878 5.663713136 6.705525467 5.218409191 6.225018419 5.349932481 7.281342043 7.303749456 5.24529528 5.578781382 4.492355296 6.929468004 6.738978786 5.471649806 6.205715734 6.675524514 7.909358056 5.35574566 6.486854965 4.768498753 6.476576628 5.39737623 6.10841496 5.438997123 6.628323313 6.68226103 6.715320327 6.22722395 6.981729842 6.11279145 8.054244685 5.125396975 5.377278476 6.796968023 6.23301756 7.184251761 6.044525367 9.574954516 6.470203423 5.682954596 4.392139856 4.795003141 7.514360268 7.732622205 4.932700944 5.929705696 4.68100626 6.66693159 7.502061802 5.905416129 8.468737742 6.035036211 7.533264582 5.748094933 6.582813465 6.332302645 5.111638984 5.982427131 6.805825584 7.399228348 6.615091481 7.804924867 5.160977399 8.976911975 6.459461331 5.990212924 7.136902302 5.828702389 6.929867043 7.602018323 7.29045755 4.96334321 8.0590114 6.092168166 5.319054904 4.968463364 6.947273454 4.320399024 6.086612086 5.256809807 6.179857583 7.172045331 4.879311949 6.166853199 5.702319044 5.682451726 8.055728143 7.031587477 6.927708521 7.188300182 7.117995895 5.577927804 7.44695891 6.030798619 6.220015048 5.167558002 6.462026651 5.35569461 6.766166918 5.881036196 4.786449103 6.378868048 5.525987486 5.815770812 7.008531852 6.285458334 7.196392135 6.811783688 6.944615091 6.331236958 8.754063751 6.655340469 6.684434431 6.228449028 5.676012724 6.30200904 7.130609178 5.925141542 6.214536187 8.738719624 6.165816913 4.693658319 5.310467087 6.964264712 6.676076038 6.856734308 6.129333936 6.299742748 5.65092152 6.3315465 6.129257651 4.925599804 6.375417124 5.285418651 6.588709958 6.53909206 7.242657749 4.033247694 7.913147014 7.582411648 7.419485873 7.097987053 5.827616199 7.107754392 7.024168677 4.165560294 6.437673247 7.466031724 5.922910319 4.24697566 6.412014464 5.473098947 4.567456767 8.545976591 6.125991228 6.511537355 5.741286679 6.756298972 6.25984357 5.951347148 5.934282487 5.491277492 5.407363674 6.200826015 5.567889839 6.273421969 6.784601118 5.876515239 5.244577734 4.930796012 6.305838383 4.004216656 6.783418016 5.680571132 5.992649057 5.626333331 6.869770736 7.022355653 6.503618682 6.548434504 6.397614683 7.608031984 6.959506457 5.965114412 7.247546755 6.22705553 7.198206726 5.642702027 6.487538248 7.171745932 4.966995114 5.378635093 7.603631789 6.122983878 4.456113435 6.3061558 4.833990788 6.382107731 6.606229085 5.59910898 3.936784304 5.854524432 6.338183805 4.951183669 6.29292425 4.965215018 5.664432148 3.941406653 5.612884135 5.332763995 5.155197044 5.70963459 5.722103392 6.542594557 7.605229388 6.989988657 5.279211403 6.595956497 6.263744107 5.786568845 5.914570118 6.638724124 6.933876271 7.175301412 5.886283076 4.676482474 6.721526392 6.419658175 7.42054567 6.221349243 7.82221279 6.568423334 6.612647551 5.218828115 6.10020087 6.561861326 7.207580486 5.194628344 5.755036302 6.228529128 6.87747201 8.218390659 6.950551795 7.215201773 6.135856752 5.697997041 7.399193528 9.365281519 7.352288143 6.535488842 4.833914618 5.532137115 5.875684856 7.723653064 5.499582989 6.660220724 4.276385541 7.218109356 4.977912407 5.072812326 7.263272658 5.990976969 5.636800436 7.563433362 5.038378212 7.205543078 5.942689219 5.639753054 4.922304314 7.678200693 6.455671035 6.452160505 4.953732427 6.479528625 5.980149046 4.689517879 5.108794524 7.691062337 6.094512027 5.694799065 5.33107226 6.503523524 7.819337869 5.189351159 6.126103054 5.462567171 5.625487817 6.541353162 6.308678441 6.487756113 5.771496328 4.633745281 7.02557293 6.063541591 6.156083144 5.630937708 8.737592932 7.484448284 5.7826518 5.531529246 7.401049249 6.818118865 5.198233451 4.863845318 8.272407228 6.336460502 6.36890063 7.482082249 6.412716441 6.657893474 5.753885508 6.611753902 6.634036431 7.390301062 7.274590793 6.617632647 6.742538055 6.653490554 6.601717654 5.427951401 6.59917762 7.702628209 7.23995131 6.445528989 6.100266413 6.864402612 5.382640705 7.442681228 5.678891872 8.70187539 7.903165093 5.389581688 8.063285841 7.573544446 5.201724098 6.816835534 7.667609812 7.948637842 5.74916379 6.916015975 6.507444628 6.06107937 7.605015815 6.885760313 5.288640643 6.676556348 5.33123449

ENSG00000166535.18 A2ML1 1.193176647 1.054304528 0.528490982 0.372919503 0.336609934 0.178993994 0.338973599 0.672648592 0.699682421 0.589399935 0.762360504 0.636346572 0.237302204 0.65037375 0.607059133 0.547008819 0.70967683 0.052864287 1.09842557 0.421734295 0.419931079 0.740200009 0.780348654 0.371756664 0.84236681 0.332532173 0.189893719 0.159904745 0.360791568 0.239247727 0.40957575 1.477298121 0.092211263 0.406862652 0.632422886 0.33959814 0.105746194 0.132247156 0.044979416 0.101305435 0.047242886 0.04256855 0.530557592 0.062352841 0.027972168 0.244454765 0.575054003 0.029122086 0.376825101 0.510340475 1.688629544 0.478035312 0.506604071 0.11812723 0.063261002 0.03982089 0.183170799 0.101937977 0.072383174 0.30601493 0.315529462 0.100681182 1.628755811 0.30670187 0.555867913 0.060995109 0.07627769 0.046829196 1.730208771 0.19989317 0.096620115 0.476508223 0.28993492 0.122382559 0.052915385 0.015540546 0.264465576 0.347945533 1.339098766 0.571739566 0.293378237 0.247415781 0.109993167 0.43164148 0.143188928 0.243107654 0.362532748 0.396722708 0.109491753 0.734084298 0.785778592 0.339616861 0.609383112 0.584648733 0.242605064 0.183156253 0.195363671 0.180077087 0.599748647 0.430045376 0.284866023 0.281959769 0.440736579 0.302097708 0.293719472 0.323324972 0.319107488 0.595636212 0.353596488 0.524417007 0.380184877 0.350067412 0.509111829 1.259123997 0.497660854 0.302068056 0.285064381 0.156169206 0.036722265 0.369678745 0.236010904 0.129991937 0.067919531 0.212420952 0.480808163 0.115239576 0.526124892 0.802553534 0.231117996 0.208201955 0.411040145 0.207172795 0.222199208 0.09249146 0.189291106 0.820219132 0.186951141 0.084676635 0.209074062 0.050907907 0.177440314 0.173674068 0.968154991 0.566048836 0.743269547 0.109799384 0.485097385 0.093886206 0.214568982 0.234367884 0.215613399 0.23993757 0.395124333 0.802700753 0.165419026 0.859538082 0.383203534 0.287690298 0.094946015 0.594741459 0.108749323 0.16365282 0.32315409 0.370159116 0.25276445 0.049373242 0.107872901 1.548207071 0.318704161 0.290288491 0.013061278 0.228790946 0.124052811 0.136157212 0.794196238 0.22672944 0.573380294 0.449737508 0.05850416 0.603863085 0.232200687 0.453390112 0.471889849 0.360521536 0.363399699 0.412211525 0.16043746 0.574757889 0.174037113 0.250977985 0.148509279 0.340540969 0.59800255 0.549785788 0.042199914 0.208434665 0.338029078 0.123068617 1.214930743 0.261990804 0.078258961 0.228534248 0.350506944 0.341578683 0.401071389 0.222463724 0.08082257 0.09923953 0.371795634 0.291304673 0.590738388 0.085014438 0.716521016 0.300343659 0.060897433 0.632065081 0.061480753 0.114179738 0.385046474 0.401776675 0.124443339 1.1601094 0.14080793 0.210792798 0.121271039 0.124970391 0.358323082 0.202452062 0.401114751 0.287518605 0.132347007 0.082025505 0.163934807 0.167721161 0.619803482 0.225685861 0.383549879 0.36305955 0.362768596 0.016225993 1.556953331 0.051726899 0.06495955 0.036117912 0.354432242 0.078655415 0.05529679 0.131047367 1.119756476 0.825127859 0.121274671 0.041182099 0.14165798 0.302872196 0.381428716 0.049766355 2.532923694 0.115155286 1.045180328 0.241351071 0.439462546 1.398065956 0.405734431 0.365738673 0.899029329 2.102922864 0.048534227 0.426635213 0.393643199 0.303330964 0.121351317 0.4562776 0.088142234 0.248599726 0.172290438 0.040090206 0.067361623 0.05683508 0.050674516 0.136883431 0.213661876 0.24727681 0.724944172 0.177641844 0.376084048 0.464131933 0.267809718 0.077542814 0.211770099 0.685366588 0.077530138 1.290612358 0.102325443 0.089239786 0.14077792 0.250257998 0.038774021 0.149105265 0.258154364 0.101243016 0.124355627 0.172888454 0.210870604 0.066147159 0.237815061 0.141968186 0.553696448 0.7259678 2.244644078 0.10441149 0.024341569 0.46818748 0.11236369 0.147933706 0.604522846 0.074347969 0.089772693 0.224063611 0.095013182 0.103487917 0.153657098 0.256443845 0.132926143 0.225387565 0.443287198 0.57652596 0.088818381 0.380652599 0.160668868 0.238932549 0.539531439 0.085792909 0.095435347 0.702897691 0.792662132 0.120801106 0.033806311 0.213722923 0.23485043 0.043648618 0.057463151 0.024168988 0.405312972 0.291476689 0.071632901 0.717879973 0.334666963 0.139428857 0.163857033 0.323407228 0.117928636 0.15352031 0.390413242 0.066039472 0.404592553 0.082668844 0.844686536 0.271068255 0.167358047 0.070171349 0.255913767 0.488551729 0.163653285 0.069705823 0.445060387 0.045659621 0.257030672 0.097291345 0.666451693 0.033914078 0.175725022 0.448126918 0.144547271 0.420181757 0.051438453 0.170536585 0.040330046 0.324428946 0.145555571 0.468908208 0.171138789 0.597928259 0.367749399 0.158608556 0.208463899 0.298918769 0.074209742 0.195094238 0.443847701 0.135591543 0.229662643 0.079820077 0.06128694 0.686611495 2.076980001 0.030271123 0.189142217 0.210361463 0.34047524 0.260271447 0.079443671 0.039258466 0.308526627 0.473517248 0.091623074 0.112837449 0.121881091 1.359593718 0.059118872 0.078565261 0.094008356 0.160958641 0.119635376 0.342539976 0.320761464 0.084972673 0.182376425 0.168393361 0.287708479 0.20376944 0.336566828 0.103402638 0.212528355 0.233744339 0.509214447 0.484253106 0.075244718 0.326584622 0.467185141 0.043214696 0.904360215 0.198810869 0.617510826 0.261839764 0.175026134 0.17024577 0.994300305 0.103552181 0.122029385 0.310541639 0.462913105 0.087386138 0.383022655 0.564445236 0.304018705 0.13145261 0.651973825 0.255844746 0.302713286 0.186329846 0.069712125 0.081751013 0.211109519 0.16391345 0.17727616 0.313487827 0.015727518 0.071085613 1.872537066 0.212258431 0.087727315 0.232992845 0.164022439 0.196491245 0.211281803 0.099094793 0.185964308 0.582058554 0.096300691 0.047174635 0.457375269 0.181609244 0.131465145 0.182977044 0.275052869 0.058697315 0.197612643 0.502202722 0.257561337 0.512390648 0.215397633 0.138425526 0.693006327 3.45454457 0.025794432 0.224825843 0.126801679 0.18924559 0.315786743 0.345830294 0.620917977 0.20660063 0.273622922 0.112959498 0.510645466 0.884832287 1.618348456 0.114167209 0.310833173 0.542544858 0.111905917 0.30565223 0.249284871 0.129120129 0.100466256 0.560001869 0.091745057 0.15236035 0.218529429 0.227543176 0.322934814 0.055558014 0.41733041 0.007254351 0.149553984 0.332835976 0.622963985 0.210425705 0.825718245 0.084176791 0.077979034 0.377413113 0.316314041 1.484815631 0.644044733 0.028319435 0.145708583 0.237247885 0.161036768 0.056913319 0.10112463 0.16568758 0.078181632 0.250307448 0.139891421 0.197708291 0.114136628 0.717991531 0.4551856 0.154543609 0.340288555 0.488823084 0.677309502 0.751175347 0.231979441 0.429851421 0.546326636 0.911569685

ENSG00000184389.9 A3GALT2 0 0.145187412 0.121323028 0 0 0.035988118 0 0 0.024514267 0 0.089124231 0 0 0.025391482 0.023138124 0.04538014 0.099547406 0 0 0.04680963 0.163303082 0.021350909 0 0.020727586 0 0.038736377 0 0 0 0 0.022974665 0.082661884 0.024075534 0 0.111969668 0.025559313 0 0.020037967 0.120288149 0.021868601 0.028290841 0 0 0.097701169 0 0 0 0.03768698 0.02163899 0.025784903 0.054473981 0.128111958 0.117961377 0 0.104729868 0 0 0.029850697 0 0.026497579 0.09145673 0.056155604 0 0 0.064837808 0.025928436 0.077750207 0 0.129863175 0.1062985 0.050076056 0.083741935 0.050064126 0.037499724 0.101295331 0.043940265 0.079558512 0.04072924 0 0.026822692 0.053637951 0 0.043443533 0.087647552 0.020919091 0.02156553 0 0.014289418 0 0 0.058684058 0.068442782 0 0.033035752 0 0 0 0 0.026834135 0 0 0.03034102 0.045844172 0.046892529 0.027749545 0.067688354 0.063407209 0 0.066548359 0.013494181 0.061205441 0.136354698 0 0.02341401 0.052400758 0 0 0 0 0.017087062 0.035452579 0.024687041 0 0.12673377 0 0.017732707 0 0 0.021399686 0 0.018534523 0 0 0 0.086230616 0 0 0.021310186 0.0274291 0 0.019978997 0 0 0.020057279 0.042612116 0.081203099 0.076706139 0 0.055854302 0.068232803 0.062480856 0 0.05887124 0 0.049201732 0.021736716 0.132008116 0.047565824 0 0.021118488 0.107858767 0.053961539 0 0.038113196 0.036738759 0.063763841 0.132761459 0.094478263 0 0 0.090750261 0.111628733 0 0.031507951 0.023804742 0 0.030884641 0 0.055740375 0.052306721 0.049792405 0.195897256 0.104782524 0.079013397 0.055647897 0.093316876 0 0.030784477 0.078660699 0.102979587 0 0 0.023016427 0 0.025253214 0 0.029258498 0.03238816 0.089723939 0.060846748 0 0 0 0 0 0.155917583 0 0 0.019526698 0 0.083332827 0 0.090410453 0.02023019 0 0 0.027755382 0 0.019200017 0 0 0 0 0 0.044465565 0 0.065253124 0.018798786 0 0 0 0 0.021581315 0 0.020178762 0 0 0 0 0 0 0.024849304 0 0.042872203 0 0 0 0.021134167 0.072570459 0.105308707 0.102526289 0.096133828 0.073969667 0.059411484 0.018881006 0 0.069190416 0 0.134018841 0.04346707 0.023172817 0.145954462 0.024036156 0.019046118 0.318402153 0.023691611 0.046233913 0.13318181 0.026457589 0.01687204 0 0 0.05308216 0.057528086 0.062859787 0.023983687 0.046138582 0.045263856 0 0.059070706 0 0.049803297 0.070544083 0.084540455 0 0.04120677 0.041011524 0.159370742 0.170452723 0 0 0.040795003 0 0 0 0.054689005 0 0 0 0.024743866 0 0.057129969 0.027680027 0.054704009 0.051075507 0.069309866 0.062855443 0 0.060337286 0.044769212 0.034629834 0.052743454 0.015088226 0 0 0 0 0 0.08866824 0.029161836 0 0 0.042583756 0.079406488 0.058547128 0.096553954 0.11629219 0.069510902 0.026137678 0.045092094 0.023999558 0.101515808 0 0.07769092 0.117086062 0.108272288 0 0 0 0 0.054747617 0.225079936 0.067288392 0 0.057108157 0.058359565 0.034954161 0.082338119 0.105359744 0.025650479 0 0 0.082564614 0.047451008 0.027519034 0 0 0.169990064 0.092011928 0 0.149764306 0.08342193 0.146901755 0 0.030435414 0 0 0.087127644 0.022812148 0.02696576 0.598102247 0 0.049618714 0 0 0.07626937 0.024127975 0 0.043083025 0 0.238569376 0.066820954 0.280260425 0 0.075462813 0 0.070722023 0.064802652 0.066318398 0.146118143 0 0.024207359 0 0.060265729 0.043831074 0.02406296 0.053441594 0.031977687 0.086731886 0.028397242 0.035987314 0 0.070477968 0.030224014 0 0 0.022520705 0.022635852 0 0.070310373 0 0.108866517 0.042796216 0.066588289 0.056505244 0 0.174102153 0.094893894 0 0.12465707 0 0 0.028898739 0 0.037741995 0.046992822 0.041804745 0.105094179 0 0.100785606 0.088600832 0.133760928 0.094235713 0 0.066565124 0 0.126319162 0 0.23121341 0 0.091315536 0.075180269 0.034765009 0 0 0 0.023439314 0 0.072148675 0.054634367 0 0.091823778 0.082983603 0.209358217 0.034120978 0.08662116 0 0 0.036873229 0 0.070017701 0.055335471 0.059258006 0.122537017 0.028219392 0.031492029 0.031148493 0 0.024309072 0.048094668 0.021305592 0.036865732 0.043985949 0 0 0 0 0.112454494 0.098889383 0.030815131 0 0.137303293 0.027709892 0.095523254 0.045717299 0.030152942 0.019578731 0.022040027 0.0549425 0 0.157886516 0 0.351725949 0.084263606 0.037541276 0.062276607 0.022892717 0 0.196696994 0 0.103389125 0 0.079831538 0 0.065992102 0 0.023950938 0.064107527 0.062201505 0.101885844 0.13830871 0.229560811 0.122598725 0.025728139 0.101786237 0.166250092 0 0 0.073162362 0.027856545 0.038918351 0.113832727 0.188254167 0 0.049534246 0.025297437 0 0.03990208 0.031656112 0 0.050672805 0.023457481 0.148503243 0.060652321 0 0 0 0.057862262 0.052110683 0 0.027139789 0.024088029 0.1080469 0.067381899 0 0.044555138 0.044050141 0.033162912

ENSG00000128274.14 A4GALT 4.139438323 3.380968985 2.92162143 3.152220871 3.390339141 3.754563281 2.644519183 3.422574281 3.756550937 3.651087519 3.628518332 2.410856882 3.923171121 3.782150102 3.31505211 3.442175181 3.655662653 4.020223113 3.964986522 3.584160341 3.591614174 3.675227557 3.808421235 3.45016415 4.300904375 3.130417125 3.996147932 4.054790563 3.409821192 4.005893722 3.705635104 4.503153411 2.562595165 3.331521433 3.212700932 3.949124388 3.710852735 3.791239797 5.251186295 5.246907283 4.711183007 4.878075696 3.526611729 5.010604142 5.320438832 3.564521514 3.13775932 5.349652663 3.009849294 3.186684531 4.402645847 4.154707227 3.684124061 3.178788472 2.614287526 3.107413422 2.975567332 2.261070098 3.239683333 4.149442677 3.375765867 2.491079287 3.38227569 3.293058637 3.911728728 3.292776866 2.23126712 2.055087032 3.912859712 2.696412738 3.848670162 3.160411135 2.528206928 1.953918318 1.938001096 2.00552348 2.061042144 3.029094676 2.203640357 3.103150872 2.950142104 3.002715545 2.584909636 2.299978898 3.346831567 2.485841556 2.933903018 4.101413708 2.211616784 2.504695189 3.354532199 2.792381507 2.973942565 4.004344716 3.102555856 3.248745947 3.248828703 2.938090436 3.030692239 3.27597981 2.992773436 3.187738716 4.20979028 3.811671331 2.727561122 2.358919589 2.57059048 2.705706848 3.105332936 3.118183309 3.71387737 3.240222589 3.183185775 2.782575442 2.873012192 2.68032503 2.322885089 3.013352876 2.772824825 2.938021559 2.478890865 2.481161764 2.201159801 3.057430427 2.645107767 2.644608398 3.684783 2.626543477 2.379162108 3.514069409 3.52064425 2.755800788 3.539818569 3.250595588 2.849777135 2.825618083 3.118706029 3.219149985 2.520330471 3.366971673 2.559667799 4.023473558 2.979681238 3.183948501 2.786135937 3.113202046 3.438567855 2.321746315 3.475923368 3.641071626 2.474844361 2.08925157 3.106912437 3.497211859 2.818382186 3.607279637 3.267835007 3.410864561 2.517655789 2.999391904 2.453990845 2.453581681 3.610479638 3.765484134 3.735377183 2.804200011 2.315798529 4.437182433 3.502794509 4.073677003 2.068102005 2.936477046 3.130407656 2.986030095 3.071793007 3.437683255 3.673133731 4.123749328 2.899771048 3.695432014 3.430751402 3.115249427 3.790750077 3.527871086 3.585466621 3.0424812 3.643482348 4.003500924 2.423107403 3.50867009 3.952399922 3.446101494 4.057157469 3.449608365 1.778497907 2.498126545 3.961419588 2.610240372 3.522380358 4.011563906 2.34406593 3.387435113 4.077637492 3.744311895 3.554363558 3.142806511 3.718655024 4.36594108 3.781504152 3.504403398 3.402606233 2.781202746 3.930463487 3.388690236 2.690723459 2.269022731 2.433601626 3.382921521 3.004095901 4.029783716 2.890615128 4.052497843 2.638105334 2.980956122 3.961597968 2.974689898 4.007732065 3.206184266 3.266826678 2.859383297 3.292133643 3.678499425 4.358971868 2.907699968 3.890577938 3.873209147 3.11751396 3.227719136 4.126548099 2.126976699 2.502801678 2.824024684 2.806050418 2.943822591 4.280963098 3.352376131 3.348141385 3.013225577 3.107903388 4.050464871 2.14751215 3.024606439 2.803204776 3.212004241 3.386871362 2.192453898 2.851207838 2.257563472 2.562305914 3.253655457 3.335631381 2.772018574 3.911747328 2.862583997 2.397118005 3.426991915 2.014154499 3.798626828 3.87618509 2.55437396 2.478433176 3.387359228 3.197706811 4.047965504 3.736890653 2.19647409 3.024903487 2.510307514 2.483048437 3.09546377 2.483478194 2.616819196 1.544055798 2.209120635 3.292742251 3.190258116 2.592545798 3.713044411 0.986124502 3.734480513 2.26563848 2.707759279 2.537396442 2.575647137 3.597256219 3.101162862 4.068210572 3.914823021 3.536058833 4.126331269 4.070619327 3.42175694 3.92611889 1.889323647 2.499179642 3.459323942 3.407704709 3.203787564 2.784277744 3.305269529 3.461451106 4.134822125 1.723472627 2.43147438 3.671793231 3.366156733 3.442515246 4.739253812 2.878359307 4.142869359 4.223648559 4.323731103 3.484708552 4.820724439 4.037900863 4.166253192 2.287152108 2.487949059 3.711493249 3.312335463 3.736215511 2.728045121 2.947541623 4.188906689 3.269742191 2.248570535 3.226957573 4.300292593 3.71918947 4.54591417 3.900640158 3.197662863 3.326038586 3.111507447 3.598870872 4.201536401 4.643200261 4.422428138 3.273983092 3.994043376 1.710940479 3.676372185 3.109323189 3.573880781 3.878679305 2.068746368 3.039906273 2.70497397 3.359451092 2.460329111 3.775432335 3.002643909 2.265411361 2.395229032 3.301030258 3.282145888 3.70743482 3.063058185 2.500597434 2.923052596 2.30813108 3.166707952 2.372406792 3.928757681 2.892417033 3.452191229 2.487427552 4.323576132 3.502943672 4.411691163 3.204270097 2.567325537 3.922287907 3.674000339 2.668379315 3.859581227 2.821481572 4.91191139 2.251237107 4.370248144 4.187065582 2.17169549 2.864925415 3.566414609 3.115495842 1.930588571 3.475978607 2.0026067 4.724073883 2.973531364 3.893693916 2.52200297 3.633470433 3.26895704 2.523820539 3.477725863 3.208962473 2.693639109 2.546939512 4.079121143 3.382912672 2.040118988 3.598687916 3.769252497 3.612660583 3.87127194 2.874008541 2.996293985 3.308940725 3.120402209 3.381811746 2.523498751 3.241964806 4.339239086 3.892198995 3.165096584 2.574493815 3.604463097 2.973751696 3.011019794 3.384004071 3.736169975 2.942423513 3.334930253 1.992458815 3.41446147 2.956485756 3.324466602 2.829869385 3.708044195 3.511278602 3.351087445 3.144405832 3.267802012 3.352040357 2.909989655 3.818309981 3.588435946 4.717667501 3.096474243 3.232238385 2.815219589 2.538276771 3.186229417 4.190131627 2.690716047 2.948293616 2.571655651 3.291575779 1.963997459 2.448859867 3.963229483 3.540081753 3.877445592 3.71958561 2.366744617 4.342882532 3.534168666 2.992417791 2.5525005 2.970246647 3.250975654 4.295609996 2.651420327 3.522826921 3.231038692 3.829088882 3.364436466 4.422560755 3.241468246 3.360568301 3.015648825 3.439433378 2.779333114 1.359981388 2.65548662 3.633778567 3.037963255 3.343179514 3.587833208 4.044525798 2.997528333 0.951660017 2.949561856 3.026424699 2.895321259 3.932290129 3.432430044 3.593230671 2.680265459 1.790701455 3.155362122 3.552404613 2.811734699 1.822949395 3.064193109 2.871717665 3.264233107 3.8873807 3.032357404 4.21551456 2.362982682 4.282421625 1.58566553 2.578203223 3.613966249 2.3786958 3.097014458 3.634134294 3.099280385 2.421663073 3.744649282 3.289880731 3.321411993 3.051602439 2.846390406 3.52240439 2.443691998 3.319612009 2.761319986 3.325005593 3.45195357 2.688818453 3.463323624 3.775108313 3.475317487 2.838661585 3.095287655 3.810240768 3.772748741 2.968167628 2.662580102 2.622101606 4.261091404 2.959079782 3.107847337 3.058053868 1.946074417

ENSG00000118017.3 A4GNT 0 0.008796249 0 0 0 0.027796648 0.01197822 0 0.028283744 0.013705705 0.041870587 0 0 0 0.01341073 0.039402009 0.014806963 0.043737752 0.07047639 0.027224055 0.024755486 0 0.031982346 0.035732322 0 0.044659135 0.016005675 0.027626734 0 0 0 0 0.013955953 0.011842311 0.011165717 0.044008789 0.025624796 0 0.028697516 0 0.080242492 0 0 0.043141667 0 0.015176651 0.038501893 0.03271084 0.024970159 0.029747754 0 0.060618645 0.01764228 0.08946155 0.015601846 0.016554158 0 0 0 0 0 0 0.086786361 0 0 0.015034086 0 0.035252304 0.013023362 0.02369961 0.014642338 0 0 0 0.020064916 0 0.015663633 0.007932178 0.100580876 0.046169631 0 0.030589138 0.02525404 0.012990897 0.012120672 0 0 0.048931625 0 0.02714809 0 0.020103319 0 0.120310126 0 0.015115098 0.049164695 0.051786689 0 0.060685803 0 0.008828816 0.008941121 0 0.016094285 0 0 0.026090431 0.057837069 0 0.035670728 0.009149385 0.021002166 0.117775966 0 0.031869278 0.011065879 0.008583734 0 0.029483219 0.08063411 0.014311702 0.023702097 0.025283202 0 0 0.01577989 0.01130761 0.012400001 0.040773393 0.031969197 0 0 0.020583757 0 0.021787116 0.077533776 0.024591169 0 0 0.045750622 0.029541077 0 0.011619873 0.024767745 0.035742072 0.022576223 0.037023646 0.010923988 0 0.060200277 0.058596753 0 0 0 0.01259591 0.013247432 0 0 0.01223656 0.012880652 0 0 0.043942113 0 0 0.039614906 0.055326462 0.022112937 0 0 0 0 0 0.027466152 0 0.017920758 0.041781856 0.016321303 0.015302977 0.014558383 0.015071071 0.041256927 0.015553937 0.079689112 0.036653772 0 0.052937045 0 0 0.022854849 0 0.026557699 0.017252638 0.014641133 0 0.066726497 0 0.017715352 0.011917051 0 0.026282292 0 0 0.037269725 0 0 0.102201311 0 0.037539021 0.024566514 0 0.013411419 0.023346235 0 0 0 0.01219758 0 0 0 0.013294404 0 0.013698715 0.012983897 0.039333988 0 0.043070563 0.012329494 0.014504925 0 0 0.012505576 0.031541025 0 0 0.010375308 0.020244856 0.058678343 0.01339525 0 0 0.015142104 0.012513733 0.084663441 0 0 0 0 0.232619057 0.030367335 0.196533956 0.028935968 0.011631276 0 0 0.040370882 0 0 0 0 0.04846633 0 0.011032446 0.574418182 0 0 0.016029395 0 0.085635909 0.031613936 0 0 0.016852163 0.036643638 0 0.01347796 0.039300836 0 0.017310615 0.014762573 0.014561607 0.334022974 0.012518802 0.123617384 0.047501071 0.011965154 0.082749918 0.100900592 0 0.032477122 0 0.033250732 0 0 0.047504646 0.029168384 0.015713051 0.014498515 0.04261336 0.017173188 0 0.016053803 0 0.014938249 0.020362337 0.018436863 0.083970908 0.058133381 0.076721095 0 0 0.017417028 0.011632996 0.03173548 0 0.014077477 0 0.01750174 0 0.020743991 0 0 0.031098489 0.017154974 0 0 0.027166135 0 0.039151395 0.027690646 0.044859226 0.017581617 0 0.011694778 0.016146573 0 0 0 0 0.047555685 0.019962091 0 0 0 0.033998162 0 0 0.020893762 0.014872317 0 0.01794137 0.016269162 0 0.047360058 0 0.02035274 0 0.053863125 0 0 0.064672879 0.103397033 0 0.035104345 0 0.049581192 0 0.026322275 0.015637919 0 0.049917679 0 0.039677267 0 0 0.027838623 0 0.025043165 0 0 0.013109021 0 0.059747457 0.029529385 0.047547782 0 0.019017115 0 0.017656698 0 0 0 0.017666001 0.075130627 0.027763705 0.031111001 0 0 0 0 0.016318489 0.027549715 0 0 0 0 0.039003433 0 0 0 0 0.012491321 0.019549703 0.032909081 0.046325124 0.052477971 0.018763252 0 0.073314549 0 0.052093997 0.03333465 0.035637543 0 0.027331323 0.012198956 0 0 0.029839029 0.013135912 0.045537043 0 0 0 0 0.015171288 0.024387819 0.017994287 0.017750777 0.07083565 0 0.020183714 0 0 0 0 0.042915613 0.021211122 0.015993106 0.017221952 0 0 0 0.019807952 0 0.042831017 0 0.021414251 0.013601583 0.020573866 0.094585184 0.017366302 0.01835158 0.110857377 0 0 0.066045412 0.041869093 0 0.036723455 0 0 0 0 0.020441096 0 0 0.057946222 0.052989355 0.014891015 0 0 0 0.013353512 0.01749433 0.022595215 0.025432391 0.021406426 0.0318758 0.056698611 0 0.015257619 0.04927248 0 0.071710231 0 0.030299318 0.030115924 0.047425553 0 0.08146905 0.031267762 0 0.057352189 0.031890917 0 0.03737773 0.018242118 0.020185222 0 0.087148919 0 0.014917513 0 0 0 0.018202284 0.028615277 0 0.022608673 0.055917222 0.135411077 0 0.028820037 0.029186125 0.015338721 0.023183456 0.054424184 0 0.10067199 0 0.010004319 0 0.019409454 0.024751788 0.01476597 0.016951447 0.226468671 0.033247477 0 0.013963221 0.01611188 0 0 0 0.025608918 0.028778179

ENSG00000094914.11 AAAS 3.610039013 3.326270784 3.355066002 3.601849832 3.371366075 2.548410976 2.930956709 3.00325655 3.020300735 3.158577025 3.467342708 3.242291673 2.913599601 3.015217924 3.125329842 2.91478732 2.885338167 2.744128301 3.137956116 3.144545546 2.862735422 3.194280965 3.288867545 3.284966029 3.1676699 3.10304807 3.260024566 3.385948827 3.542216435 3.343017534 3.413495574 3.597999202 2.904150394 3.456733915 3.737319878 3.221130955 3.368253818 3.092682571 3.326028241 2.854815324 2.982408687 3.110108824 3.377146081 2.902737371 2.923522367 3.234092373 3.268922143 2.969205133 3.470342331 3.634825163 3.37771276 3.58013816 3.678745581 3.654802153 3.750330581 3.53414355 3.706041192 3.622818938 3.334807575 3.767217864 3.654813033 3.811565978 3.0868483 3.381872725 3.331782773 3.333227949 3.667985192 3.811207775 3.317993187 3.536637227 3.332314885 3.529252462 3.519939067 3.341107413 3.078013514 3.728538285 3.329754721 3.69530238 3.601745017 3.73172405 3.079648149 3.53972819 3.605294816 3.270859133 3.107492127 3.625820336 3.591316535 3.3429561 3.587295235 3.518936217 3.164213444 3.548663196 3.395253736 3.562797404 3.541690021 3.54298507 3.300109096 3.476477742 3.1820458 3.074188393 3.564403644 3.486263102 3.399057073 3.601759274 3.304134382 3.384192905 3.242471618 3.528082983 3.208745877 3.469943137 3.476143059 3.516282339 3.545408434 3.068698461 2.972548869 2.960216282 3.329944007 3.391740945 3.298066456 3.296396248 3.24510673 3.179504072 3.141883311 3.540838065 3.158015707 3.469933856 3.448561937 3.402482061 3.298758003 3.317906667 3.519237783 3.350387549 3.687241741 3.820102218 3.714613095 3.591522401 3.326344755 3.839322032 3.666414607 3.623171144 3.561653516 3.566868099 3.316821579 3.210162934 3.378105264 3.511309 3.493145642 3.339016713 3.202769143 3.263159165 3.4227404 3.664252297 3.198628924 3.304707616 3.469009991 3.291797295 3.49123017 3.446610399 3.632881915 3.659097932 3.736432857 3.502229183 3.788522592 3.892560396 3.945263187 3.637537108 4.124041102 3.385133185 3.747849258 3.527169346 4.323650374 3.602571886 3.636292649 3.642744005 3.418785543 3.655020552 3.792866292 3.568219387 3.951094919 3.718389863 3.942645719 3.552995551 3.800233575 4.087101228 4.01580704 3.670638878 3.548119421 3.353920702 3.69138801 3.514282046 3.476125294 3.524159293 3.314606596 3.455922966 3.606964014 3.484101556 3.80857238 3.725164847 3.896877955 3.475648032 4.015586911 3.425043606 3.493073458 3.243837953 3.637048389 3.68238698 3.484614901 3.267276551 3.556242859 3.550681108 3.523995301 3.628293194 3.617288053 3.490482942 3.311768482 3.183604414 3.388186657 3.499421342 3.215713097 3.322380242 3.552854398 3.369977223 3.722779346 3.690939238 3.448963408 3.269217159 3.784772598 3.498825139 3.518729775 3.477295463 4.071891917 3.200454841 3.492831376 3.555890696 3.413589512 3.529339373 3.112940152 3.355502419 3.068794213 3.509940475 3.683813716 3.867293689 3.867118054 3.535577862 3.483703781 3.762569635 3.681959317 3.698301151 3.662828157 3.58082613 4.069236481 3.147097644 3.218915251 3.066224907 3.376550606 3.429246219 3.96747504 3.058088966 3.471407115 3.335846658 3.285473973 3.450780744 3.304490702 3.617705086 3.091987795 3.584813328 3.443414668 3.618252135 3.525412072 3.370716504 3.620511669 3.333551341 3.414679006 3.38803477 3.516135583 3.668065759 3.871241294 3.449442207 3.528777653 3.14442893 3.550729475 3.674225049 2.571231984 3.700767017 3.770012732 3.75253254 3.709618158 4.019784986 2.857459369 3.706533217 3.780312582 3.760981374 3.506241876 3.60020162 3.696491229 4.001767567 3.641198939 3.752363285 3.921916009 3.726352883 3.802807367 4.151930157 3.245831748 3.91116614 3.683094792 3.746570252 3.525958051 4.159556617 3.426627365 3.443631475 2.974314034 3.265473202 3.578935976 3.577592362 3.226129568 3.794957693 3.536133182 3.577862909 4.003964637 3.091973424 3.606645313 3.63815771 4.009714883 3.567439499 3.629420368 3.611125975 3.764347754 3.733426202 3.655095927 3.833995962 3.583197734 3.59231004 3.752059464 3.545729566 3.233506774 3.490041822 3.486703598 3.481068293 3.952601106 3.686205195 3.582771824 4.259833819 3.453704313 3.507786602 3.620003086 3.70515345 3.549312088 3.37504153 3.304228658 3.631449275 4.085750021 3.919680147 3.851493641 3.721246125 3.657261059 3.91236327 3.714873343 3.617259079 3.160216079 3.259874121 3.797432488 3.648778661 3.48367019 3.596935246 3.455808128 3.802389308 3.672077491 3.350976506 3.869597548 3.496908442 3.905668462 3.85102127 4.157612103 3.899834616 3.815836784 3.683045153 4.239602182 3.891981276 3.934274828 3.754893391 3.960264428 3.767888732 3.375447448 3.365648085 3.807929828 3.497177976 3.900747371 3.700384295 3.624683902 3.699870418 3.483161366 4.010227897 3.646512053 3.372145569 3.934653141 3.733291612 3.423668751 4.03740875 3.416993206 3.565132795 3.822793579 4.195121356 3.609923979 3.578207493 3.889730263 3.481797062 4.147362699 3.533932204 3.668259029 3.555091689 3.611594334 3.973372146 3.807734126 3.804100162 3.66414722 3.070698247 3.418870259 4.031315557 3.287907974 3.948173549 3.643537871 3.588082551 3.945874909 3.759667762 3.704599775 3.510370488 3.851214506 3.155158615 3.278763195 3.738964213 3.526880257 3.437879751 3.580074709 3.467737673 3.741391942 3.360514138 3.829422102 3.491083271 3.97681246 3.850616032 3.849833555 3.701799364 3.526298777 3.756402437 3.569187448 3.552228717 3.589596051 3.955302854 3.665498033 3.531401678 3.520364014 3.757669189 3.910436638 3.684584846 3.554662951 3.698609112 3.778469021 3.857205187 3.866642232 3.808753929 3.724696228 3.637511332 3.64755326 3.708185228 3.424557044 3.574960373 3.124524403 4.089031584 3.49188023 4.138325002 3.52411968 3.983629394 3.544612077 3.824010647 3.467070016 3.945490541 3.930018709 3.4400996 3.824431268 3.859375772 3.871054755 3.843900361 3.683320462 3.828670419 3.640822565 3.834139534 3.942636079 3.709662186 3.611734194 3.478278101 3.86318141 4.063627515 3.472700611 3.653439851 3.516149831 3.737042322 3.75775864 3.318905925 3.624114092 3.85722378 3.902939655 3.667984604 3.808931898 3.642542662 3.695204061 3.572362286 3.634856202 3.759956212 3.579168085 3.664530501 3.749735576 4.072062686 3.908511826 3.764816211 3.923013598 3.727825674 3.888560704 3.751213496 3.63957566 3.512349493 3.692019411 3.709650197 3.71683632 3.550328163 3.50239556 3.402903598 3.693733929 3.084198145 3.882085587 3.245228075 3.40024781 3.407159101 3.034259967 3.265941237 3.89113378 3.450434242 3.3001019 3.483181801 3.414919924 3.888402155 3.494673828 3.763719413 3.392648523 3.380593246 3.617397785 3.556147672 3.237465954 3.794915388

ENSG00000081760.15 AACS 2.279713208 1.720807262 1.758331175 2.538050652 1.981523449 1.485335762 1.902833146 2.113061101 1.562245181 2.419436058 1.990860831 2.340689023 1.438056331 1.423127654 2.042643577 1.391175636 1.696902647 1.235644872 1.896579481 1.824001578 1.781073635 1.923651546 1.783253212 1.729505571 1.720376964 1.979395962 1.511598 1.490292392 2.269770693 1.430295704 2.055529225 1.570353093 1.619639418 2.366451692 1.518373338 1.959913988 1.506320472 1.278041178 1.77807281 1.741000955 1.830487366 1.55373267 2.066920706 1.467754127 1.86431688 1.624892945 2.050995163 1.537407506 1.792795961 1.982040995 1.421779966 1.721302147 1.937228335 1.984281192 2.310792142 1.997557769 1.811862207 1.871500234 1.637200075 2.239066652 1.659481129 2.258729116 1.358781434 2.28543988 2.121152044 2.242951011 2.670703447 2.451763963 1.720738377 2.252446062 1.996942032 2.241676592 2.38860468 2.435801092 1.824292565 2.836926317 1.632165612 2.496617348 3.394480299 2.14634082 1.939264531 1.881077685 2.002559342 2.445521509 2.738826801 2.36131913 2.462250596 2.011475127 2.064997863 2.750063046 1.99394833 1.465621955 1.446656923 2.208490776 2.355113034 2.062765952 1.468393264 2.271238904 2.404274704 1.884220467 2.270290251 2.675736786 2.185147735 2.402581995 2.392383254 2.523056434 1.91249043 2.120875814 1.834767681 2.570013385 2.435695693 2.224237061 2.025874945 2.445770917 1.994089371 1.991935374 2.035707562 2.383988499 2.87681775 2.039778545 2.095125721 1.807881843 1.696904104 1.3049611 1.886062516 2.241150313 2.258317335 2.620026087 2.434948544 2.297363398 2.803234829 2.35045465 2.567230419 2.628771311 2.508252238 1.772030952 2.619429096 2.161634669 2.114627854 1.930082262 2.141040847 1.667345927 2.269895261 2.377094652 1.901219201 2.293126993 2.009314293 2.050256311 2.081839442 2.219485204 2.246273197 1.89830448 2.158835036 2.24726867 2.024109006 2.364107158 2.210690802 1.861351022 2.127553424 2.106310195 1.669020546 1.98650249 2.346776258 2.133536746 1.778768567 2.119801518 2.038938785 1.417162221 2.085060459 2.615694374 2.337597986 2.322320302 2.49630648 2.273314668 2.399900398 2.273134525 2.092632391 1.941542512 2.138848934 1.923259505 1.936203208 2.238361767 1.997965141 1.928967117 2.234060294 1.83635467 2.233763581 1.960237573 2.313359461 2.152943392 1.33839572 2.137249134 2.196575472 2.253276336 2.195524025 2.130211315 2.068934205 2.296411275 2.026916482 1.668433993 2.42555775 2.024658821 1.973042707 1.572888631 1.855077474 2.300952349 2.166736077 1.950734139 2.004528345 2.255492877 1.915484257 1.9938292 1.972652966 1.717208886 1.493272987 2.322705035 2.327759036 1.932418076 2.430957996 1.917972833 1.932658771 1.828993997 2.454517056 1.466069647 1.968204445 1.814265937 1.771083306 2.126169 2.050729544 1.834449407 1.273145187 1.198292427 2.299545556 2.140394921 2.036727243 2.192992012 1.317046283 2.254058272 1.426208189 2.371754673 2.703656811 1.938320988 2.205028269 2.113504028 1.778870635 2.298357475 2.338713752 2.695542688 1.761043351 1.753511719 2.166761107 2.401901548 2.577010318 2.099932526 2.121649834 2.469856973 1.508708656 2.245761199 2.247854871 2.187789239 2.262999032 1.298339286 2.190827142 2.546239538 1.996088445 1.498764097 2.607466978 1.792877847 2.052893302 2.245391301 2.246563517 2.169632439 1.384955232 2.389076898 2.156482771 2.479077853 2.252702165 2.234001407 2.254471283 1.801822151 2.690721258 2.14743877 1.448717027 2.26925535 1.965012599 2.233510815 2.115692968 1.935786157 1.697784961 2.004893686 2.3823543 1.040800948 2.038081115 1.938641951 2.449959151 2.452075792 2.556450363 2.55475148 2.140021925 2.231018296 1.993958749 2.065267347 1.663185702 2.550843482 1.622891329 1.888021449 1.940756518 1.527494578 1.751590792 1.224833103 1.013775044 1.773036314 1.648820965 2.036040953 2.229811391 2.036124438 2.274100438 2.055655214 1.762138344 1.737108733 1.542999647 2.08233693 2.327056191 2.039529261 1.87349439 2.363441508 2.207523648 1.884827382 1.961547555 2.082021862 2.135839869 2.079396389 2.288594444 1.832934259 2.200376993 2.256972974 2.007236468 1.696317922 2.439287662 1.888516274 1.950607215 2.110811571 2.229723138 1.465189458 1.378666396 2.015255043 2.266929522 1.754615411 1.770237022 2.210827522 2.241944015 2.300379023 1.512581823 2.473174841 2.048718429 1.636076776 2.264630678 2.428391985 1.569897894 1.92364925 1.897936345 2.680528624 2.187521246 1.753437415 1.94384184 1.989078591 2.414413455 2.103515565 2.690054948 2.327323505 1.559921742 2.250705828 2.420578556 2.073653428 1.73301564 1.584831752 3.0023464 1.997437512 2.037752856 1.682102068 1.920620125 2.049590991 1.657940959 2.245641565 2.256573785 1.838868237 1.987994102 1.91227331 1.962528296 1.648710122 1.841115427 1.781238306 1.778652873 1.85409302 1.807362796 2.632774678 2.246969972 0.771144489 1.877458273 2.155692768 1.787945269 2.42151969 1.939389363 2.604389471 1.85648469 1.671991657 2.44178478 2.265025939 1.572412931 1.853059526 1.922007838 1.758720671 2.113103634 2.145009794 2.187313466 1.415388426 1.949979478 2.748824767 1.187812358 1.853580983 2.082404326 1.96833506 2.304684556 2.042467585 1.935630725 1.889633029 1.786221416 2.109094103 1.95448054 2.323660618 1.969701781 1.909169547 1.81849814 1.754329432 2.024161736 2.632750014 2.098163815 2.19928204 1.150243465 1.683638432 2.226041253 2.870474888 1.92123957 2.297874772 2.237301549 1.895423129 2.547847209 1.776296667 1.532991716 1.411908977 1.78217061 1.860069119 1.958479738 1.980789147 1.701092017 1.849028728 2.222101123 2.625566685 2.391535302 1.737638999 2.117039928 2.360911171 2.00565225 1.963143088 2.007514446 2.275240578 1.880897583 1.855935662 2.294774066 2.02367499 1.63330722 1.618083913 1.327301401 1.111789634 1.930138643 1.861273883 1.84354772 1.977148386 1.619647376 1.808053001 1.736097282 2.037760418 1.973651059 2.706668776 2.85738715 2.227269122 2.185732164 2.115641241 2.54195777 1.565267231 2.13917943 2.426685045 2.224181124 1.928832482 2.428164633 2.141105035 1.954261018 1.602993991 1.794845032 1.841587607 1.931968618 1.698431486 2.339157752 1.904513527 2.535388209 1.887274873 1.53573562 2.248475349 2.255095531 1.6491592 1.694713113 2.604110588 2.220427898 2.159192265 2.104552948 2.198294489 2.926658001 2.038681252 1.799984251 1.726826231 2.339905278 2.017389964 1.940020457 2.605233448 1.484068943 1.40450465 1.864160977 1.820192383 2.040412456 1.70128868 2.431208071 1.982163265 1.726584472 1.754073072 1.286749153 1.73172456 1.585668287 1.974614322 1.926316901 1.802575234 2.323393397 1.874164751 1.71861443 1.65755098 1.552519243 2.329772479 1.879791882 2.048903118

ENSG00000204518.2 AADACL4 0 0.019707003 0 0.090334594 0 0.054197094 0.013461909 0.019442565 0.031765017 0.030641761 0 0.013471909 0.124184734 0.032898748 0 0.014894824 0.081339998 0.14252367 0.026820581 0.015369139 0.013970322 0.013903836 0.018068356 0.040117724 0.044269066 0 0.154323068 0.015597417 0.030827246 0 0 0 0 0.065351363 0.120834776 0.016652831 0.042960571 0.064085056 0.079261842 0.01424185 0.018438596 0.155241117 0 0.064207141 0.046855403 0 0 0.228859604 0.04186944 0.016800268 0 0.017299508 0 0.02571421 0 0 0.037248504 0 0.067246206 0 0.079535591 0.018477945 0.033143488 0 0.028434196 0.016894081 0.025712593 0 0.057673089 0 0.032722737 0.027732727 0 0 0 0.014417381 0.01760104 0.008916249 0.014567861 0.05181317 0 0 0.014252745 0.029052516 0 0 0.054615302 0.027714388 0 0 0.038387278 0.04482302 0.024898613 0.104637289 0.037398673 0 0.027847371 0 0.017486113 0 0.029203514 0.048949867 0.049563847 0 0 0.014926812 0 0.029304475 0 0.026178771 0.040048674 0 0 0.015251138 0.017227334 0 0 0.009648368 0 0 0.03455177 0.016082821 0.052766803 0.042391253 0.0238551 0 0.052554067 0.012708599 0 0.030672572 0 0 0.012602474 0.023124865 0 0.024475526 0.014851239 0.013877248 0.017875097 0.123897823 0 0.217643628 0.013490628 0.038828745 0.041531996 0 0.025361151 0 0.012277649 0 0.027387993 0.15901814 0.028978881 0.055042849 0 0.028173922 0.044209462 0.031073043 0 0.02737434 0 0 0 0.024869728 0.116050395 0.021018651 0 0.062065421 0.14300966 0.019234755 0 0 0 0 0 0 0.020135403 0.092318818 0.01833954 0.084001019 0.048533322 0 0 0 0.036387997 0.081160035 0 0.020069858 0.017398176 0.045496834 0 0 0.059053167 0.019385269 0 0.018782271 0.037894158 0.021119455 0.039538681 0 0 0 0.038620485 0.14427095 0.021072224 0.03023725 0 0.141670711 0.012713097 0 0.027594618 0 0.015071706 0 0 0.11766145 0.035952896 0.093330495 0 0 0.014987865 0.01494028 0 0 0 0 0 0.012238104 0.02758115 0 0 0 0 0.017817848 0.013138666 0.013583532 0.023229004 0.011417049 0.091324554 0.015053546 0.02897109 0.016188847 0 0 0.131123885 0 0 0 0.108605341 0.017631522 0.011457149 0 0 0.038866493 0.036565469 0 0.01526427 0 0.030090591 0 0.015093593 0.032876601 0.01565756 0 0.095914247 0.015432476 0.015178074 0.087890916 0 0 0.047141943 0.068136002 0 0.07429542 0.013843855 0 0.015146442 0 0 0.112953013 0 0 0 0 0.101866486 0 0 0 0.112977386 0 0 0.026628252 0.055647055 0 0.031544362 0 0.032757311 0.08619797 0.016292622 0.047829363 0.019296064 0 0 0 0 0.045395567 0 0 0.077901541 0.043635237 0.088301825 0 0 0.013074116 0.017928223 0.016748877 0.092416674 0.032684786 0.077100932 0.110465113 0 0.019011209 0.013967871 0.085769513 0.075603777 0.021459705 0 0 0.050501009 0.043950064 0.01563365 0.016977748 0 0.100134235 0.026168373 0.018143339 0.066420838 0.077966964 0 0.088552022 0.121581202 0.044510791 0.086816018 0.019075052 0 0.056888087 0.022799719 0.088928904 0 0.016712413 0 0.078997311 0.018280993 0.015582055 0.053146447 0.021752751 0 0.023247809 0.099350749 0.038701528 0.050362559 0 0.019950066 0 0 0 0.037326518 0 0.014858025 0 0.015957842 0.018912824 0.032422104 0.044539401 0 0 0 0 0.055720912 0.021252607 0.015237879 0.04375242 0.016646911 0 0.065579265 0.070712557 0.046328282 0.042420672 0.014619271 0.039408752 0.08131334 0.015769411 0.051839085 0.03942936 0.056684115 0.03118166 0 0 0.075410632 0.036781926 0 0 0 0.019703135 0 0.041266631 0 0.014742886 0 0.256567728 0 0 0 0 0 0 0 0 0 0 0.03579271 0 0 0.020144441 0 0.030696611 0.013709857 0 0.018419154 0 0.043841224 0.025778582 0.031284157 0 0 0.02443496 0.033897207 0.054277725 0.020217955 0 0 0.096922344 0 0 0 0 0.030375451 0.024285016 0.070337914 0.017971011 0.019350815 0 0.036520588 0.036130019 0 0 0 0 0.071000089 0.015285288 0.045863046 0 0.038765378 0 0.018391871 0.020532895 0.098796962 0 0 0 0.041228769 0.02405212 0 0.045658761 0.020820049 0.022964732 0 0.025101693 0.064997151 0 0.033274961 0 0.035894138 0 0 0 0.025382465 0.014353788 0.024048251 0.070720884 0.042710696 0 0 0.073256417 0.024494852 0 0.014910646 0.017113191 0.066865461 0 0 0 0 0 0 0 0.015601887 0.062494236 0.040705276 0.022677516 0 0 0.119573251 0 0 0 0.017264477 0 0.047940832 0.018154592 0 0.062726586 0.098169938 0.052171944 0 0 0.051102804 0.03888973 0.157401684 0.058609119 0.016652541 0.045361532 0 0.039684126 0 0 0.065256717 0 0.131667977 0.073721987 0.035157694 0.015691449 0.035984387 0.065688068 0 0 0.084627354 0.010853858

ENSG00000109576.12 AADAT 2.519827247 2.400553376 2.497370698 2.510952972 2.771850843 2.184366675 3.000668162 2.534986735 2.47603825 3.211389945 2.879578855 3.421607437 2.273949401 2.010201279 2.932757482 2.475580024 3.041249392 2.149884708 2.894193359 3.433807736 2.575590291 3.394609143 2.646236436 2.817347591 2.21364399 3.106232041 2.12442039 1.965695366 2.955194348 2.12120838 2.888820157 2.565008732 3.28096909 2.857616843 2.501310302 2.56993795 2.64601274 1.99646126 0.33638444 0.671102951 0.964533848 0.885190302 2.676443707 1.245235893 0.522084765 2.270871763 2.892848059 0.489728316 3.529625688 3.066398219 2.141958723 2.72298773 3.766499443 3.020883144 2.567717472 3.585466538 3.579110844 4.349479835 5.457841285 3.100338639 3.087692406 3.829775376 2.229479483 2.771492389 3.922744368 2.891903714 3.099671523 3.608575363 1.984625204 3.658509094 2.668426878 3.866134444 3.689938247 3.763357063 2.888828896 2.597345025 3.535690092 2.307479334 2.502081319 2.857341453 3.456375791 2.948965751 3.112364217 2.572702873 2.94418216 2.699090054 2.462528222 3.508981421 3.811337591 3.840761749 3.058326529 2.324757082 2.966317 2.066087586 3.611227716 1.753358179 2.675968245 3.964656777 3.464383254 2.890414271 2.849371439 3.563616433 2.896251763 2.786453076 2.902663204 4.465283937 3.286585007 2.830803403 3.479978811 2.592365609 4.538773694 3.038322923 3.169522835 2.735488165 3.147915051 3.26682869 4.209583859 2.007404071 4.797904606 1.782798365 3.713593928 2.801202571 2.30633783 4.520869689 2.928933126 3.260263083 2.405778753 3.132476652 5.013596391 3.430392359 2.917204212 2.386723322 3.223313421 3.191534563 2.988818223 1.544221162 3.089425276 4.11018377 2.457414028 2.127866539 4.210604892 3.082787719 2.845946407 3.234562015 3.091727968 3.282108562 2.576777745 4.217664339 3.474407796 3.41964737 4.533594733 3.675555145 2.896522219 2.459398093 3.246194095 2.889059567 3.266949727 4.746708528 2.568062174 3.64703596 3.513003091 4.453293738 3.315774263 3.585656356 2.659834826 3.589937931 2.927412529 2.906016471 3.071275717 1.670902117 2.851231024 5.431735804 3.756082167 3.807614822 2.324234282 3.992291328 2.762271378 2.780609713 3.795252621 3.084391422 3.360805562 3.896626733 3.085505107 4.136574069 3.348193157 2.928225386 3.379013361 2.873454822 4.873502679 3.521873194 1.950437059 3.305080183 3.904353395 4.116504775 2.239016578 4.641664089 2.784876436 1.982911179 3.039601998 2.46046304 3.416356612 3.896027902 2.821859713 2.050549097 2.586473672 2.489552738 2.609857729 1.097192508 2.508533786 4.088400722 2.690084176 3.257798792 2.846422224 2.329716287 3.14015121 2.987131906 4.081674935 3.375342772 2.986233938 2.532446736 3.177920977 2.37292737 3.558950377 1.814167306 2.539104148 2.265897031 1.899576382 2.809153251 2.660688739 3.155783734 3.78333416 1.619498647 2.595223424 3.826861495 2.373258248 3.653122278 2.806220483 3.929677158 1.490654176 1.462968268 1.814966852 2.496838673 3.327526427 3.345657816 2.344565877 3.544918768 3.665827791 2.280631301 2.901824155 1.719543643 4.379905157 2.152019261 2.557439782 2.766499703 2.843044451 2.538536103 2.863403034 1.822557484 3.059758141 2.982157376 2.850459001 2.810671218 2.647033573 3.03340499 3.048945922 2.886023061 1.438235355 2.271999813 2.863007261 3.551646511 4.922242539 2.765575564 1.648492751 3.264050075 3.352706281 3.274267137 3.478795703 4.852813158 3.915114774 4.156333959 4.551862629 3.319895421 3.042657327 4.912847731 3.68504801 4.802257125 3.419198113 2.897879962 2.310191505 2.988999817 2.798679678 3.405493564 2.364161105 2.983315251 2.988701518 2.858262605 1.594670656 2.266780025 3.588441748 3.041024636 2.509774437 2.877520839 3.447828249 4.100055202 5.342050775 2.547743384 3.906718473 1.755585915 3.588177919 3.958636662 1.776559169 1.659705452 2.504821964 3.908401309 3.307309484 4.079911963 2.937054062 2.698284442 2.736185099 1.758082168 1.627967192 2.755718689 2.623103865 3.390787388 2.052480789 3.426891108 4.690786464 3.909367343 3.064486972 3.15367308 3.311554731 4.232171097 3.205394525 2.79554244 2.64191832 3.227238421 2.116269774 2.846090485 4.098984994 3.332295666 2.467995858 4.616151184 1.920465058 3.915740306 3.651956686 2.574900784 3.758254147 2.730488364 4.481369849 2.652609088 3.062458783 3.040217927 3.31627104 2.904776136 2.860986235 4.908068755 2.481100297 3.205132812 3.707171329 4.471747029 2.961313254 5.0460837 2.398995119 3.825843994 2.480309362 3.278221637 3.255687155 4.505871455 2.319819083 2.74505961 4.946782916 2.700621047 3.940136928 2.891264526 1.514763139 3.587342729 3.995945302 3.790354694 4.113219804 3.38841783 4.303683881 2.626063112 3.206148182 2.582693968 2.779526515 2.758338569 3.117896346 3.007111103 3.634444568 1.802116604 2.033519304 3.095723773 2.634449296 2.653519981 2.820857758 2.685112816 4.806114044 1.352573662 2.433380126 3.547165962 4.138852296 1.968169466 2.841552774 2.701999579 1.735162741 3.108883772 3.385940357 3.210959027 3.428855343 2.483684025 3.045773316 1.044378178 1.937751914 3.507764125 4.030227699 2.243379878 2.873285046 2.86316839 2.445091392 3.337061064 3.440218268 3.742610701 2.068321526 3.912248689 3.425186031 3.622551501 3.625347995 2.383561511 2.906279129 4.017621196 4.203315187 1.71847725 3.015822156 2.429703837 4.066180222 3.119214936 2.380700104 2.495362872 3.27043902 2.871413442 3.525637283 2.507723794 2.190808628 2.564717403 3.495989799 2.380371364 3.605139747 3.891334493 2.409145997 3.440199095 3.623204016 4.078248271 5.317418401 4.06955891 3.053360645 3.345784428 4.303302982 4.381454491 1.188594889 3.633744757 3.436953088 3.487750182 2.998762208 4.287375757 3.434115743 4.413063876 2.142534326 3.182291217 4.195744191 4.555918246 1.657432762 2.381334063 3.882401613 3.316364109 2.865908912 2.170308852 3.250145289 3.179261165 2.806871084 2.654736207 4.495366536 4.055861356 3.079575567 2.551560021 4.807820262 4.589612072 2.910274989 3.410886375 3.701691513 1.67353269 2.825423889 3.121930475 3.332939707 2.093596125 3.354462682 2.535748764 3.020348818 3.419050998 1.738642748 2.638831747 5.307915842 2.548241914 3.324871326 2.31676339 2.504382642 3.41687024 4.864251878 3.088834828 3.217320131 3.476179821 3.104853952 3.549171982 4.364161099 3.20746133 2.860613574 2.861204957 3.072124359 2.778418357 3.865856994 2.565012405 2.568281527 2.691781988 3.121197732 2.332609293 3.121321347 4.263456132 3.05025582 3.990328354 3.883341606 5.184559569 3.333152265 3.973657944 4.572597345 2.857504053 2.208215621 1.737124988 3.149256975 3.993506942 2.148355483 2.001976357 4.60776914 2.07468351 1.971712063 1.844993302 1.840211047 4.049064691 1.610455142 4.875793042

ENSG00000103591.11 AAGAB 3.815293374 3.685713254 3.826849215 3.885164316 3.898454649 3.227789065 4.26342481 3.711878109 3.703661831 3.964937822 3.753491196 3.95479259 3.314849715 3.67965833 3.678720497 3.467208068 4.250337035 3.5206602 3.83993599 4.002589829 3.578626368 4.157686591 3.867273389 3.852429528 3.675786205 4.070472827 3.492820814 3.556346255 3.800617305 3.221996756 3.607171484 4.00557381 3.350860135 3.765289436 3.442548684 3.612887371 3.417859706 3.355433871 3.792983669 3.704614143 3.710126709 3.735960939 4.002439712 3.432201234 3.713666177 3.135622756 3.450315161 3.918437464 4.116946104 3.804362998 4.094492699 3.445449881 3.989919111 3.713566138 4.585199411 3.964419381 3.18725275 4.242863852 3.79150515 4.189122924 3.978686915 4.338588681 3.659968247 3.792319631 3.738341018 3.985895481 3.712668723 3.842314833 3.736657706 4.171915608 3.589578337 3.956688227 3.926219804 4.042311107 3.775677191 3.911501261 3.829827987 4.150076718 4.344572478 3.883950331 3.830186402 4.02655607 4.064431224 4.44378214 4.076330884 3.871663736 4.078147959 3.579009401 3.9327938 3.93273262 4.111220266 3.868109667 4.366042492 3.852272428 4.033940559 3.629052827 3.777519377 4.174810685 3.870264971 3.876744637 4.153519405 3.869256689 3.893293271 3.959003542 3.908877469 4.04289489 4.164161819 4.307637926 3.976497497 3.500578109 3.951357769 3.816863575 3.972931101 4.728047278 4.164757093 4.155983167 3.982144429 4.207315822 3.801305993 4.137659182 4.098297865 4.260571783 4.503512853 3.922614179 4.101936784 3.935340231 3.795613376 3.871928988 4.054712014 3.662150787 3.73063619 3.766912621 3.739285355 3.98844422 3.805546459 4.21320847 3.678392235 3.984209096 3.685891655 3.34248997 3.739838787 3.582870168 3.898858031 3.806870462 3.854103683 3.847776787 3.416975783 3.559446188 3.70703595 3.882125136 3.828484089 3.900946259 3.742977593 3.638505943 3.745435152 3.772462172 4.083136619 4.072598508 4.370088059 4.340610591 4.389564704 3.722571535 3.80766424 3.799589678 3.260642701 3.858166055 3.309469405 3.674812818 3.602550231 4.592112251 3.863832299 4.325914708 3.947124936 3.752255761 3.960573725 4.116702241 4.145813918 3.79444879 3.892545289 4.040985895 3.712472442 4.195855341 3.908844954 3.849216457 4.196429516 3.804375079 3.958750437 3.915633325 4.002766576 3.902526106 3.211504336 4.135207108 3.853995972 4.386933491 3.892500325 4.192953985 3.84819505 3.887857345 3.60544567 3.721042155 4.216058728 3.745721076 4.114002125 3.368069441 3.995596766 4.011688123 3.664608857 3.342732163 3.725845174 3.806121786 3.705475883 3.905147206 3.790981682 3.701701796 3.286919361 4.466815386 3.367573725 3.567409853 4.360184518 3.760101272 3.47096467 3.626188827 3.966373101 3.87343938 3.869169749 3.54232922 3.865052796 4.000897467 3.639295665 3.810988326 3.681052121 3.301183671 3.882525043 3.994213373 3.913679671 3.848659801 4.143553549 4.160408814 3.361656916 4.226374999 5.028904172 3.147970865 3.900401463 4.169442705 3.52287659 3.610517877 3.959055019 4.040775341 3.406173077 3.556061895 4.192862242 3.714955141 3.927340884 3.771124272 3.96911946 4.119631924 4.05414041 3.982597907 3.986636701 4.170634363 3.856293796 4.497306331 3.608884054 3.961055195 3.576697554 4.373410449 3.832046746 3.412538413 3.704110352 4.157355676 3.899991807 3.487250142 2.869242733 4.286693788 4.002495346 3.805649784 4.05152612 3.701660106 3.908796927 4.242260774 4.158714073 3.920845279 3.616063355 4.104301078 3.950462065 3.848125553 3.966225489 3.88732768 3.733436403 4.071616149 4.809927145 3.346282101 4.235683793 4.001057605 4.105490308 4.357478473 4.082226229 3.865967264 3.933150587 3.814623711 3.515362488 4.097845197 3.531391624 3.772156452 3.695458011 3.623980216 3.803905376 3.448927398 4.432554439 2.985289709 2.707249889 3.540833166 3.832839149 3.573832659 4.034631385 3.680629178 4.590494097 4.012153581 4.136745124 3.868680799 3.583452226 3.902406228 4.043237714 3.949279371 3.931621206 4.017144297 3.768885531 3.990145196 3.895495154 3.7949206 3.536787565 3.691212462 3.985750611 4.009982179 3.920642462 3.781447194 4.164075593 3.529725711 4.054290487 3.313447373 3.737215501 3.672254786 3.593024441 3.578147724 4.282870969 3.746567555 3.896197606 3.777985244 3.831642366 4.013300523 3.668348372 3.821330561 3.527591043 4.124932206 3.858198671 3.397606316 4.267697821 4.037908309 3.200157226 3.478225795 4.081984028 3.901499078 4.518501397 3.844810007 3.933271441 4.472026216 4.294744574 4.015196219 4.139772463 3.562078054 3.621400441 3.831423669 4.344657792 4.061090095 3.974797726 4.247036624 4.128629503 3.562620922 3.917782925 3.815580777 3.732337496 4.180744948 3.612976005 4.161818539 4.070260971 4.045930287 4.060812351 4.011193316 4.029051797 3.754872241 4.033125425 3.718770166 3.754092294 3.938641963 4.127837435 4.315866795 4.324511176 3.564747158 4.080638242 4.306056747 3.846803866 3.592586417 3.963067333 3.836884157 3.272574143 3.451273681 4.143209037 4.425366869 4.032244046 3.57974575 3.362882428 3.426746569 3.759129013 3.680247837 4.188475338 3.384490184 3.94916057 3.917519407 3.964459153 3.815480153 3.954409454 3.896369324 3.711662482 3.956543495 4.116157778 3.909593831 3.739717109 3.282823886 4.378076602 3.817923867 3.727172608 3.6422854 4.250788106 3.358222413 3.881374654 3.924571367 4.174828636 3.648422292 3.202503386 3.783149711 4.076848448 4.014955679 4.046592587 3.999922944 4.142733254 3.41955572 4.058991482 3.539832891 3.633172538 4.27549881 3.944725454 3.644521202 3.978087445 3.875976571 4.369706553 4.218078178 4.275835509 3.999501437 4.224562507 3.853821152 3.84452306 3.887134055 3.698806275 3.454255995 3.844932952 4.264357282 3.9077684 3.661635715 4.608363718 3.87395105 4.181537158 3.559508168 3.501843667 3.34616363 3.903489413 3.98679826 4.043440264 4.058697225 3.753276788 3.91533653 3.629414937 3.290329568 3.880813243 3.865353966 4.108103451 3.773120035 3.75263315 3.986279196 4.352041866 3.676715367 4.012297258 4.320015504 3.71949411 3.603667653 3.806915749 4.064278827 3.762533127 3.565706669 4.167781426 4.200176231 4.250138391 3.526336369 3.685875495 3.509822292 4.100295901 3.700458108 4.462274119 3.985823147 3.782217969 3.447342217 3.770033467 3.880624689 3.601410836 4.01099714 3.63383432 3.93439182 3.81752593 3.484892202 3.987348263 3.456827769 4.29399604 4.012655213 4.199165828 4.124354626 3.967728071 3.773119615 4.099659657 3.554815809 3.721513636 3.379097231 3.741409612 4.039086923 3.271298198 3.78963128 4.045058247 3.157607384 3.132054746 3.992699323 3.679112267 3.76238984 4.121513061 4.310787188 3.297423026 4.080064968 4.499324333 4.449723707 3.998704022 4.156051357

ENSG00000115977.17 AAK1 1.968173255 1.836706065 2.361772576 1.896487222 1.844903313 1.754166369 0.931213191 2.302189656 2.036012238 2.606896611 1.884309898 2.272736477 1.793329022 1.493153496 1.799979427 2.094226457 1.536392766 1.281014025 2.454124735 1.509816086 2.282099659 1.999417503 1.829233046 1.696779801 2.321654922 1.400396563 1.554132661 1.826834202 1.702013177 1.821113782 1.85027234 1.934709098 3.101841228 1.814906811 1.957512567 1.6194923 2.119036085 1.408133344 2.277090122 2.636607361 2.10071226 2.148147187 1.966413301 1.939816427 2.163562167 1.751575031 2.181791192 2.052081668 1.624060611 1.92387942 1.936596742 1.87748692 1.703008113 2.587129311 3.773512894 2.255122207 1.869138089 2.80642278 2.690140508 2.510892672 2.478561289 1.999540031 2.124232936 2.261976297 1.927738799 2.552530445 2.080508302 2.506801445 2.009748473 2.806085377 2.663063121 2.153848878 2.539471553 2.17204665 2.175181503 2.738976386 2.44016464 1.999323823 1.905174261 1.892497882 2.162890087 1.623009459 2.533110079 2.294665583 2.378496156 2.466532538 1.990526879 2.176623156 2.190351132 2.684975342 2.04118073 2.420547041 3.174136175 2.080122469 1.752467428 2.464065897 2.922683352 2.652564026 2.321263364 2.459189651 1.828263688 2.377516102 2.304051952 2.08705039 1.934224106 2.778399892 2.615338505 2.356519162 2.279791117 2.135305558 1.776902921 2.390478936 2.094391362 2.060272554 2.343015578 3.074439459 2.191719498 3.157132188 2.839095312 2.597605948 2.653546603 2.946867204 1.353763966 2.189560628 2.859426366 3.052572007 2.561072395 2.430895475 2.511107638 2.415915206 2.761879928 2.272323051 1.915153016 1.831086865 2.873322896 2.146052266 2.28082164 1.85054943 1.879329379 1.19191442 2.28990729 2.065058339 2.506558801 2.271953004 3.02822676 2.39581049 2.5064758 2.543249621 2.817308089 2.765840934 2.21874641 1.891131465 2.096335084 2.258841251 2.416246568 3.146403231 1.793405425 2.514339579 2.081304659 1.989827783 2.215480877 1.811234792 2.103672128 2.089560461 1.633342607 1.916743335 1.588315448 2.059805691 2.171901209 1.40760533 1.411352815 2.152480717 2.538032374 2.084601167 1.977650626 1.873936182 1.777189972 1.992061134 2.271906926 2.009717546 2.131311338 1.811726814 1.81560945 2.516298555 1.850563822 2.285729983 2.625250303 1.652565153 2.828592387 1.915837716 1.340316617 2.099983619 2.095779045 1.589250661 1.278303111 2.042380878 1.710488423 1.745572413 2.422939582 2.187775619 2.177436834 3.159787337 2.018220597 2.004194001 2.132426402 2.874155877 2.09992571 2.109891947 1.792979758 2.36611737 1.537485643 1.782815183 1.844054376 1.764984208 2.755549481 1.724007901 2.444757292 1.817547074 2.900471053 1.989176823 2.599345457 2.31273102 1.998051234 1.624516861 2.311888001 2.473594721 1.356056358 2.311542484 2.022021256 2.370562998 2.174884108 2.461995571 3.116880081 1.965334028 2.053960975 2.282229808 2.013643196 2.21606448 2.080618493 2.437288719 2.267077807 2.378326237 2.178281987 2.168532932 1.629390973 1.307348873 3.022669905 2.581710929 2.295460537 2.03217684 2.866654119 2.683985116 2.602969931 2.461736818 3.216162669 3.024265914 2.005454414 3.017349887 2.429109373 3.83708997 2.542820437 2.52424833 2.111163382 2.421417257 3.980824483 1.839977058 2.641635771 1.717999902 1.759233285 2.766261582 2.114950529 2.221606243 2.387900003 1.306169527 2.330572438 2.934801979 2.278631334 3.063718019 2.003540433 2.000667032 2.783394324 1.623562386 3.845646515 1.845411697 1.806198471 2.748638866 2.018597442 2.143888509 4.065533319 2.021249626 2.849122035 1.348261651 1.942915224 2.346529408 1.349654847 1.553116133 1.873866544 1.370908966 1.442781257 1.255524899 1.880268716 1.77395626 1.784253691 2.283451538 2.293026903 1.326395472 1.755061113 1.680425553 1.916889982 2.010031299 1.532991521 1.955803657 2.508100047 2.514228757 2.607935201 1.307259288 1.885065169 1.047559498 2.299899995 1.780316968 2.110715505 1.507148309 2.042191348 2.719461411 1.64588609 2.614444601 2.684372549 2.719603972 2.668712546 2.164833561 2.429211849 2.077975895 1.974366934 1.881116268 2.28097063 2.36284394 2.588222995 1.633662819 3.259853509 1.108425179 1.848457298 1.942603072 1.51757131 1.966439594 2.35826867 2.161819996 2.381698134 1.855530469 2.645513251 2.07873051 1.44521799 1.945211603 2.960171612 3.998113658 2.21544232 1.866851183 1.919453505 1.958304471 1.610565467 1.946420266 2.374703776 2.547676335 2.561234787 1.797064202 1.668757138 1.658885794 1.899355445 1.691542069 2.931302436 2.076810047 1.164417241 2.017435673 2.481219069 1.979333695 1.576497668 2.288037225 1.463019141 1.555279231 2.518564884 1.632691057 1.243598205 1.58569011 2.024463021 2.484168484 1.985163396 2.00254787 1.180149332 2.342599216 2.340263872 2.457140956 2.713717863 1.926350216 1.811615105 1.899612382 2.280288745 3.287689232 3.341919289 1.830341276 3.342701368 2.046843801 1.778588695 1.80042412 2.056382051 1.938084131 1.383196206 1.490580672 3.069850879 2.813147187 2.226253396 1.754490264 1.408055589 1.834314171 1.75077304 1.638037355 2.53209549 2.068499652 2.415542324 2.148968111 2.274211805 1.944224025 1.718487439 1.963763491 1.700329064 2.095230426 2.412616258 1.930564051 1.879663755 1.5915695 2.232323099 2.280642367 2.398166848 1.856823825 1.960799328 1.530851891 3.254574606 2.341493184 2.126718469 1.449080702 1.574410972 1.601595524 2.005902975 1.678663887 1.857259101 2.088176167 3.20184249 1.353166963 1.914929638 1.224270467 1.517668573 2.942015756 2.165257404 1.417697689 2.071313029 3.319463556 1.861212044 3.537133344 3.102220066 2.581124865 2.149281775 2.206269674 2.0652177 2.469220873 2.38274535 2.592215964 1.969766635 1.884015568 2.342397604 1.453422443 2.599161704 1.82454983 1.808716329 2.830820369 1.57534412 1.506172588 2.135910383 2.467883573 1.871701127 2.088831434 2.281691144 1.859867042 1.636913342 2.005334565 2.616938 2.622534944 3.004428452 2.276156373 2.885857174 3.004498148 2.213432775 1.776125936 1.999958624 2.816336347 1.156434823 1.88486761 2.365934881 2.241054104 2.429179987 1.810176853 2.247548801 2.674319055 1.587686336 1.444953365 2.4629235 2.012159678 2.557999573 2.008317462 2.066549129 2.45970209 2.26049543 2.253087303 2.037115011 2.839475668 2.056321613 2.882306912 2.21434893 2.807358683 1.698142338 2.214235826 2.094764228 2.110808359 2.724460481 2.051338925 1.881429444 2.570588045 2.543881696 2.52323082 1.981248227 2.005609774 1.556742526 1.967134451 2.094514693 2.362506712 2.752418567 1.839091175 1.226001535 1.452550208 1.019478727 1.283671535 1.694971516 1.548685782 2.409010738 1.775236083 2.331643019 1.915005415 1.474422807 2.650821161 2.609423247 2.507522739

ENSG00000087884.13 AAMDC 3.383385746 3.212062673 3.64404549 2.863287881 3.371096452 3.820734377 3.662748571 3.043996701 2.890490206 3.27285289 3.531307657 3.122894008 3.579451116 3.083865439 2.575949863 3.197977295 3.369774663 4.650164438 3.216819258 3.710770353 2.865207317 3.222890812 3.198948451 3.130302962 3.132792286 3.534624185 3.293652056 3.703466687 3.737016912 4.003193755 3.197349491 3.480328027 2.75112314 3.43148154 3.829292824 3.515594551 3.781227208 3.646661835 3.655445772 3.032310826 3.446943285 3.442832788 3.433651171 3.182542377 3.340760791 3.326504927 3.203089763 3.806768611 3.954646927 3.46537079 3.440494647 3.587412917 3.368845596 3.924469598 3.285552113 3.368811318 4.121822229 2.688996562 3.05486692 3.773746219 3.574283758 3.367431115 3.008151124 3.167080275 3.283468678 2.689727742 3.493377918 3.797818617 3.21183 3.350352102 3.088048165 3.00444849 3.042880822 2.964880098 2.616278588 3.523840492 2.635476885 2.88696953 2.49985593 3.077231773 3.437239342 2.995120542 3.258660323 2.736390222 2.5993148 3.285996747 3.086701614 2.940494758 2.993577007 2.898828757 2.800588242 3.246790668 2.566761511 3.295317476 3.353988046 2.826971728 2.992629838 2.974965548 2.571026945 3.170142616 2.625647276 2.796499802 3.071424025 2.86611066 3.1038752 2.9703137 3.382798963 3.082101615 2.887576784 3.127348718 3.079571624 3.580121747 3.669247949 3.3135233 3.172159589 3.260202302 2.890370018 3.288880024 2.57718087 3.560956624 2.973995182 2.849276873 2.716179445 2.767494652 3.070279186 2.791894085 2.729799886 2.858787458 3.415446317 2.84818739 2.982407691 2.694027111 3.487065256 3.38634162 3.721733608 3.052285332 3.056981972 3.900165916 3.288641871 4.877298069 2.975399364 3.176661069 2.67449391 2.553898592 2.615767367 3.002064852 2.845727252 2.993851997 2.882371234 3.192643536 3.59415862 2.682985379 3.274780115 3.544852607 2.970943644 2.904992668 3.223553042 3.243543246 2.709240582 3.007510425 3.40107088 3.784715148 3.228545887 3.56372635 3.631508865 4.331201405 3.284171947 3.031078696 2.942456854 4.286681894 4.728403825 2.77730608 3.165638286 3.457597619 3.268565248 4.123880871 4.196004202 3.813656804 3.858173305 3.710536181 3.386396218 3.375761443 3.462246147 3.590456412 3.209613927 3.903704343 2.742720387 3.201412357 3.823621152 3.17390858 4.25812502 3.624940307 2.983524591 3.17769994 2.611987209 2.528404831 4.517096287 3.598942164 3.831074336 3.788120645 3.688473112 2.613358203 3.69521283 3.965836862 3.444562135 3.062940343 3.532175084 5.577058456 3.637324381 3.590343901 3.357741996 3.618429755 3.439512362 3.79542725 3.533513761 3.357970632 2.675764501 3.543067217 2.856509897 3.604429245 4.188629904 3.537927215 3.472543031 3.725352388 3.22453622 3.613048332 4.533312702 3.100179695 3.561151261 3.137681811 3.639842261 3.762994341 3.381158345 3.221976701 3.251092353 3.862914197 3.053505568 3.049203501 4.000536972 3.373105957 3.616247445 3.720723659 3.874376302 3.02896703 3.647986844 4.281780293 3.141684366 3.570150976 3.032055304 2.698664704 3.162850656 2.821847768 2.807650833 2.642909572 2.123315688 2.784445673 2.536665327 3.184214835 3.228261243 3.400268363 2.891303063 2.997824593 3.121731538 3.132007663 2.487079131 2.871885651 3.1278781 3.193285433 3.538747309 2.982907795 2.990346432 3.594672117 3.134981728 3.403939597 2.95036351 3.609860957 3.41048409 2.98740029 3.690851637 3.190750101 2.89719984 3.021435555 1.535898359 3.271809469 3.378477409 3.150435806 3.294694637 3.275942725 1.755774487 3.094009313 3.478636263 3.003909987 3.132144633 2.956257369 3.832994489 4.605979944 3.540885143 4.441730768 4.673331409 4.860152911 4.948801378 4.169352233 3.452102107 3.440272658 2.895605121 3.521216115 3.296804993 3.741231106 3.476905882 3.478651339 3.966504398 3.435887768 3.474888756 3.491832756 3.073758467 4.071019482 4.775236861 4.674482219 4.72719896 5.095856882 5.633156533 4.396712887 4.421239824 3.033803343 3.469152687 2.807544749 3.178368789 3.411674645 3.409495086 3.351987002 3.262032243 3.497747386 3.318197593 3.515807169 2.952994932 3.279392844 3.240799615 3.753115722 3.2671388 3.465261421 3.852012992 3.854138227 4.224762347 3.142849544 2.932235834 3.393544916 2.723313424 3.753024153 2.647407433 3.517947573 3.805328383 3.56784106 3.380847764 3.153129386 3.816589493 4.182251178 3.92039984 2.797412711 2.645882421 3.342888298 3.654835189 3.295358686 3.131844657 2.995017386 4.378606287 4.723419396 4.081905466 4.039903204 4.325414951 3.786016892 2.882501537 3.826671922 3.303158274 3.308613284 4.401280717 4.057383119 3.667109741 4.735707995 3.198422604 4.329329822 3.35912244 3.252151878 3.152654708 4.153051687 3.850958205 3.226192304 3.252630422 3.648232885 3.106328817 3.941716714 2.743367396 3.666460467 3.47607249 3.398529815 3.897498582 3.912865058 2.949658336 3.680776439 2.856370446 3.311444961 4.064558067 3.573799216 3.482159763 3.786194156 5.37172906 3.761315748 3.495643837 2.577881847 4.55270958 4.246848206 4.594323918 4.907433389 4.63837517 3.884198163 3.296060409 3.247391737 3.063648013 3.140016134 3.164479289 3.248751344 3.615926812 2.976423228 3.604562894 3.315962088 3.359868394 3.077849421 3.117347528 3.652771533 3.377781682 3.331556538 3.132591555 3.977567366 3.454149303 3.6147561 3.412290975 3.607349176 3.013022157 4.177342412 4.249265526 3.669489027 3.58877223 2.793532465 3.788242948 3.657484391 3.185781726 3.043806206 3.445152479 3.809143443 4.302896344 3.136367661 3.665635695 4.576470518 3.10361233 3.589629709 3.676005544 2.640290269 3.558307677 3.0739693 3.809086362 3.355908009 3.978849665 3.273070167 3.421364018 3.220258805 3.512268298 2.858209639 3.605181586 3.125663123 3.50478306 3.738839878 4.424631621 3.281180729 4.120808016 4.011709083 3.170020235 3.92380818 3.498828946 3.31040754 3.60810091 3.743065303 3.815431068 4.787172163 3.261064811 2.766079621 2.597304086 3.463391003 3.861844055 2.968007979 3.382474329 3.649126351 3.745196535 3.8137376 3.308270724 2.90124618 3.602399025 3.34681174 3.082300188 3.519257197 3.082299316 2.769351688 3.070863037 3.171369868 3.065056984 3.336382617 3.393984466 3.282391271 2.304437095 3.77771074 4.125817717 3.420733124 3.767482618 3.502038218 3.328797523 3.02221593 3.46034103 3.228131884 3.444331558 3.687654203 3.395365478 2.806263636 3.63834386 3.398578339 3.120588504 3.511313478 3.839481172 3.27504938 3.539665702 3.200812726 3.489551626 3.46364252 3.877016758 3.477813608 2.728108008 3.280195452 4.439030256 3.679629081 3.831890518 4.252939757 5.015794932 4.154466813 3.351689549 4.405557346 3.464910186 3.301772544 4.274921877 3.266074989 3.980885442 3.348834111

ENSG00000127837.8 AAMP 5.80471672 5.299548842 5.759004935 5.844503712 5.686876052 4.932246524 5.562873837 5.477740852 5.236864657 5.605516687 5.633527404 5.915388046 5.327049077 5.328624857 5.503047996 5.171016831 5.516619252 5.05907193 5.440615436 5.634641286 5.252776022 5.544406189 5.607585754 5.541366277 5.372220435 5.680942199 5.442434549 5.745433519 5.849583678 5.413732264 5.505477012 5.963749815 5.230381848 5.76553433 5.486809345 5.467987414 5.665721063 5.538249411 5.132548734 5.045317746 4.937936559 4.918061008 5.764392564 5.046325221 4.978597957 5.362098013 5.595312384 5.19730358 5.638822477 5.610914064 5.782894301 5.575016543 5.629358305 5.64849902 6.047315155 5.96376514 5.846575543 5.958188721 5.858215849 6.009900646 5.874515955 6.039143964 5.286932407 6.054221415 5.602844942 5.572831634 5.594488959 6.111423208 5.446706874 5.919694872 5.519522611 5.694577055 5.78844873 5.70555207 5.666184578 5.479509798 5.42945406 5.969969289 5.818812809 5.749849057 5.395779355 5.575231972 5.589608056 5.658976271 5.405783753 5.519200203 5.761594521 5.549735663 5.831466079 6.019138061 5.379341466 5.558161034 5.704139077 5.760872313 5.725683365 5.855525002 5.262771048 5.547683719 5.500916394 5.469248666 5.694085124 5.780794216 5.640135959 5.811818814 5.67664236 5.743305701 5.584037166 5.802280711 5.512265439 5.734033103 5.779403437 5.710458697 5.102829993 5.507452038 5.358081839 5.426926312 5.55267932 6.180221191 5.93725809 5.952019727 5.399690303 5.380372035 5.990445991 5.191136066 5.395001298 5.783030594 5.867326627 6.011801454 5.550352217 5.956575881 5.914391685 6.091405701 6.08385931 6.483359613 5.825792985 5.544956005 5.881994813 6.22902742 5.766642339 5.896687261 5.609699877 5.545815183 5.505521166 5.586247851 5.727020945 6.143463287 5.420129755 5.512360766 5.460328683 5.643663195 5.691011041 5.440947462 5.543743331 5.599602338 5.651569747 5.586164412 5.725295565 5.693428763 5.746093689 5.674372458 5.660064106 5.530098365 5.672520001 5.90546059 5.597234897 5.617811219 6.008723154 5.413289607 5.664633394 6.141283921 5.972708282 5.962311237 5.911198779 5.663713874 6.225959367 6.076293869 5.883281485 5.635744241 5.826558461 5.701176564 5.653406089 5.652871879 5.649864096 5.79814043 5.016041882 5.830474701 5.724763657 5.541250792 5.965240523 5.712622551 5.632965873 5.927542099 5.52670784 5.815068639 6.077531107 6.240226917 5.879789047 6.265016546 5.667440503 5.710444912 6.064154991 5.729354082 5.826855612 4.939901224 5.911767528 5.898283088 5.812993749 5.482272034 5.962332963 5.778405257 5.665449853 5.744939387 5.740661578 5.55747104 5.239534656 5.759280685 5.358980585 5.508470706 6.091069035 5.662402269 5.709804609 5.651297277 6.067353723 5.865729088 5.902819606 5.815705922 5.61392405 5.654502786 5.750922784 5.837797982 5.643389007 5.154763073 5.932316943 5.823386071 5.823052235 5.870359228 5.700970219 5.882461776 5.373883242 5.850492136 6.256124698 5.86187172 6.207354333 5.665716091 5.563347308 5.989282468 6.072723071 6.036083642 5.466907151 5.26838731 6.148911366 5.294736518 5.598386986 5.831183498 5.703642863 5.792027658 5.667331303 6.104419118 5.738442623 6.058672049 5.613451231 5.59357152 5.475469086 5.807995982 3.386701134 5.920776502 5.64420931 5.406886858 5.787928772 5.562140307 5.660101725 5.621296565 4.543547364 5.692894495 5.766514675 5.79807893 5.934865417 5.790201142 5.890766989 5.832661733 5.957500978 5.911998449 2.675307158 5.956497501 5.628008808 5.736843936 5.775517309 5.373641856 3.551563595 5.344564699 6.116248578 5.612512072 6.178807004 5.961475907 6.169858926 6.265988758 6.177385797 6.123621896 6.001659488 5.901939303 5.786061629 6.238529198 5.442508764 5.923847237 5.97559319 5.842359334 5.683521021 5.530933197 5.904383839 5.836763887 4.591173659 5.681793487 5.964178886 5.562702517 5.833145588 5.984721651 6.233546388 5.976918958 6.093889233 5.590740645 5.556715148 5.608380491 6.035518321 5.533705942 5.794754877 5.579610664 5.909458914 5.818883779 5.863060286 5.756162496 5.68870595 5.66419782 5.986058678 5.658843156 6.151368346 5.73262346 5.951360839 5.59545692 6.153331584 5.415035084 5.833492144 6.288370033 5.849310403 5.581317293 6.103536055 5.816866867 6.013455821 5.571912813 5.882172493 6.10065266 6.013652649 5.949616395 6.068645599 6.221493489 5.701723436 5.488160729 6.255271984 6.112541115 4.983932368 5.775050613 5.823982331 6.1639863 6.143434182 5.938514994 5.972516117 5.704694605 5.856634746 5.811640951 6.221975542 5.859644243 5.198799589 5.593702796 6.384430732 5.946779135 5.62428541 5.968375035 6.043554404 5.787577939 5.934616105 5.915612868 5.8440927 6.362478613 5.59718717 5.857324857 5.89898626 6.001995048 6.325529976 5.826094861 5.745905726 5.918850061 5.661494922 5.844523375 5.486731349 5.685845112 5.674383385 5.820107406 5.932441837 5.474480562 5.814857413 5.826668163 6.066198329 6.216925454 5.731438644 5.986570161 5.820420434 5.417360647 6.759845136 6.153006167 5.754583772 5.550036453 5.328728701 5.895051184 5.627831769 5.902914218 5.720511366 5.388904734 5.574254371 5.7802328 5.520734073 5.772667039 5.683339143 6.204683971 5.812502068 5.77924265 5.738313253 5.552618998 5.79759075 5.66662423 5.71606515 5.820289015 6.076875229 5.641650399 5.696704524 5.508980621 5.717166408 5.88525826 6.191986337 5.898299518 4.793354174 5.726347744 6.016496201 5.871505703 5.898655933 6.052965561 6.066536484 5.837445767 5.801078204 5.705730811 5.598630032 6.219967397 5.656613213 5.790041016 6.028524824 5.853366549 5.846626324 5.935432847 6.325976257 6.207190001 5.900932185 5.708434536 5.934252678 5.889170197 5.684288433 5.500401378 5.54109395 5.815361039 5.484778195 5.921968176 5.969497497 6.16442324 6.011573828 4.890952891 5.490091566 4.805636246 5.835185443 5.873843319 6.212514233 5.756714693 5.719734927 5.877810077 5.696307125 5.643192147 5.958620117 5.98361932 6.374327533 6.006232652 5.893236447 5.852730948 6.276274539 5.492268858 5.894638949 6.24904571 5.779751991 5.540396872 5.807016371 6.050405693 5.502386941 5.389205597 5.720709634 5.93568304 5.520417129 5.357531859 5.446936319 5.645460485 5.089652913 5.579354575 5.852365379 5.914510775 5.838005739 5.371326695 5.660381025 5.792402316 5.71960335 5.800816038 5.689779546 6.043822388 5.975147971 5.718454364 6.033012894 5.808837736 6.260374637 5.77512088 5.875709597 6.036078861 5.824431115 5.704702323 5.808717079 6.798929402 5.404286443 5.691863189 5.795016942 6.005710163 5.490972797 5.552505249 5.9178258 5.346747781 5.643870232 6.433961713 5.629215776 6.457800785 5.677550609 5.961128957 5.92014136 5.577365513 5.754304947 6.008907825 5.824431781 5.883936272

ENSG00000129673.8 AANAT 0.042549393 0.04218738 0.019574596 0.011130906 0.0116588 0.005662977 0.028889274 0 0.034237592 0 0.075300905 0.019337975 0.010326015 0.023735562 0.032321702 0.073469733 0.070464936 0.023744372 0.047869922 0.075761104 0.029974308 0.049383441 0.013017175 0.038490845 0.021394472 0.045135954 0.02579888 0 0 0.021803685 0.010777552 0.04884006 0.044663864 0.037960198 0.070752203 0.047395828 0.051343632 0.018728916 0.034737421 0.010256603 0.013284386 0 0.025235275 0 0.042190233 0.036546481 0.020884594 0.043915812 0 0.071135135 0.012908365 0.108465395 0 0.054918012 0.06207184 0.026679867 0.040142006 0.068780155 0.060593081 0.012438231 0.113064235 0.02650385 0.115786685 0.038771059 0.020505678 0 0.054914593 0 0.04170867 0.012825213 0.023608175 0.029894672 0.011849516 0 0.079467476 0.010383192 0.062313735 0.087352471 0.020907689 0 0.050171669 0.02478665 0 0.061967113 0.009809558 0.040036998 0.026443543 0.033156486 0.11392341 0.02199337 0.013921542 0.048295137 0 0.053625237 0.01805189 0.012235438 0.030018161 0 0.061913043 0.016611306 0.021062044 0.042341776 0.05688692 0.051036941 0 0.021421697 0.049603254 0.010606235 0.015820148 0.049794832 0.038424558 0.01476894 0 0.021886076 0 0.025826127 0.017855283 0.013857532 0.024595091 0.008006959 0.024932143 0.068151387 0 0.010276374 0.0171958 0.016573392 0 0.009151036 0.020002292 0.075996011 0 0 0.027054115 0.016668239 0.020606366 0.026385866 0 0.019918596 0.012877702 0.064973729 0.037108925 0.10471543 0.019364789 0 0.039848553 0.009722089 0.027339962 0.020078042 0.05224737 0.053397642 0.039231701 0.127215969 0.020899582 0.020035461 0 0.0101945 0.021365438 0 0.027017735 0.086774679 0.051388364 0.012785196 0.016912488 0.121061267 0.224873902 0.044972129 0.016149029 0.102681324 0.017908335 0.081225004 0.064180861 0.040098625 0.116650338 0.01480378 0 0 0.01450926 0.045000162 0.013212893 0.012387677 0.124618194 0.024297227 0.066150215 0.097779445 0.114586745 0.014932833 0.078658182 0.014461939 0.012533536 0.157311581 0 0.055229368 0.06360352 0.041503874 0.035266071 0.040222379 0.093548397 0.045186026 0.097527997 0.009644633 0.050198055 0.021290754 0.01400684 0.012073971 0 0 0.022008217 0.093270194 0.018250837 0 0.039526462 0.014527478 0.021629166 0.028270866 0.029331485 0 0.02594668 0.039088468 0.017945368 0 0 0.053016572 0 0.011087862 0.031299468 0 0.295061707 0.008811846 0.029731399 0.04640151 0.095794105 0.021489622 0.03015342 0 0.009461107 0.029148876 0 0 0.028769217 0.010842018 0.041489613 0.034703928 0.012257321 0.03017297 0.055336313 0.034740208 0.017792485 0.019753992 0.034354137 0.319165751 0.06470723 0.075389315 0.023444529 0.04646345 0 0.029493794 0.010994012 0.019369412 0.032434289 0.040648837 0.021660504 0.077594064 0.011277707 0.008928188 0.356311832 0.0221457 0.010931839 0.148505879 0 0.03910319 0.008590238 0.033069733 0 0.118378283 0.029704448 0.01125298 0.010909022 0.063039404 0 0.014014704 0.035556489 0.035075869 0.408954106 0.020193421 0.118360544 0.066772271 0.009683594 0.067280706 0.121511483 0.075005364 0.03930174 0.038147735 0.026948066 0.014544759 0.033999964 0.063666512 0.081075984 0.110633404 0.046381228 0.101283196 0.120554563 0.066499023 0.076276517 0.034314222 0.082590879 0.048911287 0.014927624 0.068278813 0.074781636 0.021085759 0.01627991 0 0.041896012 0.037295166 0.012916042 0.093807923 0.056094396 0.092094887 0.135797785 0.040702731 0.066050076 0.143964209 0.097566943 0.086339741 0.133212327 0.030766994 0.011270311 0.054403072 0 0.052522719 0.044523911 0.048310627 0.0561137 0.130620307 0.018867524 0 0.048075118 0.074844506 0.0405774 0.125719162 0.05121089 0.063597634 0.123285088 0.040844673 0.053421744 0.081135166 0.064639298 0.064504237 0.033643104 0.09360903 0.012364345 0.125750854 0 0.055262964 0.012920139 0.298004109 0.048888461 0.016757054 0.057993462 0 0.048327138 0.026499796 0.07048628 0 0.04247296 0.080982044 0.079360748 0.013917135 0.0213232 0.01265909 0.153140124 0.092789653 0.023390602 0.109589922 0.093892095 0.018177871 0 0.03988273 0.050188302 0.21423854 0.085547379 0.052287218 0.180840072 0.064319045 0.070620295 0.05120239 0.049914996 0.03063343 0.02098131 0.097213814 0.104413471 0 0.055844248 0.028465114 0.040989574 0 0.025210383 0.059186367 0.081186363 0.01333461 0.06652986 0.013210613 0.065948068 0.028256012 0.046557588 0.044467484 0.041798539 0 0.033155892 0.18900337 0.016106221 0.039082329 0.078961464 0.046977693 0.128679901 0.025159309 0.042584232 0.059831934 0.065886807 0.039987332 0.03857463 0.096773437 0.053535825 0.08498156 0.069738173 0.033086972 0.019678827 0.114183048 0.026419755 0.02417764 0.082939214 0.064028953 0.201346321 0.076112669 0.04696124 0.017614776 0.118344484 0.096169629 0.071416206 0.070467162 0.071585096 0.035606554 0.048486397 0.033484536 0.043591544 0.115985604 0.032741085 0.051894598 0.067513886 0.038497331 0.013942841 0.057873484 0.052242704 0.038937803 0 0.041110811 0.034734175 0 0.117229848 0.011009172 0 0.051761446 0.02798398 0.044124116 0.139465101 0.043941155 0.057665605 0 0.033951961 0 0.00999151 0.034470405 0.041134552 0.09676672 0.0150036 0.065100379 0.092414331 0.05362266 0.151173852 0.070971801 0.059286786 0.140922907 0.025904128 0.110950906 0.010808205 0.028189523 0.063057771 0.080696114 0.025925541 0.025831424 0.105185858 0.051961756 0.16397346 0.053057231 0 0.014787884 0.021398549 0.060612303 0.151701993 0.063561098 0 0.04995825 0.062528858 0.012458094 0 0.051232584 0.033453403 0.04521611 0.043863246 0.016345086 0.129610594 0.084644433 0.044146942 0.035926971 0.032474515 0.040579315 0 0 0 0.051620885 0.045347522 0.063146953 0.266112547 0.037710426 0 0.011872251 0.061041378 0.064676083 0.087026037 0 0.011995519 0 0.024151805 0.042764041 0.121181185 0.020048908 0.047227287 0.080449131 0.16384346 0.079371908 0.025370851 0.022516452 0.025969485 0.078387062 0 0.082165029 0.02074435 0.083711885

ENSG00000131043.10 AAR2 4.315582121 3.962790173 4.263383072 3.992429302 4.401650134 4.401725501 4.282095616 4.211325653 4.276869264 4.11085476 4.277259037 4.225853088 4.285089034 4.306770264 4.38465315 4.187866613 4.205638264 4.288371246 4.072568177 4.294641618 4.146844199 4.301215914 4.350137723 4.324683726 4.103097359 4.426895332 4.181511994 4.371449176 4.596965695 4.395479864 4.482993587 4.270256792 3.338485825 4.280115753 4.12284966 4.429196444 4.136689606 4.564885114 3.501637735 3.731100907 3.590631225 3.592091273 4.391110938 4.055326876 3.568581587 4.255540714 4.247567995 3.722195981 4.370668227 4.32588881 4.120589459 3.973122993 3.866883567 3.98905098 4.412321428 3.995910107 3.605484196 3.866602579 4.307859987 4.067345739 4.006929941 4.250732372 3.911292295 4.106911593 4.010223367 4.004682837 4.239708444 4.125802098 4.142000102 4.088957233 4.256447035 3.828271402 4.230381727 4.192115493 3.964683136 4.251926239 3.949077254 4.117742025 4.731272192 4.296663782 4.10337375 4.280281388 4.109545067 4.194201639 4.081863269 4.15282822 3.957321884 4.343529985 3.608748513 4.222834481 4.196817617 4.045683062 4.625663905 4.353819077 4.404994999 4.325910496 4.041941232 4.211487353 4.010618169 4.282358206 4.507008009 4.371454824 4.142009652 4.462897706 4.177379039 4.120695525 4.153695713 4.269064093 4.083660321 4.349439651 4.384179269 4.206776403 4.35316594 4.10781374 4.115958105 4.031399092 4.135711667 4.547470748 4.243608177 4.34022211 3.996542276 4.012512287 4.146228047 3.879862859 4.031811844 4.224218525 4.414903358 4.639528829 4.124816392 4.148644593 4.157415325 3.956349593 4.233517697 3.579656799 4.509183743 4.306238854 4.260221144 4.289556623 4.193710513 3.800646546 4.391505625 4.077153372 4.17804469 4.039084891 4.15314813 4.203943424 4.077236275 4.215486259 3.854074316 4.070064038 4.03041137 4.03871925 4.314921695 4.150541199 4.259376127 4.094731951 4.22791982 3.939425998 4.287631739 4.034448385 3.466501737 4.162217309 4.299802439 4.40098187 4.282103054 4.008442449 4.082278294 4.070392072 4.213652849 4.031973645 3.958173984 3.932131303 4.196927889 3.887837071 4.182012535 4.069703955 4.317971259 4.348486441 4.077819164 4.269671583 4.148434381 4.081924348 4.123932247 4.482269882 4.227127269 3.927713125 4.141526596 4.173723936 4.105385193 4.182631663 4.359281195 4.235583819 4.087067333 4.068239747 3.646457091 4.028659588 4.047143425 3.759730863 3.958147025 4.127643319 4.409682663 4.169816122 4.357456361 3.770611845 4.215391397 4.436211813 4.200951249 4.161122347 4.157395294 4.238065955 4.431788535 4.071860233 4.417141578 4.424531665 3.495304337 4.11506527 4.326088406 4.225628726 4.275627361 4.424754741 3.795836686 4.411503246 4.473741402 4.28000552 4.441239579 4.111215583 3.938529963 4.213965258 4.434384797 4.728005477 3.765316668 4.117124483 4.531711687 4.179793991 4.393201593 4.255075244 3.95889725 4.187347998 4.224956057 4.407529636 4.461209545 3.989537859 4.102074873 4.165163594 4.568010332 3.949243721 4.150243272 4.355296403 4.053650372 4.113231715 4.159827942 4.014517732 4.008915289 3.954837776 4.136155361 4.184491895 4.347034106 4.434193182 3.82932937 4.237290419 4.084121754 4.184447452 4.17863239 4.039962932 3.277043216 4.353697646 4.186293368 4.027464584 4.251146934 4.105145034 4.246704324 4.263108632 3.175789151 4.121553581 4.241002105 4.141679973 4.230333894 4.223410562 3.775235004 3.583618122 4.092486852 4.303630565 2.563147333 4.237898441 4.18731818 4.192318496 4.29918763 3.963348217 2.750625409 4.209422848 3.939362027 4.152568066 3.927356743 3.87035177 3.999993015 4.066385241 3.945345383 4.005404601 3.823500001 4.080837888 4.11528961 4.39153243 3.957777069 4.119920192 4.013085566 4.036290893 3.981916742 3.840255256 4.080362815 4.202483758 3.631862718 4.150654617 4.245308434 4.027182022 3.848060018 3.779620049 3.916552019 3.915311648 4.074744208 3.667597579 4.121138948 4.022633391 4.024887507 4.44959027 4.184922035 4.232303798 4.083316411 4.145823075 3.86495698 4.190266553 4.228994401 4.09803617 4.147457462 4.204528508 3.888966851 4.123433208 4.292903549 4.386884609 4.148448786 4.168638392 3.936974221 3.914197034 4.132453264 2.999379529 4.109725416 4.481116067 4.474384641 4.2518314 4.108873112 4.229538686 4.027854007 4.214067447 3.957345664 4.440674184 4.324356102 4.065961492 4.012624481 4.420916923 4.028886857 3.807501492 4.411058878 4.33389929 4.166646937 4.256483702 3.943959889 4.188297355 4.538422898 4.181764757 4.334885299 3.874001697 4.044503143 4.130985015 4.399761897 4.166694995 3.934054702 4.058465759 4.114670131 4.085346329 4.247187816 4.370693208 4.116975707 4.331022272 4.166116169 4.050290085 4.296306542 4.27483594 4.387563658 4.166673501 4.344510893 4.266216986 4.098609875 4.700556182 4.018740414 3.961543883 4.109522062 3.983680582 4.289064161 3.47823536 4.462078545 4.197856027 4.480993095 4.125087814 4.187066005 4.186559849 4.023615819 3.898438264 4.020851749 4.056591465 3.722335629 3.872030722 3.858193902 3.915732589 4.126660591 4.340626874 3.9942424 4.043206623 3.855654159 4.108161281 4.019101057 4.122219294 4.251960045 4.097617385 3.889155971 4.325801413 4.185845262 4.145721934 4.429454358 4.06792175 3.499671335 4.221258919 4.446316642 4.166603331 4.141653084 4.276745896 3.986006624 4.084326367 4.806383684 3.920936544 3.553924183 4.062646055 4.33979183 4.008448386 4.182875638 4.310211414 4.232041621 4.029996266 4.115153573 4.192739546 4.141324463 4.229731736 3.81616176 3.604655719 4.045113633 3.956279915 4.285882892 4.300559565 4.190688487 4.046828777 4.532625893 4.505567354 4.258235486 4.189002687 4.225383976 4.061505217 4.175221235 4.254859916 3.934027712 4.142420956 4.024141692 4.445161396 4.277258724 2.942170089 3.703858197 3.221710995 3.801378021 3.322665176 4.130140375 4.23895779 4.200261208 4.250988008 4.005055811 4.276984066 4.154314447 4.401357014 4.089803029 4.07774224 3.907347346 4.066820899 4.1796093 4.254895156 4.362633931 4.293443633 4.856658662 3.999448906 4.152611322 3.959055785 3.998197084 3.959537714 4.223344189 4.365102741 3.993875063 4.051853639 4.316096766 3.714489006 4.102798044 3.991231262 4.156431307 4.090133155 4.038743229 4.158484485 4.16306512 4.071326228 3.804191232 3.796877143 4.076912238 3.975780396 4.330993064 4.370149998 4.011709145 4.126939649 4.083813139 4.167367565 4.208951446 4.26781632 4.124653875 3.949992332 4.246676286 4.115885525 3.979689943 4.35457626 4.264990705 4.150885306 4.12931383 4.351398014 4.269510469 3.500500675 3.877280878 4.28052704 4.176661486 4.297668088 4.161041914 4.304694151 4.147371192 4.210724636 2.982774315 4.150752773 4.235555131 4.827473996

ENSG00000205002.3 AARD 0.417040983 0.199354506 0.152675427 0.126206762 0.30538716 1.149071077 0.37846409 0.370013968 0.579421469 0.418366854 0.249980842 0.182406475 0.59332054 0.603713428 0.228267053 0.797961495 0.28648519 2.018314506 0.405668633 0.641063893 0.282181435 0.250604015 0.410533453 0.302438901 0.338585762 0.385007573 2.150670027 1.099473802 0.366377175 0.582796079 0.122351047 0.044413284 0.099459637 0.497254238 0.592369395 0.250385176 0.539978521 0.184514764 0.031574917 0.116649426 0.047673171 0.035241085 0.100435321 0.010678697 0.053420399 0.227525576 0.220938419 0.047784126 0.4627418 0.146758114 0.267845357 0.259337164 0.088464469 0.304346837 0.382249857 0.094626297 0.572156604 0.037875768 0.482593632 0.130063829 0.434660706 1.266438106 0.704657084 0.069651017 0.108336296 0.106903798 0.08230901 0.113340961 0.128575392 0.090664118 0.094114662 0.045001369 0.114194073 1.780229782 0.154602692 0.037358234 0.011517515 0.07952996 0.269557418 0.381227705 0.348361079 0.088056124 0.116735571 0.179930003 0.035312436 0.115083957 0.149483181 0.111315745 0.181787214 0 0.098192423 0.08653653 0.322020822 0.160776181 0.199902003 0.197776423 0.220881306 0.025581736 0.08909274 0.275358595 0.128911539 1.100848167 0.202427314 0.218440228 0.472106813 0.139950763 0.796471422 0.272248467 0.322251245 0.193437015 0.514884483 2.148411954 0.294085539 0.656248271 0.190543492 0.091666137 0.176362433 0.049704687 1.400111505 0.084932716 0.151588556 0.575741386 0.196545388 0.159408901 0.40181006 0.16432544 0.472596799 0.286343397 0.251133372 0.144354684 0.857290641 0.021948499 0.337467221 0.250861942 0.393394498 0.160030968 0.085165551 0.138790427 0.034810965 0.059114341 0.138217081 0.085096232 0.159059762 0.146496993 0.236336766 0.102552142 0.28691195 0.164634819 0.43465519 0.132062598 0.315027235 0.064405877 0.12211065 0.243803814 0.07244749 0.277954785 0.165966709 0.099070747 0.09579659 0.145769727 0.559842177 2.423402428 0.292589372 0.169788878 0.285444959 0.969601042 0.14039242 0.374750942 0.334384782 0.41472082 0.077341748 0.095236001 0.140060517 0.091602449 0 0.232077595 0.114496669 0.348558915 0.070557472 0.42707952 0.282094375 0.263602224 0.265861716 0.099806387 0.585324197 0.167111508 0.130255523 0.185499827 0.056044809 0.157126198 0.55480976 0.418577158 0.131576538 0.037733368 0.010764659 0.49643389 0.621748979 0.041085747 0.427308164 0.189127777 0.12221886 0.066599599 0.537807904 0.13645171 0.428542691 0.142383236 0.097321275 0.570568823 0.057217258 0.063728834 0.179389262 0.039232703 0.141251638 0.092047357 0.039802448 0.944698984 0.240314308 0.671062114 0.048366435 0.275682398 0.067271968 0.362315825 0.308920854 0.455509104 0.179838444 0.326163969 0.082527492 0.100729023 0.203194581 1.428473233 0.04427843 0.243604829 0.123655022 0.112561725 0.155107244 0.267650459 0.030262301 0.108116784 0.356606428 0.095575136 0.833069839 0.131973305 0.603168953 0.181834416 0.438318703 0.141784878 0.071353082 1.403853843 0.051674312 0.224798812 0.037075168 0.108266858 0.113453069 0.154359555 0.093622843 0.053119558 0.115494285 0.04359767 0.029479326 0.116797244 0.086521556 0.042787984 0.108865419 0.055826815 0.29212092 0.180691121 0.177873943 0.102767534 0.150427312 0.11745153 0.327517828 0.407427229 0 0.436916843 0.146698987 0.040456255 0.132882956 0.06669171 0.11260695 0.122515477 0.063932274 0.113612024 0.117717597 0.115283099 0.067326217 0.04365526 0.05198346 0.36853075 0 0.090317349 0.953165518 0.196570602 0 0.114767305 0.176210517 0.023448857 0.05309759 0.273884022 0.366506361 0.233997239 0.569082065 0.118574197 0.0580813 0.113639814 0.032702509 0.209828078 0.117683412 0.387396238 0.109020209 0.111307329 0.703571277 0.221348653 0.105413325 0.222117181 0.057728958 0.106012758 0.080790401 0.369347101 0.353672394 0.096643441 0.500511367 0.293022178 0.220542908 0.011494986 0.424960601 0.108738038 0.089615749 0.307157805 0.535791428 0.121051277 0.12761808 0.054708004 0.025745316 0.160055733 0.115919953 0.168586782 0.281384746 0.164614522 0.024697835 0.434403752 0.198667543 0.193105211 0.348661761 0.024861414 0.470225637 0.285528282 0.029704369 0.509415117 0.089859171 0.085242628 0 0.232137414 0.023828463 0.164200809 0.334991411 0.11017844 0 0.059950717 0.164831975 0.213858075 0.43077838 0.035972422 0.038825597 0.05581052 0.21756119 0.143846919 0.152180042 0.190068786 0.121514427 0.034224401 0.110886147 0.024650394 0.073056547 0.153941225 0.463150275 0.111817031 0.080279843 0.375090451 0.115790746 0.195796927 0.106037157 0.102622142 0.13555796 0.014853396 0.095351918 0.208908874 0.240165323 0.095179779 0.163124698 0.088535021 0.095112885 0.04083139 0.017130717 0.124939633 0.143611552 0.030548464 0.12171935 0 0 0.203838534 0.201606952 0.011999753 0.74801682 0.270851127 0.056174592 0.143016791 0.348889149 0.155775137 0.172039055 0.117333839 0.099433519 0.148011902 0.063081191 0.111216686 0.28716034 0.152910506 0.07642185 0.041011939 0.172848348 0.205527935 0.102743104 0.088132769 0.060610718 0.150792943 0.196790064 0.25893669 0.070222916 0.118312465 0.711633426 0.561376873 0.009656636 0.378735923 0.05072565 0.198573129 0.042702247 1.815887626 0.054931935 0.070485185 0.244043501 0.125517394 0.115218849 0.174488557 0.271294073 0.030434895 0.049374642 0.145891366 0.058915475 0.165986135 0.62643633 0.034996658 0.167928899 0.128171606 0.104784006 0.04700635 0.165824527 0.012566412 0.031571963 0.039565584 0.062010377 0.058982452 0.088515186 0.301204307 0.225206493 0.104599804 0.192094696 0.224631714 0.21084318 0.060729528 1.370469016 0.050945257 0.242437261 0.092025515 0.109403204 0.197833035 0.541619658 0.087957098 0.163664079 0.064666634 0.486682508 0.138450921 0.223537985 0.048066723 0.318172297 0.17714972 0.009816797 0.025617858 0.05736775 0.117534297 0.312047047 0.354856262 0.210343861 0.023821805 0.011218554 0.106388061 0.195798611 0.091506655 0.166211458 0.293914656 0.128200721 0.080323438 0.011320425 0.118109647 0.023024104 0.109353236 1.638838952 0.520949288 0.295369119 0.067885098 0.247151982 0.434816939 0.546830392 0.089510147 0.066292096 0.06459187 0.114604753 0.107960259 2.201754162 0.039794907 0.304910097 0.447668065 0.06541842 0.202749407 0.144172594 0.194434744 0.208591137 0.124369904 0.314165492 0.008554514 0.754352997 0.112661001 0.838676492 0.415455536 1.463411719 0.16139897 0.959603159 0.105993363 0.285761971 0.30370502 0.321461719 0.227956145 0.479908356 0.747077825 2.57429802 0.438169449 0.491897608 0.056456709 0.091867502 0.048958741

ENSG00000090861.14 AARS 5.424007934 4.820092997 5.244134399 5.492339247 5.194183818 5.262316145 4.909824079 5.45098845 5.286608787 5.482855021 5.359928737 5.521259916 5.452413317 5.029995206 5.63960868 5.130406875 4.794899198 5.03230055 5.224986673 5.142938066 5.256779133 5.390549666 5.294669544 5.456173878 5.160235071 5.135566009 5.380480218 5.126951719 5.430782698 5.186129925 5.484577639 5.001727457 4.232994321 5.067944476 5.01103001 5.455888982 5.212292659 4.89863471 5.176381999 5.398293545 5.152314852 5.166115876 5.567527852 5.218999802 5.208676086 5.02748646 5.493858769 5.497223167 5.660753343 5.646682936 4.920872633 5.133249181 5.543609258 5.004265091 5.170001921 5.327326847 5.351373241 5.52251465 5.210786771 5.581476347 5.104791538 5.53528665 4.967876362 5.575371829 5.268240656 4.970478875 5.440719856 6.009492479 4.84237418 5.171235473 5.141118329 5.566620488 5.576033318 5.288104427 4.00435351 5.661684526 5.267466169 4.880300052 6.52554766 5.303382422 5.260022295 4.696206261 6.204727044 5.454880749 5.476895319 5.533545528 5.385877928 5.417929756 5.411359802 4.679709759 5.451946775 5.207298377 4.764823475 5.502826669 5.863256408 5.391868129 5.041404974 4.957464133 4.633027371 5.045291085 5.576096745 5.911673966 5.305042857 5.60415466 5.748350197 5.542857864 5.359936781 5.711476025 5.630863094 5.865751147 5.303262109 5.239733608 5.198112471 5.490534066 5.338399292 5.592513833 5.501480704 5.759146255 6.054352076 6.005627748 5.410155652 5.31104657 4.091763412 5.304704729 4.944585778 5.627304254 5.866526188 6.051322091 5.634584895 5.620591377 5.329334201 5.525327184 5.648386892 5.045416955 5.600293505 5.739854576 5.748081238 5.03054155 4.973537811 4.730925912 5.498697821 4.792458444 5.268097753 5.673649041 5.570190745 6.28873033 4.936439892 5.498846233 4.986740249 5.573027879 5.684400876 4.735319841 5.526168185 5.437411546 5.59835897 5.052932034 5.358948635 5.208791444 4.563817229 4.745073132 4.56666435 5.520275395 5.630919859 5.538936846 4.980664305 4.173139387 5.369182507 5.103340422 5.304051955 5.807979975 5.297237062 5.558154392 5.757879701 5.299797097 5.766072598 5.483806975 5.291666639 5.298261827 5.039389572 5.454815627 5.209122646 5.599018746 5.253193768 4.772323979 5.658359933 5.317390806 5.758800553 5.279418541 5.874973732 5.268592522 5.042242872 5.478691942 5.442476631 5.844653396 5.510416327 5.443489168 5.533238336 5.398298539 5.257126308 4.752211761 5.727967598 5.177232898 5.507602084 4.595515126 5.158830257 5.698538525 4.309378572 5.152838465 5.419094692 5.68132573 5.347771439 5.1524505 5.427414753 5.351708533 3.94394145 4.861374805 5.396789361 5.187017058 5.925009615 5.442352309 6.31608465 5.523479316 5.775867695 4.337805562 5.601216504 5.181137424 5.100144448 4.874253454 5.468211029 5.675080691 4.314475442 4.422254017 5.665842369 5.709773453 5.348554967 5.748645106 4.677496545 6.068137496 5.004638874 4.967089037 5.550555777 4.386984723 5.081512689 5.901016488 5.317760998 5.414385681 5.525438288 5.961195792 5.156367757 5.1163212 5.824276207 5.70449097 5.254728632 5.780106263 4.926872207 5.597960342 4.688736468 5.541311424 5.562700152 5.293769797 5.448088335 4.477376146 5.400123686 5.593716845 4.527111437 4.586151964 5.860590464 5.035648761 5.510784406 5.649717878 6.022977112 5.363232701 4.287080122 5.276099769 5.418769412 5.826852644 5.868334691 5.517991352 5.373833826 4.615408372 5.599961373 5.645370349 4.078890487 5.850344477 5.532695225 5.610129843 5.733322742 5.769077418 4.978173423 5.163425888 4.332172403 4.225217667 4.235820881 4.938271405 5.781794138 5.775131697 5.050575428 5.598829771 5.52235508 5.683467201 4.895543485 4.632737435 5.502979884 5.653071709 4.66650353 5.059047399 5.500292304 4.808300953 5.374901984 4.560599196 4.538493326 5.254322298 4.959120862 5.400483346 5.518678901 5.350220806 6.046319327 6.069214659 4.718110679 5.278413549 5.099933975 5.402460835 5.751420838 5.333849818 4.930384905 5.165508989 5.307999009 5.750171564 5.101084214 5.584972813 5.536624096 5.44907314 5.663234397 5.079698514 5.596257149 5.441954781 4.762829669 5.43187424 5.235179783 5.307688035 5.433598052 5.202287616 5.243874222 5.452138781 5.158017476 5.328208716 5.52916724 5.244583772 4.665401574 4.813668701 5.844193293 5.412405196 4.914029596 5.53015301 5.329124933 4.406253739 5.353481567 5.781749072 4.751495235 4.463918749 4.597884035 5.997156076 5.413094415 4.674877967 5.628350741 4.803996057 5.04691633 4.937939649 5.385337824 5.826190273 4.918423939 5.268009324 4.737107856 4.963081156 4.734949119 5.294432781 5.399652382 5.323072091 5.610380091 4.879952064 5.640073759 5.039214727 5.427457066 5.719684134 5.5414793 4.732773379 4.6176881 5.171941822 5.859747569 5.081808071 5.967556051 5.064909295 4.967366121 5.259773867 5.457356064 5.308835877 5.435879188 3.929999333 5.254036845 5.308606522 5.054040972 5.992791105 5.545270104 5.657592138 4.996413497 5.129987055 4.765300665 5.894168565 4.875776989 4.275524133 5.299075271 5.041402558 4.917062549 5.350075534 5.520147676 4.639394611 5.629485145 5.792279414 4.81465897 4.94500874 5.564888201 5.441004092 5.581273833 5.297007151 5.49343187 5.434941191 4.538752435 5.042904448 5.758492222 5.184318559 5.142736902 5.249130039 5.432385117 4.745365228 5.293987926 5.642220957 4.908427935 5.3399239 4.561202006 4.974990197 5.736069765 5.516366579 5.548514507 5.671303431 5.214071593 4.510330493 5.645428693 4.801423446 5.087991406 5.726155017 5.344723203 5.048518966 5.13944414 5.519610343 5.244709321 4.498751179 5.90521738 5.66925215 5.639898594 5.470350882 5.433283935 5.175172549 4.995156085 4.851159979 5.507866044 5.345943836 5.050963889 5.082769466 5.61138797 4.676644211 5.361886761 4.588788536 3.977357661 4.329595888 5.096402486 5.336939222 4.996696561 4.454988146 4.966775086 5.288465115 4.901154345 4.121788156 4.894508855 6.144439125 6.257576255 5.889728127 5.199271538 4.471784812 5.012537706 5.148612227 5.360759444 5.964231269 6.558478931 5.212278274 5.529352634 5.273046938 5.617910967 5.036759991 5.180498334 4.83269484 5.858876512 4.833177314 5.069633585 4.880489043 6.314354616 5.805825194 5.517281794 4.936782153 5.535016177 5.202216623 4.960772541 5.547922 4.562121223 4.78445553 4.999566658 4.865591734 6.244016464 5.839330559 5.390248559 4.586817193 5.34279304 5.277821108 5.445515335 5.433068002 5.366620583 5.970480581 5.34943833 5.69129584 5.301122077 4.538173845 5.720968405 4.956659454 4.71740369 5.263273828 4.627024655 4.294322345 4.353785774 4.935520747 5.576255559 5.186734434 5.370038288 4.776244521 4.188728342 4.767654288 4.253408725 4.913966343 5.385412535 5.186133697

ENSG00000124608.4 AARS2 3.077721735 2.875840775 2.940204035 2.864493047 2.560536416 2.220121932 1.826034805 2.790056191 2.69737137 2.865750734 3.026793075 2.914365224 2.670315325 2.433947959 2.737319094 2.524481921 2.120901769 2.061798534 2.814324702 2.385950803 2.56229549 2.675921474 2.907379889 2.845889895 2.822688826 2.53194793 2.623467351 2.814486017 2.746377188 2.574864925 2.792012863 2.984169385 1.91827401 2.785020641 2.813179746 2.671074017 2.471173356 2.291272476 2.013707821 2.28549962 2.083448516 2.059569592 2.862218094 2.327458349 2.036619882 2.598062388 3.009144524 1.981194417 2.723345905 3.20606185 2.934491044 3.125516152 2.965088952 2.722803759 3.093528267 2.567180355 2.377952979 3.08729247 3.007577031 3.12083891 2.6943856 2.96995494 2.570288909 2.806478019 2.724455276 2.852841237 2.706796608 2.526046445 2.861175942 2.784465187 2.745909858 2.912484402 3.085513695 2.758395031 2.856794838 2.922311556 2.824043109 3.024254457 3.100249101 3.033302568 2.479108482 2.956748827 2.983480516 3.119198701 2.54145758 2.908754293 2.698310167 2.923410987 2.537638826 2.866534773 2.9822441 2.887647026 3.026996315 3.158325215 2.899918694 3.204689058 2.620447425 2.619499165 2.592540098 2.502717288 2.687943024 3.016551412 2.926249114 3.164840843 2.69237317 2.675580478 2.89213925 2.765019556 2.772242478 2.932466757 2.861575748 3.560137071 2.722743319 2.774330524 2.55086219 2.568787043 2.313893342 3.510501668 2.777842074 2.522131867 2.00823975 2.443789724 2.963147514 2.751287753 2.691304676 3.066383318 3.168216017 3.100511116 2.562784566 2.686441837 2.963171842 2.707462169 2.999286471 2.925264868 2.977395809 2.460722875 2.897928958 2.671978603 3.080741507 2.304121355 3.219964352 2.77021292 2.972252372 2.750183342 2.95945532 2.706256351 3.143641678 2.951181941 2.861335509 2.865962407 2.896018199 2.890199504 3.091595089 2.698280108 2.878538168 2.960842251 2.953615773 2.724371755 3.056663989 2.877586027 2.704243025 2.610488537 3.079022041 3.007337757 3.050857473 2.506942653 3.3431746 2.917680478 3.051996425 2.382688299 2.761164077 2.814831495 3.03468859 2.868138783 2.780846884 2.670875094 2.983053387 2.800157612 2.886450103 3.009410152 3.143365997 2.982153569 2.842785672 2.699044421 3.137789744 2.891430766 3.125224379 2.949498031 3.070819983 2.977108631 2.634838453 3.015480616 2.921444618 2.963699094 2.770011746 2.909090801 2.376760229 2.459805264 2.97219484 2.418834491 2.789318574 2.883316802 2.6006408 2.023642152 2.75781797 3.02443009 2.241323917 1.928113843 2.626038824 2.729075838 2.761620118 2.366362923 2.94628968 2.77928973 1.915847661 2.155886344 2.323055231 2.521186414 2.85163717 2.940448329 2.351160012 2.788438436 2.902195214 2.743354149 2.730331849 2.73804169 2.282577576 2.857305556 2.959690215 2.961803773 2.215343406 1.993561484 2.636795905 2.961586668 2.665369859 2.839275703 2.803375279 2.682181018 2.110472255 2.632381435 2.411061309 2.756205897 3.018616784 2.809216093 2.855212962 2.342931 3.146700819 3.161657922 2.957122184 2.866869558 2.782103224 2.662253048 2.916274477 2.705654104 3.130468149 2.999088763 2.866884991 3.186295778 2.743297606 2.722957939 2.856666743 3.039962798 2.742049048 2.850459683 2.515774761 2.931430686 2.934108379 2.835271148 2.767773714 2.854514826 2.892743921 2.675246924 2.370279462 2.667802205 2.843226125 2.823883248 2.951092252 2.653354688 2.658794881 2.589446772 2.997925333 3.073746889 1.410313669 2.770775002 3.160662625 3.074494695 2.819012818 2.651243405 1.893892576 3.046166515 2.728714001 2.98835689 2.838620994 2.438163451 2.579621149 2.645109615 2.158026689 2.331723718 2.560344094 2.41679319 2.500260548 2.820062921 2.768542116 3.142565164 2.859421443 2.951499418 2.955495566 2.84272065 3.199016164 2.752520962 1.885416834 2.324917824 2.782511829 3.034275112 2.575934091 2.450839626 2.30078468 2.456304804 2.351571105 2.144541938 2.145798984 2.39187767 2.65891773 2.872904961 2.85402808 2.911499405 3.119107033 2.970444807 3.086330379 3.127959237 2.871898324 3.092640626 2.905379744 2.824575178 2.966832629 2.945100011 2.814996712 2.639147692 2.987003714 2.795823087 2.827597405 2.728189091 2.73293114 2.436683582 2.756347228 3.18684016 2.948344556 2.764851403 2.983053345 2.960612238 2.936038898 3.152875242 3.083514124 2.877166701 2.840982962 2.502142696 2.472153137 2.813574855 2.737480668 2.579348934 3.037744749 2.845394245 3.077169128 3.014927418 1.909010724 2.333109282 2.867092474 2.768362635 2.718136685 2.313177353 2.908645347 2.915042289 3.411468151 3.030250106 1.764896538 2.227323944 2.925093227 2.47284198 2.579762068 2.639589049 2.913962944 3.258207676 2.837870264 2.593407337 3.006880195 2.84320321 3.169006678 2.858936836 3.092189532 2.529721105 2.72354994 2.910046294 2.59156542 2.670392452 2.955107111 3.242881802 3.029946634 2.621347665 2.784372195 3.017412304 3.066365767 3.180580576 2.893517031 2.923333015 2.312153569 2.070594832 3.02720252 2.853861023 2.490533354 2.205927631 2.365407247 2.5218757 2.203097577 3.013936442 3.029228858 2.325190548 2.682310746 3.0462042 2.793812764 2.9940227 2.843316856 2.735798123 2.35272949 2.865172423 2.827959735 3.019397491 3.085977178 3.420386576 2.944018449 2.828896939 3.244896451 2.626754087 3.200649781 2.874804564 2.807722263 2.74466355 3.398938767 2.323101097 2.58393999 2.924234231 3.005339841 2.971898414 2.669943334 3.05490971 2.567455751 2.683890411 3.019898676 3.08888276 2.730987498 3.165731503 2.731410304 2.520908882 2.853741861 2.827052067 2.938250266 3.006363542 3.026036381 2.920118822 2.92235072 3.278585204 3.076102682 2.967496963 3.085765566 2.932789554 2.61683784 3.001284143 2.454994084 2.879698068 3.198183134 3.440906916 2.796817786 3.309509386 2.420846976 2.131493276 2.665094303 2.630372427 2.864548119 2.876106976 3.085185093 3.145130814 2.741114989 3.239013308 2.979435112 3.045884531 3.236024458 3.257922559 3.035666415 2.907620124 2.84482162 2.888888889 3.175111387 2.754063392 3.448682588 2.324366721 3.064663393 3.190074505 3.799184763 2.823350472 2.945944501 3.433567554 2.827971286 2.616999749 2.969778335 2.846216963 3.003313643 2.643769898 2.948182945 3.031357531 2.750796052 3.129378453 3.062124418 3.614529503 2.747547091 2.737760762 3.364975345 2.666543944 2.648888399 3.335996892 3.34283399 3.088079165 2.796698174 3.115467327 2.960939824 2.819459085 2.702240026 3.075047145 2.772160507 3.158640031 2.709097627 3.238780432 2.531529366 2.747593614 2.522399009 2.810312111 2.435368976 2.210819976 2.386956437 2.34515452 3.051798525 2.990094288 2.862684321 2.91918375 2.941516333 2.815027775 3.49610107 3.00181249 2.606887518 3.580973325

ENSG00000266967.5 AARSD1 1.120308442 1.360267849 0.935596968 1.14036887 0.912823604 0.717327311 0.79366984 0.876989662 0.80946386 0.850600765 1.192061967 0.992289229 0.846749039 0.787251774 0.85812033 0.861056529 1.077119179 0.916817636 0.921164902 1.065556721 0.973835352 0.933596334 1.069410428 1.010897072 0.979415206 1.023939302 1.261545972 1.048027244 0.86910828 1.075490363 0.969104767 1.097663645 1.061813258 1.149084924 1.313791639 0.965524093 0.992508783 0.655863401 0.931887082 0.787067345 0.833599978 0.869161087 0.825414617 0.830989097 0.54220261 1.109627562 0.876773797 0.726158311 1.114021056 1.198467885 1.339368916 1.628930792 1.46104741 1.699632975 1.188333837 1.496159838 1.53693429 1.007532714 1.537100798 1.413512614 1.383329116 1.639570123 1.15081258 0.861810791 1.035275448 1.125668066 0.681732879 0.956244397 1.155101049 1.276694678 1.230304529 1.413034374 1.291744722 0.997403359 1.21776707 1.398162181 1.472287725 1.470641675 1.369476238 1.281247598 0.991772639 1.032162669 1.204444298 1.101703707 0.949969198 1.144315282 1.117878446 0.966395266 0.979389932 1.142721325 1.039050386 1.111492145 1.020705907 0.977602143 1.186974868 1.229302183 0.861008289 1.171432253 0.923745712 0.792969377 0.81624638 1.126177796 1.170817689 1.069005157 0.986642648 0.865960488 1.033171368 1.072975515 1.05809666 1.1588729 0.874878892 0.7857335 1.01696562 1.069082105 0.909636026 0.814135682 0.796017371 1.145766848 1.12608468 1.060663118 0.928690229 0.782220412 1.043552542 1.004263565 0.820206954 1.107352564 0.872496661 1.005961423 0.838595103 0.795384177 1.118576904 0.84287265 1.411511281 1.239027745 1.254844203 0.782629567 0.907324751 1.185271488 1.223984763 1.233234337 1.120793563 1.431011956 1.01911386 0.926688767 1.016133669 1.142308316 1.086514438 1.085937523 1.048537472 1.160321492 1.268938047 0.986282069 0.95635341 1.112165251 0.940979865 1.054875522 1.311485793 0.938484969 1.358825354 1.600386197 0.863146337 0.889553674 1.229948415 1.611368334 1.786804971 0.976180816 1.388066256 1.413379746 1.286928548 1.258547253 1.741791322 1.513617668 1.529355051 1.440204275 1.056455205 1.458394943 1.463110132 1.217446634 1.077057724 1.615863158 1.814258377 1.30517905 1.476313791 1.713867625 1.308673203 1.238487695 1.327589895 1.151285846 1.252533514 1.690376322 1.454558904 1.370880301 1.294191289 1.278892133 1.184533332 1.301847003 1.129786406 1.600095488 1.545911828 1.05017478 1.160362953 0.985577624 0.921503561 1.009828754 1.096704121 1.233221749 0.993250298 1.327277035 0.828061012 1.032916263 0.995364778 1.181780727 1.063210682 0.973303435 0.844320996 0.834204218 0.664277191 0.844532585 0.969639352 0.823979798 0.858070919 0.894301192 0.87025838 0.955824447 0.754695717 0.929975379 1.326478793 0.932978979 1.07867963 0.732835891 1.108931938 1.403771708 1.114711128 1.227847873 1.073683244 0.923729582 0.817751256 0.945365029 0.938827185 0.719586562 1.450833577 1.115028999 1.531959972 1.044147794 1.391818472 1.438299279 1.09821528 1.050957157 1.260820253 1.186352524 1.408420021 0.950474056 0.937637031 0.990551105 1.142688126 1.105658098 0.919148023 1.517045125 1.161403789 0.969998054 1.048799206 1.296964476 1.20113193 1.237579827 1.836843026 0.967717936 0.992315253 1.36806226 0.890377057 1.100050264 1.291432046 1.02019983 1.551150975 1.282465643 1.298411109 1.392347713 1.392076056 1.02453871 1.360805347 0.952328835 1.272792226 1.645848094 1.757265139 1.38549807 1.641229289 1.291850577 1.515793574 1.218248038 1.190030112 1.108967973 1.51947236 1.508754767 1.183230855 1.354818124 1.377371788 1.462214072 1.39549921 1.388425516 1.377670836 1.430882802 1.542162903 1.596875105 1.160852348 1.598353445 0.728483616 1.651140173 1.087703122 1.465864402 1.521858841 1.053152506 0.80858002 0.93755148 1.092488699 1.42638872 1.21987855 1.128757281 0.91181171 1.350042872 1.638080727 1.062416151 1.467675732 1.302466628 1.729719683 1.577589555 1.327636835 1.367548845 1.465733757 1.772968064 1.577442022 1.728981407 1.554118318 1.622391453 1.598563192 1.538542246 1.552926629 1.377535543 1.2604628 1.486655965 1.279746713 1.192381643 1.656481909 0.999465909 1.437058967 1.347684153 1.259422169 1.579920235 1.425756514 1.564309268 1.028046326 1.52244131 1.800508451 1.785391369 1.743250833 1.397581865 1.419762844 1.538874739 1.326566443 1.260215416 1.274233902 1.220040865 1.753441354 1.461372503 1.548083015 1.365716805 1.050809132 1.364688824 1.399622517 1.396920816 1.07070555 1.235616039 1.122765678 1.525913014 1.755908937 1.665912103 1.084884971 1.199725845 1.753115636 1.326617848 1.741662189 1.684780764 1.725588895 1.75267708 1.488600649 1.64594252 1.537700471 1.303668447 1.820657202 1.5903101 1.656442542 1.697417569 1.346736274 1.224393247 1.379811189 1.329945146 1.763006096 1.841063673 1.310349515 1.551549453 1.666013034 1.55215446 1.673768466 1.644600175 1.255398252 1.366651108 1.574320394 0.89337875 1.975625014 1.006409602 1.065409775 1.51011749 1.676756831 1.614451576 1.805158689 1.635515593 1.698217529 1.271774314 1.564231418 1.915619883 1.515002313 1.947901981 1.411945427 1.529544978 1.688884417 1.586381626 1.580729056 1.541601121 1.719813185 1.817735189 0.939517855 1.494959227 1.642037701 1.735748988 1.573431117 1.40575512 1.395179833 1.277147294 1.635865835 1.667379977 1.940520931 1.610686541 1.577783542 1.516785419 1.271786674 1.51619751 1.457194453 1.58003536 1.228715338 1.605387045 1.374826164 1.702448349 1.516192787 1.703395135 1.490379279 1.553658755 1.442855427 1.872015345 1.526170205 1.702291633 1.626572672 1.752198913 1.787283932 1.459148651 1.4526341 1.516387339 1.151875169 1.462252877 1.247535328 1.363679034 1.399391022 1.72996092 1.268662917 2.033966088 1.095715283 1.366912388 1.503724327 1.446863975 1.452395302 1.251374486 1.704861694 1.725763835 1.619911281 1.951576363 1.346267304 1.540766774 1.635733556 1.698564545 1.85255241 1.455860776 1.527730281 1.22355457 1.785225639 1.678460814 2.676614395 1.794773632 1.684136274 1.947466718 1.719266085 1.091859686 1.814101304 1.701773544 1.314777087 1.305785112 1.767308066 1.326283047 1.614395473 1.18379324 1.558980977 1.662103737 1.544235105 1.645610633 1.601273675 2.107930466 1.623951882 1.867409423 1.401682123 1.437835175 1.486408884 1.530070569 1.6880231 1.691195709 1.709000381 1.843463812 1.673559158 1.533399268 1.405836856 1.374462357 1.450220261 1.426803099 1.635871498 1.107529362 1.447735735 1.267844551 1.567056288 1.269745494 1.106173542 1.221628739 0.988237899 0.885704393 1.560913794 1.725681435 1.360752836 1.421524997 1.573391034 1.270820987 1.627667247 1.233504834 1.007504565 1.623301701

ENSG00000157426.12 AASDH 2.196066607 2.301110223 2.550821117 1.853269059 2.405784788 1.827937498 2.361833274 2.373769665 2.577390865 2.420926007 2.337342746 2.480643999 1.879873834 2.008905151 1.989486872 2.450830817 2.410949188 2.042505606 2.611633451 2.684696753 2.370783419 2.464490158 2.545110485 2.261466784 2.515328402 2.47771637 1.969559289 1.699383742 1.916016088 1.577682923 1.597098361 2.09414626 0.999075448 1.746515179 1.950431758 1.80362178 1.47500233 1.256762376 1.479735205 1.796737839 1.623267511 1.875751708 2.028765402 1.716825172 1.709557093 1.549715203 2.207711595 1.760503406 2.257071617 2.235802798 2.570392986 2.156340592 1.878960037 1.762043239 2.514167234 2.026478424 1.585151718 2.028203049 2.073593321 2.027244094 1.958551423 2.081502889 1.778274949 2.241797504 2.464645079 2.533916374 2.432924279 2.192883246 2.253421494 1.913382435 2.20600596 2.497503402 2.429047505 2.234066678 2.467277535 2.557682617 2.961964631 1.968686419 2.450199354 2.510541239 2.301573529 2.391903119 2.297963988 2.364874354 2.286366597 2.457399338 2.002681307 2.093101218 2.275985849 2.08597066 2.169808603 2.279544042 2.342229598 1.985745742 2.103411384 2.420203942 2.329607403 2.370804625 2.456779163 2.362204188 1.984909847 2.187515145 2.295861111 1.995338941 2.343914013 2.49360006 2.690413 2.655812921 2.616209626 2.018994012 2.090137012 2.428773838 2.342986262 2.592515966 2.333685447 2.521740303 2.267500658 2.172630775 1.985967743 1.678010518 2.575299141 2.3378488 2.317236555 2.538597962 2.566992955 2.364873526 2.217028694 2.119224742 2.443008723 2.182004082 2.120374171 1.824715374 2.226135485 1.890157716 2.210512215 1.970231358 2.127184369 1.763127975 2.329133675 1.387967331 2.466575533 1.744101464 2.171107143 2.38376558 2.578118722 2.538740734 2.611270102 2.655827722 2.485108705 2.396548274 2.284534353 2.330629191 2.370221526 2.131720706 1.944188392 2.454562762 2.18217962 2.712697302 2.863967417 2.410726384 2.256357819 2.179845658 2.137966409 2.137884063 2.513821721 1.802493164 1.936797989 2.186042865 2.174654899 1.377872488 1.988543497 2.539593353 2.662707724 2.339791371 2.287987615 1.940508611 2.278771818 2.178228605 1.958929593 2.364911024 2.09257386 2.312690995 2.166201018 1.703214411 2.157694878 2.082773372 2.161263979 2.189132904 2.452962491 2.180985905 1.884327994 1.995066506 1.961665018 1.928386563 2.485313379 2.384539486 1.728893227 1.829907029 2.257012798 1.411548618 1.869201761 1.686028041 1.665153064 0.912185969 1.491829408 1.441838772 1.149802046 1.332829576 1.753736332 1.455294726 1.614417802 1.634575309 1.887918939 1.621328948 0.861310799 1.899917164 1.820478697 1.685457163 1.701701042 1.741171223 1.308738572 2.033167849 1.6137491 1.354714401 1.419731526 1.859737766 1.498543175 2.109491737 1.953863157 1.992777391 0.832463097 1.368391394 1.727870952 2.109008863 1.881385754 1.693573803 2.141159987 1.988556749 1.43448094 1.438789984 1.673462749 1.426500449 1.81779988 2.150889941 2.083497306 1.729740643 2.381320286 2.14474323 2.014016666 2.149652674 1.961704771 1.932505323 2.263248892 2.180509257 2.4341 2.314714672 2.267710945 2.181526338 2.083450381 2.300566232 2.252651443 2.566751229 2.021969642 2.243707713 2.346719005 1.924147706 2.069084359 2.112610205 2.127156209 2.318231764 2.195421383 1.716260104 1.144718405 2.061591471 2.182818026 2.12204435 2.083897474 2.286202038 2.189716773 2.258517809 2.156991531 1.632268509 2.327855834 2.27314311 2.374142512 2.16060317 2.262012326 1.966271291 2.379605011 2.500342203 2.146136581 2.221194841 2.335066072 2.04871631 1.928034444 1.819972937 1.58743816 1.72259707 1.758286491 1.865029508 1.832241091 1.888954622 2.014452214 2.327125428 2.339045552 1.998760002 2.113721556 1.751081976 2.2599139 1.633504866 0.622319613 1.389887923 1.165608225 2.49087218 1.936137341 2.00546474 1.723439515 1.684330158 1.724063126 1.252421946 1.404799673 1.665710189 1.85366585 1.854413313 1.913627729 2.063446866 2.264485279 2.140953679 2.091921752 2.072706694 2.144604501 2.274353274 1.972858785 2.036049895 2.469213136 1.944138842 1.6662161 1.832690467 2.265333233 1.462923121 1.703634188 1.919720965 1.79776767 1.292993064 1.413687045 2.302638808 1.75125731 1.650196526 2.358974286 2.072356997 2.022173357 1.885657117 1.815685303 1.593377372 1.995866084 1.77897806 1.850351765 1.72637411 1.569833495 1.93711027 2.462678792 2.075372896 1.802432655 2.379299643 1.645075986 1.940071997 1.765261482 2.276551175 1.816552866 1.641179252 1.960069511 1.487551802 2.067626585 2.070091811 0.999947052 1.785046593 1.929779121 1.589416871 1.938041992 1.811924553 1.985836175 2.23455759 1.908124999 1.846346043 2.22492401 1.943955821 2.112425456 1.674179986 2.345093109 1.487511207 1.914790857 1.923436719 1.563758658 1.92559288 2.167367623 2.260968764 2.118669907 1.698890262 1.744892888 2.235637796 2.050484523 1.710419728 1.949060105 1.976872147 1.683522693 1.581798995 2.889768099 2.210691656 1.236656812 1.785791174 1.413636781 1.779715824 1.447494907 1.858762563 2.277997896 2.022517779 1.742911632 1.860044508 1.694806096 2.068277391 1.958325763 1.742410825 1.638090657 1.816397803 1.957549707 2.420107455 2.141516617 1.994308069 1.63440834 2.238106092 2.06634663 1.968417073 1.986857407 2.030177559 2.167590006 1.704970101 2.185259638 1.746271313 1.407348916 1.645865046 2.049855458 1.90442502 1.779879447 2.08320067 1.813662437 1.519662694 2.013908433 2.306469177 1.787306423 1.893289416 1.701725919 1.201677404 1.951386333 1.930041581 1.771738936 1.997268122 1.783418295 2.197890592 1.600029763 2.342892456 2.473216329 1.892059458 1.945810942 1.496673924 1.912945151 2.358433366 1.760910458 1.879025379 2.343419155 2.184854086 2.028767939 2.313572646 1.324175683 1.466144241 1.767872226 1.680140587 1.688232952 1.949701972 2.159420916 2.09412566 1.839190425 2.080888635 2.310360019 2.311736057 2.2363189 2.060330803 1.592284451 1.927970123 1.971213416 1.87554745 2.131278732 1.988501641 2.747751691 2.136768481 1.901184113 2.021191506 1.958456494 2.130925073 1.993507634 2.166304949 2.014922301 1.901395749 2.013988244 1.892932762 1.840896623 2.219497534 2.153104112 1.633396794 2.014674185 2.408905147 2.104242241 2.042051165 1.497163115 1.574134212 2.345222718 1.92640347 1.723287663 1.743356133 2.197441787 1.867279429 1.894014619 2.260847533 1.801197548 1.943959322 1.995232994 2.131871759 2.330787736 2.358998143 1.895718025 2.142938953 1.694003829 1.991413984 2.351724944 1.985075953 1.487458919 1.389515796 1.535382958 1.428189585 1.991027905 1.844159474 2.268849553 1.859029175 1.957789186 2.198223119 1.678264413 1.972039075 1.706020741 2.21705808

ENSG00000149313.9 AASDHPPT 2.814033928 2.752087626 3.012404345 2.181467084 2.911477049 2.69605433 3.126783738 2.968885517 3.159221495 2.947646062 2.666694774 3.077976122 2.707343549 2.844787148 3.092547788 3.102147708 3.219565186 2.763943739 3.176668831 3.087419289 2.895699575 3.131027447 2.989850005 3.051054378 3.011099761 2.960359991 2.49645419 2.651473034 2.850262688 2.520638079 3.056530322 2.771822521 3.573263141 3.027387304 2.612648237 2.437838004 2.571201028 3.85570047 2.300861442 2.775610489 2.474472292 2.550555848 2.796370332 2.433596041 2.299461814 2.672780891 2.945562285 2.21312483 2.779429668 2.759889991 3.002905566 2.442062303 2.720906125 2.472198932 3.211877636 2.860773841 2.824323703 2.996454965 2.869508495 2.617554924 2.62242147 2.727862534 2.456839765 2.875026908 2.944461117 3.216182963 3.147375278 2.900865369 3.029183675 2.467038818 2.722095241 3.166261748 3.130701437 3.022906315 3.093828774 3.558704762 2.298945471 2.510071185 2.398016282 3.120525832 2.780803546 3.023665758 3.272683637 3.058676538 2.834553963 3.345865598 2.260131742 2.535825373 3.389024064 2.442119127 3.085848399 2.87149191 2.780550851 2.343615339 2.611911671 2.973972938 3.220448142 3.162291503 3.284982597 3.175599397 3.234555135 2.877199782 2.745294127 2.730230175 3.164397687 3.2266664 3.227381105 3.40058232 3.23871721 2.415446982 2.877370811 3.122681993 3.136099548 3.417291875 3.211320701 3.461472357 3.286888522 3.027996666 2.903799771 2.550692117 3.255092016 3.132575328 3.106082724 3.270030236 3.342830754 2.924644419 2.95666838 2.998844432 3.408101264 2.686642048 2.739825991 2.746548438 2.46907209 1.744253191 2.920388341 2.959274103 2.625591098 2.151201289 3.021491509 1.815268891 3.028029655 2.938509478 2.647301579 3.063714592 3.052393531 2.949978922 2.957818021 3.025552767 3.097769937 2.814879585 2.852414567 3.128831932 2.822650868 2.861129661 2.784336294 3.029175696 2.943506683 2.844733874 3.322741204 3.028692378 3.16317748 2.873374818 2.662063846 2.82581946 2.612745263 2.006061844 2.675921338 2.632151933 2.815346009 1.845982597 1.990162485 2.867718853 2.946377376 2.700820138 2.925645524 2.456861011 2.233236403 2.440934919 2.68974254 2.7739995 2.443312202 2.805639164 2.673622908 2.618587716 2.52106331 2.68278494 2.811364845 2.778069344 3.331203633 2.805358329 2.210059682 2.592884841 2.919683456 3.108645491 2.802137558 3.464319892 1.996295533 2.094853145 2.815747278 2.415710345 2.962967495 2.652355814 2.736321023 2.334960738 2.592991372 2.757467469 1.945985808 1.362755701 3.036240546 3.022884275 2.506039066 2.376863246 2.923356721 3.013820709 3.045566509 3.252233374 3.036040176 3.103089007 2.852424078 2.651611965 2.760821915 2.893925746 2.796021073 2.890703972 2.70559582 2.745876657 1.409999482 2.812734852 2.720889185 2.25874795 2.614179785 2.017349606 2.817530687 2.698706798 2.312877687 2.357673457 3.081880055 2.85711076 2.680320582 2.522362022 3.104449886 2.826000489 2.749946641 2.887594031 2.691576764 2.059413493 2.661041768 2.867756075 2.517486841 2.964064929 2.926524465 2.551013711 2.878564408 2.19677713 2.831927569 2.380299764 2.657143177 2.891882129 2.950922245 2.593477636 3.026371625 2.369279741 2.8161805 2.845011679 2.483795392 2.778153069 2.992062585 2.587727343 2.894691049 2.939591732 2.908976394 2.535117203 1.605609516 3.026473251 2.577423109 3.005560955 2.724815873 2.857758855 3.260882588 3.350564445 2.972585704 3.183264084 2.109871279 2.718456017 2.83935146 2.413831342 3.062511388 2.728134542 2.695537094 2.874220832 3.045216771 2.796153168 2.88643113 3.130818667 1.858720572 2.126167814 1.953206764 1.918047672 2.020743483 2.133838851 1.56819757 2.420668809 2.673863721 2.786075683 2.848187113 2.885180664 2.824186622 2.503145814 2.689429774 2.96493709 1.830425491 2.465187572 3.265886259 2.975697056 3.00480516 2.092889124 2.171570446 1.88393541 1.791245585 1.639181109 1.795911003 1.874201925 1.914816195 2.568179678 2.824773122 2.765235268 3.048777372 2.858607983 2.520289043 2.793533342 2.758567299 2.48891608 2.525752877 2.261808416 2.6663027 2.25549451 2.846580535 2.215590348 3.074296813 3.013440487 2.49393006 2.539256241 2.617942378 2.376813862 2.555063602 2.844234308 2.798382651 2.224416196 2.927147546 2.598715644 2.298813636 2.677608987 2.591781144 2.646452189 2.577104231 2.625312539 1.945861441 2.505916629 2.475554322 2.939801322 2.780311263 3.026304537 2.775883103 3.150237585 1.965943307 2.00119334 2.818099337 2.809073128 2.733007587 2.746191747 2.890345558 1.884573042 2.52652474 2.692195967 1.122087574 1.747527055 2.583024237 1.442324838 2.70531369 2.133272065 2.40981815 2.643235923 2.813699364 2.856252446 2.927937169 2.757366658 2.188355687 2.592642711 2.780540645 2.394288659 2.782041416 2.668677021 2.20953568 2.636754023 2.816181535 2.695833463 2.715779312 2.364446354 2.435169228 2.914899551 2.633923002 2.114341187 2.500294202 2.601340456 1.604397813 2.358437255 2.143317865 2.723428978 2.602262066 1.627879242 1.384294952 1.547232768 1.41620186 2.586374234 2.192844865 2.645215731 2.845197851 2.64198118 2.434747265 2.698214338 2.826126597 2.72844712 2.757295228 2.805766955 2.904226872 2.862967477 3.368434087 2.999494782 2.646866239 2.707294717 2.548294349 2.267952007 2.80159948 2.593807131 2.738982254 2.639243893 2.754915362 2.228579751 2.033946675 2.325977162 2.717746376 2.822012662 2.577147489 2.548136717 2.684827886 2.387984595 2.810718933 2.457552679 2.640399037 2.89965028 2.81489734 2.749072051 2.892606584 2.475260016 2.740335997 2.768068925 2.621234393 2.872536028 2.416555005 2.971851357 3.165569869 2.628817226 2.625703997 2.089528464 2.855775564 2.761297203 2.39436293 2.401489405 2.828573189 2.940559647 1.933920203 2.676158722 2.468091145 2.143922273 2.529286898 2.60998011 2.482285448 2.420789956 2.854646427 2.893980325 2.86262302 1.962596051 2.593947682 2.711413464 3.115732319 2.575822651 2.344971664 2.803845732 2.28538054 2.220401539 2.600059001 2.414693078 2.918305274 2.80887128 2.648269923 2.680739214 2.487310974 2.642310203 2.628551576 2.62802452 2.930254344 2.525839657 2.796864268 2.875834161 2.400142073 3.061703788 2.85381174 2.522002051 2.531549115 2.92951942 2.721018697 3.20679271 2.022672847 2.314517572 2.956248935 2.704916315 2.773756521 3.385192491 2.716233896 2.673675316 3.003840222 2.72613854 2.66449275 2.894320321 2.475825086 2.775008355 2.776244451 2.704710114 2.574164974 2.880346844 2.609827925 2.71428592 3.186205077 2.661770262 2.116505828 1.979736029 2.847363958 2.720852587 2.340593131 2.065294035 2.256940985 3.162846255 2.685514924 2.585840317 2.64114989 2.449678984 1.947580494 2.947391459

ENSG00000008311.13 AASS 1.750792591 2.411327776 1.960979172 1.274251095 1.710177148 1.699368186 1.25079089 2.155007617 2.459999693 1.997688429 2.054893569 2.193003742 2.098481095 2.002496155 2.325741407 2.533191536 1.915995806 1.170384265 2.153948926 2.166875012 2.788388455 1.97703747 2.112634903 2.191034263 2.593355749 1.782720336 1.652208433 1.312765061 1.164188106 0.964649221 1.143386175 1.485530237 1.056527024 1.676729825 1.716686916 1.441810301 1.289713154 1.775950659 1.550790457 1.96083544 1.563074115 1.787042119 1.686148819 1.78220178 1.322236161 1.364380942 2.140206537 1.38243271 1.748689571 1.948994807 2.329330034 2.074315719 1.737751395 1.747060167 3.057157246 1.382450332 1.294892554 2.55176369 2.866701593 1.805199428 1.353618749 1.907120259 1.476047756 2.023756729 2.398141882 2.226898126 2.081543661 1.629695536 2.142106761 1.088574437 2.206747353 2.252580707 2.477033198 2.747447046 3.073422933 1.996651297 2.756273211 1.85182518 2.484349446 2.046839494 2.542981817 2.136679936 2.470823142 2.009054641 2.296296469 2.063970815 1.125930917 2.356436187 2.286983476 2.683137138 2.25249233 2.13016634 2.076887596 1.689086309 1.275294935 2.12564874 2.760625517 2.514399534 2.283809229 2.205649363 1.660228349 1.651043984 2.532141512 2.120864956 2.376654074 2.212788738 2.636792249 1.824284231 2.644867415 2.083144087 1.738517708 2.553740871 1.422259983 1.895226009 2.355290135 2.063285193 1.75412191 1.683572256 1.49332362 1.042136118 2.660971683 2.095166548 1.251097522 2.923081013 2.377240761 2.162778155 2.283662711 2.001805629 2.297460576 2.017295185 2.222718455 1.739704596 2.128987338 1.157430529 1.711428156 0.853669295 1.888057556 0.967138776 2.327799584 0.43465738 2.416229011 1.976999806 2.564403945 2.956057981 2.432579267 3.441822624 2.655860552 2.695649934 2.643456991 2.54911555 1.933807936 2.578460577 2.392491343 2.343659489 1.749161397 2.349492729 2.078602337 2.585994212 2.073893947 2.550820908 2.63781008 2.102662659 1.81094604 2.20814931 2.315303615 0.654391925 2.8718466 2.660726504 2.452757528 0.867270117 0.69461798 1.796501514 1.99660112 2.054403728 2.123903444 0.97768859 1.056613732 1.753960056 1.859540067 2.193672487 2.243772512 2.000618387 2.060936355 1.290872329 1.606975248 2.045009623 1.958598355 1.802984736 1.86182737 2.350419351 1.101451333 1.621965549 2.520375261 2.534542898 2.844283219 1.898696601 0.708901329 0.95677986 1.943100913 0.942229162 1.388027639 1.630243661 1.194790856 0.906083859 0.936300613 1.458115291 0.8965371 0.714312374 1.342804834 1.417445086 1.304943608 0.920770338 1.529985927 1.48373152 0.723810862 1.35866165 1.315544466 1.230287611 0.902053016 1.190277668 0.941650239 1.776024442 1.650266315 0.890503777 1.644914541 1.248168954 0.713003064 1.913652724 1.698109163 1.889544972 1.123644611 0.808719138 1.770315195 1.721538923 1.747194502 1.059311656 1.988396191 1.247158357 1.519608335 0.726746277 0.88128023 1.561262904 2.020284507 1.87641901 1.560297216 0.8183985 2.014221646 1.68391521 1.897007725 2.292127158 1.651554579 1.734950868 2.048857896 2.809620112 3.022739854 2.278659268 1.497996453 1.530641948 2.040091582 2.243372267 2.243141441 2.857905772 2.503839466 2.52276636 4.394902141 1.58125095 1.868797237 1.881694939 1.380888345 2.150247663 1.981305479 1.601770858 1.17965698 1.77985485 2.322636448 2.355098827 2.178411926 2.844430901 1.677552909 2.335276669 2.146643912 2.436739257 3.751992625 1.626552005 2.363013167 2.089895014 1.562309928 2.339162564 4.076629993 2.199613214 2.481401544 2.855476451 2.372313407 2.050244478 0.607172008 0.781443007 0.715544204 0.850107235 0.470642566 0.837022483 0.779040847 0.660432984 2.03991294 1.755631465 1.886779467 1.969768934 2.33016642 2.010266433 1.818976449 1.643620274 0.777841412 1.420083501 1.414468283 2.925221657 2.26197443 0.819783021 0.682757098 0.661483158 0.588591008 0.452642741 0.873748447 0.758472249 0.575918113 2.220518568 1.456400282 2.370006937 2.00012146 1.878297971 1.749667382 2.146531481 2.359029888 1.580100677 1.534389072 2.069423996 1.797940427 1.982687673 1.036071158 1.437312706 1.881723876 1.193276156 1.847371705 2.208679345 1.045373791 2.106283056 1.384343045 2.300938289 2.666737964 2.281171386 2.045719947 2.215816988 1.258132715 1.916721911 1.740059674 2.322234255 1.779508827 0.668062846 1.101274547 1.353239189 1.698300747 2.638783041 2.492398343 1.933918682 2.19017022 2.279280084 0.615495256 0.301166837 1.570076049 1.2270826 0.756143015 1.051053119 1.712810343 0.997059044 1.805312623 1.727968954 0.541514931 0.643861745 0.70272683 0.715285483 1.263985767 1.005789895 1.547610318 1.383582472 1.565046066 1.66666455 1.669810632 1.88975106 1.768499724 2.123589447 1.212413449 1.512254741 2.293551946 2.157446949 1.72657116 1.973662034 2.05724116 1.654186357 1.804571472 1.391274757 2.516499116 2.21030505 1.742854788 0.800402225 1.841736836 2.324751061 0.615354582 1.046241092 0.996828109 1.310319723 0.881917864 0.520058926 0.73933865 0.450772894 0.491487167 1.230999194 2.558471839 2.158918379 2.217581369 1.701415893 1.423801781 2.173932793 1.78010118 1.658060364 1.587975221 1.869117833 2.068767418 2.56671212 2.500283787 1.626623172 1.009952613 2.338854365 2.209031707 2.396322756 1.91072633 2.349351966 2.124916219 1.919711095 1.83392109 1.685372106 1.137962078 1.526864696 1.795065485 1.4075456 1.398273067 1.74511123 2.28948014 1.368142212 2.410812443 1.879271654 1.85697142 2.000531032 1.491237869 1.248828314 1.493752708 2.47024268 1.823116225 2.098180892 1.564940905 2.439924526 1.341663987 2.45606466 1.267415972 2.237553733 2.258202887 2.01682289 2.339861067 1.777887971 2.053595603 2.057363573 1.776980189 1.893553702 1.487188297 1.829826026 1.668971492 1.068726175 1.777598289 1.133882895 1.50414983 1.898254135 2.587737061 2.463937608 1.475604561 1.619919967 2.630895292 1.318929954 1.110118532 2.243887919 2.201227134 2.549379147 1.934579337 1.866201213 2.012834622 1.095663783 1.701156951 2.642645379 2.227515151 2.287650843 2.551229909 2.295550236 2.677850112 2.34793081 1.957791692 1.838828664 2.760602719 2.380556581 1.920152734 2.233639131 1.614838421 1.805610425 1.788400351 2.476519289 2.099870186 1.505875709 1.408815789 2.330402472 2.088264356 2.169031581 1.345113342 2.288061014 2.457763109 1.643563788 1.406622346 2.645345956 1.924203242 1.363355395 1.376080668 2.016829268 1.522026848 1.839033897 2.699784541 1.721216072 1.487592708 1.944515193 1.772252739 1.950651856 0.655108802 1.120923802 1.386903223 1.62461408 1.793652293 1.353778213 1.768801882 2.12606641 2.486752367 1.910511233 1.098953953 1.522584166 0.971068042 2.406851396

ENSG00000275700.3 AATF 3.414740934 3.180977187 3.560115881 3.645503794 3.188973244 3.295681631 2.687907514 3.270415608 3.354286124 3.16020841 3.188220419 3.17834071 3.482175353 3.365355796 3.331429781 3.290752263 2.707223221 3.337515962 3.19578735 3.01271087 3.398954222 2.843306063 3.270355685 3.423434651 3.432539045 3.180192643 3.506509305 3.74833538 3.262179476 3.659645362 3.462796583 3.49357408 3.313216325 3.83602463 3.343110344 3.430645908 3.540276693 3.686347015 3.119571228 3.441067644 3.194840305 3.271806894 3.336203924 3.473999249 3.171530873 3.536042823 3.236282233 3.263632633 2.990826242 3.20511635 3.557345146 3.335824876 3.096430109 3.437748074 3.363610566 3.731031035 3.962875373 3.212711229 3.737597363 3.564252559 3.354297845 3.524638198 3.387488042 3.256145925 3.572879464 3.716857847 3.569817615 3.820326825 3.55102587 3.461013289 3.583918373 3.420125786 3.470960918 3.660028992 3.823666511 3.73790558 3.356709246 3.400120066 3.386402757 3.515728534 3.235970224 3.644370636 3.466523877 3.177975576 2.970859755 3.208816155 3.595267112 3.775354654 3.30436096 3.824449593 3.211961789 3.607844171 3.542207456 3.710566987 3.731941728 3.740809898 3.561084261 3.536059009 3.33828559 3.396906299 3.729775111 3.603730304 3.467797438 3.423455641 3.464543954 3.489715207 3.244844909 3.518380939 3.53200783 3.628935439 3.526556623 3.522350164 3.559785178 3.622062283 3.225309516 3.462507624 3.449934905 3.986056263 3.546284376 3.286859119 3.316854877 3.285302099 3.698180285 3.420964042 3.629998303 3.364132339 3.550121111 3.449798373 3.53500322 3.631546193 3.637891531 3.747645391 3.647207931 3.696960905 3.870243593 3.687598927 3.505583257 3.7772175 3.631384335 4.040524283 3.646939964 3.655324574 3.635253079 3.24756936 3.049551539 3.678972462 3.338993701 3.634942009 3.543086963 3.231774788 3.545269222 3.437556481 3.423599358 3.490016061 3.394547788 3.451450823 3.712092656 3.290902157 3.51072013 3.49837384 3.521689927 3.670388596 3.335392262 3.583941105 3.66083182 3.955934022 3.487798108 3.208589141 3.507833626 4.031614207 3.989627224 3.264719722 3.711750413 3.325878468 3.778932047 3.711259656 3.650890457 3.588811463 3.669536029 3.6263978 3.351844139 3.030754934 3.400290144 4.194660413 3.491251824 3.358447765 3.371476495 3.263427591 3.514744249 3.493160129 3.942378805 3.284705172 3.488712096 3.65610524 4.01706405 4.598502153 3.670516217 3.904761045 3.515900975 3.674299904 3.63886814 3.349019004 3.685477577 3.130592041 3.889813187 3.517078615 3.429821819 4.18498247 3.943625488 3.517081654 3.440364546 3.466891095 3.711283954 3.742730553 3.17465192 3.294352548 3.172770527 3.708215447 3.485697366 3.2791637 3.639026221 3.520940691 3.70391048 3.617772943 3.454381117 3.471584405 4.007770467 3.54410446 3.540308083 3.3016536 3.587952541 3.635792562 3.794940664 3.514728125 3.536873697 3.266845232 3.723867918 3.484240366 3.424615189 3.840364079 3.839673728 3.915829864 3.661051475 3.561564831 3.609226795 4.080639193 3.766383038 3.74862169 3.252613157 3.641384907 3.863602448 3.422857802 3.626690795 3.456132745 3.360510684 3.549463138 3.419309489 3.780065739 3.575535305 3.818291759 3.638262479 3.512490953 3.43685801 3.413912432 2.899476958 3.475336636 3.63179338 3.447605801 3.772741832 3.387269233 3.076958056 3.531316389 2.803670323 3.603955266 3.503070197 3.500405048 3.586448933 3.667480627 3.680804601 3.519381617 3.394665544 3.676279894 2.890701525 3.462778206 3.290051203 3.273641763 3.860226188 3.271905108 2.843586709 3.631325548 3.437217337 3.497558995 3.698036116 3.787016967 4.005359491 4.284785737 4.080781189 3.917191747 3.858464232 3.812044683 3.911224336 3.69662004 3.934026254 3.880040566 3.67864036 3.701539751 3.060827951 3.888823026 3.56798603 3.411994813 3.266812922 3.523652743 3.936368487 3.363025443 3.464046666 4.138196277 4.080787605 4.10366799 3.781555029 4.065582299 4.196939797 4.039205934 4.12253168 3.775768355 3.847195061 3.481445861 3.702226772 3.67818546 3.682468188 3.535220843 3.452703844 3.60796408 3.587740638 3.491829987 3.366536614 3.334375123 3.797333733 3.70907331 3.691321068 4.130346549 3.794481387 3.478234746 4.034453169 3.362484867 3.677943293 3.448518035 3.468504177 3.826988177 3.584225424 3.782098251 3.506970112 3.983526138 3.449851394 4.017136466 3.548283221 4.32184947 3.576686863 3.773860508 3.068449989 3.311748282 3.434559959 3.809150403 3.584724392 3.609859915 3.533746256 3.947403234 3.473808114 3.796266566 4.144726654 3.568824077 3.023488889 3.519913341 3.701466196 3.560125582 3.983722247 3.829107626 4.036986209 4.002479755 3.861132868 3.998103121 3.417992513 3.900436744 3.653319259 3.650680294 3.588789749 3.557194177 4.042391085 3.762577716 3.534348789 3.954487739 3.21044327 3.455438096 3.858078865 3.486221987 3.629202526 3.698529669 3.630819604 3.466442756 4.011837848 3.596891605 3.666442584 3.931774861 3.402144193 3.66381759 4.169462835 3.64754881 3.98314571 3.087781589 2.552276285 3.85409831 3.907752914 4.242785774 3.865348117 3.633965818 3.662566744 3.614693833 3.288045772 3.635121325 2.836641747 3.602164001 3.28471409 2.894746811 3.576907307 3.508738172 3.423157242 3.28562462 3.785822802 3.86787995 3.814335905 3.526151164 4.450345938 3.655878896 3.661796225 3.794236499 3.3830515 3.522966049 3.502588923 3.441395912 2.965962866 3.288622188 3.728590653 3.818645287 3.824110332 3.658634206 3.830394586 3.717823644 3.35441099 3.87969642 3.579016539 4.385205295 3.702038845 3.427542025 3.654626911 3.228895696 3.451644587 3.649992393 3.896327044 3.427527488 3.986271489 3.668894751 3.913868628 3.640645748 3.411523239 3.473085378 3.548887175 3.540023659 3.422739017 3.980678505 3.411343363 3.927895789 3.492375234 3.669301849 2.770789414 2.788419276 3.502702882 3.687036989 3.734611353 3.28007848 3.814743714 3.625039221 3.334256931 3.165733927 3.625482626 4.042564456 4.352185689 3.423036344 3.259750251 3.468894765 3.674172706 3.356256245 3.575628228 3.765460727 2.97346501 3.585361759 3.610399315 3.371939077 3.986644205 3.234527962 3.620220783 3.443734705 3.618940074 3.331002844 3.501055979 3.572245454 2.892280476 3.372755757 3.573143768 3.611921484 3.642984997 3.619494243 3.440438036 3.822930149 3.842328917 3.675461229 3.395563366 3.697783913 3.865439324 3.66252135 3.454269931 3.4339412 3.834121123 3.590872846 3.661707693 4.007565956 3.667232435 3.555381843 3.780901046 3.854809237 3.446683517 3.488419912 3.873950824 3.739278613 3.497940146 3.305191024 4.082996927 3.59818753 3.166265601 3.660214805 3.842547223 4.011301879 3.434045259 3.744657648 3.657114189 3.853264924 3.885858659 3.414870071 3.559761842 4.44995632

ENSG00000181409.10 AATK 0.948737428 0.753197701 1.299236752 0.91082132 0.619796475 0.851121939 0.426457194 1.064630365 0.829681498 0.747522743 1.401659429 0.342813751 1.601361462 1.294627772 0.695156887 1.065084983 0.716117174 1.13156798 0.701938269 1.034693865 1.006761581 0.572970421 0.850832523 0.943141981 0.952757961 0.864457027 2.052584616 0.946227556 0.423879974 1.274819076 0.789174954 0.360040084 1.997191685 0.693060357 1.043342521 0.787046868 1.31835148 0.45664716 0.488476464 0.636651098 0.835369257 0.740449395 0.90712803 0.658654922 0.41359153 1.11191687 0.633624069 0.148917365 0.815528975 1.057762282 0.704176772 1.333408935 0.917700034 0.418679546 3.39567551 0.64892984 0.300548457 0.298884442 0.684047142 0.245884878 0.483302313 0.549047391 1.319855303 0.432710909 0.451683626 1.564620305 1.13824859 1.307034569 0.695242788 1.384038798 0.910236448 1.399283803 0.252774003 0.461005141 1.064698206 2.692861289 0.654940555 1.667716562 0.940552774 0.421408803 0.978989331 0.58488279 0.456038218 0.691563745 0.420547776 0.23658277 0.781666097 0.95405513 0.448085157 0.169024567 0.612347869 0.480490808 0.402206093 0.847490777 0.396951284 1.078368113 1.198135408 0.360876758 0.398113702 0.727648314 0.227412681 2.395856181 0.741651553 1.449926598 0.416961062 0.530771941 1.199804222 0.928559857 0.777150885 0.734710185 0.515701114 0.420581294 0.481640302 0.704155528 0.571720995 0.764683168 0.293522055 0.537645249 0.837034225 1.106507594 0.45417903 0.813353572 0.3486096 0.456493612 0.562351774 1.26444874 0.50832307 0.378482586 0.258777592 0.40730573 0.255035046 0.537474138 0.286601659 0.567941136 2.062046209 0.305597777 0.466992823 0.765026621 0.537117545 0.533358268 0.754880167 0.948535377 1.755746323 0.449754784 0.451026262 0.542728978 1.030853876 0.469434605 0.900808967 0.421790928 0.395268549 0.302071711 0.678635076 0.762266898 0.527916623 0.494818215 0.711699136 0.510108089 1.086735367 0.373667734 0.324915425 0.460259765 1.086939811 0.779966048 1.725010927 1.139502794 0.629005074 1.451576143 1.078433915 0.931076757 1.510190083 0.580887129 0.721161569 0.343137846 0.285375413 0.377928133 0.636668954 0.583809759 0.691530235 0.949627937 1.366437775 0.757134549 1.140764017 0.970166147 0.872202734 0.816682984 1.332250459 1.100857645 0.313250417 1.14261864 1.142998762 0.636273663 0.55623127 0.70162951 0.777258934 0.635692344 1.368725985 0.668579565 1.947734839 0.990142331 0.633383103 0.616822997 0.572774322 0.428600449 0.502775786 1.420782237 0.253362284 0.776273398 0.474728549 0.487990082 0.91220388 0.736224087 0.569085584 0.703895359 1.498004275 0.32288462 1.203155439 0.895252101 0.561267813 0.745626395 0.454077751 0.886019794 0.286568706 0.882938891 0.507721251 0.65445665 0.82519103 0.939747467 0.711323358 0.439860534 0.230319809 0.270113559 0.501926624 0.871892405 0.818731987 0.460242073 0.407610117 0.889338349 0.798504027 1.247735778 0.491021747 2.235352286 2.281023984 0.627844291 0.973007628 2.080547886 0.3725961 0.421711439 0.646343328 1.003518054 3.497257986 1.816152353 1.646027241 0.732004913 0.33317686 0.746896773 0.556754048 0.856591436 0.722964493 1.332310623 0.396872341 0.592040133 0.644522844 0.404054226 0.861920092 1.302532164 0.48409594 1.057087038 0.690998728 0.905963422 0.367303954 0.468212518 0.681315982 1.257354177 0.544618647 0.384916908 0.483234088 2.554917013 0.206367359 0.789144857 0.353865739 1.702224999 0.610760712 0.542224765 1.021232713 0.781563115 0.433665077 0.399564923 0.444318952 0.736806816 2.581919116 0.602609893 1.64428389 0.420779074 0.811516456 1.317340495 0.42526641 0.805480644 0.604170743 0.772204733 1.178817978 1.995379048 0.776607641 1.011607745 0.253955678 0.948673886 0.726806767 0.516541419 0.470960759 1.221047866 1.032813781 0.604194925 0.547253121 0.668701771 0.474196105 0.775700397 0.947818933 0.678109585 1.477566027 0.945721727 0.701573174 1.242420788 0.814053207 0.670858641 1.024712197 0.721036991 1.160962638 0.595547195 0.991660773 1.311378134 0.673951908 0.652651993 0.427971479 1.197959971 0.62682146 0.543874004 3.680492624 1.266015559 0.311329273 0.243897226 1.183472357 0.851730574 0.885503073 0.370690858 0.537270836 1.567533269 0.732018707 1.652018931 0.858729708 0.525315406 0.29881329 0.879851715 1.090343344 0.213322918 0.802171147 0.628369969 0.851483013 0.355565365 0.753113853 0.25412886 1.150176614 0.79656734 0.564751852 1.066439486 0.742191801 0.772340347 0.479827268 0.832630586 0.934687114 0.136477232 1.208758797 0.651602069 1.607454596 0.715950572 0.250063741 0.651666304 0.265822787 0.462591068 1.24333319 0.682137826 1.102413038 0.94836013 0.803916214 0.743000168 0.838624712 0.991472009 0.993348074 1.196004918 1.093444173 0.612583503 1.586835627 0.502950258 0.348310281 1.039170223 0.470172909 1.529975989 0.731599908 0.405040089 0.703038179 1.075489149 0.808432296 3.034033414 1.232234035 0.808584939 0.485426109 0.58774976 0.255693483 0.202548658 0.785614226 0.418464584 0.391785784 0.590313706 0.596564897 0.747815801 0.73092608 1.01077831 2.289520359 0.317073822 0.529932016 1.232439646 0.977636078 0.936258871 0.564433339 0.952817756 0.569011659 0.814781312 2.663697256 0.986249865 2.080044785 0.575799926 0.953673649 0.926685865 0.762194579 0.863561697 0.306474997 0.780237245 0.881361981 0.307610909 2.304060199 1.015352617 0.669561839 1.805952489 1.144353442 0.822328837 1.451460084 1.107354816 0.415521352 1.328749126 1.381518253 1.788535501 1.71917177 1.322595103 0.590673776 0.887649312 0.550013555 2.925325527 0.484022584 0.154609034 0.901409793 0.447644537 0.750518567 0.646588536 1.22452251 0.356635297 0.666798963 1.108969783 0.842103157 0.876768043 0.449765572 1.688292341 0.709891699 1.0270487 1.08010142 0.54159893 0.560161046 1.62034426 0.436961925 0.73648184 1.229422771 1.02572953 0.923960611 1.197237257 0.885751035 0.413438274 2.086123832 0.849414842 0.435660663 1.182683817 0.952792527 1.061324975 0.932424545 0.239555226 1.523736063 1.128370907 0.616255427 0.391458021 0.484945762 0.925763029 2.508422567 2.108305106 0.407732409 0.845968131 0.698954013 0.268344055 0.547453932 0.684614968 1.497703459 0.776621227 0.80982156 1.071990878 1.282084506 0.496714196 0.727623696 3.102256633 0.766191498 1.140327116 0.702625183 0.31687602 0.991911062 1.649407874 2.90902752 1.040450785 0.774069194 0.618926778 0.913818804 1.373037081 0.592476801 0.92150091 0.727915098 1.103447682 0.454287919 1.284418911 0.838281885 1.104443265 0.470238788 1.017218486 0.528015887 0.461112233 0.77163093 0.768116499 0.682956441 1.765069351 1.082885786 0.916783649 1.087869535 0.712950341 1.191547038 0.968824507

ENSG00000183044.10 ABAT 3.517540335 3.078694374 3.232223644 3.118602513 3.686570766 2.85824432 3.354063294 3.502272102 3.2524722 4.746565371 4.121501474 4.752102155 3.083733548 2.370188959 4.318653046 2.707941443 3.815389576 1.333335153 3.993693021 3.9556529 3.987895156 3.947274501 3.449520784 3.922885667 2.983138769 3.607101576 2.317984528 2.248288885 3.660213818 2.776998606 3.262789305 3.130263095 3.051285111 3.183484371 2.646531597 3.756876659 2.96663161 3.306760292 1.016093229 1.701760831 0.972782391 1.967761017 3.860543425 1.006933514 0.959352851 2.671352572 4.491340533 0.896383173 4.047796651 3.931525459 2.699186471 3.271939272 4.236128536 4.280157218 3.457566785 3.095048416 3.560131827 3.987485908 4.176737923 4.598871439 4.449482271 4.008577659 1.186042849 4.747829527 3.850272176 4.128445814 4.224402098 3.078884847 2.153655152 3.625395551 3.607315675 3.960037357 5.353270368 4.231959196 3.065955025 3.609945598 3.345461892 3.39596528 2.797664436 2.424364979 3.631748689 3.163031101 3.496540403 3.22647102 4.897118973 3.243400557 3.094892499 3.619969309 4.279698492 4.825679869 4.354199582 3.193928652 2.957403224 3.636544458 3.472671888 4.09613752 3.220074873 3.964552652 4.116848887 3.530683387 3.049605567 3.89726262 3.955789743 3.653750703 4.540478231 4.922011768 3.687521333 2.991625669 3.804833348 4.292451804 3.205730407 4.053199409 3.913586954 3.764070446 4.41386606 3.481470732 4.389308358 4.132786454 3.174984698 3.436686519 4.223627326 3.096319534 1.420592298 3.207466248 3.730092081 3.976913588 4.288924181 4.665603946 4.101743987 4.03293006 5.037919915 4.057066432 5.450843331 3.24176244 3.130097997 2.491974112 4.93088274 3.69574794 3.578257143 1.738008967 3.383041325 2.746620069 4.605006486 5.297052637 4.274249691 5.613397645 3.299776121 3.302324186 4.330499771 5.499543194 2.935072379 1.701632412 4.495905983 4.36115909 4.410074244 4.441652376 3.231093209 4.317089239 3.858210729 3.796916526 2.653276852 3.730077709 4.396419101 3.575861112 2.564991243 1.356634451 2.871806414 3.380230584 4.241539883 3.074864562 1.547880925 3.990070749 2.562288723 4.752034776 3.863546133 3.743589638 3.759170623 4.354464988 4.078577067 3.780458938 3.803113875 4.146495288 3.399721485 2.227166192 3.669423451 4.609677087 4.098867478 3.574313133 4.341099286 3.657443698 1.422439027 4.516843516 4.386778406 3.389092603 3.515462685 3.26582068 2.733930248 2.671352249 3.835270912 2.248162457 3.583497939 4.384729455 3.32018761 3.246189334 3.105673399 2.792038187 4.338417613 1.729700521 3.302640973 4.06641212 3.774738486 3.177904915 3.093114619 3.14980548 2.593669534 3.192250982 3.74123373 3.381867969 4.503484587 3.879465393 4.595700081 3.591110604 4.571757886 2.572610078 3.587408454 4.33430238 1.811044236 3.468201087 4.011944627 3.679101618 3.256018806 2.712621522 4.242063487 3.994987425 3.903991744 4.23335794 2.14828402 3.930353424 1.884393551 2.950298801 4.058519284 3.687841683 3.073400524 4.060592668 2.612802502 2.178192194 5.141678161 4.00560088 3.42031795 2.13707295 4.528668655 2.837754068 4.019571277 4.630320702 4.3111252 3.259334675 2.544631257 3.184491874 3.834657641 3.763446722 4.86984704 4.785458681 4.842212192 4.684291249 3.287898721 3.485932583 4.06871363 2.962853195 3.682081133 4.125396009 3.470267266 4.599628015 3.090097256 3.041786822 3.676436147 3.895014175 4.821685788 3.569856103 4.355275454 3.666298071 5.613539667 4.28680346 3.099443493 4.010715326 3.486893843 3.922650087 3.458931123 3.228160715 4.340295787 2.9135529 3.756282524 3.00718785 3.639798102 2.036240041 1.92310723 2.794161301 1.963152576 3.39040871 2.771184356 2.745161864 1.874257528 2.630626104 2.802241964 3.446606352 2.750205081 2.218167857 4.070179547 1.689225434 3.160695852 2.090644373 2.050432124 3.291306674 2.36027662 3.560424222 4.339260953 2.061022989 3.182475132 1.894744937 2.570513139 2.163278498 2.038672364 2.269904863 1.959895305 3.678954023 2.562948066 3.75245168 3.475002315 3.467800488 4.090256155 3.941545386 4.810951486 3.296120705 3.71770611 3.246764397 3.94218874 4.644856 3.898089644 2.551350446 4.184267585 1.250217238 3.06384052 2.496794385 1.84963698 4.704412568 2.657069087 2.67419733 4.31646334 3.154957327 2.80900842 4.386807691 3.249122571 3.058774428 2.995883896 3.478148033 3.933974923 1.715334016 2.728852975 3.155228117 2.219561158 3.388286679 2.919389195 4.556700379 3.908348336 3.237238267 3.028270409 2.481910115 4.063674559 2.532665004 1.076821267 3.113008134 2.59223211 3.716894515 2.697056757 3.743212448 1.778326046 2.964984462 1.913589744 2.324476272 2.041690092 2.236723492 3.159366759 2.769036231 2.382709974 3.77458205 3.492739982 4.103762976 2.209899634 3.77379806 1.977066096 2.868822039 2.99811444 3.058888313 4.794271116 3.676917652 3.355199955 2.95857936 4.125329094 2.858394582 4.029127287 3.667432293 2.904254489 2.23354751 3.58278058 4.478380498 1.701853927 3.452900522 3.822505465 2.76678095 3.135532146 2.425715782 2.752810713 2.216456618 2.714093011 3.465660675 3.55281625 2.731228482 4.025070208 4.668743747 3.133723254 3.67766133 4.220025188 3.902000899 3.395749204 3.603730996 4.669732773 4.223230968 3.002346318 2.868760006 3.322407409 3.905483467 2.104316779 3.076719869 3.081944257 2.853575747 4.858612806 3.197749728 3.463313359 4.831606692 3.175394882 3.943854142 2.873716998 2.816551463 2.942203575 3.537013529 3.499868021 2.281222713 4.040112591 3.090096169 2.378356551 2.411847306 3.17855117 3.209003677 3.509030748 4.518266415 3.58621353 3.6080715 4.070205794 5.186435831 2.368448375 2.894435668 2.475901151 4.758480831 3.678054776 4.319411818 3.964771408 2.803226831 4.143027401 3.776167044 3.521180541 3.167147075 3.0054636 2.626171682 1.623001682 3.038987735 4.596212722 3.08930534 3.769862995 3.62519876 2.31617769 2.881495567 3.327699056 2.936297625 3.502673749 3.352385432 3.131645871 4.186234473 3.840464264 4.493537673 3.906526471 2.930282885 3.736231202 3.66115881 2.27547404 3.749121614 4.632933206 3.678895878 3.053339508 2.52417992 2.799451007 3.125348039 4.290556448 3.048315493 3.848391423 3.702965106 2.591421209 3.86557641 2.773664758 4.273688308 4.301718898 3.142165074 2.171489743 4.095570362 3.880937127 4.132796933 2.812954895 3.582903529 2.45966657 4.387597975 4.741513573 1.810409357 3.122792816 3.372845446 2.414114822 4.162244356 4.387622513 4.573824858 2.279270694 2.358776629 3.520312106 3.45180198 3.067994238 2.634868716 2.537860889 3.777153155 2.023214351 1.509044645 2.063035214 2.362837 2.988281034 2.965954691 2.981893694 3.345072512 2.934717812 2.25774617 2.02769277 4.045250102 2.012107498 1.363678412

ENSG00000165029.14 ABCA1 1.333923499 1.807036551 1.575071458 1.231427267 1.655160565 1.47288861 0.933066892 1.767347933 2.194015987 0.975723134 1.334600027 1.163939819 1.294266388 1.972280174 1.762446898 2.048917659 1.391870237 0.829013676 2.272893915 1.269270562 2.36515614 1.126906968 2.054211766 1.286849938 1.999801395 0.902674622 1.256472745 1.299611798 0.417562445 0.850889671 0.787689799 0.979173191 0.737568879 1.142498385 1.153442817 0.797309976 0.850011173 1.495854227 1.977806409 1.488596316 2.236526931 2.382043119 1.266734312 1.934085012 1.892577156 1.402449465 1.405290579 1.421267363 0.963008836 1.071772461 1.51289315 1.533536312 0.809472308 1.32899704 2.251361534 1.206225632 1.165479249 0.679068893 1.267243249 1.25299869 1.320935108 1.177001833 2.654614143 1.755132704 2.734521115 2.678173639 2.526977244 0.928402588 2.268273782 2.25522874 2.355511835 2.046944936 2.137199093 2.199588715 1.943896156 2.33909157 2.189025252 2.497091423 2.451540479 2.371397369 2.257346021 2.726997758 2.616992803 1.75020439 1.327661292 2.369407827 1.30794943 2.449797935 1.760287197 1.763378492 2.243175966 2.454102288 2.045547042 2.037916521 1.748571166 2.209540997 2.922737778 2.120190656 2.38083167 2.445961662 2.274782553 1.633828622 2.199133047 1.445784175 2.013907026 1.952527605 1.96182094 2.6040532 2.688012014 2.535832292 1.779125396 2.433660061 1.526483433 3.03061327 1.633212542 2.747913339 1.9262074 1.999381302 1.699664639 1.500180446 2.405903563 2.970268596 2.018816112 2.692609947 2.702874203 2.167098587 2.107773725 2.177124689 2.632676452 1.972214764 1.871174985 1.274265118 0.902486913 1.017698279 1.371686588 2.267674413 1.256953935 0.848570303 2.201146815 0.596435925 2.783910809 1.620858391 2.422460205 2.211097912 2.126049636 1.63916473 2.344898672 2.854578189 3.04062994 2.013197074 2.080189324 1.156347118 2.046447098 2.809792949 1.247482241 2.679748613 1.915423339 2.153873086 1.66127934 1.696953128 1.375100073 2.086155656 0.99587131 1.47167795 1.691270265 1.344593954 1.384933377 1.868488834 1.735749338 1.014817546 0.37189251 1.106275694 2.148029818 1.156970059 1.601634248 1.13929106 1.154938236 1.28760235 1.915695162 1.431455421 1.501310485 1.126339345 1.52078683 1.716637904 1.559594941 1.507377079 1.236430231 1.807306854 1.609892407 1.473073686 1.160799495 1.077683793 2.158888085 1.498050068 0.93985961 0.669914378 0.523702334 0.221261742 1.485728957 1.188211314 1.056189278 0.903521664 0.721981366 0.99334439 0.994417303 1.24375571 0.38951174 0.855174885 1.155892149 0.757910298 1.127270271 0.56215916 2.286668281 1.343418931 0.485703516 0.883674094 2.259643974 1.526696768 1.416198871 0.800981111 0.801907282 1.38439526 1.097066239 1.562232032 0.992951702 1.479032379 0.776624315 1.563281984 1.206064578 1.641121905 0.678541831 1.205344325 1.233662517 1.442306515 1.730378486 0.708442623 1.403466199 1.245815531 1.607907143 1.13037469 1.540232587 1.998402619 1.136535504 1.659281271 1.472425669 0.633925223 1.45012965 1.775986823 1.665144598 3.503591724 1.427917153 2.560242654 1.817942482 2.443189587 1.903643579 3.111637687 1.668379174 1.848070343 1.903764895 1.338403432 2.60065122 1.649710242 1.642212772 1.444892616 3.956812125 1.420833374 2.081236452 2.073042686 1.755098412 2.471556947 1.774912892 1.694646846 0.665478497 1.834579592 1.739331824 1.851729673 1.823553702 2.217150629 1.575365294 1.425322167 1.384750603 1.823969517 3.546232415 1.363656997 1.375128399 1.802175931 1.866209812 1.625004328 2.598929367 2.774364577 2.473115201 2.273101488 1.514603737 1.122325811 0.654094203 0.85846698 0.475485511 0.781887889 0.845955757 0.970050618 0.725928723 1.05370612 1.00911852 1.488845943 1.149281634 1.318966045 2.153077136 2.449683232 1.515018696 1.485244585 0.504088158 1.455135442 1.231100766 1.163832787 1.317459788 0.799516499 0.905839427 0.777232577 1.035162232 0.514821863 0.639849026 0.971478238 0.787698141 1.398017272 1.752931351 1.481591417 1.749389922 2.123014043 1.364754536 1.317494684 1.228095592 1.243874559 1.420049083 1.280688087 1.654233597 1.075963351 1.162130342 1.172136137 1.675485374 1.175266124 1.237457623 1.889268255 1.621240762 0.964338202 0.964729678 1.774143974 0.925728428 0.98886696 1.874885393 1.539963738 0.521170783 1.168445238 0.770662471 1.150938577 1.466507801 1.179442964 0.937768265 1.137007858 1.806098109 1.328291023 1.841233172 0.941903011 1.120883408 1.254830733 0.545250299 1.015605981 1.45245104 1.655758491 0.36435118 0.601182614 1.335352137 0.848156016 0.99983353 1.526944777 0.468910348 0.848016721 1.258447492 0.970194055 1.203676364 1.005048162 1.184626642 1.079825861 1.321549933 1.213836732 1.564122679 1.698064491 1.020025418 1.201773919 1.002487781 1.325604112 1.547421274 0.707454871 1.05224302 1.244133589 1.600481151 1.324998011 1.091499336 0.862465824 1.581956824 2.160436699 1.227066087 0.806554818 1.184282339 1.295145187 0.733239385 1.019186141 0.256126955 1.179948965 0.750034488 0.476769778 0.65843467 0.300259894 0.535180781 1.473810392 2.033642171 1.783364737 1.458660553 1.094699563 1.164780538 1.768820246 1.435393463 1.079304895 1.326627739 1.358879726 1.064738543 1.444607694 0.821433915 1.660515241 1.2851717 1.794977018 1.504998084 1.432950142 1.410266153 1.330943635 1.280981318 1.221637811 1.287871527 0.705263422 0.825050062 1.086953771 1.382827865 1.5961646 1.564964565 1.086525442 0.976560418 1.115195941 1.859120361 1.944097138 1.496259376 0.788313201 1.347109132 0.967615396 0.62530146 0.81133978 0.99285112 1.439497384 0.77199469 0.994698987 1.141653344 1.246772921 1.970917946 1.937328182 1.610702576 1.947263943 2.352053595 2.981517599 1.735990464 1.831155042 1.511807772 1.564856436 1.203761483 1.844710198 1.423617362 0.803347494 1.134973795 1.040970801 0.405135461 1.336085399 1.949617442 1.419755203 1.024650779 1.38653645 1.061566138 1.99165529 0.812251425 1.756822932 1.148438038 1.172690506 1.212062575 1.179755924 0.9121624 0.679405491 1.891701159 1.255687839 1.366126442 1.558861267 1.577092047 1.538151216 2.275826238 0.921001633 1.09538182 1.785555015 1.716816935 0.948910858 2.154068214 1.870871794 1.313935807 1.72496676 1.104075039 2.018454926 1.986814008 2.284190451 1.523380794 2.096807159 1.509469568 0.610299546 1.292814112 1.672283201 2.056102836 1.462307166 1.610780025 1.535078739 1.862128361 1.488718501 1.227255454 1.262959693 1.799104737 0.9665749 1.690425611 1.980301642 1.547722082 1.02239068 1.082204519 1.812942148 1.385453563 0.925350197 1.749698862 1.64448708 2.008410876 1.606138193 1.722638478 1.376535483 2.193709038 2.809783771 1.236074103 0.785519712 1.069170855 2.181988174

ENSG00000154263.16 ABCA10 0.189519777 0.433501705 0.127691197 0.082054494 0.102988965 0.400243189 0.04981812 0.116232564 0.143120541 0.095429563 0.240610438 0.032600818 0.160807426 0.102292722 0.050216403 0.256367858 0.294037227 0.706007196 0.21411154 0.140587145 0.137879043 0.256007689 0.098754373 0.10532447 0.277574672 0.11035589 0.342527835 0.093854714 0.06563081 0.172447812 0.082175735 0.195780067 0.268815208 0.022375425 0.2587622 0.105870587 0.107564052 0.02435233 0.164333874 0.095969114 0.236784707 0.267147524 0.052112384 0.137772391 0.119823507 0.129068665 0.074766261 0.147848013 0.245827955 0.203449791 0.270322356 0.447784244 0.165876712 0.328965974 0.363316911 0.152971949 0.287532061 0.220823533 0.138275511 0.130611234 0.145622323 0.170701596 0.168784223 0.094132789 0.160263912 0.119133142 0.199457387 0.044425522 0.350655558 0.169251557 0.166737657 0.216418761 0.155609313 0.123989413 0.302322483 0.097118719 0.276931303 0.063925637 0.032554071 0.185288256 0.221533879 0.171755311 0.128321919 0.168493274 0.127554617 0.155294444 0.051231794 0.109358922 0.060488545 0.158433112 0.178079861 0.149775643 0.03716677 0.086814886 0.096121436 0.151806163 0.126053293 0.157708301 0.161981466 0.092726534 0.021944905 0.143741767 0.152304409 0.160858346 0.092458022 0.141812555 0.142615352 0.065079469 0.134457247 0.059798079 0.090927549 0.181487869 0.12789085 0.102568685 0.106395403 0.079260257 0.041527936 0.075968509 0.334956101 0.012498228 0.133427453 0.138593202 0.015027869 0.244153148 0.137471824 0.090482187 0.033050498 0.047063234 0.08409406 0.062526684 0.079504726 0.065147667 0.269643284 0.059894801 0.032086756 0.029757112 0.057579343 0.098468197 0.144214764 0.129114899 0.130992008 0.204847996 0.15174275 0.171325444 0.111390817 0.174548448 0.21765031 0.126033303 0.190215205 0.195857067 0.065830785 0.126590232 0.14924946 0.225811749 0.039062989 0.102870783 0.156628845 0.218088584 0.096204546 0.37021725 0.221926971 0.292753845 0.151350812 0.284874818 0.550519177 0.171850267 0.0862306 0.892737427 0.318857769 0.115030477 0.122869542 0.479342457 0.178437166 0.306253414 0.144434747 0.251925754 0.144499547 0.220463042 0.253121534 0.27912346 0.294578031 0.193866252 0.398193295 0.132151197 0.350748134 0.305508073 0.143118491 0.230514197 0.193115634 0.17897632 0.075646366 0.228464432 0.162724137 0.201080787 0.180470473 0.185879325 0.156662702 0.092640535 0.369126157 0.106883427 0.061875383 0.123131413 0.03621547 0.144189275 0.193292045 0.120770895 0.062202055 0.125485757 0.060909139 0.080517668 0.122551039 0.16856388 0.080069364 0.146084664 0.218130822 0.022791464 0.063393739 0.033169054 0.037157615 0.035457234 0.033486651 0.044338555 0.026933077 0.073500057 0.048637685 0.077689152 0.086710929 0.06968372 0.061296508 0.033399292 0.075416082 0.231437297 0.031413236 0.131557242 0.081732 0.089248654 0.028244226 0.065094372 0.166656725 0.019714093 0.074799132 0.246204521 0.180645004 0.094799797 0.242922776 0.105953261 0.061637797 0.073254348 0.099177016 0.341739623 0.34700845 0.089901888 0.077919114 0.074333639 0.078770918 0.078076375 0.039706024 0.049934862 0.036568582 0.034495942 0.098887788 0.149808474 0.116147636 0.311508891 1.06097676 0.135859516 0.019876911 0.187636261 0.041642518 0.165687344 0.097642346 0.125153139 0.119916784 0.11329863 0.081091001 0.150788739 0.194556341 0.120727091 0.155611555 0.166184077 0.249194672 0.21185465 0.871461008 0.195437559 0.39916711 0.133537067 0.171649239 0.182759595 0.251934972 0.18488941 0.113101931 0.157297848 0.082638041 0.092212684 0.14756858 0.043436474 0.119115653 0.166452374 0.221567977 0.125039526 0.171409187 0.122337058 0.154678232 0.140011809 0.109629649 0.167096561 0.176425679 0.265494934 0.106002421 0.287567097 0.114757705 0.041885438 0.140606007 0.147268677 0.144625134 0.091726297 0.103503908 0.144305295 0.09685091 0.136730964 0.30447786 0.216073922 0.118604443 0.129772902 0.187293542 0.149934763 0.096906119 0.209396837 0.219915443 0.224159023 0.294693697 0.302072177 0.258637768 0.379388008 0.095720983 0.118191233 0.125958296 0.156346854 0.10010032 0.138589417 0.104767135 0.033491469 0.188363448 0.213652549 0.176624177 0.437466527 0.204430455 0.438339014 0.127254419 0.226880972 0.787954761 0.215169563 0.181187528 0.085488069 0.174938539 0.785000959 0.096063701 0.118005977 0.078758 0.039870972 0.395170234 0.186071951 0.112216165 0.113029512 0.065860937 0.075503851 0.192110995 0.088334262 0.089518457 0.17452978 0.30997587 0.132821566 0.126400163 0.345130768 0.039888397 0.118889671 0.143637137 0.088545116 0.129722008 0.19134183 0.172924003 0.078304092 0.151745075 0.45858761 0.279180599 0.202406648 0.047724397 0.108737561 0.200208549 0.162665286 0.108661492 0.035221119 0.141217535 0.179379171 0.257297061 0.118995204 0.18914938 0.30722972 0.161115457 0.133272744 0.21777148 0.044267268 0.142203982 0.168236507 0.103078522 0.17000297 0.088400437 0.057165353 0.141403104 0.523726738 0.233287502 0.122002964 0.233766145 0.264511095 0.238641973 0.242877871 0.144536582 0.203773166 0.230818037 0.40694138 0.293894176 0.19486743 0.343040162 0.518150951 0.166834707 0.190354693 0.125982681 0.139036371 0.138979485 0.281867358 0.162843526 0.339371945 0.198973137 0.190494353 0.175448451 0.115328474 0.093322819 0.216171747 0.326395868 0.346291556 0.226091228 0.308859575 0.079160353 0.189943821 0.373778892 0.25859704 0.155277734 0.135681661 0.529225385 0.095067784 0.200744911 0.272576248 0.279511335 0.198261831 0.136243587 0.160914923 0.118646572 0.233322656 0.150926072 0.22608155 0.312792826 0.375670164 0.15681939 0.332221126 0.185280167 0.208879777 0.348925302 0.069497604 0.238707071 0.144321327 0.120242906 0.242102821 0.124900858 0.661245273 0.27144322 0.116614596 0.190730249 0.169898631 0.500291299 0.157843466 0.318497892 0.071819681 0.16702128 0.104315976 0.058173039 0.131080116 0.381471779 0.231743163 0.156999679 0.16051117 0.204171217 0.119025217 0.430001341 0.206309513 0.23447798 0.225428393 0.022295406 0.288496745 0.152011662 0.098172716 0.127879587 0.118608783 0.17243019 0.288601035 0.188489551 0.205469292 0.18202413 0.271892176 0.333511867 0.203400417 0.469392773 0.345149976 0.269164676 0.112408019 0.199486931 0.139578276 0.051155806 0.194675817 0.298712405 0.418956466 0.215877393 0.365265623 0.169167182 0.110170713 0.197460334 0.278759674 0.285062478 0.188966291 0.226646617 0.092260908 0.218052003 0.113436514 0.27985016 0.200305776 0.091720874 0.187206057 0.170854075 0.100602639 0.253986539 0.234918613 0.17560029 0.102689114 0.44327345 0.162597297 0.128657493 0.17118008 0.094810461 0.147368697

ENSG00000144452.13 ABCA12 0.112660193 0.212553453 0.13552623 0.063551249 0.11004446 0.05692866 0.057652149 0.149173696 0.138212623 0.065818363 0.075877398 0.083563738 0.148730627 0.083794023 0.081291568 0.145205875 0.110434423 0.035746944 0.229746561 0.058251828 0.193115311 0.101505144 0.059578132 0.190927969 0.243868898 0.068718143 0.13293312 0.071638629 0.107917307 0.037822433 0.047030178 0.21831424 0.039031663 0.05915639 0.101738941 0.081691805 0.036016713 0.041062068 0.02164319 0.235795558 0.033746107 0.030144555 0.055179944 0.013663102 0.027615328 0.050748831 0.135337866 0.026715993 0.044317304 0.10359969 0.377312411 0.188057433 0.023182963 0.03006275 0.026312048 0.015573174 0.003152368 0 0.022847012 0.002902504 0.043780329 0.015470039 1.196916159 0.011379681 0.051892023 0.011325261 0.042754786 0.003352137 0.300948025 0.023772835 0.013774107 0.09075812 0.024693547 0.02455522 0.099122435 0.026416956 0.029321324 0.05143309 0.155409581 0.054831424 0.125039196 0.034473372 0.079242253 0.067136974 0.004571368 0.051019955 0.039791826 0.053598417 0 0.010272282 0.091396778 0.026409862 0.469222369 0.023407801 0.016801533 0.033892996 0.097733089 0.080362797 0.05201892 0.097650981 0.102374239 0.077814492 0.0675564 0.088700376 0.021152362 0.042046967 0.079908031 0.10041173 0.04731232 0.02771809 0.075000504 0.062500338 0.043118898 0.127549206 0.070710082 0.062274789 0.041192657 0.024086947 0.01149297 0.309721465 0.051704964 0.058319587 0.082969207 0.063351767 0.102863715 0.073685599 0.038282639 0.056526733 0.116930092 0.028241912 0.024117344 0.019693547 0.012647781 0.02703961 0.106958154 0.050709623 0.019838763 0.02084058 0.023869759 0.009244655 0.040942099 0.027797881 0.109123952 0.058062903 0.106396422 0.022511719 0.20881262 0.253759479 0.032627098 0.030049837 0.025215351 0.057875435 0.056021179 0.020962815 0.071602497 0.051420821 0.024816393 0.015676274 0.94358611 0.022929717 0.050219083 0.011898132 0.077039944 0.077598843 0.120177391 0.007065656 0.011286994 0.121991454 0.065546281 0.009684159 0.007612073 0.015676304 0.015614023 0.013777329 0.007802018 0.024363621 0.053270399 0.03414417 0.015354044 0.051166051 0.051363898 0.077657534 0.069161539 0.093936424 0.009216457 0.00348694 0.031877984 0.098009588 0.011663862 0.100771809 0.012940382 0.059325161 0.07609804 0.073193337 0.05432012 0.012593759 0.038029201 0.007099707 0.084633729 0.068136773 0.005928154 0.014888659 0.064030164 0.027927757 0.062535413 0.035341362 0.007716291 0.029574301 0.040021759 0.039975541 0.054865025 0.010151931 0.130741548 0.008826499 0.017127483 0.047070752 0.027145075 0.054260235 0.043437891 0.053909786 0 0.194742309 0.017541701 0.045861349 0.012213589 0.022325975 0.021594146 0.078025967 0.066005447 0.327311401 0.007658438 0 0.025754312 0.026740249 0.056288285 0.006832277 0.074426876 0.026595393 0.013453848 0.005053472 0.014610568 0.037628854 0.13387795 0.018788102 0.064153681 0 0.022721309 0.004618637 0.042493731 0.055304851 0.007675744 0.124108766 0.05395191 0.008759357 0.068548779 0.123891343 0.646875133 0.009042344 0.057335187 0.070180056 0.005066962 0.07232137 0.026095364 0.030911201 0.499725091 0.045973653 0.131628054 0.065201277 0.054092154 0.041796546 0.049247358 0.038386176 0.005862586 0.059338743 0.01849523 0.01567939 0.057419987 0.01984545 0.042696653 0.032388338 0.01940493 0.021855026 0.258218955 0.009428457 0.047752216 0.026994581 0.12130386 0.004569601 0.166879844 0.044085514 0.112845705 0.128926521 0.012597957 0.066445535 0.002664779 0.015060449 0.002768314 0.017719899 0.02176057 0.013494094 0.066670868 0.02199147 0.021099251 0.01006055 0.13214672 0.01534604 0.130064278 0.105794703 1.042297694 0.041392188 0 0.057213778 0.022887529 0.049662349 0.012019936 0.011227735 0.021130182 0.016493958 0.074229669 0.019076449 0.015634181 0.022234917 0.023288336 0.035046619 0.156709855 0.0357178 0.0363791 2.977955997 0.036780014 0.041691064 0.031210794 0.017038692 0.01982548 0.048323625 0.287403216 0.009132946 0.02256232 0.039650833 0.006366984 0.030232962 0.015076813 0.029932508 0.014890301 0.006409424 0.006315103 0.106006027 0.037936503 0.018295133 0.039050484 0.10307379 0.011506363 0.043480389 0.024410631 0.005231529 0.047505195 0.007313668 0.049266187 0.019468673 0.010285539 0.006546282 0.141431113 0.015467664 0.084821998 0.141508108 0.013305561 0.011028422 0.006311195 0.01617177 0.012436844 0.011780739 0.03709582 0.015834515 0.100615727 0.026241694 0.004505751 0.0665165 0.002641005 0.052336626 0.011829718 0.080102221 0.007666568 0.42030639 0.03048262 0.015217445 0.196422006 0.027021315 0.035009385 0.014330571 0.808415934 0.016610754 0.243996328 0.007934721 0.004407616 0.023214074 1.793544487 0.013121354 0.00590294 0.007008948 0.041483093 0.061007285 0.084570308 0.006160175 0.096123102 0.036047417 0.003663716 0.013937015 0.007378964 0.029441494 0.007779773 0.007806902 0.007514434 0.100820344 0.030353491 0.036553142 0.182094206 0.040739938 0.049404273 0.021156819 0.031022945 0.028017544 0.021062077 0.019817464 0.022030429 0.026893095 0.07290874 0.04345058 0.022859512 0.054239964 0.202194112 0.014107519 0.421057557 0.036514913 0.238384236 0.013569328 0.032927631 0.0285693 0.660461748 0.009222361 0.00679524 0.039758251 0.030402403 0.002792135 0.059905513 0.046516036 1.167878115 0.011192588 0.057872843 0.060174909 0.062911311 0.006036919 0.035406404 0.010263907 0.027575018 0.021261587 0.003746386 0.031973746 0 0.005128556 0.135457274 0.005131541 0.023196556 0.048214865 0.054808668 0.027525241 0.01233144 0.10664912 0.010226431 0.037637216 0.46884316 0.01847775 0.009297653 0.024138247 0.043024039 0.019269265 0.0243479 0.019231307 0.008851726 0.082315596 0.100228616 0.457271481 0.090035031 0 0.041939349 0.103491612 0.025014314 0.026243348 0.035955865 0.004817786 0.028117796 0.076729618 0.094709976 0.006139762 0.086770745 0.034159667 0.008240812 0.040899884 0.024854807 0.028511819 0.019895694 1.177160983 0.014483277 0.050344098 0.055401539 0.045825795 0.358645621 0.053577369 0.049011437 0.024714166 0.020569678 0.037734224 0.07377881 0.023463297 0.041114682 0.165047352 0.015197005 0.025302258 0.040109355 0.010298899 0.016215951 0.018219734 0.014922946 0.052645735 0.084844552 0.008859176 0.008189356 0.011048286 0.070745729 0.019646109 0.027553474 0.230668928 0.016712324 0.189913068 0.020622027 0.033263762 0.010983913 0.004687958 0.038561113 0.034853705 0.022877838 0.08595112 0.055473335 0.262295155 0.033137604 0.04064107 1.036445406 0.004907853 0.019308746 1.899055277

ENSG00000107331.15 ABCA2 3.308036334 2.888328426 3.341352238 2.959948416 3.054427129 2.763102612 1.285425736 3.032864105 3.058553544 3.228528706 3.216382197 2.764310842 2.723719613 2.779681161 2.702706173 3.075058096 2.33067292 1.880217237 2.881315656 2.557251865 3.33898706 2.837501214 2.836753005 2.641982777 3.251491851 2.436014746 2.961939293 2.414901674 2.240449354 2.409850897 2.704439808 2.362257918 2.751747962 3.088337446 2.67547293 2.28256612 2.640885349 2.851889284 2.157380258 2.633884432 2.232181849 3.189148034 3.048412161 2.678882176 2.217136449 2.745124812 2.931135001 1.926702043 2.175628316 2.895806885 2.573862872 3.317853169 2.564320385 2.61218011 4.057055771 3.421325635 2.370618837 3.064046642 4.20118284 2.506456043 2.456533998 3.474317858 2.639065857 3.746843296 3.263618216 3.717912254 3.963379059 3.084723205 2.815443072 3.763596512 3.611752123 3.37682327 3.254430785 3.250075369 3.309143507 4.002691114 3.534381216 3.702071141 2.680958231 3.006199499 2.926577677 3.601000748 3.122417865 3.432980507 3.273493533 2.938793346 2.691276889 3.439662135 2.626707279 2.465435927 3.08382119 3.262843344 3.369405576 3.589890172 3.640151301 4.049046066 3.757406693 3.414088027 3.421743043 3.408285907 2.982728706 3.845215868 3.476154275 3.7007204 3.321706745 3.300369211 3.149949679 3.586187397 3.03777362 3.680724068 3.247805071 2.962390429 2.798897886 2.896704095 2.812970305 3.017230399 2.281573352 3.830340354 4.795300265 3.211171859 3.367697927 3.240429243 3.375200094 3.069798539 3.45966008 3.990441043 3.292742169 3.289223406 2.97532276 3.343237495 3.649369352 3.896810355 3.207736371 2.96651472 3.745247781 3.515080911 3.860523814 2.904009796 3.361864359 2.679836581 3.669939042 3.084208203 3.806059495 3.051844406 3.173050363 4.34825545 3.782174669 3.816117169 3.706204179 3.317454071 3.48804759 4.071984506 3.914099154 3.228352023 3.082374648 3.583428837 2.972494594 2.977570413 3.373843758 2.65884411 2.642915432 2.968052171 3.098471795 3.488654437 3.366406914 3.028337194 3.450234195 3.32622146 3.676223785 2.676734525 3.603087982 2.781805423 3.689244535 2.993538727 3.318814732 2.524272246 3.086451324 3.143090453 3.468954565 3.144613838 3.541498255 3.236492541 3.544318337 3.695162215 2.827935456 3.497296272 3.26365668 3.069157131 2.997342794 3.343828007 2.513057559 2.799919166 3.609052438 3.077974421 3.781250955 2.181609209 2.827399921 3.340816417 3.441335777 2.738160976 3.057535526 3.006890515 2.578034516 2.039936874 2.861829966 3.011391459 2.86399465 2.403467635 2.731088539 2.901111334 3.101869316 2.136702196 3.257264073 3.415943629 2.549412637 2.122796252 3.276455165 2.960351891 2.958694662 2.613694838 2.595162799 2.795365448 3.02533278 3.729699844 2.96161047 2.191190848 2.651806206 3.711289023 2.753024906 3.09495978 2.831857272 2.066376548 2.38278075 3.071196497 3.110202387 2.166284511 3.298187365 3.074317959 2.855101246 3.037596906 3.387421121 4.392028112 3.753607147 3.129772287 2.845166139 3.312677016 3.314372282 3.444041301 2.681042419 3.160663079 3.598560062 4.10357625 4.080958424 4.287842055 2.736831138 3.945138849 3.778755978 3.997535297 3.385106889 3.778349132 2.564564995 3.674705129 3.394069854 3.161866156 2.449805439 3.160192834 3.912848194 2.988388539 3.084705172 3.622093431 3.228648586 3.305475814 2.719035521 2.893383431 3.039108668 3.429988741 3.297975898 4.239850091 2.659284123 2.645304465 3.429207851 3.676940756 1.590959504 2.78256901 3.126963443 3.159100995 3.644508581 2.934606974 1.807979998 3.362809381 4.09913523 4.295925941 3.579640213 3.956056414 3.107951662 3.452806533 2.27372176 2.62356006 2.982295417 2.90569709 3.093709296 3.440079641 2.796288895 3.859109246 3.564790514 3.545990196 3.542374194 4.268667275 3.401234096 3.167094297 2.552714538 2.438267087 3.956522216 3.078596975 2.595553548 2.999604998 3.221857118 2.954182947 2.885278645 2.471551251 2.46111561 3.123534414 3.379729662 2.806861591 3.254941894 2.924974404 3.977531841 3.764750878 3.285704688 3.613825986 2.973744009 3.799098865 3.007565631 3.150821635 3.717236325 3.573634448 5.489154896 3.082240739 2.155202456 2.896607268 3.539850067 4.176159085 2.909536086 2.500539854 3.177416653 3.337026811 3.129005128 3.071801545 4.116161304 3.194392466 2.937913346 3.775653077 2.826400099 3.047574236 2.980080644 3.802929957 2.663179683 3.09154181 2.561514297 2.717814741 3.485433538 3.315566911 2.907410361 3.903095603 2.242365482 2.764873227 3.263749759 3.209538372 3.607080901 2.503821272 4.869629329 3.274800563 3.709431779 3.504778567 2.470698081 3.104624087 3.638524814 2.87315352 3.304867908 3.156554692 3.444892542 4.649823805 3.640560693 3.040884936 3.699648901 3.348719648 3.580288972 3.197859466 3.388498581 2.82294598 4.117863725 2.8878029 3.236511197 2.785647003 3.58776514 4.182063631 3.124343432 3.432340514 2.531615574 3.629460905 2.788445839 4.437900548 3.060464707 3.535266413 3.262784698 2.326210339 3.302723221 2.879259894 2.894956412 2.916375994 2.517931819 3.394315806 3.115275839 3.328323443 3.470440243 3.525653379 3.303296357 3.389510443 2.62018395 3.643675711 2.895843978 3.249388094 3.371767308 3.046962111 2.296214353 3.269880409 3.914045984 3.41942964 3.641431654 3.358607874 3.858581463 3.082581766 3.51427417 3.618579756 2.62449966 3.095820153 3.88594204 2.124012022 2.675788804 2.982341805 3.405768476 3.353813786 2.759313619 3.265999241 3.89962662 3.431967741 3.161702025 3.064079113 3.164562711 3.153105259 3.317804421 3.283736485 3.118012719 3.207967703 2.636485917 4.053232022 2.535909156 3.279996887 3.63833193 3.658413465 3.148436871 3.728615823 3.531951127 3.342871803 3.086716017 3.797809325 2.907634458 4.063632263 3.125475898 4.320308444 3.372209548 3.336889484 3.136712823 1.916624969 2.795613836 3.002648436 3.076602336 3.5801354 3.591756939 3.476606135 3.197271214 3.429531346 3.02871386 3.439175632 4.603786071 3.197268149 2.953688285 3.700819771 3.246410323 2.62724211 2.933960025 2.855201231 4.611502939 3.708397495 3.07470444 3.514160661 3.606579495 3.539891379 3.743710104 3.717360572 2.810121299 3.372978053 3.387449839 3.373595844 3.360267819 2.906766898 3.698433111 3.520580242 2.77242257 3.61799835 3.128473847 4.473442388 2.889599885 4.085119449 3.871352739 3.031825505 4.589638302 3.002336197 3.567087408 3.601681311 3.934265684 3.303932802 3.433866116 3.697219411 2.888939812 3.94807047 3.506209829 3.633500001 3.041228877 3.363154848 3.202254804 3.414997786 3.818238904 2.805561415 3.068232216 2.362208452 2.742721218 2.344671657 2.799221741 2.968538805 3.283395064 3.078636918 4.555924137 3.378081804 1.625384675 2.807323362 2.91237452 4.09803766

ENSG00000167972.12 ABCA3 3.15900791 2.698310842 3.304273107 2.128712094 3.159153191 2.852747065 1.632328735 2.914358703 2.545687772 3.846470164 3.311033861 3.218229158 2.366958893 2.398729199 2.679040867 2.505099505 2.677232465 2.227606894 2.951448613 2.531913396 2.959962686 3.182624069 2.675163051 2.692676182 2.738684377 2.598962757 2.763410642 2.168921231 2.773717194 2.369787427 3.076625894 2.349102042 2.296689719 2.748026659 2.432252588 2.822372082 2.318308084 2.903950699 2.893091312 2.796273648 2.557179987 2.699926416 2.781947436 2.677710195 2.959552132 2.677468562 3.040603324 3.049438234 2.978553111 2.890588396 1.907523937 2.890010052 3.198653761 2.840517042 4.178477244 2.925954676 3.204981026 2.81280369 3.367938137 3.477032228 2.605816836 3.524831893 2.09707226 3.629903319 3.047013075 3.63239936 3.767518919 3.115230958 2.401745818 3.251495561 3.372219681 3.517865379 3.553781479 3.831359994 2.89081266 4.583500184 3.215531606 3.165696155 2.714804982 3.490679444 3.122471498 3.515516391 3.326063448 3.938653902 3.898355089 3.136550982 2.006698834 3.90130248 2.862318276 4.200157187 3.280292354 2.982531813 3.272070695 3.784338786 4.135619134 2.96697206 3.229613658 3.413349109 3.258784528 2.922983096 3.578786454 3.575302644 3.362593418 3.155799082 3.140889346 3.345405742 2.924232079 3.341387632 2.856183025 4.229193079 3.350666806 3.779680678 3.166721671 3.10956607 3.452818674 2.659277178 2.81533637 3.62214801 4.629780471 2.765646779 3.571181459 3.45555756 2.937562265 2.828709585 3.328122696 3.529899045 3.511903642 3.571772425 3.675921046 3.600537897 3.900713568 3.394560714 3.157951337 3.056754577 3.312651251 3.179439357 4.127863786 3.352884026 3.547012312 2.760412099 3.514800731 2.850684256 3.941892709 3.340825355 3.829760047 4.417474091 3.473931518 3.962786434 3.42031714 3.952076759 3.243184179 3.831194034 3.108691822 2.800947155 3.13440133 4.286363032 3.320051542 3.041328525 3.518016997 2.436946629 2.890826326 2.950375961 3.291151001 3.01338228 2.609278212 3.351259069 3.273679488 2.860081263 3.674717251 3.41516861 3.432107876 3.341663353 2.994880337 3.206129422 3.156502942 3.053557623 2.876255875 2.940170415 3.221358717 3.006266014 3.1154803 3.507920637 3.233224065 3.535689001 2.764874081 3.999356135 3.434910355 2.720143899 4.229928335 2.800769591 1.938743703 3.347061488 3.482304405 3.792168388 3.121602307 3.055271357 2.743928418 2.174670904 3.470202849 2.330024039 3.176054339 3.514513139 3.077941972 2.580382292 2.79037497 3.282552988 3.254171445 2.57941363 2.520814632 3.163261318 3.080255795 2.683597159 2.947949228 3.729803799 2.145252037 2.006593727 3.85449573 2.826305613 3.137975396 2.808699568 3.524093104 2.787752559 3.628653198 3.122277796 3.080927297 2.253888641 2.709410294 3.503971761 3.248208216 4.00468628 3.517900811 1.77906848 2.638222936 3.624531462 3.479624223 3.808300017 3.249037315 3.259088754 2.588205912 2.891513221 3.788855821 3.096634739 3.000213374 3.881460564 2.868121669 3.431874251 2.90751523 3.71679822 3.737871576 2.043301275 3.732326708 3.468750711 3.652676316 3.321310961 3.766352604 3.360331398 3.790017683 3.340000142 3.979750895 3.845196794 3.226612059 2.784692055 3.171122628 3.482467037 2.427452035 3.438111553 3.935756413 2.629965275 3.19599083 3.760448892 3.737295862 3.494132735 3.198130289 2.862745452 3.27455705 3.605722175 3.823883476 3.519006429 2.456390486 3.246292933 3.395791364 3.480173981 1.33947918 3.558851023 3.17126096 3.848546531 3.282663192 3.865237768 2.371755243 2.573354523 3.468198646 2.84490733 3.625122243 4.2029116 3.320754248 3.727771114 2.423234598 3.321391558 3.353483481 3.390037748 3.231781205 2.994763337 2.489683122 3.361652148 4.21147301 2.827774868 3.262375603 3.068995324 2.907972278 3.051051351 2.368011822 2.599134879 3.349397648 3.414923094 3.000558716 2.633086212 2.69267954 3.322486244 3.213517732 2.819118788 2.675816439 2.553269151 3.264505196 2.98556445 2.437390098 2.847415596 3.283844637 3.844369839 2.911858688 3.264986527 3.180743602 3.409785368 2.945947635 2.902395609 3.562939609 3.18450596 4.332088497 2.676781579 4.110088955 3.321533952 3.268568235 3.496801058 3.300574248 2.487159845 3.582607971 2.559396243 3.262491074 2.593395644 3.247627195 3.101978365 3.485447027 3.275612841 2.438682697 2.999202976 3.081902606 2.664031517 3.260919814 3.602903881 3.133914626 3.699899921 3.123427901 3.898030522 3.403653363 3.460235924 3.258350593 3.426493371 3.844179906 3.127905402 3.78776813 3.31262448 4.827444308 4.155675636 3.342878284 3.011110745 3.745988282 3.300024545 3.569284684 3.907537243 3.127419433 2.636999954 3.01669457 3.773481577 3.266652922 2.838094749 3.569643051 3.775937956 3.003738341 3.02173621 3.735146881 3.113367495 3.099851407 4.412180096 4.194348058 4.168072652 3.192830078 3.719450254 4.234283162 3.815877389 2.643174489 3.184854318 2.671399012 3.618353228 2.671417541 3.222480403 2.831368209 2.276370686 2.70962219 3.853558777 2.69965348 3.901560203 3.714364061 3.51102765 3.982983634 3.459883823 3.101724657 2.792661178 3.264308064 4.158327299 3.195805308 2.879089377 2.865712296 2.807231365 4.112998399 2.827494033 3.22755302 3.254212017 3.330129132 2.464724101 4.425162487 3.621035401 3.793398602 3.708301642 3.015693496 3.455367442 3.124655781 3.157920348 3.865055954 2.003574131 2.800539339 3.617233107 3.322243712 2.953832806 2.006532706 3.083890023 3.566691184 3.79088155 4.055076823 3.087380888 2.773638626 3.141905432 2.743243518 2.722976601 2.95893302 2.911795502 3.495398241 3.098991822 3.591861597 3.348585131 4.034132282 3.779275326 2.613668504 3.172552277 3.469228832 4.620149336 3.279926527 3.342207355 3.561447275 3.738174539 3.729990798 3.500000404 3.393336988 3.199060584 3.219599881 2.009811744 3.080703175 3.12647022 3.627755597 3.629247685 2.816656441 2.724467118 3.124771841 3.818872076 3.173189721 3.592102421 3.24408942 4.053924204 3.150110466 3.658510723 3.276411722 2.864242617 2.390082183 3.222032748 3.912562211 3.44523558 3.283901015 2.952242276 4.306882803 3.101569177 3.175139988 3.72164676 4.457172548 3.079313722 3.994679989 3.364495928 3.147726728 2.923887636 4.01817319 3.319346915 3.08832944 2.672400886 2.64064343 3.369455263 5.557284896 4.115132688 3.691618274 3.343041279 4.457162943 4.443612592 3.858623522 2.844949036 4.186107575 3.139973778 2.959149812 3.216306358 3.460389507 3.094390799 2.706113237 4.158435004 3.631917767 4.251333916 3.35197551 3.299011286 3.660267783 2.820629103 3.337784712 2.278703285 3.354219815 2.911832961 3.019135576 3.293288748 3.619598413 3.140359129 3.678931286 3.080947993 1.911740831 4.575290258 3.774466611 4.140770306

ENSG00000198691.10 ABCA4 0.121578163 0.09582404 0.076858388 0.069892905 0.033982469 0.075277508 0.066000854 0.087512423 0.182279824 0.066826437 0.065026733 0.028313678 0.038231913 0.104747053 0.065413763 0.078343988 0.099347997 0.089844531 0.100404918 0.020623805 0.091403418 0.065565716 0.110860225 0.068654706 0.170786711 0.064645543 0.135895793 0.199396165 0.072926266 0.02903922 0.048292593 0.072437731 0.044725534 0.089602088 0.248432715 0.078256203 0.112724143 0.087880005 0.690559887 1.532022142 1.078352771 1.52546806 0.11745279 0.840757436 1.119587989 0.08632176 0.033317878 0.556270286 0.045508041 0.044734089 0.22107614 0.074896238 0.05269115 0.024700738 0.082294854 0.087197152 0.042837946 0.136322482 0.048743465 0.039485167 0.06899039 0.145938211 0.735522425 0.074142015 0.054121513 0.057579866 0.034461663 0.27301126 0.121837679 0.14393989 0.065242451 0.133450271 0.025197331 0.046629003 0.025944957 0.024829722 0.04024516 0.088279405 0.177073693 0.066010229 0.130272808 0.077969379 0.195234182 0.074148636 0.015685281 0.053212664 0.076337043 0.085208649 0.012606989 0.04084319 0.036880671 0.064128643 0.084306909 0.090867525 0.156164295 0.051567366 0.151104095 0.059484329 0.066038012 0.078151999 0.085248963 0.070900944 0.086451168 0.095837087 0.027692571 0.008618919 0.184446354 0.50157783 0.078532754 0.031779679 0.051078916 0.056364818 0.049398869 0.082959304 0.045860047 0.087740435 0.014321817 0.0457464 0.030586365 0.173527691 0.065641352 0.084447038 0.114066202 0.103596105 0.074275718 0.071637826 0.183166842 0.045841743 0.113727054 0.02362048 0.020791807 0.022588468 0.024106511 0.035377932 0.080826328 0.10731255 0.002864063 0.070551928 0.077337614 0.055701943 0.190169308 0.047546353 0.007788011 0.027450965 0.026724698 0.041088652 0.111082719 0.053005587 0.07834507 0.028764356 0.062354098 0.075477257 0.087253965 0.052894442 0.093664156 0.061505784 0.050825188 0.248725736 0.035962103 0.041846619 0.078833024 0.037237991 0.088111632 0.093271514 0.111611088 0.067520021 0.124564476 0.182791258 0.070433235 0.107459623 0.065665062 0.028668355 0.110977723 0.023646521 0.126066532 0.024475559 0.034628072 0.053908274 0.115746112 0.068132914 0.049690946 0.0795567 0.087662114 0.033381145 0.157405852 0.027789304 0.080953978 0.097541959 0.03322979 0.069322623 0.009915426 0.044412589 0.073350302 0.065935165 0.101232474 0.035857799 0.224869376 0.083347573 0.160138999 0.082851309 0.046970029 0.072601692 0.139607745 0.025672384 0.102279245 0.155611198 0.029310939 0.053320621 0.092604418 0.066867104 0.052537152 0.108859407 0.084751824 0.081516224 0.035175556 0.141898424 0.161862383 0.195157198 0.091093027 0.031194146 0.008654249 0.121629835 0.010096336 0.063952334 0.074109517 0.021368464 0.096611181 0.093665722 0.023867081 0.024967716 0.081415079 0.011517016 0.05325119 0.047416039 0.042460624 0.015641107 0.024525134 0.101973985 0.048326017 0.048577712 0.071271872 0.024798233 0.045301307 0.091996463 0.066244028 0.046200504 0.023707809 0.034117475 0.054655791 0.06332104 0.140899875 0.160234732 0.064796608 0.066533738 0.080676555 0.109767936 0.223429141 0.058674435 0.196250375 0.277105484 0.034550212 0.05840826 0.079406089 0.028417258 0.41147975 0.055531915 0.077025694 0.116957753 0.036181196 0.056055426 0.029604379 0.048313169 0.016764316 0.172314691 0.108053846 0.064887233 0.043209327 0.086272572 0.015228541 0.048079389 0.015933357 0.025064931 0.121035734 0.040163557 0.095582059 0.058751538 0.053483106 0.018275962 0.119079549 0.089671347 0.135310446 0.060644564 0.070869078 0.042313906 0.03030351 0.051226473 0.053096319 0.073171538 0.079579233 0.084637448 0.243182218 0.035896813 0.091183294 0.094222941 0.032067583 0.13920073 0.111759558 0.290721633 0.372723767 0.085342848 0.025996476 0.100297078 0.130170494 0.193282535 0.00346044 0.081758368 0.059839682 0.086252038 0.04123269 0.078659262 0.044428915 0.082147222 0.093915689 0.050036677 0.214318983 0.080740631 0.076492674 0.091771678 0.113834 0.066984452 0.059146161 0.054706694 0.018974506 0.120909859 0.128498069 0.003502172 0.051294443 0.132221366 0.007307884 0.125887981 0.116938782 0.080203205 0.200247644 0.025585567 0.454393081 0.151486006 0.073142996 0.092122298 0.154982785 0.041368774 0.01648531 0.075938255 0.079057337 0.04151927 0.061071491 0.008394061 0.135112775 0.13728476 0.042814123 0.026128198 0.143541101 0.123145888 0.114899248 0.128035436 0.011464934 0.071296222 0.021623405 0.145519264 0.099728324 0 0.036514528 0.064353219 0.097391865 0.067875473 0.055898114 0.080741838 0.108140809 0.056474156 0.169646609 0.177730074 0.049165624 0.101559925 0.108370236 0.085252833 0.111540893 0.198025116 0.083436549 0.099814071 0.23153482 0.041594753 0.084241174 0.030134316 0.039987511 0.03788239 0.326497346 0.041764892 0.03687713 0.047607328 0.076017484 0.110161473 0.183304227 0.173431517 0.153690764 0.059706328 0.01258054 0.027867984 0.142756727 0.042068888 0.044101484 0.026714563 0.017197542 0.166350257 0.086742915 0.054224304 0.110659287 0.053225829 0.272612977 0.079345029 0.074550423 0.073869384 0.02073023 0.07078457 0.028839429 0.097894867 0.092380014 0.07545185 0.124133775 0.264341735 0.211547827 0.048022906 0.255285399 0.070671781 0.147117416 0.046211403 0.070482326 0.046567238 0.070696521 0.02109028 0.134315694 0.089718279 0.102129262 0.062791519 0.072752332 0.07061378 0.221425791 0.038192313 0.046409028 0.193389162 0.225912818 0.179381381 0.090626826 0.05795303 0.226342368 0.017459861 0.050779898 0.177883455 0.451709912 0.011749711 0.378871266 0.182702446 0.111890029 0.048434564 0.160511014 0.035455638 0.052359723 0.031427455 0.053966294 0.067707368 0.036237379 0.027194293 0.029154812 0.023103632 0.081239324 0.05248745 0.039722849 0.056666006 0.074500666 0.07116017 0.145615446 0.126396402 0.093850813 0.130480664 0.24936844 0.216906189 0.014413104 0.084874118 0.106613064 0.169693106 0.141824199 0.047916533 0.112083932 0.062218518 0.055238914 0.080686158 0.055838058 0.062150254 0.036942603 0.197444832 0.443464898 0.346910979 0.065348434 0.04893403 0.043700111 0.003337359 0.395365376 0.091071339 0.741913196 0.04031719 0.04296213 0.06438596 0.141322483 0.08292926 0.058647887 0.038343575 0.021760937 0.229797255 1.067571972 0.00788973 0.078935445 0.048302887 0.169277478 0.204028001 0.097000663 0.059954669 0.028014345 0.065349849 0.084082924 0.032437394 0.225038964 0 0.022342611 0.185234409 0.017240047 0.049371724 0.04568649 0.0214054 0.105053134 0.029161232 0.032721876 0.138406202 0.105412907 0.216259243 0.078309259 0.054873346 0.252406244 0.033475954 0.022147535 0.206307965

ENSG00000154265.14 ABCA5 1.850208307 2.138447901 1.544327244 1.034242746 1.611378793 1.434008635 1.356656258 1.637501226 1.579130557 1.637309417 1.745171304 1.711570217 1.483834319 1.372350317 1.49188282 1.91318765 1.847716686 1.202191057 2.065600219 1.759481232 1.822441413 1.910721688 1.745592008 1.595393428 2.009860245 1.559017808 1.473960268 1.181799428 1.156575627 1.177720317 1.338469894 1.625771438 2.200563277 1.060786097 1.693183236 1.324020188 1.157121114 1.299507787 1.97499165 1.78152989 2.086486038 2.223934819 1.282863876 1.38766795 1.745044127 1.419701285 1.521625977 1.828460623 2.057178758 1.896924026 2.179118944 2.047833462 2.109481841 1.884760336 2.944256546 1.886170963 1.594967124 1.880469266 1.849192201 1.586505612 1.181840979 1.877330236 1.511584796 1.975284423 2.128069889 1.863692502 1.754536396 1.092191465 2.218907386 1.726634495 2.081353433 2.139795678 2.194455562 1.894877475 0.999320706 2.066750212 2.312822532 1.049152194 1.378454879 1.906416195 1.881984775 2.03379492 2.132430615 1.93709835 1.747345672 2.040343275 1.131923177 1.716361498 1.613331364 1.689013277 2.004124086 2.355370097 1.196557375 1.27552549 1.622906735 1.758874386 1.883561625 2.335375395 2.066206647 1.924032159 1.286428446 1.610670693 1.82218805 1.603809066 1.89855814 1.853208989 1.893974508 1.834612565 2.104823151 1.41654915 1.667993526 2.460286544 1.88984557 1.390616887 1.958238546 2.400391721 1.739434806 1.436586861 2.702628425 1.089658752 2.022144774 2.03313943 1.103133661 2.47365297 1.922947172 1.71570451 1.465451518 1.50784803 1.961299236 1.619104262 1.70525511 1.583999466 1.774734793 1.522164343 1.206960989 1.274582548 1.112210829 1.196067057 1.850225644 1.283184666 1.740624115 1.893287475 1.971853112 1.907081108 1.948048026 2.100815512 2.116355203 2.001127734 2.137329085 2.075765373 1.61518503 1.555225063 1.615205256 1.813488903 1.423886067 1.793097508 1.95023266 1.92756509 1.457437853 2.582955718 2.534979399 2.007502887 1.69744899 2.173499175 2.334759815 1.674603192 1.778191532 2.497739022 2.236618619 1.294161485 1.268013163 2.546996449 1.868783825 2.092570524 1.84172767 1.773027272 1.572915373 1.480417137 2.084683002 1.91624298 2.05569772 1.846001263 2.035710337 1.392460337 2.110659813 1.833756598 1.840803941 1.908170282 1.879470487 1.978017398 1.200452522 1.608440056 1.98849866 1.531699691 1.153555098 2.676054385 1.319435805 0.850513859 2.071349642 1.547169518 1.454919164 1.497572536 1.089860044 1.504870857 1.804078368 1.414485606 0.954331376 0.87182171 1.216807711 1.472001596 1.274579378 2.087798937 1.42529305 1.301989116 1.92655503 1.263191881 1.670745486 1.022854998 1.502981535 1.218549797 0.879711449 1.21847925 1.064942886 1.231617143 1.364305605 1.173471307 1.215362302 1.810002661 1.294687974 1.24296802 1.069604031 1.34261692 1.186535338 1.920138443 1.246853323 1.047300845 1.284392185 1.577623798 1.455274677 0.561537368 1.310968592 1.701784292 1.880718641 1.661977587 1.652773649 1.148576322 1.553989979 1.552484984 1.474128333 2.07334893 2.509826843 1.524466752 1.360597571 1.851319211 1.603675449 1.83923838 1.162731831 1.537086443 1.428432523 1.404268552 1.918712374 1.685053737 2.00714545 2.275751691 3.321011544 1.449643776 1.202514875 1.843186548 1.516307694 1.96047311 1.393497752 1.348011365 1.153995313 1.541982364 1.977201787 2.0089684 2.048511188 2.061948848 2.343632296 1.827479029 1.987911025 2.202230056 3.035631158 2.350737908 2.350977862 1.832852549 1.98358466 1.416703667 3.020223559 1.954123569 1.432503003 1.688469743 1.590499328 1.788891418 1.399385148 0.931412926 1.386809968 1.524970553 1.661237626 1.27035244 1.22411727 1.497267852 1.28766566 1.692621706 1.253432576 1.753738235 2.04183504 2.049585788 1.86825509 1.512868217 0.805673842 1.155401633 1.997262367 2.227466477 1.875283993 1.274233214 1.383388842 1.149124931 0.95702125 1.222512616 1.511877127 1.476681149 1.551862561 1.78429031 1.818734914 2.020921042 1.790438654 1.862544642 1.948020763 1.768372521 1.921550481 2.223828485 2.204515765 2.24429938 1.642452011 1.469539223 1.895386532 1.373402568 2.180246166 1.48228669 1.479853317 1.815734654 1.671937224 1.697396771 1.478331895 2.128682874 2.328039509 2.011503373 1.578701744 1.833259985 3.005838714 2.09224968 1.532439083 1.620979027 1.435023318 2.869537339 1.856663416 1.516449415 1.177120736 1.512697103 2.341835941 1.946945518 1.537749341 2.111082758 0.960874194 1.249109971 1.714762996 1.838550146 1.532758102 2.021434245 2.061874889 1.068849097 1.674782272 2.061797011 0.95116547 0.926860433 1.831127395 1.025525551 1.66475136 1.504242472 1.191362009 1.392534347 1.573088832 2.380627574 1.951660676 1.71918334 1.241971758 1.683816125 1.48071891 1.402791973 1.511799558 1.841995393 2.070811484 1.782814063 2.057756979 1.966015675 1.790836199 2.508654518 1.955154567 1.781255296 1.87991478 1.115478595 1.63374981 2.116487872 1.116485662 1.38589693 1.307512835 1.237912045 2.121305813 1.842380446 1.417090937 1.35530523 1.318151182 1.978828612 2.090592761 1.584172908 2.310744749 1.718312658 1.505559873 1.88624135 2.109874019 1.802930747 1.989692176 2.533999994 1.69510054 2.422054046 1.783419369 1.989417413 1.560063129 2.003996132 1.570943606 1.703206468 1.924915229 1.477826534 1.795251685 1.486097886 1.222322081 2.101892342 1.856688055 1.997337678 1.730410197 0.998657279 1.179384946 1.482255015 1.48136027 1.513962652 1.910583155 1.520750199 1.56540303 1.390608536 1.894161337 2.343068645 1.835337556 1.880445025 1.625670318 1.556823307 1.536120773 1.899908568 1.572638471 2.245875029 2.488158703 2.156879884 1.919903865 1.822943226 1.832520638 1.893112516 2.132651807 1.237026075 2.193343013 1.91806623 1.32339051 2.246991704 1.524766525 2.380927447 1.680103599 1.468301268 1.631608065 1.83057179 2.422603673 1.679060454 2.025795635 1.923960313 1.838024845 1.292930855 1.359372453 1.300457398 2.740099624 1.916678539 1.666278267 1.507536843 2.042674853 1.263543463 1.832480424 1.546922001 1.640299129 2.187924465 1.492149231 1.716657385 1.869161773 1.958378443 1.789839413 1.572583346 1.838900217 2.076879973 2.18348635 1.745254053 2.159368355 1.829497437 1.709741897 1.803998393 2.029849485 2.475338499 1.665464456 1.612510408 1.641509165 2.166633916 1.138691711 1.91331918 1.908009614 1.828868954 2.154242669 2.578556513 1.995858637 1.183754893 1.68469053 2.088348795 1.741382823 1.927077782 1.90265413 1.106929082 1.032112821 1.02764197 1.833649679 1.685537583 0.848243027 1.093749906 1.707822768 1.729531448 1.76640967 1.966707385 2.078274483 1.487391922 2.488850622 1.665758133 1.01991141 2.053324252 0.695545176 1.21002143

ENSG00000154262.11 ABCA6 0.13798388 0.231115374 0.077197207 0.067563922 0.250492918 0.379267808 0.174200503 0.210450092 0.44415068 0.15508482 0.319435747 0.151447874 0.353790684 0.24625226 0.214276048 0.522664642 0.33581413 0.425297331 0.341786975 0.329163148 0.418798751 0.234824001 0.389559351 0.357731413 0.681188619 0.204726435 0.328263641 0.073165323 0.022367566 0.12217575 0.098671992 0.115205734 0.070220664 0.076792317 0.064470015 0.085059466 0.098482181 0.080837422 0.06905601 0.526628228 0.209198279 0.224772776 0.12267524 0.227682806 0.194349504 0.19441479 0.062126984 0.192248663 0.186059325 0.134582299 0.128545167 0.283930578 0.104941983 0.189171845 0.118663023 0.135157285 0.109156903 0.074365341 0.099892702 0.097181381 0.209301629 0.166006168 0.406973138 0.036035407 0.591985882 0.273154329 0.086844033 0.03529414 0.176972123 0.09918506 0.378526721 0.115256211 0.15852746 0.218741758 0.416595036 0.107193059 0.133562278 0.096260719 0.099177041 0.27695336 0.290628724 0.178123391 0.179639266 0.324741165 0.129576027 0.070502029 0.07445265 0.160935224 0.08892953 0.063736512 0.151917114 0.107378877 0.125735227 0.125993782 0.053803532 0.346561451 0.292208174 0.13632609 0.474585572 0.320908823 0.131706208 0.102657704 0.334155721 0.220075985 0.361771624 0.101548942 0.115523735 0.100226649 0.149530537 0.28056963 0.114367225 0.25709633 0.049448161 0.469278075 0.116672446 0.368170964 0.204457824 0.336725086 0.137804397 0.060829245 0.323991503 0.465235989 0.200707434 0.305258872 0.45145467 0.299437344 0.13993055 0.243852197 0.397922999 0.155234244 0.154586876 0.198658462 0.130562773 0.078268284 0.081182152 0.098230701 0.094840169 0.107626589 0.146452264 0.080020459 0.291362425 0.246602911 0.150289894 0.371278771 0.254437867 0.127085141 0.243140866 0.152761981 0.473111403 0.261111569 0.422477239 0.127082195 0.288426182 0.472378528 0.039261365 0.364607403 0.099736018 0.522231315 0.117482705 0.20735658 0.095538827 0.14364914 0.118718647 0.117896198 0.128595322 0.007052208 0.141146619 0.503354696 0.130559125 0.087790381 0.026833795 0.204968845 0.280149165 0.148662216 0.090781634 0.077578081 0.081173552 0.242020062 0.032498585 0.132364078 0.22504839 0.084682834 0.127739301 0.171412442 0.144292122 0.327007014 0.142823838 0.177558727 0.057291209 0.225742619 0.137397804 0.130094274 0.493712556 0.291998965 0.032774928 0.04352347 0.048326515 0.030458577 0.06541664 0.103987692 0.050490875 0.110200419 0.038685114 0.201096265 0.055615109 0.053413431 0.035594216 0.117671572 0.113386072 0.114338758 0.072564019 0.051057078 0.180462038 0.208104408 0.062813458 0.088210815 0.194891526 0.084837012 0.057523537 0.065498604 0.029871681 0.114046614 0.063184672 0.136324044 0.066976367 0.151456299 0.111399802 0.219127847 0.113212849 0.152230007 0.042789313 0.211545336 0.035707626 0.140583166 0.12649902 0.085515592 0.14611632 0.131593162 0.471466206 0.064233362 0.082291414 0.046358072 0.077870793 0.175991443 0.225813183 0.053415123 0.039624576 0.027440641 0.194345126 0.596308373 0.047847315 0.459699895 0.100655752 0.195181137 0.116104386 0.127891214 0.060211202 0.072104139 0.098114011 0.195140095 0.19700629 0.263160788 0.323547601 0.072967525 2.385681021 0.101669596 0.069618578 0.227233532 0.077048192 0.273036169 0.217435384 0.140476647 0.072420071 0.186782851 0.178871049 0.09144839 0.067860278 0.164739723 0.035059399 0.082589786 0.132957505 0.202987999 1.685765144 0.073619542 0.400105311 0.077935737 0.137578613 0.433399776 0.974934708 0.377057794 0.166738742 0.109411181 0.05371315 0.365731234 0.096113187 0.078418655 0.027395097 0.128551023 0.083261549 0.102704035 0.102232149 0.091805956 0.249814917 0.104916551 0.089263281 0.203147267 0.217318916 0.252489367 0.122354096 0.333136477 0.119099031 0.234273167 0.082050645 0.311671197 0.193265824 0.087304545 0.094227928 0.106360877 0.056187337 0.157222751 0.093644098 0.090799225 0.033964731 0.072878272 0.555883468 0.218417513 0.206820085 0.192491229 0.132881421 0.18758931 0.185269236 0.148005483 0.098401556 0.182256852 0.080033344 0.096286899 0.076131533 0.165491544 0.037715953 0.481620638 0.190552582 0.168388481 0.184392649 0.118915873 0.218427507 0.172272326 0.098845401 0.356400312 0.395984965 0.14071047 0.082170608 0.131630671 0.167972899 0.04463782 0.195078999 0.121579713 0.108671253 0.071247459 0.116508018 0.231434267 0.183647361 0.044825235 0.044038117 0.266675891 0.056685265 0.068336239 0.144071587 0.195993255 0.039352061 0.050274818 0.047432563 0.074330356 0.032476478 0.225582187 0.014936359 0.077166789 0.091985845 0.094859162 0.182401028 0.136326088 0.283860961 0.156054413 0.222345692 0.270682847 0.086935755 0.134674858 0.106045319 0.177838725 0.14607545 0.117375477 0.223773379 0.041744333 0.046247381 0.292920399 0.149612249 0.065163296 0.122558215 0.039200612 0.193252481 0.171796401 0.10415462 0.022417338 0.124964945 0.140832451 0.016986016 0.118470642 0.01631573 0.042256586 0.060980112 0.06118888 0.046855353 0.058081582 0.041781224 0.055571748 0.262124783 0.345841724 0.308898146 0.048800044 0.283268236 0.157381708 0.136522469 0.147138286 0.089983478 0.291359076 0.196138933 0.198119111 0.170438406 0.154318367 0.078300461 0.301905877 0.157840258 0.279417038 0.284946753 0.170897285 0.088348448 0.098071967 0.079824759 0.140662308 0.208520642 0.150564425 0.261100241 0.498238746 0.236440373 0.081534418 0.735215468 0.135486749 0.097425283 0.193572858 0.466593185 0.19370015 0.237376136 0.092898398 0.050561185 0.109735606 0.208782688 0.133464085 0.048153407 0.033787121 0.165618307 0.037144086 0.1265413 0.172742924 0.101311421 0.140825478 0.365634181 0.056423183 0.280456007 0.098081633 0.123897317 0.108917495 0.179242118 0.179954616 0.144290842 0.149457612 0.146365964 0.104343479 0.032133456 0.08482271 0.283480671 0.167739048 0.142577878 0.073094349 0.203399646 0.199697124 0.015031987 0.114298649 0.081717948 0.052033235 0.063363727 0.156568364 0.093404937 0.158788804 0.052772477 0.211611154 0.12880283 0.08703412 0.084236234 0.422223963 0.197257249 0.057179918 0.043879781 0.301773551 0.123156569 0.099140998 0.069099843 0.30773986 0.101125895 0.146336853 0.211209082 0.274810986 0.235696414 0.059468743 0.117637929 0.097936496 0.246983353 0.080510543 0.11869702 0.166527033 0.239410375 0.571677101 0.057307772 0.193369732 0.214250961 0.129964906 0.12552858 0.231330687 0.267031741 0.245498978 0.310999766 0.24999043 0.50452083 0.406954956 0.162607812 0.596611634 0.176989799 0.145505727 0.146474738 0.246560367 0.281400054 0.085791906 0.162770555 0.090188438 0.19682339 0.290359914 0.101265754 0.131236971 0.123869742 0.197975436

ENSG00000064687.11 ABCA7 2.233619259 1.460184658 1.387443165 0.924533653 1.237010412 0.832961361 0.717311412 1.40174878 1.503592296 1.889603274 1.830705291 1.223489478 1.074173804 1.031550226 1.326677068 1.302807652 1.19015116 0.781442251 1.135812042 1.252784886 1.411151002 1.567192224 1.244181558 1.442544747 1.581287999 1.098465827 1.74497922 1.260313686 1.167416239 1.207477877 1.832387732 1.545807331 0.717203145 1.08527098 1.854574958 1.310062985 1.360902305 0.60717176 1.69930748 1.633544712 1.26708928 1.45508495 1.647912969 1.546516207 1.861841446 1.513808361 1.664355076 1.326521453 1.414010846 1.759442548 1.591523364 2.112510828 1.717718776 1.425370346 1.758856331 1.181278533 0.458567939 1.485184845 0.92973007 0.911975415 1.263219155 1.558769803 1.258996233 1.086028747 1.500968489 0.924471814 1.265900632 1.114336024 1.606939669 1.547705039 1.245546987 1.202040127 0.922841619 1.156996347 1.372346164 1.190179584 0.630046041 1.601238012 1.270106044 1.137499176 1.236931874 0.693202429 1.005275768 1.135274357 1.134012589 0.927723596 0.793713419 1.082175366 0.494289004 1.000839827 1.812529979 0.93270467 1.00197098 1.724768126 0.98343196 1.386253578 1.513266044 1.03163896 1.416179234 0.869872738 1.160664785 1.648343206 1.759441706 1.961575831 0.878290205 1.622511205 1.512549849 1.861695737 1.28181536 1.548772873 1.220307855 0.405485548 1.03499571 1.173963235 1.273699666 1.079551513 0.849477121 1.289091525 1.588420014 1.475892373 1.238585565 1.245874766 0.466938141 0.999383332 1.185361785 1.55804717 1.488727727 1.437662867 1.038604761 1.276076084 1.163130686 1.542764245 1.180615548 1.333878959 1.37238481 1.183065358 1.811745155 1.853791635 0.724574443 0.865772337 1.074863433 1.426546671 1.430806495 1.350946097 1.126873441 1.271466386 1.682558997 1.020708561 1.365479329 1.448420655 0.820997625 1.400940593 1.742035173 1.026940857 1.464824584 1.387727552 1.089885837 1.551051626 1.062494687 1.657406986 1.363813146 1.157156965 1.967351744 1.671933705 1.60722019 1.399546372 1.675818023 2.488479363 1.80776946 1.430440002 1.472040006 1.988414095 1.106723409 1.309986035 0.661536677 1.125067588 1.568377837 1.679797305 1.401835315 1.430339158 1.671468459 1.990601583 2.145111847 1.805805027 1.996642776 1.695340151 2.015932193 2.318962539 0.888977917 1.972690794 1.090087312 1.572983347 1.841429373 1.657304692 0.404741188 0.616174061 2.37663589 1.118641383 2.277049047 0.869406532 0.932226206 1.455923482 1.076049263 1.194742191 0.896353828 1.164738616 1.378140793 1.478665389 1.222114848 1.69653911 1.772884522 1.426210767 1.302645047 1.073815869 0.440953738 0.656122621 1.294839723 0.911196984 1.641375507 1.564868226 1.042331172 1.268363611 1.32127788 0.642828176 1.648694805 0.94436593 2.415031829 1.149733737 1.339966726 1.294112288 1.142056371 1.107567624 1.17306267 1.383719248 1.315939865 1.19711121 0.881086833 1.794604154 1.049763869 1.060697116 1.975427358 1.389978958 1.815219025 1.441857966 1.372340534 0.842501894 1.159786318 2.167700029 1.399366661 2.679646289 1.868690313 1.039772293 1.710501203 1.441911495 1.093491379 1.024568569 2.215380615 0.856116593 1.103367932 0.866124729 1.089794775 1.048807417 1.374020442 1.199508136 1.271890903 1.533483726 1.103479436 1.657361827 1.528579098 1.581113134 1.023778615 1.088006152 1.901378432 1.796816002 1.389009238 1.361750713 2.004253921 1.420936942 0.613956684 1.131352969 1.705717952 1.137698678 0.683993117 1.312158779 1.74111093 1.293180512 0.994534365 1.327790955 1.01959502 2.223081542 1.399680284 2.024434239 1.579574645 0.999898237 1.77475371 2.397235453 1.482353244 1.625805815 1.488773289 1.998182914 2.293010669 1.371741997 1.028403087 0.897718131 0.998543284 1.195792851 1.452511357 1.836715782 1.818609452 0.876747726 0.907849684 1.025542176 0.567574303 0.750200103 1.02718111 1.38592663 2.138067565 2.051915 2.295338355 1.970295791 1.451560942 2.399112535 2.223993945 1.443781272 2.348111428 2.049528769 0.818538025 1.855694286 1.635940237 1.417448859 1.640905793 0.957968212 1.644097934 1.897693077 1.522383518 1.800041966 1.653659019 1.505739765 1.225865784 1.378359582 1.038823515 1.708342569 0.584115756 0.845362089 1.346405604 1.681756357 1.252356211 1.353820771 1.535532828 1.416344231 0.840565665 1.458457251 1.24528276 1.587504776 1.672381523 2.26149877 1.370053257 1.693907927 1.212773023 1.340761057 1.165960291 1.535868383 2.333929254 0.724791741 1.010082455 1.15322657 1.138412934 1.048333804 2.837542786 1.000828725 1.003585087 2.788023092 1.972393734 2.022600659 1.194335535 1.26335642 0.969992547 1.866357054 1.189362785 1.829148951 1.617625718 1.484778329 1.744446182 0.91078816 1.785243886 1.504304703 0.60027404 1.681463499 1.305911496 1.245480938 1.490356937 0.892585896 1.230434033 1.370466828 2.539178895 2.015593735 2.11403855 1.399831708 1.398557412 1.416692186 1.623615135 1.509315711 1.798706696 1.803265673 0.848976962 1.140384517 1.03038802 1.885680159 1.235060619 1.005425601 2.577474229 0.99585827 2.0994657 1.944938524 1.908594118 0.920895606 1.981207126 1.939059964 1.568563582 1.426668904 1.38147589 1.30353126 1.102893278 1.799122696 1.586308825 1.653295083 0.893237378 1.746717422 2.147212412 1.56969554 2.268972223 1.133960318 1.935295235 1.206684361 1.107480738 0.747123536 2.047843992 0.630859705 1.551551125 1.737586613 1.511360165 1.245672452 1.495213419 1.74849153 1.836487966 0.877376339 1.506120494 0.976897392 1.725672804 0.844100459 1.227803459 0.927962765 1.00099792 1.589630256 1.513585671 1.208776831 1.615102796 1.557634831 2.533309722 0.478172841 0.646588524 1.771567565 1.519077223 1.278148171 2.015431054 0.872677484 1.62109444 1.213318613 2.645321132 1.000671971 1.531954324 1.398395911 1.438764498 1.2889931 1.369766708 1.455622031 1.879849195 1.499292278 2.12623007 1.906061867 1.672318406 1.131915331 1.207160861 2.25665179 1.55519304 1.607752713 1.578365968 1.607782484 1.302489119 1.228327056 1.712191501 1.692328307 2.621914525 1.377535472 1.321260143 1.428053653 1.102949855 1.281443109 1.590451158 1.359317509 1.51480796 1.817168092 1.726606761 0.921728953 2.042231442 1.504865299 1.248178308 2.274669834 1.773430086 1.939661186 1.524684319 1.434493221 1.048349199 1.032092763 1.463501122 1.294847534 1.889783723 1.140113803 1.80893614 0.906760631 0.70503571 1.686336927 1.747373898 1.579876678 1.145951372 1.617138194 1.699929282 1.389635332 1.668164655 0.400527251 1.520293557 1.215861041 0.825208451 1.549256905 0.824723545 0.733757346 1.02145366 1.433576443 1.440083604 1.3640786 1.181239628 0.94123834 1.599339265 1.769152468 1.447623915 1.731229628 0.7537129 2.047701231

ENSG00000141338.12 ABCA8 0.942014009 2.004117075 0.96932674 0.649632622 1.684502047 2.99651389 0.675855376 1.393402323 1.771384327 1.348905498 1.82785079 0.885730227 1.942287261 1.227093842 1.313700955 2.317550283 2.039808994 2.154000091 1.837853152 1.536513722 1.524574624 1.755643455 1.800030796 1.458694329 2.799379777 0.891822356 1.590776426 0.743794624 0.291476199 1.286062659 0.731348613 1.039629163 0.267410782 0.528798654 0.627247924 1.182818893 0.700667892 0.702561777 0.267983023 0.82933675 0.735435424 0.720023901 1.035659891 1.049263123 0.568761178 1.075710698 0.672305677 0.470213222 1.358073374 1.086775739 0.712809331 1.234880949 0.845234144 0.902378463 0.563500738 0.981398643 0.421066144 0.393829062 0.833543378 0.752552696 0.792813728 1.041368701 1.014787353 0.559281178 2.133981926 1.697085147 1.158233836 0.781477297 1.45631384 1.011955409 2.035494661 1.288293487 1.122701062 1.272674356 1.387948171 0.580386927 0.777040502 0.339000099 0.283949244 1.51057692 1.738459491 1.317293633 1.429756573 1.375381623 1.504179065 0.961183502 0.679641023 1.434896638 0.482257947 0.295615842 1.468770499 0.851649895 0.947951108 1.623017327 0.581741896 1.419864182 1.876398251 1.253144855 2.555093786 1.412780764 0.783981023 1.133029709 1.746369756 2.128231736 0.95612418 1.440851194 1.032260468 0.782479789 1.288819097 1.961396617 1.123686339 1.395883057 0.733734188 1.113838787 1.541853043 1.18500673 0.960540522 1.382097521 0.689662614 0.665835158 1.775371916 1.898315446 1.310027589 2.086024906 1.991161241 1.088425573 1.430820316 1.132490854 2.13325978 1.432570185 1.015215336 0.797700533 0.527307989 0.679965848 0.788403702 0.400518818 0.901696452 0.795118726 1.230536169 0.345949179 1.82950588 1.888089818 1.132500794 1.851525177 1.818493261 0.972900801 1.487945198 0.995415829 2.429096778 1.151002557 1.431964963 0.453039172 1.462372966 2.411494411 0.708836934 2.254695495 0.605641338 2.334635726 0.899423673 0.940417214 0.410505486 1.214883999 1.086230948 0.993764814 1.698633169 0.105768273 1.162751346 2.423877557 1.138747933 0.768249962 0.066618747 1.402349464 1.400755526 0.915321806 0.503664712 0.685248059 0.933286064 1.167773684 0.670306593 1.323872077 2.056047679 0.794474102 0.803105931 1.716447318 0.651435682 1.505992387 1.502698412 1.59703989 0.765737229 1.781185746 1.325758895 0.940938157 2.408930785 1.936360396 0.038877713 0.213759121 0.789841823 0.141812336 0.496351743 0.506610696 0.429286509 1.110909033 0.54209761 0.709437828 0.245499993 0.576325886 0.40422705 1.096944306 0.949476823 0.75761666 0.942977575 0.64506438 1.83477407 1.048059716 0.23638157 0.96518185 1.582780447 0.595408364 0.550358519 0.74411492 0.313646613 1.126076932 0.736339936 0.502105879 0.566889176 1.105387886 1.220546565 1.880309506 1.055795996 0.840827307 0.292911116 0.550688599 0.492921427 1.308156207 1.206967331 0.911699623 0.364726808 1.469718488 1.726623207 0.892695009 0.515081893 0.373632385 0.453082816 0.679014725 1.275287055 0.394967693 0.44248996 0.404465117 1.754298922 1.75210498 0.33880939 1.648865465 0.799396278 1.992727344 0.768588351 0.782527278 0.836129127 0.887839797 0.835027938 1.214503626 1.553084557 1.388316751 1.503030326 0.568000548 2.764263775 0.960597 0.763689678 1.201999915 0.890499841 1.772221247 0.983429276 1.161906797 0.31082879 1.412514185 0.924655311 1.130745656 0.749817782 0.47363399 0.507770215 1.100753207 0.960623893 1.87459547 3.694494176 0.812636171 1.857472113 0.908112859 1.242224343 1.145923252 2.755152884 1.429014559 0.657531413 0.242053423 0.521258681 1.065875129 0.791460876 0.404268857 0.182845416 0.90921693 0.81337251 1.169789483 0.79075554 0.695381716 1.378765492 0.598904459 0.439114451 1.79105658 1.817345996 1.188512033 0.538128022 1.49990708 0.48096911 1.047703789 0.346729088 1.869923743 1.406052909 0.793140913 0.738809563 1.155623571 0.612309166 1.038049087 1.220324384 0.980909737 0.682680622 1.055025376 1.741573163 1.226123236 0.896534095 1.092084612 1.725650052 1.621561198 0.947097105 0.953776822 0.914239868 1.371541379 0.469494352 0.688526924 0.638421912 1.178100935 0.485489384 2.496289808 1.204067623 0.546845355 0.927104365 0.564795704 1.968457428 1.765614812 0.885930095 1.801154973 1.450274505 1.103724976 0.211133416 1.155768166 1.124863181 0.804818366 1.152562138 0.315850377 0.405451915 0.428573526 1.67317098 0.873597861 1.224688799 0.413821298 0.209204066 1.103743223 0.483040941 0.410737686 1.091459563 0.9840827 0.122290026 0.423981528 0.171473385 0.436238654 0.162718956 1.608333843 0.157390131 0.390887967 0.431183429 0.633622222 0.958512061 0.92613188 1.50361315 0.694448469 1.413153805 1.349053947 0.667171911 1.080940553 0.78242223 1.507662191 0.711726613 1.180539652 1.516024882 0.288897152 0.215917612 1.384488345 0.728396353 0.454542457 1.069561573 0.080113631 1.610735792 1.370870362 1.045503887 0.324983418 0.779647337 1.223384763 0.21111808 0.775139802 0.04072447 0.229792962 0.198084389 0.753762505 0.579387784 0.358067775 0.543503211 0.700772996 1.239463118 1.991355854 1.477985475 0.347313373 1.270886209 1.794514739 1.482683157 1.446763454 0.821872372 2.115969936 0.925689759 1.328160494 1.259281656 0.930010502 0.496100788 1.367431297 0.762052452 1.384096927 1.139988278 1.55662046 0.798268111 1.111392098 0.336973014 0.81555179 1.107400439 1.241876744 1.741775786 2.164580564 1.052494116 0.984627668 2.339645187 1.051706622 0.931789246 1.408781922 2.072310028 0.313032862 1.610852684 0.695886576 0.463515928 0.774428276 1.033824769 0.923814073 0.290530941 0.363247007 1.291993451 0.189360427 0.917730355 1.441815057 1.163457068 0.521619835 1.353134506 0.611747858 1.246251225 0.817096566 0.600663386 0.488019383 1.121024568 0.672177022 0.912026859 1.082085929 1.099206115 0.862493302 0.492817771 0.89763813 1.512359746 1.228755692 1.053364006 0.303804941 1.640364672 0.914143615 0.047803113 0.891555238 0.452066037 0.190896898 0.926504441 1.565660203 1.107982159 0.750359129 0.270221385 1.638906038 1.12921498 0.434245253 0.579969744 1.652140015 1.089550031 0.608492138 0.123759321 1.511374419 1.131374085 0.292406466 0.392886328 1.362904135 0.62218624 1.044423159 1.322678122 1.462341054 1.139436819 0.122046354 0.963677671 0.396140111 1.596585728 0.982671104 0.47988632 0.628540663 1.954084018 2.063752832 0.175523557 1.695356849 1.041445178 0.664945377 1.211533079 0.995015415 2.071205039 0.943902564 1.295963544 1.532799797 1.563887974 1.455292814 1.188505896 2.145468473 0.63268895 1.2620436 0.757306324 0.756040655 1.292256083 0.728866085 1.314821435 0.664676951 0.780920203 1.297151685 0.752092397 0.920882896 1.144201319 0.367218138

ENSG00000154258.15 ABCA9 0.187353157 0.361130734 0.113484052 0.100821571 0.242171451 0.668159805 0.048136032 0.091491252 0.201166265 0.217574861 0.326468778 0.070556803 0.360663118 0.192956085 0.158109232 0.359050436 0.346965083 0.850615699 0.301981549 0.151527434 0.215351187 0.253294894 0.176726885 0.19429715 0.508381228 0.128175236 0.76957827 0.182537671 0.050132674 0.214822043 0.126496977 0.129697248 0.147137029 0.177563548 0.138652163 0.2486265 0.205141231 0.13981362 0.123726522 0.271274256 0.327600359 0.383565148 0.212342034 0.400109932 0.284046851 0.119118436 0.090368611 0.232409804 0.178804684 0.162337175 0.119221038 0.221960378 0.140810401 0.368857205 0.253069745 0.209823787 0.298519845 0.201350816 0.149202365 0.163944115 0.215576848 0.556754863 0.500311172 0.102229533 0.552400691 0.493669567 0.211699711 0.093706906 0.300949773 0.141052135 0.644674442 0.16403417 0.231328878 0.32596702 0.458937253 0.204537583 0.246295443 0.138066359 0.092834185 0.205824789 0.428212751 0.413823277 0.275548714 0.283745984 0.409928318 0.105493212 0.119607611 0.332979855 0.10937349 0.117249831 0.194164226 0.127571318 0.096264908 0.227894565 0.100862452 0.292198672 0.377331513 0.229389571 0.516835407 0.239252552 0.126478676 0.171323101 0.293113164 0.192818718 0.296616722 0.17052064 0.160085856 0.163804092 0.182376934 0.463298347 0.167221387 0.343424075 0.095182059 0.452207107 0.213389066 0.405586032 0.087691206 0.547361104 0.357892388 0.102122131 0.299995017 0.587175939 0.273396241 0.288036698 0.39465528 0.230258454 0.140514516 0.108043901 0.279903705 0.386666731 0.195014915 0.157442895 0.178296394 0.137828334 0.075098508 0.088679958 0.111359002 0.135242335 0.194029068 0.062336594 0.420858951 0.3925312 0.289026634 0.393328447 0.256554807 0.175842345 0.261423624 0.238381324 0.51461463 0.289568552 0.226631491 0.280811605 0.212279129 0.434953333 0.052265736 0.371815674 0.111622159 0.327852914 0.131100566 0.322960257 0.101638731 0.374617794 0.071574115 0.171023201 0.356203454 0.010905185 0.581425689 0.527996674 0.230942069 0.160878294 0.028548568 0.219516214 0.328400139 0.272157259 0.116104277 0.249302343 0.180426065 0.331292053 0.133575284 0.225829664 0.482343768 0.153057954 0.186159972 0.18197257 0.173331055 0.502197887 0.232123597 0.189064801 0.173184549 0.480042632 0.073962517 0.260085907 0.449356327 0.761056027 0.019824892 0.148477801 0.067858737 0.010957669 0.087123008 0.074563501 0.054058047 0.223352741 0.052980584 0.448390723 0.078315813 0.086854768 0.077590309 0.096734903 0.150333814 0.179918876 0.135111792 0.111074016 0.19279686 0.321641176 0.141901732 0.17861858 0.416681246 0.085258297 0.102300784 0.04640768 0.157279291 0.126302619 0.060100596 0.231170254 0.07869234 0.174465153 0.076397037 0.484737898 0.141588648 0.114023513 0.065727866 0.133689958 0.116835042 0.143968733 0.320902917 0.178942639 0.292996772 0.325743411 0.517196397 0.250722797 0.120874359 0.105470206 0.066297635 0.064304399 0.357380974 0.033316015 0.067057982 0.069778746 0.447639952 0.474998093 0.23205551 0.515371531 0.106423677 0.75183136 0.098662799 0.320909073 0.155297644 0.108002632 0.144483779 0.26133399 0.403400639 0.248567066 0.250886287 0.251458011 1.686760818 0.328570441 0.108859307 0.275024243 0.166373895 0.337260379 0.147088401 0.232690982 0.116240197 0.266207529 0.24623681 0.180436638 0.147062886 0.298792854 0.159176155 0.253158832 0.175750887 0.220491655 1.615555813 0.154652739 0.538367059 0.136087478 0.255395797 0.418636901 1.487446204 0.367215183 0.184936866 0.132659885 0.085417941 0.140098887 0.19505385 0.116807107 0.053249319 0.142720083 0.134699339 0.128224988 0.052594207 0.066918526 0.403914931 0.090703369 0.125337416 0.297042043 0.291971471 0.263440788 0.229106946 0.354697994 0.102220343 0.101570787 0.132905844 0.308558192 0.429700433 0.081830287 0.066955844 0.138419042 0.139734797 0.16202303 0.182325637 0.101855671 0.038192227 0.198857353 0.354073219 0.185080369 0.127088354 0.261726483 0.475997384 0.358999956 0.166136436 0.306435225 0.152733418 0.326449178 0.111618184 0.10320144 0.090797491 0.221416502 0.173252764 0.675627862 0.153890973 0.123135248 0.341674638 0.200497036 0.705332001 0.253103426 0.246922678 0.74035573 0.208491733 0.280785723 0.58611571 0.238110445 0.429997404 0.086455026 0.194629897 0.419803592 0.058382667 0.105126526 0.463010716 0.273233283 0.357977207 0.102050686 0.03758852 0.126453989 0.047415137 0.017008689 0.242377662 0.152502456 0.075219701 0.133759366 0.248352925 0.079774744 0.087693258 0.329599953 0.076953445 0.089237194 0.12514221 0.053893663 0.14650636 0.180137732 0.322731545 0.193174469 0.211643874 0.447062067 0.181731762 0.272356633 0.17908295 0.229314965 0.241894437 0.225797409 0.317084754 0.120521438 0.151000084 0.53728563 0.137683763 0.097151559 0.23821328 0.074074427 0.35324974 0.203276819 0.236490315 0.059193225 0.145974984 0.292936005 0.037308986 0.16029663 0.052391729 0.040292712 0.115785309 0.216763205 0.026915727 0.100820971 0.031263597 0.106577053 0.280502017 0.513880726 0.289880858 0.115983176 0.291546436 0.499977354 0.246289499 0.426946411 0.369790147 0.477746138 0.221757195 0.296228253 0.263941203 0.228960618 0.194004772 0.369139822 0.233245925 0.425816084 0.200663243 0.204811737 0.185777174 0.308529029 0.105406573 0.375543972 0.229813067 0.24455339 0.294827972 1.374493738 0.091562836 0.22503464 0.62321513 0.292406735 0.169511697 0.405703795 0.567726696 0.142332495 0.480899717 0.138204134 0.187990574 0.245792114 0.270566714 0.151635419 0.07389477 0.141086167 0.238379041 0.064731943 0.276831999 0.414287592 0.242508003 0.375946491 0.290800743 0.205591834 0.450444024 0.116320796 0.408344816 0.257515765 0.389298071 0.108659261 0.230535241 0.262422945 0.223845457 0.165647938 0.126898539 0.117287948 0.420996572 0.189243128 0.23008803 0.081501646 0.409023648 0.176657127 0.048567944 0.300637492 0.144741454 0.188501371 0.17121954 0.352840151 0.135387744 0.144644509 0.133345377 0.456849139 0.329686168 0.07968169 0.102196383 0.550660483 0.25600488 0.164641823 0.06165282 0.168432905 0.320054605 0.232136972 0.05196285 0.162353874 0.283763742 0.420330993 0.214474484 0.536560361 0.266307359 0.398965854 0.261307755 0.156792427 0.27828549 0.340962508 0.145105235 0.320201641 0.74790551 1.080789494 0.160712236 0.342602313 0.301355549 0.118592382 0.236227299 0.661891978 0.315467306 0.615503653 0.508088972 1.020720128 0.557955277 0.38563331 0.296181916 0.335647171 0.274980556 0.155768555 0.218437512 0.242730268 0.441687092 0.154379229 0.17318052 0.10485544 0.228883638 0.59306965 0.104419003 0.246201247 0.301618133 0.446895038

ENSG00000085563.13 ABCB1 1.150139662 1.839187589 1.036433715 1.549995164 1.654674072 2.205963611 0.54626587 1.896699601 3.150223131 2.046134345 1.64043856 0.857466996 2.242116646 1.811091377 2.602516335 1.766111597 1.415641068 1.726706947 1.952672385 1.772825545 2.510583914 1.658443159 2.616257816 1.773872158 2.614121424 1.225150609 1.190869241 0.849637386 0.790293359 1.542369621 1.25624293 0.833395841 0.307081332 1.243477042 0.746570455 1.440030667 1.025635281 1.131106665 0.636551208 0.994971315 1.262552739 0.698857098 1.127464051 0.961641857 1.143334623 1.623212237 0.891618395 1.077162932 2.199953052 1.009777847 1.602850963 1.609664213 0.957547473 1.439528423 1.126990706 0.992172356 0.311773016 0.207535097 1.383083163 1.249917885 1.675720968 0.911642183 2.39636386 0.975099495 2.398825259 2.171598059 1.436873518 0.941493654 1.688790013 1.073646775 2.062446205 1.236186563 1.479610291 1.162760028 1.563920031 0.81345217 1.011203309 1.305390241 0.857709924 1.80040889 1.842465932 1.751129317 1.520601386 1.428308953 0.700753528 1.429209822 1.540195937 2.145189925 1.179205272 1.125671905 1.977068401 1.183467168 1.674083315 2.621182538 0.885778055 1.570130756 2.347527898 1.254647053 2.126800105 2.213714627 1.718507619 1.076190877 1.952701707 2.074267495 2.295068862 1.233512923 0.919868207 1.896082673 2.023314096 1.797863982 1.426280387 1.526291414 1.281757954 2.349533295 1.292849813 2.411292348 1.640846896 2.101942097 1.32461033 1.263157771 2.208812414 2.284269224 2.83777648 2.456664948 2.813292655 1.119249161 1.964132122 1.459284452 2.462842792 1.385019895 1.660857174 0.659499956 0.959676127 0.638462418 1.254838889 2.308058707 1.12545956 0.745418525 1.213484788 0.495767329 2.295463187 2.53736151 1.794746741 2.205163157 1.503575488 1.404679024 1.857707294 1.301156496 2.309628861 1.74886408 1.615413696 0.632287847 2.154135248 2.766591862 1.240696479 2.273720312 1.333111802 2.413904006 1.353000841 1.639194813 0.77504513 2.214003977 1.577117884 1.190913897 1.363121246 0.261896849 1.522282356 2.006116395 1.23398585 1.183130369 0.280348876 0.539616974 1.736818243 0.916104566 1.372294716 0.947654046 0.97142783 1.920117679 0.588045982 1.774010107 1.590186161 1.031562495 1.040851421 2.001976536 1.440924212 1.799204943 1.351634065 1.573405441 0.785812972 1.677555707 0.9597053 0.845357023 2.66283015 2.560458514 0.116372925 0.54454549 0.576219863 0.243686825 0.747049786 1.135349744 0.55985074 1.284274514 0.992701878 1.010353526 0.750617782 1.454647974 0.709206219 0.96677615 2.115236948 1.058566173 1.487841343 0.306473088 2.141714628 1.783808929 0.397954309 1.427831732 1.716461439 1.418489907 0.508473144 1.097450867 0.356655547 1.564092072 0.771277354 0.905377709 1.183482354 0.807636171 0.780233402 1.660252234 1.160723488 1.016714652 0.7648446 0.742961087 0.830316876 1.333627727 1.719465524 0.985706163 1.144606465 1.524687234 2.443432261 1.39683376 1.059352941 0.499021317 0.81782851 1.408978589 1.735660465 0.412460088 1.076780484 1.032277143 1.46328481 2.63928703 0.848207928 3.193224773 1.720610991 2.1447747 1.297280549 1.401202478 1.979329608 0.817377494 1.876908883 1.949269864 2.383918598 2.115278354 2.537858509 1.19988254 2.049300964 1.259722237 1.235220432 1.978598444 1.357305228 2.330358392 1.636204169 2.012521217 0.195701895 2.813155084 1.246827765 1.099666683 1.101112186 2.305166033 1.035073457 1.323236996 0.961592069 1.759473855 2.549997854 0.76481429 1.766137646 0.998599494 1.307966205 1.800097807 2.149294116 2.360791016 0.888073567 1.032847629 1.037419974 1.647394287 0.858015571 1.027245571 0.687530619 1.339074611 0.749211708 1.586195486 0.725802398 0.446766893 1.131728818 0.772449943 0.70082728 2.049308234 2.324647135 2.178938038 1.440025883 1.201318223 0.329791364 1.99463585 1.417307869 1.298353326 1.644705885 0.913627554 1.275431412 1.731893865 0.6331931 0.574633643 1.440433803 1.051238549 0.714849763 1.646261917 2.159077945 2.046225178 1.010535513 1.64386796 1.042509561 1.263826868 1.543606822 1.274619024 0.907018633 1.959333637 0.930682458 0.976817897 1.006986432 1.629396257 0.509151923 2.84524076 1.932793088 1.955014393 1.616027924 1.157810174 2.145888079 1.933014298 0.717073799 1.529508653 1.364684082 1.545255308 0.402079627 1.968052284 0.877462349 0.881660512 2.20274637 1.364174652 1.109088155 0.958313201 1.700923135 0.880344158 1.524059133 1.116191916 0.863677226 0.376537809 0.814614887 0.483606671 1.823061248 1.831630888 0.905621085 0.763926151 0.591577259 1.245488862 0.349771855 2.186184269 0.302588808 0.676122365 1.137832848 1.003188918 1.898906454 1.503720187 1.578918844 1.572428768 2.67648638 1.81706602 1.304337421 2.415084252 1.272861256 2.506615297 0.824429451 1.64429508 1.963597796 0.562693156 0.72251861 1.858738381 1.587657794 0.762488768 1.313887405 0.727304522 2.037103009 1.87948343 0.988080183 0.321727458 1.018777887 1.696820177 0.328457456 1.312529251 0.133591862 1.014209887 0.493402952 0.543580826 0.522220607 0.733702328 0.614004709 1.203238015 1.611209229 2.308840982 2.32521124 0.86280499 1.392849499 1.861698537 2.016495513 1.074914632 1.91729328 1.930234341 1.554914084 1.656566952 1.190381383 1.509059293 1.5078541 0.941951254 1.858140221 2.125114856 2.175502163 1.846045282 0.721563011 1.113065189 1.319740042 1.95445052 0.785391687 1.505395956 1.787538002 1.68901228 2.161026485 2.189196062 1.430369735 1.573724265 1.642117575 1.865229425 2.441473708 0.713031147 1.607697643 0.63047593 0.710481725 0.905871428 1.555701021 1.06371878 1.091231864 0.35951515 2.23942638 0.909483425 0.821051071 2.09041279 1.551853183 1.247630472 2.849100398 0.945191426 2.059607513 1.282397051 1.034153195 0.746670563 1.443404624 0.835436782 1.237707166 0.427113533 1.045906039 1.15820359 0.448169385 1.023736926 2.164657117 1.754315883 1.354474499 0.557274993 1.670577273 2.428743474 0.676709093 1.328185477 1.152500798 1.010383857 1.51043639 1.799287142 2.100615223 0.486622886 0.108133601 2.195977171 1.825997029 0.870078843 1.145237628 2.5929914 1.659340447 1.004171153 1.040577661 1.978791616 1.94122705 0.563265619 0.870901839 1.400360744 1.648685447 1.811427767 1.54085038 1.518650066 0.976688904 0.793405948 1.37736092 0.914208339 1.036950072 1.760757043 1.061773663 0.8921262 2.021968954 1.414713375 0.786663309 2.138104825 1.911319709 1.624243137 0.845616993 1.068413457 2.475411219 1.075085067 2.082897718 0.768037696 1.953149816 1.814997268 0.448986903 1.65296559 1.086621331 0.837124154 1.298172292 1.406632093 2.378360281 0.850846938 1.82221795 1.729834885 1.550226 2.328475078 0.481970933 0.603582628 0.828406728 0.714749269

ENSG00000135776.4 ABCB10 2.738362688 2.670063779 2.854985923 3.068495815 2.728611993 2.631120129 2.91647532 2.907305315 3.027695259 2.861519293 2.52544666 2.739091926 2.662188403 2.740895619 2.901930068 3.063877013 3.131153542 2.606264257 3.070206327 2.992005053 2.742669822 3.073527173 2.928531879 2.835775757 2.720884002 2.877410594 2.537226416 2.165942179 2.594387525 2.301487432 2.562652847 2.255182165 3.095539768 2.444011554 2.192985151 2.422451732 1.930282191 2.736527564 1.707346529 1.751878586 2.021730175 1.997960101 2.576931087 1.97778866 1.576317043 2.250370421 2.873591177 1.741704086 2.486017079 2.56237631 2.563507459 2.460582085 2.590374419 2.376486152 3.374371958 2.211165069 2.086550752 2.464058808 3.217090874 2.545650447 2.509201967 2.579031315 2.222779671 1.903088184 2.754709828 2.980306423 3.163334272 2.31714037 2.832717631 2.601230176 2.609344984 2.943083579 2.989653354 2.982896377 3.228745313 3.382581285 2.876933032 2.612057247 3.48453252 3.169523589 2.702302597 2.515290342 3.172038253 3.174470891 2.534090646 3.195268686 3.038280138 2.063413013 3.043850443 2.597167406 3.192632438 2.949823301 2.821410108 2.882616748 2.623060118 2.486996811 2.90381957 2.495313642 2.91779259 3.055145304 2.873826754 2.692355668 2.689436407 2.646692058 2.872879045 2.830218983 2.777599871 2.971216703 3.047526905 2.224292315 2.584978134 2.877941294 2.788001977 3.194823231 3.189331195 3.368985373 2.85371851 2.427702855 2.542956127 2.548747684 3.25749734 3.179591873 3.095539429 2.994006672 3.055088432 2.734224699 2.680166995 2.942863847 3.070910569 2.331043813 2.123196963 2.797261364 2.187536528 2.20153739 2.407221142 2.954724811 2.260289371 2.006780449 2.861033705 1.358657994 2.704237835 2.695826792 2.290989694 2.790595985 2.784602217 2.587410593 2.796862318 3.483621265 3.005554182 2.749247223 3.245809115 2.650354014 2.608405594 2.691111245 2.723277139 2.70829181 2.71077553 2.58190195 2.777325212 2.481938424 3.040634888 2.977306423 2.715924364 2.626076599 2.467781843 2.284595689 2.982649077 2.626010805 2.563249988 2.411763155 2.117555813 2.632643062 3.00708521 2.608875601 2.634753647 2.309275336 2.330812787 2.252790772 2.464997632 2.708808126 2.415299274 2.589966312 2.502244779 2.281252856 3.364761835 2.703628552 2.539848149 2.800515685 2.663096891 2.682204357 2.045995453 2.409340956 2.658798783 3.203979095 3.754881201 3.190984954 1.777275401 1.833854902 2.209303518 1.93221718 2.150201497 1.940426031 2.227987965 1.49601115 2.429908792 2.658593228 1.307540185 1.502247409 2.533240502 2.38596199 2.366153162 2.231884747 2.331701969 2.478220676 2.365559656 2.604974921 2.393286744 2.355026571 2.065179692 2.40012351 1.406087411 2.640435838 2.320612462 1.949474582 2.285319658 1.967098704 1.159936007 2.776342178 2.556349516 2.819480792 1.630433444 1.314118584 2.07495591 2.485116933 2.050268416 1.883069157 2.289639338 2.452082472 2.11217807 1.813638761 1.940149668 2.052657633 2.14553282 2.956190653 2.863447556 1.979296027 2.687848766 2.72883407 2.793151065 2.766876184 2.303361812 2.507714678 2.754772963 1.985795399 2.626192119 2.744938844 2.95137928 2.992509617 3.000727757 3.146630956 3.070827206 2.545106208 1.936114645 2.613641113 2.44000251 2.446111634 3.060469517 2.784605544 2.852758446 2.730159736 2.435574667 2.303725151 1.103403303 2.797043206 2.57612039 2.822152006 2.805563787 2.472642384 3.0654642 3.065010099 2.683150132 2.529641079 2.22461064 2.340657908 2.412816946 2.122132306 2.288298981 3.04935021 2.25796588 2.735806304 2.419108993 2.933339897 2.917174146 2.90522531 2.006701725 2.202638086 1.885218056 1.884844726 2.153996034 2.120114313 1.63124115 2.256087476 2.191154852 2.386723627 2.627589837 2.675446314 2.904290269 2.718088378 2.823226441 2.305463074 1.312213609 1.95511828 2.902904863 2.70373493 2.775917073 2.027422698 2.431473193 1.839080517 1.952154009 1.511135838 1.749475894 2.024328245 1.578873831 2.616359569 2.767404885 2.640438253 2.731548307 2.718167582 2.560674444 2.750042734 2.899811453 2.639357772 2.255671335 2.532966611 3.199438935 2.160388432 2.507019715 2.246977886 2.506489893 2.443405221 2.405923294 2.471292728 2.924709987 1.810255991 2.223024049 2.523050049 2.478810235 2.45414916 2.754575639 2.248319449 2.210175413 2.420398607 2.333293398 2.351347804 2.663837451 2.490916029 2.491381429 2.004254327 2.225803538 2.837476897 2.678808029 2.301613777 2.516038316 2.950167776 2.254525397 1.882303905 2.196603494 2.765721547 2.469794087 2.097207748 2.393969564 1.18557032 2.073375126 2.309356407 1.210943589 1.880327377 1.647209875 1.279722698 2.311428037 2.070991635 1.97789505 2.419278234 2.899064571 2.536897393 2.768216608 2.749712253 2.530138111 2.288977271 2.597960607 2.351728117 2.618478523 2.774959223 1.593265767 2.461113495 2.69917785 2.61974237 2.325917785 2.228015467 2.239821603 2.784801162 2.318692589 1.965824452 2.393955952 2.590378927 1.892004946 2.343444192 1.565045665 3.042831792 1.623636216 1.719342223 0.986998998 1.119552306 1.289122508 2.199631463 2.690064671 2.658485955 2.129571176 2.341729543 2.167520666 2.0132773 2.567276577 2.053744959 1.770710878 2.530272777 2.406134589 2.72912558 3.043280033 2.629618048 2.724843476 2.668304415 3.474154762 2.678274741 2.700476538 2.291509024 2.557352307 2.412428206 2.077257097 2.070181366 1.677495728 1.817311477 2.381051716 1.826132996 2.47991214 2.451693163 2.464273794 1.896261551 2.067980441 2.672981271 2.483246912 3.164212532 2.670591926 2.034225539 2.618758191 2.267504365 2.601237975 2.854432071 2.216875003 2.427763664 2.302374117 2.987940139 2.766242833 2.108682055 2.626512501 2.046932307 2.755839136 2.641753634 2.220871641 2.065915655 2.685334038 2.507197954 2.30683429 2.293008947 2.020906934 1.74931877 2.411306057 2.273763727 2.212720733 2.503357149 2.761541156 2.597981879 2.402464763 2.785422969 2.910074016 2.997225224 2.74659433 2.046938349 2.20354266 2.490039787 2.305585718 2.168452291 2.558900114 1.912500477 2.525050944 2.95537444 2.380631828 2.645960667 2.544143276 2.731932395 2.654739211 3.145319946 3.089027046 2.516026427 2.395670806 2.509630633 2.761844274 2.777120207 2.734087761 1.911963812 2.213399404 3.00006962 2.623146159 2.495210645 2.199207409 2.47470848 2.54255413 2.235383812 3.078011697 2.802294453 2.77385393 2.396295366 2.611184507 2.782555453 2.580343236 2.34910434 1.968450692 1.750216282 2.711674827 3.386919075 2.364202975 2.278338123 2.207104988 2.574525317 2.605174367 2.482212896 1.78966639 1.613260269 2.263450301 2.408253411 2.220362689 1.717836658 2.697458152 2.74898372 3.214271064 2.250395043 2.570230281 2.147404487 2.139380684 3.578520667

ENSG00000005471.14 ABCB4 0.10932692 0.306876054 0.152868735 0.29435995 0.188907281 0.280871942 0.066656294 0.152130268 0.504619925 0.142434044 0.21852111 0.112155045 0.176510934 0.321529739 0.204495305 0.284642016 0.262929646 0.30190967 0.331489944 0.194160117 0.182658067 0.158350234 0.314616181 0.184348921 0.406281331 0.157254113 0.244338906 0.138183834 0.091960817 0.053564609 0.14714132 0.212864134 0.064804635 0.174478785 0.124287608 0.127923628 0.147948102 0.095900993 0.111985089 0.140339619 0.105266565 0.135088082 0.158368894 0.11913456 0.082663816 0.12097601 0.103760173 0.073666659 0.210118287 0.180062798 0.303056294 0.235693115 0.188486428 0.089840587 0.107134037 0.117091267 0.050647965 0.068349882 0.097876495 0.095344678 0.19275283 0.207168863 0.817154039 0.094452115 0.253907328 0.165175002 0.169562913 0.309959227 0.179974483 0.132972108 0.227939448 0.137422573 0.110409258 0.180156465 0.496109778 0.122545907 0.093660588 0.188289856 0.323876426 0.293290642 0.230475867 0.158952661 0.101691452 0.222474116 0.155291847 0.14385028 0.246713158 0.265989754 0.138618592 0.216124153 0.125222025 0.171221885 0.233415732 0.307015089 0.184146378 0.117195958 0.314662065 0.076834675 0.190242879 0.18316248 0.107497179 0.122327614 0.186628523 0.228205116 0.117476048 0.103419477 0.18261078 0.268854759 0.2471819 0.190438481 0.218686708 0.098505734 0.142692397 0.264257607 0.161771429 0.279504698 0.158926742 0.303463009 0.140345746 0.099715196 0.222074504 0.240513311 0.418629569 0.243466956 0.264815404 0.148019559 0.205821474 0.123345255 0.34400303 0.165990096 0.093658335 0.093031929 0.102668251 0.05762492 0.096755678 0.362753694 0.070413077 0.126194533 0.102141454 0.108463779 0.303506813 0.188190083 0.306775192 0.11393649 0.205961845 0.199370827 0.216776091 0.463444742 0.231717786 0.193136184 0.16198283 0.092374852 0.191741746 0.273124912 0.149160068 0.21358837 0.185663999 0.248055554 0.241598723 0.202851693 0.153580993 0.179722355 0.186296165 0.130948668 0.163125784 0.039502305 0.211508106 0.310584149 0.19661402 0.35212753 0.047168606 0.095770862 0.215783602 0.084448205 0.10731004 0.096999291 0.08280719 0.19639067 0.045812724 0.094968028 0.190820589 0.106927041 0.150158262 0.212656653 0.208743816 0.422812181 0.13520992 0.183240314 0.054151079 0.173685321 0.130060286 0.133479109 0.225322124 0.24809954 0.126443943 0.084865581 0.119951945 0.035334022 0.061840626 0.111521518 0.118178814 0.128834627 0.064354393 0.604704727 0.123899241 0.114167567 0.057179424 0.06125238 0.138094328 0.090006312 0.134098039 0.098933695 0.179418037 0.185061679 0.034228252 0.13907769 0.235925112 0.135297094 0.233361956 0.099980579 0.049793004 0.144063318 0.022006497 0.115593955 0.157519628 0.113230224 0.183703255 0.174928833 0.107119367 0.112621877 0.075191638 0.132882005 0.066702428 0.163679912 0.169897694 0.126253082 0.138335165 0.18301538 0.471463177 0.147980947 0.157177719 0.047123514 0.087239892 0.196821971 0.118626067 0.037193199 0.063958108 0.09829275 0.123272558 0.453703263 0.061524645 0.518721004 0.147598779 0.311543797 0.159484845 0.161827923 0.21808548 0.047642419 0.154393481 0.104549601 0.215808667 0.213955798 0.223319294 0.110791999 0.571820794 0.223122357 0.143354799 0.234996407 0.102012564 0.271605954 0.259678541 0.052788714 0.06141396 0.31538925 0.133917705 0.061400504 0.119696957 0.306984155 0.147198491 0.185140857 0.085105609 0.103421303 0.686015875 0.146783167 0.306154424 0.117745154 0.135411177 0.218382811 0.53118505 0.269433839 0.1474444 0.243684126 0.165067335 0.380765425 0.106559364 0.148111777 0.051301684 0.152225489 0.070561514 0.206013396 0.128803039 0.084956372 0.165612679 0.052316741 0.179922805 0.272937939 0.188799441 0.429746408 0.188939893 0.215795912 0.136835198 0.129659189 0.182570893 0.231563483 0.278425739 0.095878862 0.10941007 0.185549163 0.073022746 0.126951888 0.180757793 0.15974409 0.105213261 0.107341567 0.633420627 0.146766108 0.077249544 0.221826691 0.066838029 0.151232672 0.077183534 0.143381895 0.069371065 0.135121706 0.152586903 0.09646952 0.131881043 0.284336573 0.03954122 0.64961799 0.3228412 0.207585718 0.265495608 0.047632023 0.182748356 0.21487652 0.084418357 0.129003103 0.18637028 0.172918145 0.067350494 0.242876822 0.184830871 0.058081798 0.154507301 0.252868246 0.17323777 0.281062309 0.299526576 0.11484208 0.143429326 0.119925632 0.109714222 0.112697425 0.113022671 0.110421831 0.179136551 0.229951249 0.114600508 0.06892515 0.107206943 0.133650143 0.086914641 0.240269722 0.066486559 0.117993837 0.229791864 0.160158515 0.223534378 0.189479718 0.217733638 0.237614287 0.378525316 0.197307458 0.088861013 0.25420802 0.163529732 0.190435896 0.174318853 0.17009434 0.227247941 0.036198527 0.191876389 0.371916374 0.193044265 0.07424093 0.072469706 0.146764668 0.1687138 0.21425168 0.123861471 0.034489296 0.173220822 0.168988113 0.071924264 0.150262759 0.009265395 0.327375366 0.062342926 0.076615092 0.073809748 0.107785186 0.072278692 0.081387147 0.184364882 0.39352661 0.221982223 0.065440182 0.145130235 0.204070183 0.134443191 0.143094346 0.200564437 0.292302643 0.158060118 0.127836895 0.078867518 0.35058064 0.310736456 0.155218742 0.264267347 0.437527943 0.277306035 0.250933435 0.06811379 0.207102397 0.207758383 0.235443547 0.115098473 0.140644797 0.196214204 0.19052215 0.146058866 0.261828772 0.15613763 0.18523304 0.14124234 0.317504224 0.324249565 0.154812359 0.308464894 0.159140744 0.04213265 0.082195274 0.173018203 0.09053114 0.123023337 0.093719242 0.306582638 0.126683415 0.112983665 0.250337066 0.160065454 0.092960626 0.55236004 0.128807522 0.565280324 0.137019411 0.164298512 0.081126002 0.239705301 0.088952964 0.240890524 0.148120879 0.077229387 0.080422447 0.086597296 0.151420413 0.301521672 0.17966442 0.166835777 0.0691086 0.175960976 0.361774626 0.068114858 0.254798857 0.13844759 0.113643078 0.212842227 0.178860063 0.105383091 0.038145615 0.039332295 0.279143474 0.138508795 0.063727687 0.185935969 0.299505223 0.358006715 0.168296222 0.17543962 0.302635139 0.192053227 0.053829754 0.119362692 0.21549709 0.19115959 0.172686567 0.144372778 0.184620348 0.253730956 0.18691363 0.105238646 0.075926439 0.171720044 0.141192869 0.247601126 0.071775172 0.272982039 0.152871139 0.091287803 0.3136228 0.306592487 0.133224366 0.405174587 0.09764929 0.356576554 0.134379862 0.262886258 0.182774053 0.289286603 0.173190907 0.102995208 0.117680723 0.22607224 0.192991615 0.884441496 0.250880103 0.374324913 0.165051889 0.198822369 0.290355479 0.135139073 0.571686342 0.372093221 0.042604247 0.094530165 0.194627176

ENSG00000004846.15 ABCB5 0.016927224 0.023440431 0.278933275 0.062088794 0.051681515 0.001341743 0.009175357 0.049110233 0.029745897 0.028693332 0.022180162 0.020578335 0.00733632 0.019681273 0.040663609 0.040192577 0.047611065 0.022479437 0.031886832 0.01828597 0.063089118 0.046774595 0.027576337 0.009199247 0.050111253 0.023668091 0.033479379 0.007982372 0.005291716 0.012935441 0.035392177 0.009371317 0.005355915 0.207308847 0.087367406 0.01699799 0.081726175 0.006674133 0.041001009 0.754439964 0.052699809 0.075454246 0.08194481 0.024918399 0.051730348 0.017407607 0.027115779 0.148244687 0.021522978 0.017148465 0.003064278 0.035102462 0.043485339 0.004407538 0.002997943 0.018986277 0.003206453 0.023145693 0.043265512 0.029254673 0.010396654 0.018860686 0.176936275 0.009266904 0.103672373 0.031458589 0.004407259 0.003409646 0.027271844 0.001523039 0.53298069 0.032969318 0.030636034 0.105227373 0.107958422 0.03410772 0.256076741 0.031607375 0.110269527 0.041290447 0.35902224 0.02346664 0.036099586 0.022295978 0.011596422 0.13294722 0.055732265 0.039138858 0.055026041 0.05149981 0.039176847 0.011572407 0.037953848 0.066750944 0.014963402 0.164652812 0.09039622 0.010052417 0.029625889 0.019628744 0.012494143 0.036800373 0.058839434 0.026098886 0.012332386 0.131659542 0.134420076 0.051941013 0.048111563 0.051508247 0.006892907 0.029546913 0.039916774 0.061286408 0.063454259 0.024451228 0.021097042 0.02125482 0.007803566 0.044883352 0.031387549 0.056657712 0.022680844 0.105815617 0.036370042 0.057972038 0.027064011 0.012972374 0.032975621 0.036457297 0.050637874 0.017185086 0.025614421 0.019695948 0.417087711 0.047507168 0.012643747 0.009458877 0.042219187 0.01563767 0.153800451 0.022660701 0.013770557 0.017746631 0.007151258 0.016064803 0.072167786 0.042419241 0.020827453 0.022980888 0.027953511 0.047823601 0.044141048 0.007141808 0.011020027 0.019233879 0.02022635 0.093123661 0.62817478 0.009373442 0.012323671 0.127842547 0.031843273 0.078895901 0.068266033 0.007186715 0.132076214 0.007175687 0.008501139 0.058115394 0.00516626 0.041093673 0.01903602 0 0.015827907 0.064157456 0.060798669 0.002700923 0.024905654 0.107720597 0.071006244 0.020147668 0.015925772 0.058628326 0.009374248 0.014135017 0.013596735 0.013689162 0.023630004 0.038794943 0.030525765 0.014906997 0.04045102 0.055372113 0 0.003212956 0.022685468 0 0.05683727 0.011401574 0.015027419 0.025150831 0.016553886 0.011428291 0.007205121 0.020647157 0.007848468 0.020120853 0.023702642 0.035928958 0.025838217 0.003450111 0.05562457 0.06600881 0.00699322 0.04112445 0.030641966 0.034730616 0.014875011 0.02995156 0.035447826 0.052684763 0.020055855 0.005256905 0.032078585 0.018946771 0.064908482 0.028981861 0.037413401 0 0.007789626 0.017816598 0.037943038 0.042085099 0.015634476 0.011563717 0.019784751 0.023214861 0.009136967 0.002572347 0.004970438 0.011037299 0.065471419 0.014355187 0.013284298 0.049067131 0.014748151 0.007041034 0.019065053 0.050419044 0.001955771 0.112665777 0.013912117 0.01776401 0.010463323 0.06882573 0.041180614 0.018336065 0.04331723 0.019376037 0.030650422 0.03539557 0.098353328 0.062202372 0.213931531 0.026159381 0.020620354 0.063347574 0.0747511 0.053389064 0.024254822 0.023548517 0.002984618 0.054103211 0.219153938 0.018588035 0.06830384 0.047483706 0.026873721 0.032938344 0.008491351 0.022227535 0.407887589 0.049650252 0.080754105 0.045470423 0.018272817 0.002325853 0.049140571 0.021094653 0.012513204 0.004565409 0.006420917 0.013767578 0.013501897 0.036493022 0 0.021004326 0.024874197 0.005505587 0.026201545 0.006425828 0.141114943 0.162960906 0.005734097 0.053905558 0.059119862 0.016447481 0.018044894 0.172658028 0.00772583 0.023553035 0.101912491 0.170513736 0.083479778 0.251844761 0.029467518 0.005613576 0.003364805 0.041712554 0.007972529 0.044878699 0.302633407 0.017930666 0.019674764 0.014639381 0.070520176 0.023418249 0.028857697 0.032532542 0.005338904 0.008690821 0.013473689 0.040588688 0.02670894 0.003103088 0.0038499 0.027006465 0.0064761 0.053384292 0.17678919 0.037952245 0.026399835 0.003263315 0.025523496 0.026089052 0.01941869 0.040016896 0.101061746 0.031121985 0.008786288 0.027369765 0.043169667 0.031636848 0.015270621 0.011144114 0.031034775 0.031552111 0.071686238 0.163852932 0.143743232 0.021976933 0.027087406 0.011813028 0.013533032 0.022347686 0.034958149 0.08978305 0.007603018 0.003004905 0.008167327 0.009684545 0.008344021 0.037976695 0.004583019 0.256988935 0.531505724 0.006363939 0.033440089 0.043063439 0.048689449 0.071272087 0.036566209 0.079464684 0.072353972 0.033517502 0.011965676 0.018196452 0.142703145 0.016894399 0.072171158 0.010750837 0.022277993 0.043543287 0.062559687 0.021294244 0.008996835 0.014223022 0.064399661 0.16455893 0.039718311 0 0.036776427 0.046497045 0.007443525 0.086392708 0.009998518 0 0.027507791 0.031503595 0.030332658 0.040052506 0.061091923 0.029814603 0.003180665 0.002999048 0.01525601 0 0.011912257 0 0.021421171 0.033437719 0.016039646 0.034108074 0.226377221 0.033910064 0.064175786 0.191514883 0.025013526 0.045422108 0.002522362 0.015406438 0.031929237 4.652252005 0.014979012 0.016674498 0.011624263 0.014047606 0.037610885 0.037105302 0.010380149 0.063947002 0.003883347 0.055037108 0.005225611 0.011384078 0.020741841 0.02887709 0.004082196 0.024406293 0.064813209 0.201746254 0.021859002 0.024686919 0.011401885 0.026071799 0.016538938 0 0.122590732 0.068918498 0.302834477 0.012414366 0.121731913 0.003529032 0.043429245 0.003514193 0.030982044 0.035124081 0.063597704 0.02145792 0.231773252 0 0.034143303 0.493210896 0.014202021 0.015668676 0.112875651 0.017130734 0.033541873 0.134640771 0.050645763 0.006353598 0.024525111 0.016278347 0.035491982 0.075440553 0.040807234 0.007344368 0.024545394 0.036530638 0.021958535 0.012463067 0.085453396 0.089840931 0.004197061 0.013997775 0.005091156 0.014571176 0.08204383 0.009194621 0.008856504 0.035634358 0.003020474 0.0205731 0.014847771 0.00922415 0.107953618 0.035774498 0.104963778 0.023146549 1.30706369 0.033970384 0.028008025 0.01427289 0.347071345 0.016136834 0.146017543 0.010475154 0.080641277 0.015458714 0.059776499 0.034489421 0.017181458 0.040112946 0.065327976 0.111231892 0.026310228 0.059132154 0.028022171 0.020212086 0.44097639 0.028465797 0.020973673 0.006829447 0.011171846 0.05160455 0.028118717 0 0.029026385 0.03809999 0.018052379 0.075818211 0.030668855 0.138020596 0.037685712 0.004992005 0.095621976 0.005550277

ENSG00000115657.11 ABCB6 1.704215357 1.470314264 1.358038443 1.709896507 1.683583021 0.887968663 1.049248424 1.282634105 1.007519615 1.428733911 1.290026297 1.319663261 1.00679797 1.105424307 1.343661499 0.959930237 1.130881386 1.031274736 1.139953635 1.255232318 1.180205206 1.602442175 1.358139815 1.217462953 1.192501995 1.312807866 1.42307244 1.319951499 1.760717897 1.323356149 1.926604324 1.545059178 1.630817163 1.521327045 1.806603789 1.371776905 1.605322091 1.601297438 1.047117177 1.336265201 1.25671076 1.302612069 1.752054918 1.496323174 1.162609816 1.336647719 1.466675745 1.173922423 1.590142872 1.670523007 1.407361861 1.554979526 1.597505515 1.19514782 1.680295472 1.894237926 1.764700126 1.579543843 1.529970106 1.353684049 1.346833177 1.270224102 0.97592815 1.345647894 1.354421758 1.384618936 1.792827761 1.719610826 1.163705552 1.481353587 1.673612818 1.549096615 1.592680396 1.55370123 1.685192794 1.78129666 1.280956756 2.115525461 1.797096821 1.812182763 1.17515085 1.416677256 1.385852171 1.496702017 1.261826625 1.662986481 1.551008088 1.386254747 1.302081404 2.183294467 1.352975286 1.502522263 1.382180519 1.56329623 1.794416444 1.615470177 1.327548869 1.553741777 1.339880453 1.196117686 1.472927469 1.560061436 1.361496444 1.672751364 1.684301342 1.498392784 1.183726781 0.912044363 1.229813632 1.456037823 1.371339002 1.352892839 1.135841566 1.211168155 1.359895083 1.350698406 1.104412234 1.700323365 1.806650156 1.934210533 1.386024656 0.90701792 1.719802308 1.373553729 1.208955798 1.568721598 1.547642989 1.508256424 1.215167091 1.68582819 1.629484504 1.422267319 1.541757239 1.806715931 1.525763627 0.738012474 1.616578975 2.219952392 1.487098059 1.809076475 1.365084388 1.581111687 1.255377966 1.281429662 1.169313978 1.728109212 1.55578029 1.472411038 1.53129835 1.504435542 1.481287845 1.872692871 1.464669275 1.141566937 1.34538267 1.528909545 1.450255768 1.175243807 1.412688866 1.674020293 1.763564954 1.601067233 1.647730239 1.747227105 1.67352437 2.061826489 1.61587507 1.341496448 1.643008612 1.897304306 2.533135481 1.753471483 1.635201244 1.514681188 2.006513004 1.908276045 1.600679423 1.280790132 1.990237098 1.42803155 1.780864144 1.640996729 1.689668816 1.301113276 1.449603727 1.292193165 1.659452322 1.651062321 1.602897067 1.459371526 1.610560546 1.540404097 1.449999324 1.868878901 2.39272272 1.383975294 2.146224266 2.16366909 1.627275058 1.445917958 1.653169658 1.519646648 1.586488259 1.288479999 1.527703752 1.88805981 1.476046613 1.447096339 1.454630871 2.002801652 1.497309831 1.548294965 1.775941705 1.815609067 1.041679581 1.375286715 1.782226889 1.479352674 1.567704852 1.501310412 1.670631444 1.156043941 1.815608017 0.944969755 1.551746377 1.152219166 2.130373033 1.366022832 1.466681299 1.473796994 1.39618863 1.088873637 1.77778074 1.721397743 1.46934096 1.370621858 1.467642237 1.528491924 1.320289625 1.820797441 1.948694095 2.284561269 1.586501648 1.51675357 1.546340347 2.428183164 1.289036582 2.102590084 1.718739504 1.212350738 1.573340201 1.270803494 1.55772662 1.38168462 0.963624505 1.864651586 1.791686643 1.984496298 1.555212991 1.361241711 1.174431459 1.442608519 1.245070904 2.013512589 0.900497616 1.412999393 1.75745478 1.374527465 1.567702283 1.302064623 1.719898445 1.312022681 1.145181004 2.044833625 1.650125974 2.008810341 1.76890779 1.864074002 1.567810407 1.567171947 1.990808641 1.9387754 0.660932645 1.91554959 1.700341516 1.869358253 1.67757586 1.476104023 0.61348005 1.54028897 1.946557834 1.997547442 1.169573268 2.021853881 2.075218463 2.396080007 1.869707719 1.99577686 2.138240664 2.190042349 1.889624914 2.269835062 1.37164469 1.904783073 1.61571219 1.613669728 1.65474271 1.808621783 1.757516357 1.529204336 0.991593345 1.322076378 1.972252229 1.332171731 1.368272437 1.91734104 2.096354273 2.000813312 1.911352098 1.599643625 1.537087618 1.956775728 2.232799726 1.761715277 1.486984522 2.053659248 2.00575192 1.990997935 1.643214638 2.009492711 1.43535026 1.789620353 1.955533166 1.463618993 1.663752358 1.522357212 1.728735217 1.718435646 1.663739731 1.840413076 1.950785347 1.998588065 1.663437053 1.224718416 1.799089288 1.588843373 1.471851366 1.378890774 1.659525052 1.613057519 2.402666201 2.075401159 1.801156318 1.42705085 1.310294929 1.670655044 1.834667778 2.085511599 1.660628527 1.912395466 1.515860901 2.715693708 2.421341112 1.932986012 1.620218156 1.913012767 1.83739426 1.790482244 1.943883917 1.843297912 1.390790471 2.424455304 2.311718788 1.755576875 1.786687167 1.801609467 1.996322184 2.106405416 1.968412866 1.716198035 1.99800733 2.222775119 1.404371016 1.849609168 2.026715345 1.783892732 1.960987086 1.831821154 1.752728213 1.612206591 1.107309311 1.068646217 1.782849928 1.327185417 2.037092674 1.966787785 1.327319321 1.522126141 1.280300306 1.550916835 1.824450017 2.284055801 1.663585603 1.839867647 1.79498733 1.068698339 2.053414116 1.771868144 1.72188634 1.90250082 2.072892619 2.414859913 2.089044341 2.068232835 1.781403864 0.969777667 1.640409538 1.645726185 1.519934096 1.960226476 1.674879554 2.211704419 2.338045601 1.786908887 1.534195121 1.705449082 1.691753924 2.005355126 1.719814793 2.172835227 2.131892316 1.374616561 1.60079021 1.218920725 1.582733558 1.820752829 2.069587125 1.379045756 1.855096244 1.68321874 2.030839677 1.72047858 1.494052753 1.697189752 1.42111474 2.255777248 1.654094566 1.138806234 1.710522577 1.98630711 1.724438464 1.742616602 1.403064215 1.637034425 1.327638914 1.520499795 2.414629831 1.523410641 1.388469066 1.81309262 2.090720679 1.734885647 1.617523148 1.567758813 1.332252986 1.319851196 1.264650921 1.641456808 1.721478376 1.860638022 1.656313516 0.891123786 1.854544677 1.659922474 1.55136403 2.13705925 2.026038359 1.788124984 1.768811269 1.969034451 1.85994879 1.432667401 1.243862862 1.725669072 2.249631639 1.793153021 2.345358265 1.788174524 1.73969777 1.397414881 1.755996824 2.107627824 2.922930506 1.393634937 1.885324187 1.504880916 1.428281481 1.392222366 1.506385573 1.592843732 2.048152776 1.306043575 1.543930629 1.583045858 1.305590048 1.547975343 1.63185022 2.122139623 1.328278254 1.55423437 1.613787107 2.001553736 2.042766011 1.644042542 2.040173036 1.456479829 1.976776945 1.920846429 1.817771309 1.833294877 1.952247622 1.532706484 1.68932886 1.62987746 1.436183327 1.63062166 1.455250869 1.666491129 1.547385729 1.381768145 2.003824117 0.770255662 1.837392432 1.188085775 1.714230774 1.332896489 1.617626762 1.47586718 1.24261364 1.683963603 1.182048198 1.617391999 1.179389195 0.831860583 2.164502126 1.964770011 1.57551742 1.753573605

ENSG00000131269.14 ABCB7 2.736593898 3.032834268 3.10855841 3.169210793 3.20019075 2.926111535 3.343050714 2.833936134 3.136142924 2.775162389 2.854723665 2.933922606 2.816287153 2.928139817 2.866457312 2.967939523 3.178295724 2.96070946 3.002767484 3.187515568 2.989206504 3.002707298 2.993491966 2.922096773 3.126559609 3.088070093 2.789501629 2.580246188 2.78388024 2.283412841 2.657173656 2.623939031 2.323436201 3.067305051 2.525573001 2.784910665 2.281831301 2.755324092 2.184627272 2.60580379 2.521221129 2.453734722 2.914228306 2.517091561 2.463749054 2.589650466 2.764535095 2.314617279 2.737369103 2.611661006 3.070828807 2.768724797 2.433498043 2.547117154 3.417710578 3.05334864 1.497692547 3.047176574 3.167231194 2.763621053 2.336313039 2.961237945 2.626675394 2.81979403 3.139312838 3.487081819 3.378873107 2.964790982 3.04929798 2.669687052 2.771320535 3.091506005 2.994435399 3.313749637 3.839133301 3.767952641 3.218156937 3.124740461 3.263897133 2.956275041 2.930059996 3.597434513 3.104773195 3.020455355 2.837474612 3.384212444 3.219349567 2.858715689 2.908264101 3.259216566 2.757618009 3.343949629 3.345821386 3.051750056 3.049456719 3.119900969 3.264458492 3.254440989 2.97354126 3.178317807 3.395198761 2.991792427 2.871208511 2.833859444 2.931521588 3.020533117 3.10153188 3.07871389 3.289942619 2.987341958 2.752114183 3.180958489 3.07895121 3.587781123 2.965304548 3.287439371 2.764320713 3.264206564 2.863119782 2.868576256 3.18008477 3.228084528 3.632108532 3.253863942 3.304054411 3.131160747 3.042642406 2.917389081 3.290776931 2.842081544 3.153938923 2.659016216 2.794275633 2.512026315 3.055540116 2.960210907 2.574083837 2.652968852 3.561886252 2.687427604 3.354298447 2.992297115 2.879152964 2.988089438 3.277859468 3.097359301 3.242474453 3.187772916 3.105195126 2.824436551 3.08324292 3.145337845 2.904599728 2.974033907 2.888492083 3.179994877 3.18800079 2.734952935 3.387452745 3.178796282 3.091562569 2.93650696 2.679936921 2.942062146 3.177749114 2.826545659 2.751782928 2.719631432 2.795048986 2.667312497 2.844641677 3.065359014 3.191371558 2.749917002 2.686677202 2.770756533 2.808858129 2.835616595 2.864375919 2.876957029 2.770614046 2.819967043 2.782722644 3.042500028 2.945802424 2.755731655 2.832103828 2.781606139 2.980155402 2.848276887 2.559273625 2.599529059 2.91386075 2.877491667 2.369152499 3.094195467 2.553162307 2.764416073 2.728702738 2.688294685 2.673171052 2.32862568 2.65578457 2.380664717 2.331678983 2.849117045 2.055455388 2.255657851 2.898893001 2.625655497 2.5990693 2.405576818 2.857681254 2.861893761 1.49436484 3.17570357 2.665830651 2.753030454 2.46287262 2.528080551 1.813203962 2.841192752 2.661731776 2.90845562 2.838111579 2.44841735 2.353810738 3.179413025 2.613031337 2.677798191 1.656033488 1.923757389 2.619310597 2.754146334 2.712599524 2.286237211 3.010460669 2.716816174 2.626058443 2.776433138 3.025472153 2.982900562 3.10224845 3.052856565 2.669662485 2.525182874 2.859718292 3.017169932 2.861296776 3.07503724 2.710911181 2.936106336 3.139158683 2.783240953 3.118019771 3.225894047 3.12558817 3.183158492 3.195199455 3.004918373 3.297819487 3.141938133 2.79434828 2.83651011 2.904748427 2.901688449 3.471895077 2.853832996 3.072822231 3.081258227 2.956798846 2.75741451 1.619161803 3.172341326 2.971120305 2.780759594 2.833603886 2.896105267 3.1329378 3.345251115 2.926995557 3.139040903 2.769129285 2.831194893 2.903815192 2.899571108 2.795833423 2.790563147 3.216281997 2.861906354 3.120588822 3.095492499 3.178983639 3.284175532 2.570735074 2.958584083 2.500373037 2.553705436 2.505809687 2.620164569 2.528527297 2.868451453 2.996273499 3.128094596 2.830513774 3.347574056 3.054638379 2.84088846 2.967357597 2.923019353 1.904842249 2.590051932 3.162828206 3.039505011 3.071587867 3.275266065 2.79505015 2.635272275 2.74855242 2.426179657 2.557753248 2.754259705 2.567314981 2.677772922 3.322164616 3.004539941 3.189822887 2.885010845 2.987918277 2.879540313 2.224874866 2.810207976 2.749482338 2.93444859 2.771645805 2.704121723 3.793644014 2.714921369 3.038193319 2.893081736 2.990506977 2.817274506 3.078933775 2.582579264 2.614738761 2.878904039 2.681378733 2.727694579 2.862300746 2.993411248 2.278708756 2.777693447 2.823635068 2.646950411 2.7422929 2.618237399 2.971817998 2.58618497 2.383813611 2.914320775 3.088181323 2.730887598 3.316775785 3.203688741 2.253172563 2.716147194 2.770589346 2.936289739 2.890925985 2.408330739 3.081896002 2.243905225 2.869777485 2.72500539 2.382592922 3.015609968 2.694231297 2.696909788 2.835811432 2.9964839 2.5340132 3.245727127 2.599075218 2.727393764 3.194855962 2.838714025 3.345301762 2.490757423 3.026973496 2.78887256 3.241157103 2.530868072 2.60021325 2.736441107 2.91758497 3.302119795 2.685573653 2.248501126 2.874123966 3.030130727 2.843388689 2.828785732 2.958159355 2.873726883 2.535185672 2.505350018 2.775497776 3.01453889 2.201609572 2.304323075 2.178393332 2.567325147 2.461020947 2.700320531 2.917593901 2.603716731 3.016273836 2.892246161 2.537700547 2.802978826 2.774278215 2.550911406 2.313948674 2.635094418 2.435550514 2.949130117 3.123785039 3.11161551 3.02398867 2.789310662 3.456231332 3.091590376 2.983866545 2.961784569 3.036557895 2.799155175 3.258025584 2.599914909 2.187418518 2.421003537 2.517614717 3.718833678 2.856374437 2.941736001 3.001181231 2.590906053 2.86939752 3.303950645 2.61618312 3.457317026 2.814878106 2.172658328 2.6698052 2.044998538 2.677958376 3.177871782 2.601002253 2.817942935 2.957540424 3.149564633 3.557555423 2.815658671 2.854379746 2.584955444 2.627054333 3.150991461 2.662198409 2.704132327 2.964771181 3.250623724 2.677185188 3.064176777 2.229446045 1.940762468 2.570738917 2.573414401 2.552914108 2.729243614 3.159333307 3.016888514 2.434265423 2.451789293 3.11463604 3.2079839 3.43119057 2.854893051 2.305643039 2.927304762 2.928647152 2.428355566 2.885500222 2.762778931 3.149581712 3.271986686 2.792784116 2.676702721 2.741044725 2.86339669 3.355977069 3.162075227 3.261819171 2.682896652 2.706279499 2.910063919 2.733486562 2.801349773 3.090668394 2.7418104 2.82483982 3.141090162 2.85145529 3.29277727 2.698529752 3.388203406 2.843446932 2.650707986 3.193171745 2.721373779 2.835908133 3.034248557 3.02985663 2.998961145 3.135584311 2.849994773 2.809380834 3.123301138 3.200084736 2.731447416 2.983288931 2.832562436 2.907148271 3.056161009 3.162586262 2.609370445 2.623143895 2.79353234 2.737299476 2.724646061 2.938066412 2.805607224 2.859912453 3.469782636 3.354460451 3.159967666 3.559452039 2.697817435 2.584450051 3.533111472

ENSG00000197150.11 ABCB8 2.400089141 1.989954281 2.101297844 2.039286825 2.453301177 1.8371715 2.257927511 2.276605673 2.056061386 2.400185349 2.409385042 2.534178949 2.039900401 2.154925711 2.313264501 2.017229776 2.257850769 1.878220902 2.097464213 2.288779844 2.097000511 2.588288266 2.278732865 2.204944546 2.069674041 2.376469414 2.163899091 2.035303979 2.457471444 2.303477624 2.508708601 2.155553092 1.735838561 2.44495485 2.406577608 2.365736299 2.256034729 1.810338046 2.122905302 2.015895541 1.841739514 1.754645097 2.447815024 2.147928894 2.205232826 2.31905932 2.439902248 2.061494349 2.502846658 2.643469192 2.06251859 2.582080417 2.441541032 2.321529236 2.726943395 2.012290927 2.040167003 2.58242534 2.072363033 2.655181475 2.493806388 2.628607961 2.599643046 2.531300029 2.055356562 2.219802268 1.683264502 2.26749236 1.846969203 2.434011 1.876702876 2.025487601 2.245944656 1.879961596 1.922024699 1.978085392 2.37493688 2.594039078 1.533724035 1.817678149 2.147459724 2.147592208 1.977385045 2.391889375 2.243484301 2.085559299 1.870116358 1.805962625 1.605537204 2.589659491 2.232169088 1.914933734 2.667613698 2.396502961 2.12813886 2.388333647 1.671972052 2.049852155 2.072137369 2.049739997 1.92316615 2.556994452 2.207290117 2.76310268 2.186404444 2.333117114 2.132773991 2.254993612 1.834205478 2.507218498 2.293327104 1.875128536 2.300122644 1.842092161 2.227203156 1.722774048 2.078465423 2.579807815 2.695685223 2.834308032 1.931552694 1.883324614 2.186106174 1.741681688 1.837950498 2.092394905 2.629098874 2.683199289 1.983309534 2.309408729 2.595135774 2.458695458 2.77474379 2.771456921 2.293068942 2.301987275 2.736869508 2.643202384 1.590093297 1.96837935 1.949330164 1.816940545 2.41457058 2.364878648 2.251277893 2.661327138 2.244822497 2.605799548 1.913356187 2.351637346 2.182295273 2.99864008 2.436805888 2.002929142 2.327691648 2.471502587 2.083738685 2.219788781 2.200461342 2.457306204 2.232709759 1.838612905 2.650230405 2.324781347 1.959176052 2.599450549 2.166958482 2.402299844 2.475591551 3.290297999 2.482023688 2.448432641 2.149688439 2.528240965 2.950238805 2.550634667 2.608836828 2.409390009 2.214413087 2.301706104 2.770562277 2.949557583 2.526106681 2.310413864 2.924260942 2.706103416 2.588254403 2.480270835 2.234091576 2.452227088 2.413731866 2.467321632 2.273885173 2.533604505 1.62476108 2.986194234 3.042235799 2.789402433 2.78444523 2.20311463 2.371164038 2.917368132 2.240542057 2.181647566 2.210678444 2.702995354 2.605086585 2.578917792 2.087682599 2.428211422 2.755601884 2.39218698 2.020722321 1.982708865 2.318137289 2.40485921 2.53068545 2.049880301 1.936927728 2.569899109 2.442096768 2.285627258 2.302842103 1.938895529 2.174913548 2.152083301 2.849619709 2.393802314 2.539615717 2.639914475 1.753913179 1.924135925 2.329965615 2.234663213 2.48365505 2.499176618 2.842664474 2.387221544 2.279108645 2.348978554 2.627812907 1.730624638 2.625226955 2.231157309 2.604949857 2.484550378 2.38145104 2.480932205 2.628025988 2.118999202 3.077868469 2.490131172 2.194699586 2.550522149 2.102813185 2.263528329 2.462350028 2.361110155 2.281893738 2.055633093 2.155243827 2.366136121 2.100248209 2.36379214 2.030264023 2.769052308 2.175838082 2.121277073 2.331064633 2.234059682 2.675017745 2.26850706 2.033676655 2.616594247 2.348399563 2.283986933 2.439962933 2.931079081 1.834198061 2.224315666 2.474984057 2.249854418 1.602747028 2.441561127 2.588347561 2.71561237 2.072793621 2.175675835 1.478438792 2.310918133 2.911812932 2.350840716 2.145830664 2.25892938 3.045970301 2.950304303 2.885702704 3.058487726 2.781232078 2.699380009 2.952696591 2.667047822 1.905627113 2.335002372 2.508220618 2.138426916 2.250124726 2.550933899 2.514563975 2.272045146 1.937246777 1.998123072 2.290600856 2.323012199 2.17590128 2.110817107 2.911000308 2.832123679 3.475089816 2.615467455 2.52011931 2.698560604 3.08086295 2.556206944 2.389839152 2.326164262 2.355873736 1.763870505 2.958132655 2.333147392 2.368783604 2.215544391 2.509946963 2.404445175 2.593689335 2.850991289 2.910304876 2.239456891 2.454435081 2.657086815 2.318478311 2.507424264 2.076398813 2.445739832 2.587008018 2.351269962 2.387485562 2.235447301 2.278583215 2.586043946 2.981304171 2.355727598 2.683338929 2.420774144 2.621805737 2.157031506 3.281950023 2.86798093 2.138693264 1.913854252 2.559835779 2.997860367 3.038908229 1.821847926 2.863875761 2.721918837 2.866340799 2.164620062 3.396450299 3.172026945 2.38456953 3.333162507 3.175457569 2.748009706 2.783198097 2.455571935 2.771185845 3.143665081 2.704246996 2.63922354 2.459826328 3.030071446 2.349334792 2.222056507 2.626686411 2.873526355 2.151723157 2.547907482 2.450378888 2.360232462 2.491266741 2.830836693 2.340358737 2.958038962 2.473284397 2.333358099 2.53215117 2.846253488 2.065760729 2.267896924 2.621393235 2.363236532 2.552816585 2.893003024 2.388622318 2.353878282 2.758042643 3.073794891 2.118675505 3.201288515 3.164505698 2.546422245 3.303500735 2.520719366 2.369871118 1.99107442 2.344628248 2.972923501 2.567326969 2.470504313 2.399422249 2.458502381 2.41650821 2.605450999 1.992741885 2.245021164 2.08219118 2.064296853 2.88223329 2.715424109 2.683447647 2.395381848 2.045024477 1.750032081 2.542887916 2.163827802 2.689422886 2.118989107 1.952812664 2.75315038 2.476530774 1.980530751 2.319073471 2.671385841 2.213365128 2.462761565 2.817683904 1.779989665 2.341499039 2.527111107 2.257474506 2.066956851 2.987090665 3.01022829 2.482665326 2.768337391 2.979654151 2.747252466 2.813693287 2.576040781 1.95296958 2.560023762 2.506343564 2.831301923 1.974760433 2.179686276 2.171837243 2.149469719 2.447270236 2.644376256 3.415105439 2.123652839 2.488900492 1.750641744 2.375982513 2.28656989 3.300782001 2.88783429 2.157735387 2.474724004 2.370286579 3.370040749 2.41604546 3.065790423 1.839523412 3.085204199 2.57861113 2.957156142 2.792425564 2.236462629 2.384744946 3.316733756 1.233442363 1.86853589 2.346166131 2.621771844 2.261884508 1.992519632 2.695552881 3.268189882 2.979505467 3.329982095 2.77956512 2.502046491 2.751976222 2.375471756 2.647614799 2.419961149 2.315564832 2.11889492 2.323784815 2.650227416 2.861192249 1.817088934 2.739095107 3.221709834 2.575703655 1.787382838 2.830349528 2.293683461 2.084183973 2.298891105 2.973160033 2.319461518 2.311341944 2.598634217 2.206398655 1.959240804 2.31808952 2.357075144 2.183028284 2.327147496 1.624941441 2.501448865 2.004980638 1.791591127 2.017993665 1.568650686 2.109999562 2.153628246 2.220389501 2.324511555 2.707185105 2.12723886 2.026997383 2.861040465 2.508854364 2.513148346

ENSG00000150967.16 ABCB9 0.751812967 0.58059319 0.495349311 0.305792037 0.469935406 0.328813758 0.292081847 0.555302913 0.60128094 0.409417756 0.500748555 0.392209171 0.567272339 0.66192745 0.538995395 0.575972894 0.381190976 0.406412162 0.477644315 0.46809693 0.491477519 0.440306758 0.537067968 0.514825576 0.564430306 0.489812395 0.502051995 0.548366774 0.401848474 0.475265279 0.454871452 0.367918052 0.203567464 0.42036395 0.420653906 0.389068838 0.502516048 0.314163348 1.100601519 0.686129888 0.705789457 0.869638823 0.448789613 0.797008723 0.9875729 0.719654022 0.573114638 0.920113878 0.408004718 0.530236985 0.466834616 0.683574421 0.241204869 0.425569128 0.738035791 0.262924164 0.262926703 0.419995241 0.396860356 0.262725664 0.331798266 0.367626411 0.347817497 0.333782353 0.448289929 0.321756222 0.51431707 0.361887678 0.6048048 0.465789502 0.607214422 0.430065973 0.317265204 0.378487453 0.568843159 0.744008296 0.397363975 0.652860777 1.110599138 0.418510124 0.337829008 0.458441282 0.43861713 0.549941922 0.284623506 0.382619694 0.273562869 0.621617622 0.282882376 0.2901193 0.407006394 0.450454792 0.661508449 0.464398954 0.396588259 0.504067465 0.436549816 0.342379533 0.369489529 0.380166171 0.436998543 0.616633534 0.54325902 0.651957028 0.311110825 0.449992592 0.419818402 0.409550989 0.431702703 0.553831902 0.557225043 0.435793384 0.451376534 0.411373102 0.270719867 0.308124532 0.236976895 0.364962769 0.565348083 0.364047374 0.351335094 0.33688438 0.308453749 0.294138017 0.377758315 0.368802629 0.561954201 0.504207115 0.286513843 0.541814388 0.453359881 0.207144707 0.287370095 0.573628655 0.436361339 0.461656928 0.482617953 0.499246445 0.380046717 0.169261753 0.386224329 0.496438513 0.544663491 0.367215882 0.469018262 0.594807732 0.543087577 0.345026945 0.507225592 0.416034664 0.299264888 0.552603798 0.430638938 0.463674571 0.374507319 0.499408844 0.389293614 0.442140064 0.838387841 0.45776123 0.246612986 0.326239724 0.523230368 0.543422809 0.619187911 0.543376315 0.337286444 0.686594319 0.639457877 0.403672666 0.548176344 0.467127929 0.583042605 0.301971535 0.248826383 0.410110033 0.529092506 0.424327103 0.361778008 0.499257445 0.58628862 0.314374733 0.561163686 0.700794687 0.59312957 0.318309637 0.386624743 0.490619724 0.531760476 0.580586466 0.59313285 0.404814857 0.449060354 0.700600495 0.301487259 0.210185332 0.412674209 0.371366678 0.684800391 0.348221305 0.31280048 0.374525089 0.445405387 0.382132362 0.294737116 0.410115136 0.370216652 0.639287401 0.309337429 0.373019467 0.474076455 0.302714786 0.486164921 0.370027722 0.178858057 0.256623224 0.374102455 0.449351428 0.309450704 0.547869797 0.504138056 0.455543621 0.333421565 0.32243065 0.377116828 0.436755597 0.708323025 0.387249082 0.516023248 0.564946361 0.294247214 0.208870426 0.33855513 0.350925322 0.426175749 0.324494875 0.311959272 0.342928346 0.397798377 0.341684129 0.479445333 0.604241221 0.695994553 0.330785578 0.710376414 0.352868842 0.375738596 0.59018952 0.607993901 0.608395226 0.399827603 0.367292059 0.415099928 0.271537627 0.349098688 0.521665401 0.324337142 0.425436909 0.495285761 0.270231897 0.425165303 0.211029088 0.352445403 0.379314123 0.587212137 0.447004214 0.432604469 0.541568329 0.427332146 0.34770336 0.35886677 0.420209188 0.402338145 0.351114594 0.494720312 0.384922404 0.44248244 0.323599256 0.25676106 0.614274281 0.438243871 0.507656718 0.322751933 0.354684377 0.487543697 0.545380841 0.349474753 0.386021063 0.300613946 0.454993053 0.35340951 0.452760155 0.531882091 0.279420601 0.441025052 0.509701899 0.763875173 0.763408521 0.424216701 0.407316955 0.796737535 0.441001641 0.344685635 0.371268548 0.225519767 0.517864695 0.395427906 0.436555444 0.42896221 0.327368825 0.28985474 0.340889441 0.247228347 0.313505816 0.342786967 0.398637535 0.483883385 0.585903453 0.573272077 0.254971989 0.6032236 0.642623452 0.60183094 0.261695148 0.690329015 0.607745084 0.310756345 0.484317523 0.480697702 0.525935225 0.424540252 0.331257628 0.455090248 0.511825368 0.513291692 0.315506206 1.217289893 0.37663251 0.351341223 0.286190978 0.513183256 0.612691432 0.321808371 0.337303879 0.635247639 0.646691278 0.310022999 0.341092672 0.434911423 0.495770522 0.353856509 0.487330352 0.431966986 0.18936822 0.548403108 0.599204409 0.62426934 0.477887793 0.416347735 0.599800592 0.591273291 0.419570464 0.398854891 0.338959576 0.567990698 0.519592355 0.527117008 0.454754363 0.799868754 0.314947808 0.640478822 0.570218286 0.483986375 0.607750583 0.433104804 0.281187196 0.526117545 0.495202825 0.36006394 0.54844435 0.446126242 0.798667915 0.715677407 0.364395953 0.451913131 0.397248967 0.330171364 0.470340734 0.430382976 0.29639452 0.695766738 0.28237572 0.384765357 0.290739755 0.345592382 0.577179453 0.287770367 0.273499474 0.315933649 0.42187481 0.513697032 0.490264795 0.483558601 0.561509111 0.234545658 0.369913628 0.388230868 0.634682342 0.183947762 0.473265997 0.605638794 0.383676045 0.771307092 0.474080608 0.494568609 0.38974377 0.332446164 0.534346155 0.283936172 0.646136664 0.31163823 0.388089356 0.403121864 0.532620139 0.451252182 0.360809683 0.555387933 0.70322947 0.364882094 0.559139197 0.925588946 0.526178219 0.432308983 0.477947383 0.46636631 0.782910012 0.671773121 0.187221313 0.320984904 0.409548529 0.429770884 0.635141662 0.569819761 0.475889484 0.498984425 0.527479074 0.430778407 0.556522981 0.621784301 0.231169244 0.270733054 0.218529792 0.270932247 0.374073708 0.318276349 0.375052489 0.370844733 0.420153321 0.546251601 0.356376131 0.357644933 0.496518033 0.462151672 0.473963055 0.519342244 0.507164771 0.298826051 0.442441184 0.501733069 0.447819064 0.290774425 0.31156459 0.349395322 0.457765142 0.385829595 0.309845511 0.393884372 0.478292998 0.587269692 0.61674087 0.432706614 0.430146607 0.485455774 0.699862259 0.704379678 0.485326821 0.373529505 0.484712626 0.532241416 0.523656351 0.647216484 0.343585026 0.936408669 0.394845217 0.518243081 0.703213149 0.874561156 0.426112893 0.410187595 0.261714273 0.497457242 0.3784729 0.704107897 0.575463156 0.534573023 0.48908449 0.608250264 0.509115745 0.489070751 0.491106953 0.649316171 0.841210711 0.436464106 0.752028263 0.741088208 0.34425134 0.848256512 0.189566867 0.345889818 0.666067373 1.016377835 0.546208213 0.583297869 0.580423117 0.282655897 0.429958591 0.460482613 0.313634718 0.443918376 0.270654102 0.369223903 0.349129203 0.579378775 0.362744392 0.194569259 0.401092814 0.464365484 0.302268787 0.421929305 0.576099244 0.75443613 0.300964976 0.799699785 0.664616984 0.678901654 0.367033617 0.405498329 0.79766051

ENSG00000103222.17 ABCC1 4.085557293 3.635924817 4.355750086 3.875051319 4.062237527 3.866475132 2.446782243 4.462368517 3.85333896 4.03764375 3.950760191 3.798602974 3.607787583 3.432676683 4.000785296 3.957418105 2.855903187 2.938079251 3.841905088 3.440417671 4.268989713 3.690178027 3.752561693 3.520672424 3.993054562 3.317495704 3.649230959 2.732422442 3.501589636 3.102283203 3.749533459 3.386412871 3.460848268 3.480153869 3.427109123 3.614568804 3.650216833 3.819838177 2.66355768 3.142075219 2.841949945 2.923671756 4.120070146 3.165457108 2.919681205 3.326505659 3.794579556 2.525873401 3.422600437 3.691406843 3.580141074 4.012976301 3.891616707 3.250058211 4.303989852 3.890505338 2.986992623 3.405167446 4.315548704 3.843259396 3.19175179 3.62743543 3.226760921 3.77259571 4.063357869 5.359906732 3.77030022 3.562489285 3.606075683 3.849380893 4.237867729 5.065387858 4.254216511 4.363005677 4.22378167 4.776742577 4.366774984 4.324504562 3.361831165 4.209816929 4.127812709 4.440386861 4.167389522 3.470012712 4.546095733 4.305300689 3.821445415 4.017529488 3.934178481 4.560498406 3.963700685 4.195745784 3.557251449 4.115362628 4.117630259 4.373189185 4.305568351 4.606853412 4.064347802 4.174595868 4.132150539 4.276375072 4.137133169 4.138028457 3.862603172 4.763574234 4.122642733 3.812854611 4.032246024 4.7433217 3.54200316 4.288511458 4.097057997 3.766410094 4.003920049 4.027310294 3.620337504 4.195219237 3.409456963 3.556148011 4.141416971 3.873904801 4.336918099 4.428584176 4.070890005 4.26804827 4.627138516 3.891031294 4.269206021 4.35896096 5.442211185 3.74162411 4.481422452 3.935948771 5.178666494 3.719205082 4.488200742 3.479317921 3.994464716 2.92506955 4.45596088 3.832391319 4.49628775 4.143687332 3.89352219 4.247196142 4.278663459 4.037261174 5.191476297 4.285503851 4.206458062 4.00499742 4.245759694 3.872552882 3.895966451 4.70360356 4.388986889 3.768693262 4.774798716 4.317625084 3.902424653 3.460850246 3.752103749 3.754679659 3.925782929 3.294153397 3.633030125 4.21751981 4.23231823 3.036101922 3.429489178 4.403929228 3.854394335 4.004191174 4.487807003 4.318309992 3.623367395 3.859974148 4.002831635 3.883816947 3.904336055 4.475364323 4.205899507 4.499977451 3.475424579 3.427737461 3.996584191 3.934751948 4.624659899 4.054660279 2.961068549 3.956475717 4.002225572 4.634269557 3.708097395 3.378490057 3.153689715 3.09675316 3.106721073 3.495664291 3.277375233 3.871664755 3.598490238 3.528585241 3.139669655 3.681531588 3.63243843 3.08494592 3.101632275 3.923660416 3.90273492 3.201396629 3.740179089 3.319924247 3.468316238 2.503411111 3.79906782 3.28277362 2.915036622 3.587777223 4.434354125 3.759428752 3.651924688 3.480839801 3.440381266 3.301016008 3.220802242 4.56565243 3.951559535 3.491027795 3.711225785 3.650303237 3.938208731 4.250002981 3.935342483 3.092758688 3.589256788 3.735190347 3.256119545 3.928034758 3.597495805 4.144400133 3.634153276 3.285069108 3.858868222 3.365379425 4.042688547 3.682785972 3.834428684 4.188988604 3.883088872 4.000896096 4.593802635 4.340844713 4.115933001 4.534013466 4.434629547 4.37228877 3.889398707 4.240545799 3.791033355 4.088342245 4.196809425 4.60898937 4.572438047 4.179632723 3.99994825 3.895888068 3.968063281 4.186520286 3.347205369 3.777501336 3.288597626 3.599619496 4.438643926 4.134045327 3.885655462 4.511717773 3.611671951 3.83717473 3.925933893 3.846832828 4.582722283 4.010478761 3.959722411 4.699886947 3.749279642 4.402249947 5.739060761 4.13841462 3.645369263 5.142463849 3.269010724 3.605945981 3.470722341 3.557910242 3.342906187 3.508678947 3.391005155 2.957120232 3.265203259 3.723417809 3.746457267 4.33001498 4.910917768 3.963367815 4.012837397 2.981990655 3.995519856 3.678444393 2.852767425 3.195183168 3.722665988 4.384448576 3.430063361 3.489753332 2.903204085 2.725158606 3.07415979 2.923698254 3.238963738 3.523881583 3.589588534 4.408878308 3.650913504 4.474945929 4.305678744 3.314114762 4.849417977 4.013895183 3.768413887 4.344554528 4.013467049 3.982713928 3.912514537 4.935076294 4.174098411 3.427090745 4.580868976 3.630143244 4.130838673 3.877819075 3.763419309 3.855687927 3.765171671 3.929387408 4.290596256 3.594634182 3.644299767 4.188201555 3.431871428 3.963915465 3.347927612 4.202087369 3.551901653 3.521541324 3.72177777 3.183644287 3.794744744 4.146087064 4.234644997 4.048576783 3.760896788 4.056041603 2.649586736 3.455517789 3.575123291 3.717440039 3.344609329 3.808384172 3.72790676 4.229141272 3.842950764 3.852891821 2.671221036 2.600778059 3.917184197 3.520146621 4.263877171 3.153161237 3.477969078 4.419934157 4.136656547 3.588849264 4.203340519 3.871696195 3.977058927 4.592019618 3.641267281 4.079471203 4.084383869 3.304524081 5.062334195 3.402536366 3.556918841 4.146735867 3.702650991 4.543671883 3.925502726 4.096100922 3.505399533 3.834466608 3.979815284 4.232713668 2.812351385 2.891876453 3.374620168 3.474630394 3.385898484 3.623442762 4.382019924 3.283253978 3.696956712 3.998658262 3.902575992 3.606633309 3.374490341 4.398440826 3.162489314 3.993335977 3.835068473 3.493284135 3.59652168 4.020753163 3.138087501 4.507862136 4.042575856 4.353835335 3.707324747 3.893015275 4.959281309 3.851120128 3.93700282 4.836390466 4.277592474 3.512175744 4.30426206 3.466244182 3.881515783 3.838487382 3.920241924 4.319112552 3.359746493 3.781255901 3.809586965 4.370621495 5.198852711 3.247271644 3.219411775 3.963696055 3.750931241 4.182793067 3.244206313 4.235160181 3.370274506 4.078272323 3.920568123 4.41432589 3.69057584 4.548628991 4.342808908 4.593468663 3.972440528 4.690448567 3.606431057 4.470694963 4.100077312 4.525748417 3.372079293 4.709703011 3.798373877 4.276424366 3.595473486 3.833984355 3.256897923 3.699344183 4.068865983 4.175931212 3.63436774 3.793842412 3.774902877 3.888563548 3.625899699 4.464203866 4.225551701 4.189653114 3.823400085 5.373982118 4.235026011 3.26104805 3.674007965 3.402478155 3.189151446 4.229930672 4.208821692 3.495612007 4.356255955 4.071813459 4.419542748 4.060375405 4.820470767 3.547953097 4.512477092 4.461341772 3.151430564 3.538345327 3.818881442 3.853398554 4.060147964 4.099663759 3.551004096 4.452263763 4.339551903 4.946063444 3.859162071 3.684781835 3.967687854 3.631097983 4.211279726 3.654846002 4.434666084 4.293131022 3.920371545 3.279381826 3.537220361 4.160882394 3.776080089 3.684610629 4.195361864 5.142012492 3.703307734 3.535727747 3.683535705 3.284568215 3.53274435 3.343043319 4.734046865 3.410815364 3.165488711 3.413896297 3.38124223 3.669699833 2.963548836 3.857855942 3.157678855 5.040206852 3.135534407 4.602668982

ENSG00000124574.13 ABCC10 2.490540798 2.346785239 2.133094413 2.131143085 2.267303617 1.546073151 1.167236713 2.016344406 2.218663655 2.086055037 2.437771813 1.723935647 1.79238666 1.807827458 1.750771414 2.0504724 1.732746183 1.589647978 2.243901789 1.821940455 1.927601646 2.06464147 2.21162696 1.933058405 2.301718507 1.76871799 2.226104799 1.844133792 1.699232856 1.783456522 2.032128614 2.086296914 1.407463423 1.710885542 2.213984293 1.809106584 2.010499575 1.637725276 1.893744937 1.903454188 1.942868351 1.889712995 1.924792126 2.232009843 1.908049659 1.887272883 2.04783609 2.087759648 1.859145411 2.285525755 2.289485397 2.617851218 1.902618307 1.698734329 2.034567886 1.761066243 1.513530166 1.601575447 1.91747072 1.983174727 1.628919696 1.95266771 2.224165542 1.786656164 1.984530477 1.843558797 1.758162949 1.344372105 2.325776505 1.708457866 2.201142564 2.128765322 2.031428075 2.292608158 1.693275097 1.915740479 1.701801653 2.271087486 2.14053884 1.834888541 1.857869948 1.857820883 1.777305961 2.197588763 1.628784218 2.027293992 2.025843167 2.116332895 1.620514228 1.934268929 1.957761538 1.656322732 1.638418662 2.253087115 1.91786317 1.708569159 1.818581173 1.613836708 1.984852285 1.758988815 1.633111534 2.261254181 2.206337205 2.28859583 1.770287745 1.693873911 2.065036581 1.9608224 1.953772706 2.098083215 1.860323955 2.632119611 1.721259308 2.04360037 1.861278679 1.484645646 1.206300554 1.908269638 2.213126461 1.645072617 1.854603052 1.580109957 1.426347769 1.774417648 1.695262152 1.867244898 2.035201905 1.674119296 1.668473119 2.109743772 1.836933553 1.66058502 2.060482125 1.532179004 1.922472906 1.683495724 1.903821349 1.952408174 1.719502065 1.340660561 2.019698555 2.062599896 2.133719339 1.796381727 1.907855129 2.449615099 2.180032455 1.979877519 2.025920717 1.995361238 1.751728735 1.617527761 2.236045222 1.969223865 1.802127766 2.225112286 2.130721091 1.831112729 1.748946057 2.170881296 1.795723523 1.744783209 2.29023152 2.308560223 2.570997237 1.023125236 2.163454243 2.597139264 2.617568795 1.776938259 1.549353648 1.634611418 1.499729476 1.786910673 1.786109306 1.65085614 2.246833464 2.065882223 1.816681832 2.407000203 2.377868646 2.364126952 2.570150684 1.921510413 2.058476638 2.004849063 2.243307907 2.398310431 1.804913945 2.17363414 2.07919363 2.149783403 2.02116153 1.521793373 1.81412396 1.209407063 1.806116777 0.976980823 2.702985265 1.477482389 1.589651068 1.649334436 1.643956057 1.068685098 1.962912262 1.888767511 1.308265809 1.183110362 1.781117666 1.979573394 2.043096283 1.626065939 1.980629325 1.491718083 1.062071891 1.125062488 1.894929166 1.644465329 1.810562938 1.809811325 1.265462208 2.005086571 1.637320101 1.345153843 1.634848708 1.387861534 1.543424722 1.678555509 1.918150329 1.488823714 1.714448049 1.095208103 1.461987734 1.827727141 1.750083929 1.3506115 1.353929372 1.798487276 1.681813166 1.17044445 1.428489599 1.456101047 1.851055925 2.00362319 2.342205083 1.38229754 1.814162214 2.127390364 1.773137412 2.618082747 1.859440671 1.842842439 2.035816688 1.790753188 1.624827257 1.585677764 1.733537667 1.971051424 1.918496129 1.75240012 1.815823822 2.125166317 1.976084507 1.750510878 2.113150761 1.749174938 1.822464833 2.300569003 1.783732107 2.153180959 1.895916951 1.715276223 1.710215297 1.900702623 1.996756536 1.834756349 2.218587799 1.690032245 1.556135511 1.588516554 2.075015598 1.889467805 1.646637403 1.97282323 2.387895139 1.992572433 2.083671444 1.610184518 1.317303572 2.150152895 1.622973343 1.803262557 1.848072332 1.286025309 1.49341915 1.624349213 0.950851094 1.607234057 1.701691698 1.673286401 1.744510437 1.778594671 1.599322788 1.706899209 1.538130853 1.76206018 2.065959878 2.313870394 2.315151868 1.742235157 1.334552664 1.730572933 1.615286003 1.796073078 1.574568132 1.526915077 1.750777055 1.413139459 1.559148366 1.038708304 1.220049644 1.807104037 1.696308926 1.829522029 2.04956351 2.347120927 1.788357243 2.276853036 2.003717863 2.089758021 1.847414263 2.041316779 1.91856896 2.221022732 2.006242976 1.996821847 1.876396883 1.721360543 1.570852939 2.026595887 1.894999726 1.75749852 1.808057996 1.313844605 1.40216417 2.421513512 1.762586798 1.773278792 1.999298168 1.955606091 1.924510493 2.238124624 2.205664024 1.619944477 2.004505712 1.634465713 1.026153121 1.868903406 1.355359395 1.419064684 2.099542133 2.092455244 2.083920423 1.762987646 1.391988197 1.596794797 2.032946448 1.720445035 1.95622558 1.155330019 1.464573106 1.836417862 1.962919471 2.078169541 1.251587594 1.357897002 1.64722974 1.935516515 2.006265419 2.028437028 2.364090046 1.973026278 1.921547386 2.003930539 2.150202908 2.105551825 1.690971297 1.995899635 1.909402966 1.715982196 2.043587911 1.993135484 1.651777622 1.612098858 2.345885341 1.791072376 1.816312485 1.95573891 1.640316232 2.031914607 2.105081705 1.801108101 2.271157222 2.196933995 1.369995612 1.534524233 1.588364248 1.737101897 1.38716503 1.194999795 1.401914886 1.61861512 1.463927645 2.091271914 2.144024812 1.744084464 1.998197211 2.063783969 1.704825752 1.972339592 2.094948851 1.572278052 1.840450858 2.064774904 1.813928228 2.104308583 2.09198106 2.350383233 2.337984443 1.998036144 2.093091882 1.890845113 1.984763527 1.973874758 1.50016451 1.707790549 1.705150105 1.542233676 1.884462993 2.116473076 1.921013795 1.277380725 1.818900202 2.06603479 1.700584385 1.776921757 2.060649276 1.720530684 2.335332433 1.523938132 1.762022002 1.623902297 1.73893983 1.759881104 1.961882957 1.786056237 2.121229618 1.69975758 2.302091289 1.823040304 1.821009001 2.173470916 2.141502795 1.957315991 2.200824955 1.623313414 1.939913474 1.800641887 1.861754009 1.942776606 1.640755077 1.516333669 1.534250564 1.966113068 1.699958338 1.519295224 1.951492103 2.014475987 2.680008016 2.301336784 2.10533276 2.176524599 1.958413623 1.890388939 1.576473641 2.073928039 2.229669818 2.00085615 1.968995074 1.927800746 2.531187174 1.707294912 1.891096824 1.366405491 1.847671669 1.890529212 2.91156457 1.924015325 2.150854828 1.854580819 1.356632551 1.874467684 2.024140574 1.677793871 2.0841492 1.855989328 1.834183604 2.138012443 1.854059217 2.15223922 2.355621721 2.132986643 1.891773303 1.760799806 1.978958648 1.775822796 1.870965654 2.434698523 2.35654992 1.391552283 1.825197714 2.265486567 2.209199909 2.249848106 1.703486714 1.481965864 2.102683709 1.667086496 1.799939898 1.632502495 2.027343044 1.698118038 1.178824034 1.861113047 1.507361811 1.204309411 1.529378666 1.697147035 2.299886108 2.542749193 2.088889781 1.846160494 2.263533818 2.395106604 2.070638456 1.997508958 1.492039053 1.957575427

ENSG00000121270.14 ABCC11 2.258522085 0.338688929 3.915827622 0.110497857 1.972176333 0.288213114 0.11848676 0.274310198 0.474654328 0.531729593 0.61266167 0.212982474 0.117270217 0.137877924 0.110897575 0.88265012 0.51724486 0.131335137 0.202802319 0.523776646 0.09449366 0.409704516 0.219567097 0.236055701 0.704859874 0.395165028 0.936257032 0.4452439 1.18867415 0.647247131 0.248689835 1.04087981 0.013647283 0.416247461 1.822669051 0.177905711 1.627383877 0.058624076 0.034997956 0.018548567 0.016046884 0.050569847 2.908812468 0.021252693 0.012883763 0.107756574 0.438094444 0.026708967 0.045453481 0.205117096 0.678430499 2.06837797 0.096497395 0.519994951 0.252616562 0.222112602 1.126084552 3.684950934 1.855644427 0.323389046 1.014058873 0.308233279 0.298501535 0.066733937 0.119743532 0.240992015 0.066138198 0.076448598 0.134261818 0.690691925 0.557076751 0.237955885 0.828073205 0.554351986 1.189272894 0.114990311 0.319808858 0.336627404 0.253317876 0.255118022 2.3534175 0.066460032 0.380909981 0.584674656 0.282789151 0.09210272 0.1087353 0.182600354 1.260540956 0.672974829 0.765966368 0.077080384 0.231533052 1.08307626 0.218669448 0.858811252 0.724637453 0.143039391 0.103296966 0.073831763 0.065815129 0.227812106 0.602363061 0.480226437 0.146585505 0.21196378 3.78996868 1.323839271 0.246886702 0.214816471 0.80282005 0.161168235 0.351640067 4.574447578 1.073574969 0.666245432 2.758909128 2.492388327 2.813318059 2.373098504 0.248457854 3.275450641 0.04032311 0.317690259 0.520986654 0.249258854 0.367754082 1.694641925 0.293696017 0.353826319 0.074431731 0.199128314 0.380014856 0.280089599 0.416659522 0.424596803 0.075855307 0.68835116 0.127169015 0.115763165 1.015721639 0.102087408 0.179833675 0.243474152 2.503569843 0.205756377 0.258966116 0.108852261 0.375479547 0.116820634 0.2300848 0.579154226 0.27354968 1.050736722 0.118658811 1.253825658 0.303941523 3.372554703 0.242271269 0.257879973 0.321827452 1.059597448 0.975228903 1.475272518 1.705590139 0.102158657 1.9718952 0.81665993 1.514798688 3.595800054 0.083502665 0.114172972 1.643475816 0.273677846 0.355637646 0.401574428 1.841905971 1.141903772 0.134009444 2.94323412 1.49682702 1.10433721 0.574635452 0.258307125 4.952864201 0.260791728 0.114062355 0.289610295 1.06483146 0.732629825 0.19461784 1.263511572 0.101198288 0.062275776 0.100718802 0.309514184 1.033660467 4.481611687 0.068081866 0.36503249 0.038094621 0.249441688 0.366259352 0.032613609 0.620452137 0.847079704 0.078280385 0.09092044 0.418699832 0.298463141 0.267365005 0.836908145 0.110903085 0.13971928 0.026623791 0.043747574 0.392514437 0.070130015 2.803563149 0.260548545 0.082737129 1.852580946 0.887987906 0.249628432 0.12512816 1.143637341 0.297086021 0.19698799 0.347939513 1.774271034 2.816776634 1.272211312 0.605385871 0.499100299 0.430869185 1.579482 1.016700573 1.049026331 0.457768924 0.035739705 3.761602679 0.13181663 2.140650292 0.089342162 0.180108032 0.04190016 2.851460041 1.353002187 1.469937583 0.900012297 1.023636814 0.287513068 0.260738298 0.169488288 2.680546572 1.757342465 0.17862007 0.773041482 0.203137383 2.863044174 0.38049916 0.505120645 0.076641872 0.187587821 1.838085176 3.555702085 0.052119115 0.478483627 0.101884586 0.487449765 1.346793895 0.586053922 0.383269029 2.664310428 1.237726892 0.130507851 0.482252352 0.708891209 0.285951917 0.386119649 0.865624142 0.106847258 0.858962716 0.255637563 0.154868292 0.169644004 0.305851359 1.978856134 0.179908008 0.851089403 0.952298741 2.472976152 1.586549769 0.021926477 0.941308297 0.31671275 0.150344416 0.078903611 0.877332633 0.373575535 1.72916995 0.403002675 0.484758082 0.106208715 4.03944133 0.110918067 0.214547113 1.11414515 2.933517874 0.513225312 0.611900168 0.724172029 0.152294901 0.229187227 0.641528534 0.071446845 0.681692486 0.696146103 1.945686808 0.057089427 0.098693592 0.073005285 0.376522889 0.121526945 0.265145655 0.74080522 0.173785287 0.499313963 1.082555041 0.376422496 0.729842431 0.365378655 0.440064428 1.466223261 0.523767904 0.110742926 0.180342693 1.731956037 0.487884587 0.053589406 1.098559159 0.239407377 0.160894306 0.259122772 0.564545254 2.258785958 0.436386193 0.165599156 0.262663097 0.13590861 3.641117643 0.073136356 2.618802303 1.005771204 0.371476194 1.019430235 2.340084618 0.145277821 0.257343809 0.264189888 2.568598608 0.784810976 0.277262116 0.073854796 0.769301666 0.06333549 0.6557994 0.296950896 3.138243966 0.121563535 5.946859776 0.122907739 0.217908983 0.408353885 0.347528612 0.861066614 1.287299044 0.098429831 0.238506512 0.754602672 0.307722755 4.122106166 0.142009632 0.164252974 1.499447902 5.451272314 0.050287395 0.272124614 3.488032638 0.369579273 0.158287184 4.825497987 0.078163775 0.239641777 0.90727318 0.248066251 1.295546532 0.018148129 1.581047055 1.683383106 0.667637408 0.671089873 0.456604962 0.397110291 0.512278693 0.183483105 5.635657202 2.867706035 0.876766656 0.098310775 0.166287366 0.089006716 0.184017146 0.167863759 1.656123252 0.669264967 0.183747277 0.190042189 2.388867277 1.893088468 0.177676145 0.685429504 0.554378039 3.816179991 3.19105818 0.227296791 0.070137769 0.132284732 0.747399543 1.159429651 0.201386184 0.363015759 0.228428793 0.66858939 0.209999387 1.501141719 0.608409445 0.035629237 0.056422113 0.259065077 0.262916134 0.862561889 0.100737533 3.224509477 0.121800081 0.165384554 0.184541719 1.156321089 1.203747231 0.434572091 0.251940564 0.039612468 6.367662264 0.107261382 0.922594619 0.020857862 0.087612568 0.404881833 0.164307977 5.082954972 0.254716207 0.165382179 1.445128037 0.690049242 0.786736083 0.97067709 0.434368037 0.529780492 0.500892772 0.315116215 0.029992965 2.86295524 0.043483576 0.223746199 0.675580849 0.170658281 4.75678174 0.760796237 0.465383802 1.803454164 0.741641334 0.059764466 3.331081242 3.480701816 0.920915252 3.828351578 1.383512269 0.529502678 3.509505197 1.156220764 1.306247464 1.244322379 0.108313336 0.132578107 0.321243966 1.008743568 0.154353073 0.539984916 0.365978857 0.371936517 3.113595575 1.142141053 1.054152525 0.033643612 1.926814982 0.156702469 3.19936559 0.124093045 0.356081304 0.229227405 0.220080781 0.11396029 0.289593086 0.112769027 0.159606219 1.893844692 1.314127615 0.590622661 4.757103494 0.16140331 0.145579171 0.442934591 1.282351011 0.905030784 0.096331388 1.480730974 0.059084907 6.006259578 0.499264231 1.709887781 0.14535152 1.348326304 1.209864058 0.113497034 0.060796505 0.012153936 0.461748615 0.953136003 1.574686232 1.779620415 0.228491393 0.171206808 0.349957736 0.317793127 0.009724309 0.224402863 3.616370613 0.078359601

ENSG00000023839.9 ABCC2 0.14428844 0.297966331 0.224060963 0.06525498 0.125973067 0.066249604 0.02373061 0.077056029 0.1428516 0.079926477 0.138055493 0.076605184 0.077971956 0.105384912 0.045229251 0.123234027 0.10597715 0.109327778 0.158578214 0.072349643 0.099384188 0.137954645 0.09770739 0.086460011 0.191250323 0.063050144 0.16001856 0.07340304 0.080402109 0.034329513 0.088468243 0.201686144 0.043184652 0.030096494 0.127940899 0.074250414 0.082109732 0.052045613 0.181636856 0.114684 0.168037117 0.225374654 0.120103003 0.092389796 0.112822769 0.096424429 0.086089271 0.096954827 0.120118315 0.134467654 0.257996092 0.185476856 0.179976894 0.178623095 0.14411981 0.139688019 0.353462028 0.326488135 0.140779366 0.142028314 0.179508034 0.091063116 0.144983013 0.10323883 0.121722011 0.169961071 0.101265999 0.044929117 0.297891156 0.0728265 0.154527449 0.210344853 0.213379239 0.131062728 0.094451115 0.136181269 0.529684649 0.070576779 0.065114595 0.195074056 0.095447785 0.212049145 0.147818323 0.117463001 0.10020626 0.227225827 0.086450804 0.15623741 0.112164613 0.216542785 0.099707204 0.110700507 0.109951054 0.163203224 0.122333698 0.116103327 0.132313092 0.091907859 0.1234975 0.063178851 0.104249414 0.109024113 0.197404304 0.095848394 0.160861525 0.081101219 0.13855026 0.069381563 0.158647833 0.090567744 0.099145892 0.20849686 0.132044919 0.118953073 0.068429958 0.049506126 0.049644371 0.09697632 0.098275044 0.04171454 0.1375744 0.117686219 0.033562847 0.134838996 0.119527763 0.128801823 0.057456473 0.072353097 0.082529765 0.105717522 0.106227719 0.038013754 0.180199435 0.074624335 0.046644144 0.043021042 0.051874017 0.072170247 0.159085894 0.013945089 0.086518471 0.145117085 0.167956037 0.120786718 0.11590381 0.253946456 0.176294113 0.320429975 0.147954418 0.110365755 0.133394986 0.470708662 0.072143928 0.128783637 0.083786185 0.137082369 0.160876098 0.150620598 0.301593332 0.204762223 0.192471027 0.296719944 0.109336489 0.138907356 0.297050606 0.031753278 0.43515469 0.263004542 0.200675483 0.024263363 0.082179165 0.203971606 0.239828625 0.226940319 0.027319897 0.085579938 0.099012392 0.108273191 0.150466393 0.141476094 0.212283605 0.162685528 0.194427531 0.296247026 0.219851202 0.096893895 0.101838168 0.154831341 0.190357225 0.149662449 0.088858479 0.143479917 0.070605431 0.118319266 0.552557866 0.056224098 0.061734752 0.073376246 0.202856032 0.050127871 0.082841153 0.077047252 0.058165284 0.106732067 0.083377528 0.06787972 0.042238448 0.007504663 0.056949759 0.080567526 0.038215149 0.074981121 0.110526199 0.084430617 0.02067698 0.030654577 0.089189986 0.034396522 0.065136041 0.034172849 0.063319042 0.088330538 0.065169997 0.079886631 0.040204912 0.087918966 0.079925226 0.057840132 0.078743608 0.048875988 0.051286456 0.123867014 0.045669859 0.158600404 0.068401652 0.047497558 0.035078851 0.067901215 0.076236582 0.015216939 0.050863433 0.293134614 0.163414272 0.08663585 0.109706702 0.024503333 0.109017077 0.123836145 0.190476163 0.298614076 0.347131085 0.079367659 0.068927838 0.111715894 0.131124916 0.07481457 0.097465179 0.105674231 0.107333032 0.056816902 0.117766906 0.113107218 0.081221848 0.174670968 0.526188464 0.091165562 0.104123314 0.147894358 0.080964935 0.165626768 0.099435599 0.091104451 0.123279645 0.084202099 0.108327467 0.220457876 0.204185525 0.271017273 0.106754756 0.145795531 0.170798549 0.100400213 0.355792499 0.139525462 0.291826307 0.076827482 0.1885697 0.178637621 0.18118307 0.263074009 0.054944118 0.426192957 0.042386135 0.118303439 0.112376446 0.022694748 0.101044728 0.124657637 0.032752885 0.079693892 0.108715436 0.04705521 0.172738253 0.128350246 0.37710855 0.117464429 0.150102798 0.077723516 0.113425319 0.100887636 0.01715764 0.056208918 0.186579409 0.23812002 0.147238205 0.102695935 0.027869747 0.061337205 0.06840697 0.089071303 0.01770367 0.066169513 0.04539214 0.124046078 0.140097277 0.179584323 0.084923473 0.199813573 0.10437362 0.153223451 0.14357 0.306079635 0.097189275 0.191925762 0.163852198 0.148927309 0.039537155 0.065957637 0.088801547 0.430780751 0.084493596 0.088617332 0.134838038 0.089376777 0.179592648 0.126287618 0.068063643 0.09421946 0.246772421 0.141515469 0.330094277 0.127579019 0.170808362 0.143116484 0.118164452 0.172106536 0.017369419 0.130174605 0.175970917 0.043934166 0.135876396 0.08219881 0.054792756 0.158362717 0.020056453 0.073372848 0.114768327 0.085851792 0.037259098 0.203835815 0.360542264 0.176401509 0.149434477 0.120569476 0.013572817 0.019157252 0.242102947 0.051187334 0.12045818 0.078962361 0.299572236 0.132216357 0.190029008 0.121834135 0.129645654 0.168365493 0.097749417 0.178843293 0.14809786 0.13485384 0.08616423 0.027781397 0.332155358 0.157741597 0.511318743 0.151125878 0.069781558 0.234620576 0.148599431 0.181150017 0.153088787 0.03683524 0.153566427 0.073310741 0.027450114 0.046840489 0.135058216 0.189257389 0.029168874 0.091646551 0.016974642 0.089948247 0.083122938 0.086828555 0.235157061 0.132013374 0.037478103 0.342378982 0.069375665 0.29531914 0.097386397 0.092445739 0.459754858 0.132178534 0.108783311 0.120298306 0.219204225 0.246179509 0.068615273 0.261772093 0.196053632 0.247092744 0.265694795 0.25141381 0.16368691 0.153467765 0.124493711 0.15241414 0.155921872 0.116942852 0.192841145 0.174773966 0.073200277 0.114387822 0.060905591 0.284155205 0.122644796 0.265003922 0.138834764 0.071330102 0.136148449 0.147795812 0.112354033 0.123967059 0.114368621 0.066816884 0.107110774 0.235800837 0.094902698 0.535598249 0.248236836 0.202680323 0.199100021 0.263138431 0.302708219 0.153547587 0.184142728 0.158955561 0.163425163 0.130400158 0.092124761 0.167631041 0.064520037 0.096361174 0.12190079 0.123284915 0.14049919 0.163195667 0.355596183 0.204627311 0.137824918 0.069249355 0.236667994 0.138985498 0.144962714 0.049170246 0.31813535 0.174889102 0.120464011 0.156107227 0.140800147 0.124609854 0.387559166 0.157286225 0.142559829 0.19967926 0.062998185 0.179754005 0.067578233 0.062562979 0.260842787 0.114429145 0.165316822 0.185417331 0.142376578 0.176591629 0.226966973 0.251202546 0.167290356 0.214322762 0.307815025 0.311105757 0.177642964 0.126395935 0.3149812 0.075142064 0.072754515 0.320730299 0.195558404 0.18601366 0.060241854 0.268312485 0.13971852 0.229184828 0.084345687 0.132016985 0.245237144 0.071266475 0.125707436 0.08083668 0.086260812 0.045848708 0.363713523 0.103003085 0.091441234 0.069256837 0.109607247 0.093796697 0.148839873 0.153968519 0.10408698 0.133212038 0.127702602 0.135021123 0.055629378 0.093469161 0.07161739 0.116331496

ENSG00000108846.14 ABCC3 1.542843326 2.021405649 1.18244012 1.860897426 1.950425027 0.37766007 0.928316432 1.273871878 1.565736365 1.135822296 1.436901367 0.658000315 1.413637592 1.587173153 1.34074244 0.739718203 1.125667968 0.228966658 1.349247725 1.326055262 2.648479397 1.85948271 1.289978055 1.306615327 2.315674884 1.039487192 0.822602647 1.472702675 1.178328769 0.881995247 1.718323641 1.811446813 0.575920173 1.736441197 1.411546748 1.265219904 1.066485609 1.156576661 3.186957712 3.599680174 2.959174766 3.87837492 1.745644338 3.382086048 3.103804012 1.428097089 1.836945418 3.621441635 1.721538679 1.66547437 3.202507077 2.46510264 0.871475685 0.141582397 1.375364373 0.540960364 0.115770188 0.152157535 1.980736135 0.206453321 0.298432954 0.368957893 1.338318657 1.890176906 1.823164373 1.332648249 0.973296701 0.662888394 1.990352594 1.507338156 0.9929858 1.539125445 0.782015231 0.869173013 0.864258921 0.452658617 0.797430696 1.147752618 0.417756542 1.197834545 1.447369336 0.781099874 0.78965486 1.305260868 0.274780382 1.118659993 1.873896904 1.728694551 0.242971365 0.512889657 1.324332665 1.257405752 1.150950394 1.668477566 1.158264632 1.513365307 2.225464879 0.797702219 1.475058244 1.461663859 2.01079466 1.607239235 1.713729937 1.559513928 0.505383182 0.720228128 1.234506941 1.015743984 1.43048048 0.953040672 0.867028055 0.693871344 1.017055044 1.213930109 1.110447742 1.434778053 0.328041756 0.601945029 0.846154407 1.348613933 1.252886907 0.977949122 1.134975709 1.73772237 1.354381133 1.03479521 1.413201943 0.470472404 1.387119772 1.635469488 0.39966026 0.23501233 0.548751951 0.506789872 1.823606595 0.519102514 0.625214508 0.727297685 1.104255128 0.882584881 0.93885098 2.261135624 1.322285993 0.921287615 0.663324812 0.785687924 1.279746925 0.738811233 1.229907066 0.823412344 0.682030387 0.313177774 1.469017799 0.538386764 1.310511945 1.470947692 1.877618504 0.809481475 1.098368963 1.926299301 1.365153095 0.763429155 1.425924538 1.929281852 2.776371415 0.571096651 3.244056344 2.708320313 1.96951747 0.905293848 0.575790986 0.748920273 0.488363911 0.254177713 0.325563448 0.917028335 1.473795658 1.142139674 0.93167501 1.868642032 2.133922678 1.502761108 2.227939684 1.874072842 0.775154292 0.451390484 1.443370545 2.114350943 0.652992206 2.052833298 0.917103755 1.410880331 1.702824803 1.136798443 0.192575246 0.215375105 1.348024726 0.516033949 2.536036913 0.753639487 0.695125272 0.784956992 1.885859277 0.195971033 1.148770393 1.461517698 0.373938337 0.573873718 1.638143727 1.248466593 1.755267704 0.857182211 1.647693253 0.329922469 0.497456415 1.047724331 0.664432631 1.09351737 0.306432963 0.670824189 0.106999563 1.472623117 0.392878344 0.691630496 0.759392728 0.393003518 1.816884166 1.36882675 1.742549603 0.442811669 0.58287778 0.828627084 0.86735058 0.73261231 1.152500527 0.118008909 0.463539513 0.823131137 0.279899566 1.284828092 1.772242421 0.993407077 1.451876346 0.511228103 1.919697098 0.42278535 0.285781947 0.428092605 1.326397919 2.394642045 0.670569341 2.406386128 1.589608532 1.178235075 1.016688338 0.503003282 1.344058173 1.183708956 1.03780355 1.455708823 0.723574803 1.013137529 1.361524211 0.690381153 1.921867462 0.774008872 1.228225197 2.292219435 1.364867106 1.117932018 1.010674524 0.64873698 0.170724126 1.531904167 1.698039352 0.800769052 1.253944937 0.795712226 0.318073561 1.426386787 0.720686132 1.103839328 2.100998143 0.548299085 1.965869917 1.404847417 1.0416252 0.383491396 0.777318333 1.984031841 0.274641524 1.007304505 0.583240976 0.807476125 0.600321053 0.609959654 0.659367057 0.941018184 1.122834897 1.218582056 1.512441094 1.033240195 1.794690393 0.578664323 0.549902247 2.628030113 1.461120345 3.760190719 1.122376188 0.853261024 0.208022028 1.446524293 1.951286293 1.703953266 0.372937632 1.147139875 1.567266783 1.118492475 0.84109438 0.561527433 1.049491405 1.764223097 1.072390978 1.721577182 1.685594339 2.591663718 0.869588353 0.58565946 1.123599423 1.021757365 0.499884413 1.735960234 1.151810873 2.217262507 0.985903298 0.623978901 2.191750247 1.405773679 0.076078768 1.12922681 1.489947321 1.28375321 0.744766736 0.073587583 0.49602235 2.356838009 1.094343807 1.395686465 0.337252934 1.550797395 0.222377751 1.846231044 0.429522927 0.807960546 0.958003654 0.652995516 0.507274333 1.214116321 0.858276635 0.577428708 1.844696896 0.871673657 0.730531516 0.194686902 0.557656853 0.252957105 1.14040747 1.337985315 0.653716335 0.070649972 0.703664227 0.913922259 0.831908794 2.01556065 0.530293786 1.064738173 0.602764441 1.474727853 2.132117229 1.528152948 1.575931521 1.636238176 2.094940018 0.29991806 1.817078395 1.884058403 1.250538783 1.983724502 0.606200513 1.395579587 2.460543614 0.198671385 0.096161963 0.571127121 0.778684377 1.678655071 0.493215138 0.297424132 1.930962311 2.036685949 1.956492126 0.446270764 2.002282702 1.534453075 0.64644879 1.852849526 0.021574403 0.491113639 0.44372327 0.489422843 1.04175588 0.990038179 0.990415837 1.209070878 1.791154287 1.185444824 1.208023861 0.194951894 1.100484033 1.342837471 1.448055167 0.592624858 0.431651845 1.861132623 0.821429654 1.241954997 1.606179587 2.397686032 1.539584215 0.509405749 1.331376324 1.026234831 1.884257656 2.168454419 1.092915606 1.197123979 0.636914261 0.233721134 1.131375055 1.3405634 1.66731094 0.175069664 1.577915189 1.421982669 1.11568311 1.533826999 1.569366575 1.334113964 2.057331537 0.180750836 0.608130308 0.9710994 0.864808438 0.449531438 0.45071074 1.518843998 0.295823647 0.044590594 3.506981003 0.797515846 0.988583387 1.721001402 1.525240593 0.348855428 1.672657125 0.741231813 1.005692514 0.809728638 0.483145921 0.762273946 0.516616568 1.275673507 1.678305083 1.953637809 0.977613161 0.689266654 1.406851684 1.472010522 3.334430348 2.01162585 2.271469102 1.133690099 0.970513567 0.931186608 0.318075034 0.887137536 0.956552776 0.543020343 1.262348007 1.678845584 1.954092804 0.45162039 0.209990515 1.157650537 0.688440512 0.720498504 0.628841851 1.936100353 2.448615255 1.204833464 0.267314343 1.38142418 0.868183068 0.080373432 0.765313026 2.186246437 1.012285834 1.533710629 0.858680914 1.503345535 2.355455271 0.547071787 1.687159637 0.539795035 0.821693675 0.323385372 0.410733577 0.218746166 1.567154002 0.372490839 0.31376303 2.317186737 1.827562153 1.906089039 0.098447691 0.429044394 2.139575498 0.211830686 1.719039727 0.071683661 0.902402024 1.146663226 0.275271583 1.786134429 0.332833254 1.119736838 1.267859533 0.804700863 1.330336539 2.053862697 1.860330656 0.847220233 1.220468 2.409899073 0.450968097 0.461669855 0.232477359 0.635061499

ENSG00000125257.12 ABCC4 4.974700612 4.32688049 5.199729057 6.453638007 5.631743196 3.237822399 5.303790954 5.442967617 4.313163478 6.260193389 5.57003389 6.402722771 4.176404574 3.435276905 5.228289608 4.09331302 5.463899309 2.723134105 4.882342072 5.59039679 4.74637786 5.854739745 5.126438122 5.351342848 4.726003163 4.916974921 3.266653485 2.881554158 4.882219069 3.023112677 4.452663966 4.707150725 5.546652029 6.418260555 3.955732864 4.502963064 4.494476679 6.061277876 1.108249525 2.227407444 1.007536264 1.308772772 5.198904683 1.798977713 0.978796267 3.824931584 5.1599209 1.259286078 5.11978642 5.39747277 4.021229282 4.664074775 5.926044986 5.889870638 5.956708584 6.383402889 6.130330201 8.252218786 7.117138829 7.591238286 6.318340076 6.425347603 3.232706291 5.921033835 6.77770492 7.497150966 7.15455095 6.572649225 4.276670393 6.313410851 6.773136027 6.962323912 7.827731878 7.47984759 5.540281954 6.462753051 8.002124778 5.420963102 4.309915187 7.484593065 5.959304863 7.258698001 7.619036153 5.444276885 7.983245757 7.903883561 6.519677468 6.837753973 7.988955566 4.321928574 5.93011527 8.08901827 5.431841836 6.507460397 4.257399858 6.486887906 6.12643452 6.372299013 7.110249492 6.31278621 6.249392317 6.584439858 6.216896273 5.168495812 7.176476289 7.69951303 5.219579185 4.939608101 7.055259921 6.992777368 6.36911172 8.109131976 6.80728692 4.529051418 6.012876772 6.487941624 7.60796479 7.672275304 6.450669984 5.343070898 6.061681801 6.874452052 7.426427438 7.484485049 6.305717983 7.051507666 7.094420567 8.183271792 7.483056745 7.143605497 7.934051664 7.38233601 7.111963644 6.692428901 6.390598185 4.166449536 6.970464054 6.044903454 7.327417104 4.264987406 6.907530825 5.734140273 7.412658317 7.451152012 7.659519602 5.811564072 5.35939833 7.785872978 7.68262715 6.006418049 7.61961579 8.271107698 5.592046565 7.103886 6.602193367 6.752945866 6.799190443 6.945924003 8.198853621 7.972906522 5.744601686 7.391052612 6.027280386 5.591254531 4.431940705 5.982470015 6.328167863 4.920571719 6.591617595 5.828074903 5.789316889 7.816749031 4.783513212 6.613872788 8.429228482 6.774014051 5.654274874 6.066826143 5.860597496 4.412667924 6.618415093 5.493996032 4.984846455 5.940717902 5.964131079 6.89084661 5.931135737 4.903603712 7.408325798 5.130511849 2.337525805 6.629178949 6.196393429 5.96208199 6.738036632 7.539257021 3.503855891 6.542753831 3.398922707 4.256161642 6.173718675 6.905497375 5.196557313 5.175012511 6.121728086 5.129837457 6.4052129 3.705362353 5.644952119 5.966979277 4.97303274 6.165583387 6.447786854 5.202870703 4.276925078 6.320573954 5.212309637 5.816169744 6.921870409 5.049484427 6.567188746 4.303926408 7.226683259 6.391582638 6.671351183 5.312926808 4.037771656 6.623638388 5.359180239 7.207110178 6.261296619 4.290725128 6.711463372 6.82471572 6.965638943 5.736292226 6.426618124 6.123755777 5.334644303 6.631429539 6.002954726 4.75666479 6.087771522 6.942041545 3.643465583 5.31926642 7.284855544 7.011671611 6.14851577 3.951614069 7.125037392 5.602626577 7.454501909 7.459689781 7.589258498 6.756167283 6.303652929 7.413627729 6.906401048 4.817372159 6.643123843 6.787285498 7.630191406 7.367947933 6.669873139 7.84475611 6.737518502 5.33886725 5.817055176 6.953673464 5.693895866 5.863084293 4.00639955 6.953400468 7.227294599 6.592574115 6.88658939 6.346236135 6.790941849 7.576564085 7.617504568 6.591835973 6.21710266 7.438657055 6.66655041 8.000087321 6.143082709 6.23404288 7.246885026 5.694027454 6.646345979 6.179060285 6.717210616 6.466063201 6.017568363 5.475468983 5.299622071 5.887946993 5.897009532 3.129967795 3.7921676 5.969280006 6.146341565 7.874881412 7.925313503 5.310307883 6.155580634 2.443299269 5.115108628 6.232057248 2.108358257 4.159133096 6.902950968 7.690646343 7.292320385 6.016706993 4.376335624 4.523002532 3.807486311 4.713146427 4.136551092 5.09328921 5.680764419 6.74608167 5.41441827 6.879463127 6.73039776 6.593787533 6.079202417 5.502536458 6.291360928 6.947782499 6.729754963 5.229762716 6.200067166 7.101869865 3.97016409 4.699169063 6.304261305 3.440930727 5.053401041 7.371552228 4.762831787 7.111196519 6.122405092 4.523288494 7.047920834 5.051537965 6.373179044 7.351042872 6.130136262 6.019519778 5.866680372 7.460849544 6.39634793 4.748130413 7.414529674 5.492106409 7.266231662 6.8313463 5.511050397 7.844213034 3.951147093 7.223153513 6.176723889 6.837126168 6.717441756 5.486144392 5.265565504 5.550108769 6.943155057 5.890640159 6.242019212 6.826724903 5.439637509 4.443942747 6.534645255 4.998257492 6.602887571 5.30615229 5.204467138 5.442971007 4.521684873 6.794539286 4.884522878 7.374077051 7.113810117 6.310174647 3.893789052 5.864020012 5.474456357 6.792156742 6.92028347 5.633095664 5.575184569 6.423708304 7.512658854 7.229830246 6.603412931 6.740672124 6.04495924 4.911152718 5.351388191 7.058293054 4.362229921 4.874181686 6.387893907 7.734366303 4.687964695 5.198856354 4.642657354 4.768867733 5.413060646 6.479104143 6.191299913 4.626890378 4.193881877 7.463158662 6.176051554 7.051028606 5.898452718 6.353718484 6.194910499 7.033596973 7.112251751 7.015723089 6.656692355 4.809393489 5.942683673 7.34968664 5.433546111 4.882065653 4.549839589 4.183681088 7.471037242 6.419662424 5.708224312 6.374101245 3.236632101 5.341866063 6.539124842 7.319121031 5.651626181 6.373079165 4.958652879 5.334986604 7.859200053 5.900104444 4.459676944 5.664821529 6.031853706 6.115616903 7.386510982 7.697117613 6.723525176 6.334657505 7.052343188 7.284649984 4.196259583 7.180019644 6.393018994 6.944556702 6.24746396 7.107535157 5.754954401 6.637781853 5.604280082 7.503758398 6.932298577 6.412307752 7.034974939 6.751929233 4.986698355 4.55245336 6.252833821 6.503064996 6.949445842 7.130107527 3.278114978 4.526985636 6.372372995 6.255404874 6.164672607 4.923361411 5.239653496 7.230059439 5.126606542 8.515411485 6.524790327 4.370080113 4.931307942 6.592718583 3.135907073 7.56435982 6.588927178 6.539012085 5.973275528 6.543338296 5.350132448 5.77705908 8.37912127 5.652341002 7.051792146 5.889392743 4.763105501 5.95204123 7.82407902 6.561716421 6.319843026 6.049696173 4.429887112 4.22259738 5.116095212 7.651796753 5.844104738 7.07849171 5.987177844 7.385250692 7.430446324 6.377265438 5.983964749 5.71266811 5.645347051 4.219984442 6.725176489 6.668206149 5.23740977 6.379241392 7.57252034 6.11954972 4.357297209 4.541232503 8.739303688 4.55227652 5.820101964 4.519384652 5.945066288 5.829858757 4.789958828 4.420123757 5.154100878 5.057361644 3.429676204 5.132873133 3.306318352 7.767786369 4.41788363 5.74194944

ENSG00000114770.15 ABCC5 1.989762603 2.952045143 2.460161772 1.344202542 1.847991019 1.505861625 1.443770898 2.123753901 1.773803063 1.918117416 2.296367298 1.698066612 1.64291173 1.901304109 1.363355417 1.774041643 1.902527609 1.556999629 2.658164338 2.086296683 1.793861276 2.240238926 1.928701548 1.806765661 2.420617696 1.710491335 3.04792538 2.453043164 1.690162831 1.345949322 1.556791753 2.681196672 1.415313289 1.341900096 2.766406577 1.410863038 1.854339473 1.649493705 2.389674838 1.947492075 2.13742761 2.207252732 1.267116461 2.1283493 2.097958544 1.224910442 1.618714491 2.223210982 1.837045751 1.988609048 4.061485153 2.65972193 1.645835064 2.428121405 3.589889013 1.549687146 2.546646519 2.649260629 2.914689189 2.281008749 1.73509977 1.970514514 2.554723196 1.415999333 1.883397561 1.84901329 1.988459437 1.202798182 2.706253489 2.103655632 2.033842487 2.154039199 1.934576617 2.367544883 2.84934322 2.093259812 3.389759773 2.719707811 2.68432373 2.058773745 1.82245227 2.323965393 2.150147264 2.182562206 1.597200071 1.629475289 1.264349856 2.157183765 1.592805364 2.252100634 2.181350065 2.04461541 2.588754424 2.058914673 1.721107818 2.091057901 2.311161657 1.598897519 1.928565822 1.86310747 1.559730522 2.056702705 1.795447783 2.027556043 1.696808357 2.287715068 2.242932986 2.344163757 1.971231598 1.963694185 2.448934905 2.157238094 1.76619964 3.604209316 1.927770677 1.644475371 1.217460765 2.676027295 2.594025496 1.838321243 2.090477302 1.891376587 2.243647866 2.280979947 2.486200764 1.555079985 1.655411201 1.813610291 1.835722319 1.801087568 2.009626728 1.26169669 1.776217011 1.520280763 1.547667016 1.618975 1.13709194 1.760588266 1.842539403 1.94416324 2.13880033 1.929187365 2.665981406 1.523024896 2.141043674 2.569587422 2.847480001 2.369809155 1.82859673 1.696781744 1.927728632 2.639489431 1.829765147 1.481377052 1.427095585 2.072507647 2.026058962 3.392869885 2.755614009 2.784732085 2.742372525 2.212374528 1.92985025 2.103015528 2.719840774 1.843990086 2.087216267 3.467503731 2.412164677 2.094369719 1.845958264 2.508879029 2.685373038 1.567743366 2.524664245 2.205086234 1.936847351 1.673953756 1.751451552 2.344960946 2.596313695 2.175570359 2.672981472 2.921370081 2.730076005 1.827501983 1.806387134 2.07544466 2.353421763 2.184614478 1.533974547 1.825176024 1.952389205 2.982797238 2.153786394 2.251324863 1.949815355 1.961569911 1.837624548 1.320236678 1.252543627 1.585251475 1.284568158 2.369194454 1.657690635 2.275001719 0.849450223 1.435371099 1.658398603 1.282318338 1.70472672 1.389311126 1.772811496 1.843360018 1.162489104 0.952951334 1.723060301 1.16563386 2.337368188 1.414675678 1.082034419 1.913475986 1.266482256 1.602416502 1.538329031 1.263742009 1.861268646 1.901489909 1.58023642 2.039527908 1.838233825 1.619445936 1.164244225 1.593909882 1.791154256 1.309978342 3.991813867 1.612272945 1.264287564 0.676078183 3.597899821 2.517424549 2.481000099 1.523946872 2.003853918 0.964743498 1.483233004 1.671329894 1.975904584 2.471624414 2.500906798 1.841403346 1.681569465 1.592091958 1.966514248 1.667097076 2.621911795 1.257405447 1.665260127 1.552019489 1.612952144 3.436864617 1.826890016 1.689576995 3.634846281 2.146430751 1.209021132 2.157817032 1.511060672 2.052462975 2.211572173 1.557780135 1.84147896 1.570323855 1.835283952 1.857203394 1.878228483 2.659744811 1.2650683 2.117385017 1.907321864 1.66954756 2.685996721 1.748242497 2.603434443 2.208873157 2.209473666 2.566938784 2.358015059 2.304881395 2.265391824 4.423537676 2.84227769 2.562920281 1.991935038 1.268713842 1.269990419 1.651989736 1.716237181 1.689677526 2.354738209 1.516974797 2.305685799 1.49742016 2.990736364 1.903631141 2.056419223 3.549996 2.972188035 2.40499899 1.036587799 1.402213872 2.51647963 2.183589269 2.276674261 1.551200082 1.508779117 1.863404912 2.241673871 1.372076968 1.684776598 1.698690008 1.864370508 1.842117164 2.016473141 2.411406591 1.265210785 2.41526385 1.752897688 2.039622019 1.698363516 2.255734775 2.117202179 2.488611684 3.052203178 1.455323123 2.572617205 1.964326838 2.438551887 2.983771239 2.112190575 2.749306752 2.064425629 1.627879529 1.796243202 2.798957997 1.6629027 1.810856596 2.472512129 2.210952341 2.370151239 2.099369975 2.098537676 1.745445534 2.057696437 3.343382115 2.229004128 1.675273683 1.871699812 2.717412624 2.70805525 2.2127006 2.414192058 1.776005272 0.933069986 1.322788448 2.186585032 1.703207313 1.430742121 0.877156498 3.219430884 2.178214363 2.603220321 2.29587895 1.583866813 1.743916482 2.694311426 1.829427694 1.924421618 1.99300732 2.985987193 2.927581397 2.273807049 1.7719622 1.953415691 2.389351678 2.253048647 2.140634516 3.354315301 2.230249428 3.248261673 2.327683878 2.635813138 1.966567509 2.650600236 2.059588553 2.02345462 2.846891337 2.955565313 2.684037426 2.336122743 1.742192202 2.178716848 1.977429122 1.916432208 1.277422185 1.125426186 2.91639795 1.536141267 2.044721984 1.651912897 1.708839692 1.914651136 1.841091664 2.701674769 2.603211671 2.118722427 2.557174792 2.303880204 2.747594692 1.664458043 1.564573669 3.237350732 2.447312607 2.042283065 2.302461499 2.251942084 2.789113646 2.648724173 2.14117909 3.03655405 2.852182656 2.181197332 2.413967102 2.054502073 1.966707294 2.714158587 1.11706249 1.893004092 2.067851247 2.223742801 2.286132336 2.29322688 2.567101961 2.190857526 2.275803471 2.351976913 2.661248751 2.14201306 1.658513716 1.55615795 1.444500841 2.794528831 1.516390974 1.96254813 1.899747703 1.294534491 0.988971262 2.267464059 3.084730641 1.389995744 2.397011878 2.274750412 2.894964658 2.582011623 2.67425253 1.634002302 2.894745535 2.322088539 2.627268107 2.065575399 2.520392756 1.84103797 2.15824183 1.701859351 1.371925134 2.645021839 2.448970247 3.082891725 2.711473483 2.164086157 2.272040778 2.460270984 3.437762162 1.85875216 2.630715192 2.869184722 2.579841121 2.370726576 2.439094853 2.092080272 1.216150767 3.409529958 2.02596354 2.03223418 2.871599199 3.16377279 1.921916284 2.922411004 3.569295669 2.263977051 2.030958492 3.760246428 1.923698625 2.579688596 2.093498782 2.606959029 2.445944631 1.880013585 2.656809477 2.74419158 2.38188638 1.876029049 2.390729844 2.459793502 2.676117001 2.826901505 2.769253659 2.956638637 1.769431174 2.397142158 2.558634761 2.91674753 2.389017944 2.126630459 2.493859513 2.386086876 2.793198181 2.566921731 2.325527471 1.78460765 1.848079057 2.511674381 1.859922433 2.943824171 1.432034683 2.757681372 1.983568005 2.378437922 2.875880752 2.598106439 2.195816164 3.885106225 2.808301177 2.083302442 2.341802758 2.298644646 3.011308513

ENSG00000091262.13 ABCC6 0.995946063 0.491080984 0.54115845 0.227173998 0.696537251 0.458115223 0.548870352 0.700231693 0.812359293 2.033916283 1.219164545 1.999512295 0.720035301 0.71510149 1.414182428 0.784266432 0.968740391 0.250849254 0.867868702 0.747078944 1.934413043 1.007841761 1.650824671 0.996305324 0.671742364 0.670040897 0.674403059 0.302502785 0.326833096 0.500510621 1.817369393 1.784963574 0.358845195 0.661069559 0.356515082 0.799268683 0.46244234 0.369041693 0.288337572 0.181877313 0.408135491 0.389961499 0.662481912 0.339720395 0.287001083 0.486849391 1.91505899 0.097404434 0.870506058 0.917136059 0.363369261 1.903904907 0.794999498 0.182557893 0.205201241 0.363571349 0.211685088 1.253123366 0.457144646 1.406363205 1.402975989 0.185203893 0.49978582 0.36517862 0.408863672 0.482942226 0.50171693 0.127932382 1.270718917 0.619937823 0.377122199 1.708719091 0.311887115 0.321316949 0.215096707 0.409850929 0.34898333 0.677490741 0.680473989 0.510080906 0.501039995 0.90966727 0.414733914 0.414370682 0.508940999 0.499531774 0.18480472 0.471573779 0.268707133 0.25262482 2.446958559 0.291079756 0.301166083 0.560631346 0.788906394 0.196085798 0.330980766 1.234453759 1.367580297 0.687631301 0.461602856 0.563132809 0.357393736 1.097704374 0.174773313 0.682205507 0.378491142 0.987961629 0.406902524 0.674885659 0.276367767 0.628115559 0.803669901 0.179968992 1.959185937 0.292808276 0.726696554 0.358872103 0.401059203 0.249327981 0.324831721 0.2780484 0.512889589 0.508424972 0.454145658 0.313466247 0.441652269 0.59341902 0.336755536 0.655657525 0.613736099 0.263702257 1.533011676 0.389277861 0.581085035 0.15354382 1.449555854 0.333185133 0.333156481 0.871951437 0.786337063 0.609645006 0.751184407 0.892496826 0.444739504 0.360702986 0.681817098 0.302715647 0.662031859 2.054815863 0.255409488 0.305109957 0.618702954 1.425166271 0.678947473 0.546174245 0.52263264 0.294923267 0.379869493 0.687657079 0.390635397 0.2235832 0.963283142 1.598768887 0.52588671 0.511001406 1.316373407 0.754420977 1.136064408 0.643497731 0.531982202 0.567317638 0.227483663 0.291738061 1.777981 0.831592532 0.662441945 0.300741819 0.932791659 0.617915662 1.52347886 1.008054787 0.910869868 0.400524861 0.48730461 0.191152684 1.558676786 1.092552469 1.44843093 0.827304376 0.564489773 0.682464467 0.715493837 0.40505547 0.240771185 0.194980975 0.548967133 0.376338066 0.317358137 0.792963682 0.456131577 0.649443627 0.535475766 0.268468189 0.399260024 0.412491024 0.427767641 0.606373571 0.322863988 1.555493903 1.114275372 0.393678018 1.173761237 0.311270132 0.325272401 0.31263493 0.426942368 0.329541514 0.860695266 1.001381572 0.444878742 0.612801346 0.294750361 0.395247091 0.326731196 0.671310931 1.412780534 0.610866272 0.558318889 0.389716039 0.540188795 0.359081367 0.355511768 0.435248934 0.487703955 0.119989262 1.563883184 0.701113149 1.012429105 0.372997506 2.327498104 0.73993686 0.202626456 0.299405836 1.583346601 0.403224515 0.524679506 0.139932724 0.293657463 0.700564683 0.293882035 0.200635967 0.461897979 0.306922244 1.240053207 0.147579965 0.370213385 0.494896618 0.997734895 0.22105185 0.270212169 0.303028925 0.358321129 1.671379826 0.952918799 0.430096036 0.217939109 0.587450409 1.484631039 0.345557647 0.208792882 1.595522015 0.320241565 0.537503637 0.421720701 0.75480297 1.649602313 0.446070902 0.13090514 0.52442588 0.541451472 0.372636074 1.704168139 0.238401141 0.693656285 0.320771294 0.379048267 0.329831234 1.157376938 0.785832877 0.12169359 0.604184871 0.222319505 0.28012694 0.282712635 0.739221185 1.018919603 1.495228336 0.540372381 0.62369038 1.039058882 0.481648512 0.239880034 0.28648741 0.457433024 0.502106728 0.69397065 0.94352263 0.270417864 0.248149719 0.395756088 0.588735156 0.448097672 0.315784411 0.22571082 0.53858487 0.301038851 0.518112662 0.242463805 0.916140826 0.428782674 0.961996609 0.665240835 0.749780512 0.559376334 1.430555958 0.425562323 0.877556346 1.502544569 0.57098234 0.425238868 0.406620387 0.396153963 0.93245237 0.301756863 1.545799623 0.357746293 0.883326444 0.206979203 0.544331062 0.436506411 0.226194116 0.788215348 0.107888267 0.550399862 0.432076862 0.748800572 1.168595595 0.383146318 0.374268229 0.341214404 0.499157345 0.149178834 0.315408816 0.429356968 0.254705104 0.495107018 0.322472803 1.362658248 0.187508156 0.68434106 0.440591957 0.506589883 0.164559723 0.622526081 0.411184885 0.50363687 0.308838338 0.175578611 0.90751171 1.497675393 1.439843973 0.339356082 1.066443043 0.433689865 0.225254904 0.47775513 0.570025751 0.975357183 0.63263839 0.240517059 0.279599098 0.940277451 0.338196219 0.685300596 0.543925571 0.315894836 0.555252307 0.970262949 0.268837647 0.345524116 0.086552294 0.878551211 0.333183071 0.663998635 0.146631702 0.360028021 2.321293591 0.355603603 0.325253276 1.349650505 0.45465268 0.746355698 0.483627076 0.176147596 1.262820682 0.192390144 0.406322728 0.5801984 1.000238556 0.728588591 0.621373882 0.50351206 0.743685144 0.49655344 0.993728432 0.198552354 0.435848312 0.522662835 0.366319871 0.963454321 0.200778806 1.391646977 0.489858638 0.246753477 1.010717528 0.378968343 1.473014348 0.153732598 1.650608314 0.977190481 1.347060511 0.349425336 0.583350257 0.458224088 0.253613803 0.416153839 0.25787384 0.864261632 0.455505159 0.43378337 0.385674011 1.422297049 0.354928476 0.27099176 0.437343646 0.684716647 0.163832954 0.411055064 0.267490197 0.309354254 0.619733212 0.301433665 0.292273122 1.046570751 0.323356023 0.346880336 0.230209544 0.944813613 0.30997415 0.40019142 0.755248178 0.517293727 0.597295989 0.341785981 0.319494148 0.502305841 1.453925079 0.171825421 0.479789478 0.342314671 0.304149169 0.650586251 0.490911697 0.211004828 0.357078982 0.526885125 0.453501643 0.514888268 0.309858096 1.375510751 0.282387636 0.357071201 0.980193282 0.302307467 0.326987844 0.321506641 0.881029709 0.391440184 1.062609633 0.731620914 0.166315416 0.156504941 0.180087288 1.848387357 0.196938343 0.49257216 0.433564432 0.409964939 0.264193516 0.568151123 1.03903878 0.825827977 0.320488268 0.071315773 0.525949251 0.657831486 1.447829403 0.46634471 0.303782011 0.677289372 0.436218688 0.606110869 0.464544194 0.337789095 0.175660995 0.251735043 0.229630469 0.36848187 0.257314712 0.448373139 0.91469468 0.354747818 0.546333154 0.75112473 0.312427375 0.678993711 0.681189139 0.358649534 0.693927249 0.186350281 0.197134473 0.153795402 0.647604769 0.164254945 0.935248565 0.947071147 0.248898579 0.314880177 0.539378759 0.272824295 0.110698794 0.303548688 0.252040317 0.108071357 0.622078134 1.370762513 0.377357769

ENSG00000006071.10 ABCC8 0.381872165 0.75045808 0.721438512 0.785110205 0.246272477 0.347034954 0.639391513 1.313210403 0.608866621 1.053072085 0.610756077 1.060501258 1.026758294 1.443862045 1.531623215 1.383196731 0.688742599 0.498614045 0.846339923 0.800435789 1.757413271 0.467326343 0.811925935 1.031393829 1.217268998 1.066681273 0.47872876 0.28059639 0.434354304 0.400334392 1.327332557 1.078979476 2.046703176 1.280945824 0.481157802 0.586674643 0.504947679 0.230320837 1.953850283 1.200284867 2.345631813 3.234573196 0.306387322 2.163252101 3.322432639 1.090940842 0.701862761 1.837755082 0.542179674 0.769720186 0.657986292 0.799111123 1.271734241 0.110226383 0.520719562 1.586029402 0.31041578 0.109541169 0.104122637 0.291386039 0.096455549 1.41110284 1.176910246 0.365387584 0.210709802 1.235631912 1.014046464 0.909960101 0.342253595 2.630018039 0.250042041 1.755647114 0.391518658 0.313182447 0.046748224 0.874336428 0.326369839 1.966606675 0.286076487 0.136364518 0.185496892 0.550634052 1.159791777 0.650379407 0.366005239 0.64916784 0.643233627 0.207568605 0.089113763 0.440804186 0.962942537 1.573465851 0.430693438 0.724209542 0.678135913 0.526371123 2.172354867 2.163236191 1.042470104 1.1673591 1.321431157 0.41359967 0.463072775 0.596390459 0.531604136 0.568846538 0.38672454 0.632947376 0.57344279 0.251030488 0.417664975 0.044861166 0.887991743 0.62831028 0.693224854 2.440839369 0.554599095 0.935197186 0.226963282 2.531880156 1.183341753 0.915071958 0.125506972 0.80025901 1.425069164 1.419172786 0.331845584 0.560645143 0.574761456 0.147116416 0.68954805 0.248784672 1.139369532 1.210467405 1.579847827 0.1702302 0.779476055 0.541936933 0.72152529 0.760091621 1.357081511 0.603038595 0.841938017 0.831623597 0.25373219 0.751730227 1.981316453 0.665745193 1.543227889 0.477460359 0.274184907 0.894022878 0.718890241 0.275133991 0.875902174 0.61068457 0.331018627 0.295910678 1.190711524 0.117126315 0.230696422 0.288604591 1.275677812 0.876850766 0.462161811 0.235517704 0.511501426 0.945713263 1.318008992 0.270040536 1.018945357 0.382442383 0.312511187 0.13309346 0.540518723 0.343835434 0.460034929 0.343375264 1.991211711 0.296867836 0.843987993 0.623430723 1.25068465 0.281251027 0.748121951 0.285119163 1.384806223 0.445009812 0.051928751 1.278385932 0.982798565 0.255636025 1.891880856 0.198794619 0.159973851 0.219621734 0.191865369 0.043820223 0.227858087 0.95164648 1.924905911 0.415597262 0.395307602 0.775210351 0.285475144 2.161773513 0.3285907 0.435577332 0.306286011 1.960003324 1.664313678 0.874874004 1.183568573 0.598029121 2.134594367 1.876655845 1.485484268 0.918085484 0.370944115 0.766239837 0.319057641 0.519017542 0.210635617 0.377551194 1.509535439 0.741251698 0.405690181 0.485287593 0.996893668 0.724334754 0.382010924 0.252812929 0.353342508 1.215705294 0.420090884 0.147581314 0.390848658 1.605844809 0.457450596 1.508451357 0.663996216 0.758943097 1.690424944 0.311827786 0.829426094 0.896061587 0.097833886 0.479462144 0.48511143 0.111035943 0.332712427 2.746950355 1.209633275 1.381464347 0.790509708 1.523671418 0.628751132 0.880243562 1.477744257 3.127197371 0.218764519 1.172057874 0.55243366 0.720590067 1.075536879 0.38942922 0.299845436 0.788183391 1.086077027 1.426777593 0.187680413 0.172948873 0.418538607 0.345009321 1.136632938 1.739979058 0.861288106 0.619516613 0.842238753 0.601466081 0.42362843 0.758566212 0.2498541 0.872756489 0.694558006 0.186185216 0.529978933 0.167871522 0.317382766 0.573893869 1.066639761 0.372232396 0.130173872 1.036272851 0.63430834 1.838905485 0.28860981 0.330582514 0.749215609 0.508907391 0.459912681 1.072786678 0.211488281 1.904337291 0.287213707 0.516842854 1.163521064 0.119191453 0.562682655 0.660249821 0.692428781 0.469849782 0.139538267 1.176807796 0.246057121 0.24098308 1.26390113 0.207156375 1.264944531 0.722701849 0.16976441 1.174783317 1.061697572 1.273607875 0.94730593 0.261217007 1.672115536 1.276765842 0.621123972 1.588704685 0.696241085 0.143427921 1.39367629 1.120780772 0.888039642 0.802404233 1.551166757 0.558878419 0.247812856 0.69075132 1.902194902 0.09156894 0.718048872 0.211298968 0.732250316 0.418404527 1.102621144 0.523374879 0.49348499 0.606479309 0.299623604 1.115875675 0.43575549 0.301175256 1.212057448 0.221466906 0.307523345 2.124177221 0.427145769 0.049279864 1.258419684 0.178585207 0.818524986 1.203229189 0.919415239 1.445877557 0.383524048 1.332847567 0.067789899 0.564031701 0.025867594 0.764779173 1.295710058 0.743235008 0.187837093 0.516185778 0.276518946 0.350180044 0.957902284 1.527487865 0.530187254 0.371117519 1.988833338 1.421858431 0.999702363 0.451756004 0.567080053 0.451541831 0.253018616 0.315596891 1.415732109 0.377179961 0.900828036 0.641669628 0.744241158 3.086379952 0.312743558 0.178780731 0.095695371 1.44633123 0.3542167 1.132155714 0.285852116 0.431125922 0.040203415 0.415499449 0.268085348 0.721764087 1.378487846 0.239895632 0.457978676 0.415970741 0.178597724 1.557182661 0.599746822 0.444601511 1.839767276 0.348949123 0.575977339 0.4228026 0.971003661 0.28802125 0.407899464 0.222352879 0.481353145 0.98509499 0.818228668 0.204222965 1.441903274 0.235682549 0.243697085 0.281849983 1.178411758 0.532412677 0.365851035 0.270704219 0.278207341 0.60939732 1.371395736 0.493209231 1.275075667 0.380081247 0.749332716 0.922387609 0.808023325 0.314335071 0.418151322 0.221163508 0.328164448 1.376356475 2.52527774 1.718608674 0.633246881 0.483121176 0.203778334 2.181605435 0.694268313 0.18716091 0.539275321 0.839584848 1.221724982 0.569412086 1.763544402 0.084347208 0.244690843 0.491963732 0.211779194 0.340515827 0.739311994 1.150713203 0.865392825 0.139558401 0.854670263 0.356356246 0.216772183 1.454797433 0.718528665 0.366887031 0.462950864 1.230040893 1.506239554 0.498675146 0.918222567 0.566003863 0.08507386 0.271664259 0.457606082 0.593435462 0.912815454 0.309558395 1.065363699 0.306831486 0.051182225 1.160010869 0.313498426 0.222118763 0.126621498 0.245830977 2.25647235 1.741178308 0.043815184 1.164089496 0.689339167 0.291809147 1.322585603 0.494472138 0.18771907 0.458127373 0.385024719 0.309518011 0.952657844 1.495176649 0.193967945 0.813678891 0.436226228 0.45925239 0.093747147 0.293476433 0.504610317 0.160536466 1.92917053 0.245943164 0.224407502 0.985331431 0.291678327 0.488668809 1.280909975 0.465651276 0.066091762 0.419216554 0.625025341 1.865842698 0.074131618 0.583998395 0.398908438 0.272605801 0.042039077 0.454788744 0.467841325 0.181433552 0.14541291 0.744977118 0.397159815 0.056154169 0.071218773 0.69189987 0.116670733 0.059514183

ENSG00000069431.9 ABCC9 1.17071017 1.399321838 1.391795501 1.315921236 1.548249185 2.573145006 1.095171167 2.152661084 2.630980799 1.567623059 1.383844037 0.716339971 2.689055258 2.129242086 2.253407306 2.685079758 1.3240679 1.797271596 1.897179878 1.779898076 2.573259775 1.562561506 2.295851829 1.61458781 2.560414832 1.483621647 1.201111552 1.170036396 0.905100431 1.659619584 1.272779634 0.566247872 0.826394652 1.409471999 1.07697191 1.679430234 1.089638787 1.657529478 0.433472308 1.393226867 1.32025281 0.95606899 1.326185191 2.081927855 0.795102128 2.009759015 1.519911545 0.54822233 0.999255029 1.145001763 1.414951878 1.066696521 0.619287494 0.854776932 0.566103645 0.949019357 0.293286577 0.126654305 0.895569597 0.723212578 0.966108002 0.699503475 1.592543972 0.532634464 1.625803014 1.51185667 0.664703299 0.28979951 1.458285641 0.532957019 1.376319721 0.993972638 0.917443479 0.685767034 0.903696714 1.041358741 0.567966891 0.964604796 0.786573958 1.431736653 1.842803829 1.276322831 1.009463676 0.787892218 0.713463923 0.891618574 1.396943996 1.71712864 1.083149759 0.67089731 1.778253741 0.711526518 0.893000558 1.70826999 0.629342737 0.732424219 2.33639664 0.87427979 1.484220451 2.161252672 1.200744572 0.99271896 1.276715994 1.402908691 2.026368126 1.019279077 1.258509615 1.188441852 1.604197253 1.597841704 1.072944034 1.318232431 1.498726528 1.418026163 1.902400205 1.924781243 1.559423661 1.531320053 0.958246246 0.777320986 1.526451495 1.844370502 1.485735614 2.133712534 1.965766323 1.230836454 1.567836335 1.10228357 1.858992216 1.427115985 0.862086915 1.024592506 0.657302399 0.3670066 0.526048627 1.369838021 0.611609688 0.423546872 0.720859791 0.143043439 1.742856769 1.782242633 2.014156829 1.921284986 1.579331097 1.348676529 1.720918446 0.841089865 2.051199564 1.397267392 1.436178894 0.9809854 2.042249324 2.188951988 1.022668247 1.768417167 0.999907347 1.294647145 0.930704627 0.635449697 0.610753383 1.60797435 0.922500935 1.132070233 1.409507305 0.101459015 1.097387621 1.619768027 1.239032545 0.492829106 0.087483198 0.334809408 0.966832934 0.682741383 0.571249087 0.485604085 0.577537911 1.498576942 0.374499317 1.720343181 0.968055447 0.773972236 1.445144114 0.718471814 1.073338579 1.194599229 0.924454636 1.387885087 0.317244486 1.514939635 1.24142176 0.725807312 1.82003733 1.737947746 0.109807547 0.366402904 0.352871997 0.079894882 0.984766743 1.19050549 0.273543677 0.723694135 0.584471907 1.502790504 0.514121942 0.736340462 0.510407978 0.796657349 1.251253733 0.839102963 1.308410357 0.265228222 1.204671 1.492530908 0.536996663 1.071436178 1.641989172 1.938588114 0.56092172 1.178528467 0.432905789 1.692536198 0.430771022 1.147033543 0.457217483 1.308297582 0.483217816 1.589229797 1.119365686 0.895478199 0.466516261 0.532097872 0.646753876 1.181521334 1.162803803 0.784688823 0.836466105 0.960992899 1.861557195 0.884545615 0.533626559 0.233700465 0.218606369 1.423215516 1.818336585 0.384416615 0.596066029 0.385882111 0.863492652 1.714754539 0.302288336 2.061584379 0.743176327 1.796090685 0.686037933 0.927258627 0.796053409 0.6037436 0.801249255 1.345469082 1.938287372 1.343362827 1.443612094 0.846972504 3.1481396 1.144168549 0.891169025 1.572911021 0.720895635 1.686157673 1.015410668 1.684449687 0.264745245 2.09066848 0.931180661 0.617803491 0.797351718 1.310471474 0.366868169 0.688923414 0.490670231 0.757509191 3.229644502 0.577164921 0.770822776 0.577057669 0.688961808 1.786350438 2.438378551 1.391553465 0.606431183 0.692920165 0.48677467 0.807339345 0.269955822 0.24716646 0.109929287 0.440000254 0.265484805 0.585435115 0.437930246 0.208618453 1.14591745 0.369525238 0.362519347 1.252164666 1.282116475 1.238262194 1.188410589 1.617938313 1.068142705 1.412847701 1.051431477 1.303823339 1.295958993 0.243152313 0.315913824 0.579689736 0.169811343 0.322585822 0.816643672 0.521417335 0.203646442 0.824056492 1.155702005 1.239947562 0.96155889 0.796506969 0.49507744 1.227039297 1.426974126 0.786435612 0.406123101 1.37616037 0.512570104 0.587486353 0.797877709 1.552034081 0.210140637 0.94020127 1.31997781 0.967124585 1.531210394 0.401852402 0.929176504 1.604587465 0.462397535 1.484103288 1.287434243 0.879980816 0.224465798 0.940930042 0.577630021 0.269225797 1.596983695 0.423839615 0.614419329 0.656022798 1.342358453 0.572820987 0.88432672 0.438792953 0.3126054 0.264938295 0.446619431 0.143245711 0.844138678 1.166931265 0.271425045 0.339505334 0.346519826 0.41271833 0.108833534 0.625400581 0.121257294 0.262277581 0.343566913 0.389511986 1.200298371 0.668481294 0.966951471 0.501956694 1.554908751 1.233074321 0.679189432 1.643880668 0.738371872 0.974522067 0.628876918 0.617907515 1.382472958 0.436312891 0.309933696 1.079840969 0.886171371 0.306288536 0.631603246 0.30437455 0.873817152 1.315299816 0.921175855 0.095982186 0.886777108 0.896014914 0.082290346 1.059643804 0.034154319 0.37253354 0.228513978 0.198649652 0.263277311 0.081231584 0.130127466 0.874625223 0.98833947 2.460315708 1.042524857 0.256854458 0.745449467 1.30208473 1.095071482 0.875249431 0.880421037 1.245446942 0.963233538 1.118648251 0.64240693 0.994729985 0.817156968 0.91183679 0.56500943 1.74662773 0.922060195 1.874639632 0.261581491 0.987156308 0.730298683 0.53808947 0.603723289 0.699462965 0.953404414 1.069589507 1.369535408 1.191075741 1.065319166 0.797774477 1.304560457 1.843638831 1.893235198 0.68101218 1.004009747 0.239416148 0.483097337 0.507324475 0.983697973 0.472998548 0.501682325 0.07831453 1.142814183 0.326665607 0.555753222 1.345408572 1.657999357 0.498656375 1.402542246 0.548489543 1.404905446 0.65262035 0.590071272 0.430459768 1.064471238 0.447824808 0.755059773 0.495863587 0.727261999 0.550574658 0.245130861 0.441717566 1.384013831 1.414127834 0.920594457 0.30371643 1.600428672 1.265497929 0.493717717 0.875213566 0.418526215 0.452241717 0.87262903 1.32991097 1.494154554 0.220649009 0.187115978 1.488815746 1.080370305 0.432618829 0.649631911 1.752574257 1.774215446 0.555667369 0.346165727 1.461741062 2.104541426 0.167438043 0.359504265 0.842931127 0.543327189 1.17999484 0.997436316 1.798991035 1.218219683 0.399049778 0.686827976 0.48345652 0.753037996 1.012519736 0.62096162 0.483058655 1.4514896 1.04265564 0.352485542 1.469786135 1.833320839 1.245575456 0.951550825 1.094960264 1.36989631 0.521879457 1.574919731 0.92857168 1.391445342 0.954748414 0.714526564 1.769559246 0.699359887 0.479698735 0.767151325 1.058765854 2.01229811 0.580396097 1.324033465 0.863063 0.936791611 1.410026383 0.471055069 0.302386553 1.052928659 0.558560552

ENSG00000101986.11 ABCD1 2.426890134 2.080339978 2.286892583 2.191026644 2.481323995 3.130036174 1.854432469 2.804719869 2.696764042 2.225916903 2.766797313 1.895022727 3.271865997 2.977158403 2.842659716 2.855971726 2.038294938 2.950647069 2.298745067 2.547374951 3.140232324 2.368847717 2.421227922 2.498589289 2.689129798 2.666062636 3.110358667 2.769766048 2.004862835 2.928636188 2.913950232 2.030491916 1.868521787 2.632306904 2.60192047 2.469890349 3.413199072 2.128726298 3.066240488 2.4890344 2.835019943 2.64299537 2.510196908 3.218126043 2.994087847 3.373861634 2.587157119 2.68204913 2.075159069 2.363066966 1.882682426 2.802945252 2.544396045 2.265232711 2.744156506 2.258642575 2.309148004 1.856889229 1.92117546 2.175237389 2.682515516 2.185604707 2.492115727 2.072437567 2.240385585 2.217464741 2.101611316 1.883914178 2.309464121 2.398695498 2.626891254 2.061388067 1.887880679 2.270477411 2.898616858 2.189445823 1.713265645 2.791018785 2.431737065 2.047482263 2.174801572 1.918122746 1.786244225 2.009878396 1.682413518 1.831347661 1.968705426 2.785272733 2.075114866 2.087634517 2.332945126 1.976499351 2.018189549 2.853511523 2.05411679 2.771814809 2.229931783 1.837524006 2.080817711 2.338045667 1.934604315 2.279429338 2.563309126 2.849814744 2.191253533 2.15937751 2.032473024 1.89603272 1.827777385 3.03510402 2.25938709 2.176795348 2.096318297 2.19114557 1.994837274 1.655041195 2.091972516 2.481279027 2.36576567 2.135782725 1.883835113 2.026462436 1.58634206 1.822556102 1.856374192 2.123150989 2.643865546 2.276539277 1.717946456 2.513291266 2.273268625 2.607047117 2.240238833 2.738847992 2.242600113 2.378977341 2.285971171 2.744177869 1.740953604 2.127620038 2.359433636 2.51636823 2.58968804 2.342997442 2.515068045 2.177181361 2.290764584 2.298335013 2.462672523 2.37905291 2.038818885 2.765781196 2.660444402 2.697721157 2.23444041 2.507170928 2.089958047 2.58062316 2.352657051 2.175476017 2.382280507 1.989282563 2.800422868 2.680005981 2.607061549 1.576353199 2.750722878 2.937802035 2.846133703 2.428858855 2.07067223 2.330367957 2.266011836 2.156329747 2.382312601 2.22050254 2.34749407 2.699406195 1.955988554 2.665581449 2.764754733 2.701560316 2.844764251 2.462666364 2.489072723 2.650899016 2.341125205 2.626620847 2.162193882 2.836967333 3.000238061 2.508832021 2.486846409 2.636799941 1.680913977 1.39957106 2.725683155 1.935734302 2.562052657 2.561558021 1.83742825 2.704601115 2.322684485 2.791185318 2.214291432 2.248281127 2.638079441 3.218461248 1.885134748 2.195596398 3.09439227 1.900410508 2.365578598 2.696939532 1.734280866 1.780107291 2.172087743 2.569895221 2.493141981 2.793288937 2.303368167 2.561555908 2.081138158 2.307898182 2.338684958 2.710349865 2.775702708 2.321966806 2.360832622 2.208852174 2.995217655 2.152910429 2.012242336 2.329788534 2.638890871 2.547634672 2.691436252 2.091079914 2.993554543 2.029674449 2.844229439 2.78025811 2.205050754 2.174258481 3.157025057 2.281748142 2.157176071 2.610463878 2.384698207 2.81165183 2.244255108 2.325745696 2.452960044 2.440895266 1.991570402 2.171487288 2.778466167 2.137025438 2.034772072 2.009739306 2.266453084 2.319288821 2.331050461 2.423481912 1.693214701 3.068789915 1.950388914 2.475664589 2.256895944 2.416373234 2.147175483 2.875764925 2.874068621 2.69951073 2.28633746 1.891517498 2.479946661 2.331215067 1.47954628 2.388793967 1.995069533 1.685037103 1.214176206 2.074618249 2.10453942 2.853698839 1.902522824 2.393765171 1.174920539 2.54616412 2.288728161 2.531717101 2.489015042 2.236193439 2.431960933 2.437342689 2.024181602 2.777234472 2.308971745 2.896592414 3.117739137 2.263739187 2.521663482 2.034799911 2.192431515 2.168894482 2.163978187 2.409455997 2.180614945 3.046644618 3.310951616 2.964876709 2.37706765 2.081127678 2.277301905 1.797643564 2.554266362 2.680230703 2.333181505 2.429478027 2.610674439 2.66228565 2.545013514 2.189021585 2.679194374 2.42382957 2.362517751 2.726534488 2.38635811 2.672390641 2.654068294 2.520857849 2.108984225 2.739478236 2.350475342 2.421054731 2.243067768 2.50495788 2.002058535 2.884199285 2.156554262 3.414000727 2.510021928 1.987817327 3.011678496 2.726127765 2.143977397 2.688354398 2.828478827 2.410860871 2.557033644 2.512650632 2.490922378 1.939752096 2.666199299 2.404901745 3.026931871 2.223073374 2.851528673 2.754051433 2.263754647 2.419394625 2.896288489 2.026647017 2.794046085 2.165565665 2.182715364 2.040643753 3.147535262 1.674997005 3.21363779 3.345765198 2.48550642 2.779893158 2.048965759 2.293722046 2.465742592 2.997301539 2.459891423 2.43758976 2.383879601 2.394540574 2.753425255 2.140242624 2.461409393 2.752373877 2.377155386 2.841500852 2.073729371 2.271379399 2.530150973 2.673899638 2.629100032 2.548556702 2.475726389 1.970285385 2.663406898 2.370369416 2.262590692 2.386146064 2.498612224 1.561337432 2.634763027 2.604203479 1.938739505 2.530432713 2.238328253 2.17992117 1.692041279 2.215697847 2.834601544 2.321021146 2.614138347 2.445331961 2.30549161 2.59725118 2.153306699 2.678111808 2.538764952 3.013266478 2.887989027 2.610519917 2.8253312 2.863566334 2.587198377 2.613772573 2.286740524 2.953214066 2.779484716 2.659005594 4.623938021 2.570596718 2.578053856 2.59907207 2.325177806 2.245925371 2.953240917 1.948657357 2.319258488 2.897947812 2.571103183 2.81589298 2.586003499 2.772134959 2.254988209 2.784570821 2.547252885 2.6724467 3.341990918 2.244993874 2.123074019 2.232808098 2.515270403 2.429646329 2.606400653 1.956534868 3.073701282 2.100119447 2.885145465 2.28801278 2.079601982 2.466324921 2.785026616 2.301952689 2.583347471 2.670245547 2.493408512 2.89871555 2.477852271 2.54334594 3.24241441 2.646292723 2.466357967 2.033040561 2.180068855 2.276417005 3.09593375 2.438593061 2.706385157 2.56307912 2.929719341 3.93406122 2.34009208 2.756388407 2.459767424 3.213181045 2.433406222 2.408220656 2.384472035 2.659544564 2.407154424 2.261446267 3.184473085 2.077957984 2.327761686 2.062426974 2.910186598 2.608083845 2.792070977 2.430369416 2.72973548 3.098479212 2.640185226 1.995772517 2.157458076 2.455214103 2.07458216 2.653725231 2.503081516 2.618306458 2.715517341 1.960427034 2.527049501 1.713085937 2.54321456 2.466235969 2.868372029 2.501821826 2.306717257 2.816628213 2.330832561 2.385750806 2.904972795 2.593434038 2.427953995 2.750303123 2.096912794 2.992239153 2.391295837 2.496577622 2.686706987 1.889772171 2.234535129 2.629560524 2.813405244 2.203350051 2.39888909 2.665716612 2.644623088 2.52601713 2.02023667 2.050065151 2.484199937 3.115512122 2.333378083 2.429982621 2.649608213 4.585365401

ENSG00000173208.3 ABCD2 0.116454458 0.196415522 0.181782051 0.08382065 0.231144375 0.626694924 0.281425776 0.48977672 1.02049149 0.18676486 0.191722018 0.139935983 0.772838463 0.574530995 0.512855283 1.083274202 0.415636914 0.683377117 0.264661691 0.417175312 0.418549347 0.188234397 0.308769559 0.121422769 0.323960955 0.258661301 0.526318644 0.106811908 0.124296606 0.273216651 0.297516676 0.087951826 0.035394774 0.150534646 0.110241559 0.201000687 0.204930813 0.17433599 0.01229091 0.053211377 0.208999501 0.067373049 0.223129634 0.551265618 0.061786377 0.163619671 0.163885014 0.090997444 0.178045147 0.134344156 0.151125312 0.169768086 0.078459083 0.149224991 0.111378401 0.078198781 0.018929548 0.009858951 0.051221547 0.076857075 0.241786754 0.112853788 0.230317154 0.040805623 0.170745679 0.20000187 0.113333775 0.054674507 0.321606133 0.06413197 0.13549244 0.105619953 0.154351002 0.322478814 0.788794362 0.060889896 0.115937008 0.223400879 0.047275245 0.40060632 0.180856168 0.211859454 0.094481334 0.177557942 0.103333499 0.148832884 0.086370091 0.18745459 0.179759622 0.053460336 0.214118626 0.067370812 0.098048725 0.222974804 0.040868515 0.079718784 0.455782675 0.16930083 0.255763622 0.454149255 0.185001853 0.113578383 0.206083431 0.150774598 0.093401672 0.144092797 0.179678121 0.198693086 0.319318625 0.191287901 0.152305599 0.103067381 0.26283458 0.228637376 0.281364631 0.208343964 0.313416052 0.121809796 0.065267969 0.087495575 0.407508117 0.356378595 0.129686964 0.281940597 0.3702367 0.079686704 0.247733454 0.084495004 0.225395247 0.130858882 0.056983986 0.079034718 0.040959346 0.085689873 0.043068336 0.327548111 0.044534444 0.051866876 0.17109228 0.013931741 0.188399152 0.219650147 0.152490597 0.206423562 0.356387463 0.143325651 0.272873407 0.135405319 0.332214469 0.202772865 0.174063438 0.078473834 0.201972733 0.372477066 0.114361155 0.194585644 0.11610671 0.293228153 0.101653666 0.431064382 0.05407273 0.149749535 0.081281338 0.200712039 0.156014805 0 0.146265127 0.18509042 0.144362233 0.080807123 0.007659944 0.042201842 0.190624571 0.025886264 0.034998203 0.107182521 0.098920349 0.13033398 0.068259577 0.2444922 0.108014955 0.230102619 0.146745053 0.236937647 0.166760679 0.259720442 0.178985173 0.252167908 0.030608572 0.201195918 0.223712716 0.20680529 0.154978065 0.189078437 0.077272415 0.037689312 0.01446889 0.031874646 0.02010125 0.117395078 0.057137086 0.146653247 0.053356318 0.070606661 0.031803627 0.082462834 0.023170185 0.16329077 0.066164336 0.087283786 0.169068958 0.045411605 0.264854539 0.193558103 0.025775655 0.109250487 0.217186159 0.176536892 0.049844925 0.252866321 0.081349272 0.137338629 0.042653368 0.130667821 0.068692192 0.134992066 0.064230007 0.215761476 0.227209467 0.0843972 0.017282315 0.066835918 0.086499966 0.070666765 0.109074604 0.112643991 0.241680981 0.08157745 0.240610694 0.045134832 0.127218821 0.040529889 0.042580982 0.093260138 0.154779547 0.056486869 0.111827205 0.031085338 0.117166693 1.332366509 0.011570568 0.22589352 0.111731597 0.171716812 0.069960914 0.064777195 0.090113122 0.037169772 0.186953285 0.163999509 0.390315218 0.195970027 0.16152784 0.117689254 0.700289944 0.193976074 0.049232339 0.266644843 0.15355142 0.282219694 0.118973718 0.10200358 0 0.302760495 0.081921903 0.065895244 0.100234491 0.153645484 0.054313272 0.228174722 0.077899546 0.108038117 0.685400139 0.03526171 0.121336594 0.102523821 0.066657956 0.10650692 0.517934769 0.581571881 0.09909246 0.196364372 0.018974037 0.279101148 0.108542984 0.036107854 0.008341173 0.087029368 0.032721736 0.075740998 0.01948656 0.042377949 0.062778984 0.030160631 0.138117159 0.205827974 0.237466249 0.298306681 0.165307525 0.233568729 0.147398356 0.126439544 0.077477862 0.165841066 0.16346368 0.050159579 0.089536512 0.127721451 0.024781412 0.070704575 0.08634202 0.070715336 0.099274897 0.056875637 0.513896542 0.295162163 0.088584634 0.130193982 0.088090723 0.163181338 0.069724665 0.071501575 0.03962715 0.176390032 0.093575876 0.071933959 0.08874582 0.085091363 0.004807947 0.100201371 0.080092796 0.099229024 0.119197157 0.004840012 0.042361687 0.161530441 0.040054943 0.246365939 0.1421376 0.070404614 0.013019442 0.074903216 0.111688933 0.039022994 0.134751551 0.546257808 0.017352812 0.069315055 0.095451642 0.081817727 0.115986504 0.068755496 0.010108655 0.077111608 0.059295847 0.028951336 0.149509627 0.175449911 0.022449282 0.039625309 0.016115566 0.02855535 0.193405974 0.241323434 0.020292239 0.025462588 0.011921023 0.051138962 0.093710827 0.073761326 0.097210854 0.036910558 0.293521195 0.089530278 0.094256934 0.184406175 0.058243457 0.063786443 0.0474402 0.059288419 0.101913914 0.070318859 0.019852635 0.148521189 0.125729506 0.043121344 0.073963425 0.010566664 0.121765583 0.22993021 0.109296577 0.004651696 0.171067968 0.129270906 0.011032218 0.131249839 0.003717463 0.047836776 0.011727169 0 0.033719795 0.022996291 0.109697634 0.112820162 0.173428784 0.135952207 0.269134383 0.010683891 0.097235149 0.148655006 0.122730515 0.054189478 0.037796771 0.191572186 0.231277095 0.204181929 0.074556542 0.168542006 0.050586723 0.107192932 0.20240401 0.164308614 0.343053065 0.105779961 0.033132888 0.158911565 0.140181224 0.094587864 0.050511943 0.130795676 0.099547639 0.211517399 0.111026696 0.069649575 0.038329815 0.066294995 0.157697338 0.207016453 0.255110072 0.03161162 0.076627671 0.020583372 0.04595327 0.049931929 0.082529218 0.043266326 0.012289752 0.023079751 0.117575328 0.011592756 0.051997451 0.102646859 0.282857635 0.025981564 0.677422161 0.076267727 0.280663893 0.115191697 0.074432881 0.05507672 0.088732745 0.059972987 0.021808022 0.074190531 0.057098486 0.011642177 0.019964795 0.044072675 0.232081546 0.18660196 0.172050411 0.004716949 0.244226257 0.400470873 0.168745329 0.049123748 0.022453246 0.035969857 0.080375416 0.139445103 0.12067682 0.018456364 0.004348143 0.148609869 0.102319715 0.051261815 0.052029675 0.224925233 0.139873653 0.0360475 0.234704943 0.14154268 0.120463391 0.021797758 0.02197913 0.168307935 0.128741924 0.088601087 0.190162553 0.105681345 0.183075613 0.040240882 0.02599509 0.016928806 0.094821578 0.061290044 0.017435037 0.020653294 0.095247055 0.106663295 0.044588734 0.190766997 0.275965465 0.097250082 0.167566025 0.104907727 0.21892659 0.314697961 0.16825544 0.143632779 0.30049539 0.045805302 0.050411554 0.167648205 0.147528128 0.048780448 0.053763261 0.460289477 0.261892535 0.065338953 0.145200738 0.100021602 0.286880323 0.322986282 0.170795099 0.022092421 0.085444047 0.14636129

ENSG00000117528.10 ABCD3 3.072673395 3.057169733 3.297037721 2.530559891 3.41301559 2.919784384 3.692169851 3.5960354 3.587544449 3.39402855 3.070515401 3.555261677 3.427884256 3.393815116 3.5095711 3.591169618 3.506314393 3.044400567 3.380418144 3.675079116 3.540217544 3.670942308 3.492283891 3.462501325 3.444182916 3.485740569 2.892829593 2.908759732 3.225186223 2.975201523 3.118971524 2.816698103 2.85167001 3.001142142 2.897034626 2.885293278 2.830233036 2.712076082 3.793542838 3.802455569 3.919254881 3.941389321 3.162019117 3.43930147 3.960481275 3.095881001 3.249274564 3.888289762 3.049370635 3.045289257 3.458022732 2.837523579 3.022058417 2.58195465 3.192420395 2.887271143 2.284118412 3.202605584 3.273217491 2.930782886 2.434191038 3.026787612 2.615496396 3.109370844 3.246557895 3.156262033 3.007208118 3.307367195 3.135766155 2.909770843 2.99157593 3.179503789 3.278413578 3.187691044 3.089358332 3.216497921 2.667493063 2.623066722 2.863058623 3.298543838 3.391348271 3.020007203 3.3372562 3.625839607 3.232628761 3.193944204 2.534854742 2.843081666 3.146418251 2.596196321 3.43370567 3.154734627 2.513807797 2.914714548 2.8927848 2.91723592 3.410436912 3.373372406 3.340435256 3.557039801 3.34755636 3.226249389 3.169347994 3.129404625 3.393998217 3.464488245 3.744745294 3.600820712 3.444255422 2.938548856 3.281038679 3.008253278 3.148615743 3.457927202 3.528660237 3.575514733 3.390007963 3.007286213 3.456055017 3.071478752 3.506225454 3.548806255 2.932935631 3.617097479 3.49008149 3.13402115 3.09390418 3.277654478 3.569906424 3.116017955 2.977271213 3.54943814 2.761264882 2.027374637 2.890023725 3.309378436 2.873598107 2.546671882 2.83161586 1.814376537 3.275562555 2.638283623 3.188984783 3.583571881 3.273658325 3.249708237 3.24106665 2.973320318 3.243577583 3.169142228 3.233647423 3.421521722 3.168116388 3.039786033 3.109358258 3.011824849 3.072324145 3.411555734 3.298222959 2.603080968 3.132785007 3.063143984 3.087045241 3.000717562 2.675842305 2.706471987 2.184016811 3.305362756 2.857274337 2.6134508 2.359449623 3.317148614 3.248830358 3.099344726 3.247878181 2.849809881 2.61136485 2.619881885 2.836876433 2.848267635 2.708710526 3.124254344 2.884666284 2.806783282 2.368117771 2.678797388 3.065645215 2.986430628 2.96267327 2.908121512 2.441083386 2.920510446 3.20344769 3.225499816 2.8889827 3.950729514 2.533960976 2.392427175 2.847684509 2.861811662 2.820462468 2.949096443 2.933181003 1.707822782 2.902125778 3.067754107 2.183880769 1.950013644 3.029913046 2.764509414 2.841730205 2.952606697 2.695915221 2.286601396 2.830636155 3.66070882 3.449067251 2.848697584 2.898814204 3.094813709 1.802053266 3.174848669 2.947799924 2.338013813 2.961708729 2.571530944 2.069667153 3.188064666 2.901302995 3.296485663 2.463569734 2.505665445 2.886282498 3.039299036 2.80302691 2.52598873 3.311062937 3.188753226 2.9484203 2.552526251 2.952899635 2.422532079 3.109673453 3.22388833 2.99662215 2.268061955 2.847346661 3.290118673 2.286620085 2.769992027 3.106697549 3.199660198 2.980119578 3.21300588 3.436825758 3.402039412 3.313404668 2.94494322 3.10694709 3.687213644 3.175764826 2.482865302 3.009674401 3.025047097 2.581834633 3.309138964 2.929709466 2.611916648 2.740046906 3.359012868 3.095487372 2.705832186 1.745577278 3.369794463 3.175427863 3.188932791 2.868892131 3.163394198 3.13194742 3.316393008 3.272830053 2.334238115 2.642265394 2.951786924 2.766454396 2.616962888 2.860625447 3.091616467 2.487225701 2.706684061 3.171754565 2.656040177 3.255151306 3.137047954 2.644073819 2.187779662 2.649006896 2.687608478 2.548034423 2.497245927 2.261091123 1.996289184 2.402038556 2.659772904 2.818694022 2.665242919 3.077255794 3.054602146 3.116731295 2.925915775 2.144376425 2.783831758 2.953925288 3.025603348 3.133245894 2.006604705 2.544463228 2.621747811 1.901793578 2.169176424 2.184025298 2.283258063 2.172885102 2.71105901 2.689700828 3.137093291 2.991129084 3.35848406 3.087386974 2.782653987 2.972090417 2.622367083 2.913695939 2.717090403 3.084131713 2.666656488 2.542526373 2.693942745 3.049786968 2.782571228 2.319452384 2.65308065 2.723794278 2.703053917 2.834093048 3.066966794 3.013828607 2.66763835 3.37480614 2.874011868 2.595492132 2.631409454 2.656479722 2.781935308 2.998759922 2.7597216 3.347062342 2.994373385 2.326843867 2.572687382 3.225620362 2.666329604 2.982565499 3.024798671 2.686812727 2.465014807 2.805835175 2.744209715 3.777525073 2.847322786 2.435726712 2.292006038 3.320928762 2.864980726 1.798920075 2.497209631 2.487349323 1.635221095 2.807524971 2.43508313 2.494453806 2.750525588 2.634515389 2.853568631 2.719018392 2.936027018 2.692684609 2.609670507 2.974720252 1.782078466 3.23966701 2.977990414 2.286215543 2.765729401 3.205061949 3.165143634 2.976800479 2.31421177 2.596195798 3.043141726 3.088370659 1.888858245 2.999180237 3.068853612 2.226809407 2.651202665 2.364697733 3.06543008 2.250372156 1.838058416 1.799844312 1.649915731 1.97939361 2.730394464 3.112551419 3.037263735 2.948326414 2.726355538 2.453579897 2.571497646 3.13589247 2.667234738 2.482574912 3.036384291 3.066060747 3.009363542 3.177959256 2.32666128 2.348724982 3.333305215 4.053288479 2.564870266 2.598276132 2.60518751 2.892311101 3.023425369 2.97906167 2.703365383 2.119816823 2.627978492 2.927563725 2.499646837 2.982866772 3.003060531 3.852030378 2.073412365 3.280661327 2.448649672 2.596439481 2.745322825 2.229967422 2.344861468 3.271544712 2.770200247 3.174684178 2.860175788 2.795798235 3.193416757 2.771725152 2.375124426 2.943273647 2.301990043 3.07853662 2.186487216 3.083474021 2.931613314 2.661562277 2.14774811 3.695432091 2.76007756 2.543910542 2.824072545 2.361890166 2.314365785 2.710989868 2.397383867 2.377549522 2.839578626 2.872372114 3.126151711 2.734388549 2.254331043 3.344940843 2.896243964 2.614745629 2.534326327 2.921643373 2.883165159 3.205797582 2.453641461 2.966505535 2.751900491 2.139375713 2.342705434 2.812683548 2.716456508 2.753825527 2.747684844 3.042039676 3.247704367 3.069808225 2.644095306 2.565109354 2.976970835 3.533618935 2.882196411 3.475878342 2.678083285 2.781863678 2.539862593 3.134814478 2.723042521 2.045600548 2.778362196 2.526251993 2.624814427 2.672160654 2.589322093 3.005478452 2.724693531 2.716521488 3.132372147 2.880796236 2.651646978 2.923075045 2.382450602 2.915657921 2.787148987 2.396420451 2.627727219 2.622446936 3.305950836 2.505305861 2.976899238 2.338559086 2.226668643 2.232680673 2.789868062 2.485133508 2.142557756 2.940398072 3.261563646 3.030533917 2.872460033 3.18487176 2.252124194 2.026844684 4.004616758

ENSG00000119688.19 ABCD4 2.410282126 2.274285341 2.05747831 2.124934756 2.222748711 1.393189282 2.113429799 1.93430505 2.098538162 1.963964188 2.271598746 2.175811191 1.582488011 2.005750599 1.90831989 1.964278901 2.313571985 1.991194651 2.273129416 2.2662483 2.032693591 2.311997073 2.398801904 1.949159508 2.288107459 2.163867404 2.129327342 1.83485051 2.02447008 1.722843106 1.920881364 2.114834512 1.544983577 1.918351449 2.13349046 1.668600344 1.731153148 1.496994629 1.832828603 1.404040117 1.863274038 2.031384246 1.81948425 1.854278119 1.784195038 1.982453715 2.117479398 1.805853679 2.263334814 2.078991279 2.305599117 2.52122938 2.217323872 2.271298421 2.390874264 2.252926255 2.074920572 1.93244253 2.22412583 2.228006468 2.257564349 2.318120989 2.232737436 1.824065785 2.181905539 2.181834538 2.375476181 2.01431846 2.368645694 2.161343845 2.530473374 2.331510542 2.047841912 2.184345503 2.200068828 2.213091097 1.949228182 2.198095911 2.013876078 2.221820529 1.966314341 2.139478576 2.359433436 2.349273277 1.680260267 2.033511546 2.050950855 2.060101648 1.848808878 2.174456969 1.956693883 2.458767078 2.276741525 2.390612997 2.58682175 2.088240428 1.856059074 2.288358397 2.143688122 1.852655004 2.019967587 2.305915264 2.332443963 2.245619581 1.890421838 1.993236785 1.972634923 2.206509754 2.095629112 2.007700841 2.016829541 2.443072677 2.108036939 2.106350349 1.889715691 1.907545842 1.672636344 1.839131126 1.969802614 2.30626525 2.131328618 1.985417901 2.152443804 2.093848597 1.921981057 2.298238937 2.204562222 1.820294687 1.997590014 2.080136254 2.045612045 1.630248928 2.175950422 2.159653081 2.047144677 2.186661269 1.794124736 1.712653887 2.140156772 2.270273758 2.312600036 2.178325571 1.828372301 1.891866334 2.104774483 2.17444152 2.121318983 2.240539036 2.36607326 2.064496497 2.008025505 1.736187768 2.086861841 2.199138083 1.977569409 2.063755602 2.111629663 1.998337538 1.969980206 2.408047043 2.479010574 2.009202106 2.236816332 2.500688644 2.570637481 2.393508467 1.693739247 2.517725855 2.463860692 2.475975046 2.654041499 2.498925974 2.399585405 2.302564133 2.021072111 2.19672131 2.341056743 2.399125447 2.256962797 2.422540497 2.533313766 2.277785277 2.433749075 2.972670833 2.760436964 2.172449873 2.215541833 2.357230026 2.255269395 2.259814463 2.193202174 2.20543393 2.194634945 2.252043828 1.906946105 2.050350808 2.502192217 2.380915966 2.515008377 1.721734373 2.292019206 1.951682393 1.88168134 1.541010964 2.01402068 2.400446636 1.651764406 2.28105139 1.939031332 2.116492267 2.169830645 2.351119063 2.198320622 1.927470837 1.569086123 1.953973651 2.026040824 1.865589892 1.728892414 1.936508347 1.375350325 1.963422673 1.915825167 1.77564811 1.942150692 1.830494993 2.7389213 1.83490125 2.023125334 1.88112232 1.663999219 1.488763261 1.709248359 2.152579614 1.795496516 1.998370528 1.555174959 2.170991746 1.651309817 2.068715112 2.504438977 2.142464851 2.604587431 2.700807685 2.393082593 2.344310876 2.040753881 2.244649249 2.217587682 2.620652015 2.086746098 1.996510577 2.355062119 1.650762401 1.987702771 2.086762567 2.07431217 2.052923945 2.325568722 2.155161548 1.862743311 1.863915469 1.596744267 2.220440256 2.508884115 2.040448448 2.225182552 2.164047553 2.281140599 2.128237516 2.192857561 1.908242891 1.686595431 2.509365178 2.190314477 2.34576829 2.319660861 2.041843796 2.112566835 2.166273318 1.699346667 2.414997306 1.442959133 2.509503533 2.38306524 2.048509537 2.036160517 2.247060903 1.688787658 2.461248693 2.171839273 2.108504132 2.203807154 2.064692401 2.470442508 3.223516285 2.022428795 2.633216257 2.58881158 2.611623877 2.765402855 2.435215681 2.140104215 2.322078984 2.097342932 2.355186807 2.26698434 2.14185174 2.267025732 2.050948176 1.146016458 1.805534206 1.534134099 2.333032893 1.877701767 2.345817965 2.620788107 2.558096684 2.993857798 1.736427286 2.421725198 2.7973138 2.647691739 2.334639663 2.324200844 2.455999183 2.363570343 1.83678223 2.373647466 2.431528246 2.266656717 2.262863227 2.458630081 2.446653987 2.316513275 2.145830558 2.119518056 2.36895433 2.3393356 1.985128081 1.963957355 2.584356252 2.283445934 2.07153028 2.126991091 2.469619671 1.866491881 1.998742878 2.061097138 2.299791792 2.386319156 2.475534883 2.085930509 2.314118017 2.375728206 2.585352363 1.787974013 2.321979325 2.003273229 1.961940282 2.410194017 2.030591315 1.613904065 2.008119804 2.428951729 2.728973503 2.437793728 2.565978261 2.540863044 2.026750061 1.987907107 2.475706069 2.770869709 2.419621674 1.786003788 2.64027134 2.166178102 2.333866914 2.387161118 2.345814493 2.514596698 2.462314007 2.254238274 1.803522721 2.354967662 2.345622765 2.278244618 2.364109215 2.222158129 2.07998783 1.878284579 1.865982034 2.186401906 2.058334949 2.418795474 2.272630156 2.07814096 2.392725381 2.193235772 2.362926021 2.517634589 2.342256982 2.328322074 2.300700201 2.217370806 1.925503223 1.8781332 1.857330708 1.993276925 2.58612342 2.453541955 2.400366989 2.551645905 2.512523346 2.514948752 2.057734289 2.520946311 2.442625965 1.949377821 2.398091609 2.067670528 2.009802703 2.265945935 2.438863438 1.846977258 2.126384232 2.006596024 2.566073186 2.309058096 2.318605566 2.310505701 2.354482102 2.231170852 2.441197463 2.309775044 2.051255705 2.524737972 2.163794792 2.312353433 2.270649586 2.528398828 2.41775588 2.072694293 2.407181214 2.139908066 2.268336915 2.25171352 2.318480925 2.651353553 1.847202013 2.399406011 1.940798742 2.145221331 2.025823627 1.599221444 2.209124636 2.108925078 2.241329081 2.657495603 1.362125071 2.355880078 2.417534441 2.451775599 2.202104831 2.40974092 2.500126436 2.069692039 2.165495358 1.995456541 2.199992155 2.172389593 2.248909507 2.246484898 2.175404867 2.220439858 2.509597103 1.974848361 2.026304031 2.74773319 2.376004708 2.504031407 2.523605003 2.200034417 2.480224468 2.285768964 2.531072046 2.609368321 2.410225298 2.245054282 2.228431152 2.545533351 2.384684431 2.494640038 2.222624999 2.403374798 2.395342575 2.784668442 2.227797368 2.58685501 2.660099128 2.168340812 2.12966275 2.219461956 1.953095421 2.347591347 2.320474984 1.985726418 2.478189281 2.375443293 2.234173759 2.555951393 2.21552233 2.516890669 2.332453322 2.247777458 2.111809266 2.082280357 2.07221824 2.278465413 2.342349685 2.299705095 2.538844181 2.640188939 2.075341544 1.881047566 1.966325032 2.524433492 2.09036536 2.491175057 2.799336604 2.196088858 2.12432909 1.871981533 2.243908801 1.904455755 2.150220043 2.264310207 2.05257914 2.448427537 2.36402186 2.360678988 1.971128484 2.194351062 2.498055068 1.590525431 2.035357698 2.244872237 2.272940325
[truncated: 260,510 more chars]
